# Rapid Access to 1,2,3,4-Tetrasubstituted Benzenes and 3‑Alkenyl-1,2-dihydropyridines

**DOI:** 10.1021/acsomega.6c02327

**Published:** 2026-05-04

**Authors:** Buse Aysen Dundar Ozdogan, Metin Zora

**Affiliations:** Department of Chemistry, Faculty of Arts and Science, 52984 Middle East Technical University, 06800 Ankara, Turkey

## Abstract

Herein, we report unprecedented protocols for the synthesis of 1,2,3,4-tetrasubstituted benzenes and 3-alkenyl-1,2-dihydropyridines from readily available *N*-methyl-*N*-propargyl β-enaminones. When subjected to the reaction with dialkyl acetylenedicarboxylates in refluxing toluene under gold catalysis, *N*-methyl-*N*-propargyl β-enaminones afforded 1,2,3,4-tetrasubstituted benzenes. On the other hand, the reaction of *N*-methyl-*N*-propargyl β-enaminones with dialkyl acetylenedicarboxylates in refluxing acetonitrile under copper catalysis formed 3-alkenyl-1,2-dihydropyridines as the major product, accompanied by 1,2,3,4-tetrasubstituted benzenes as the minor product. An internal alkyne-tethered *N*-methyl-*N*-propargyl β-enaminone yielded a pentasubstituted benzene derivative under both gold and copper catalysis. The skeletal diversity of the synthesized 1,2,3,4-tetrasubstituted benzenes and 3-alkenyl-1,2-dihydropyridines may be of use in medicinal and pharmaceutical chemistry as new and novel molecular entities and structural leads.

## Graphical abstract

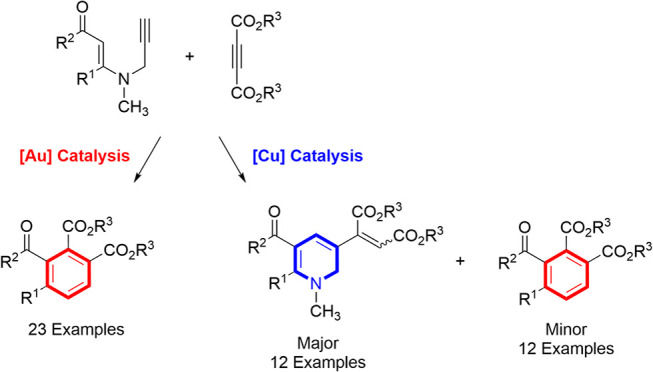

## Introduction

1,2,3,4-Tetrasubstituted benzenes are important scaffolds in medicinal chemistry since they provide a rigid, planar, and electronically delocalized core that can place the substituents in defined orientations for binding to biological targets.^1^ In fact, 1,2,3,4-tetrasubstituted benzenes have four contiguous (adjacent) substituents around a single aromatic core, which may allow dense functionalization of diverse nature substituents (such as H–bond donors/acceptors, lipophilic groups, and polar groups), enabling fine-tuning of bonding, lipophilicity, solubility, and selectivity.^1,2^ 1,2,3,4-Tetrasubstituted benzene rings are frequently present in the structures of various drugs, natural products, and agrochemicals, as depicted in Figure 1. *Cobimetinib* is an anticancer medication used to treat melanoma and histiocytic neoplasms.^3^ *Belzutifan* is employed to treat von Hippel-Lindau (VHL) disease associated with kidney and pancreatic cancers as well as brain and spinal cord tumors.^4^ *Tembotrione* is a broad-spectrum post-emergence herbicide used for the control of broad leaf and grossy weeds in corn.^5^ *Totarol* is naturally produced diterpene with antimicrobial and therapeutic properties.^6^ *S1319* is a marine sponge-derived β_2_-adrenoceptor agonist.^7^

**Figure 1:**
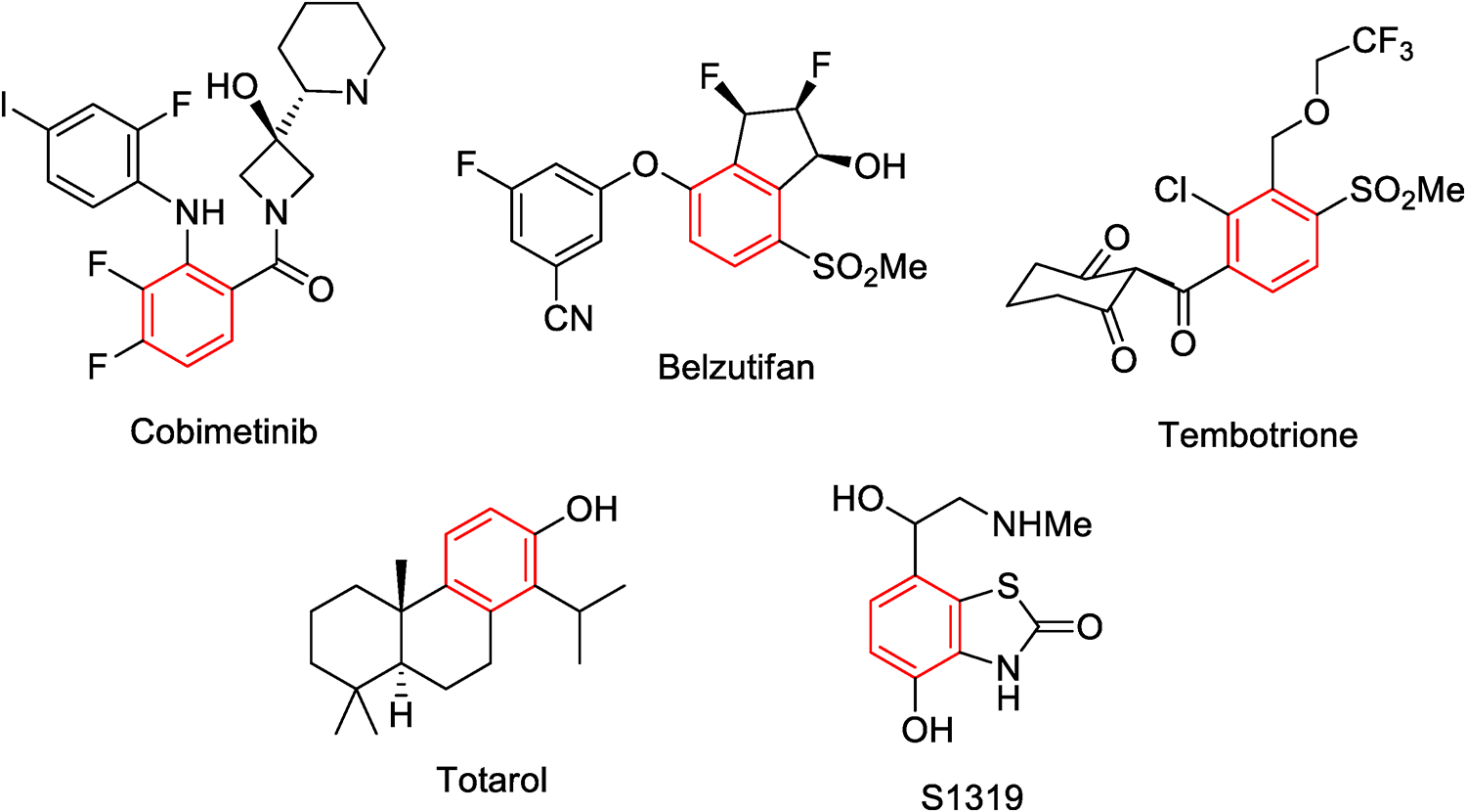
Representative examples of bioactive 1,2,3,4-tetrasubstituted benzenes.

1,2,3,4-Tetrasubstituted benzenes are generally synthesized by (i) electrophilic aromatic substitution (EAS) of trisubstituted benzenes,^8^ (ii) C–H bond activation strategy,^9^ (iii) vicinal difunctionalization of arynes,^10^ and (iv) [2+2+2] cycloaddition of alkynes and alkenes,^11^ and [4+2] cycloaddition (Diels–Alder reaction) of dienes and dienophiles.^12^ The first method often suffers from site selectivity. The second method requires the use of a directing group (DG). The third method obligates the employment of pre-difunctionalized precursors and strong bases for the generation of aryne intermediates. The fourth method necessitates control of regioselectivity for unsymmetrical substrates. In brief, some drawbacks of these protocols, such as the poor reactivity and regioselectivity, limit their practical application to some extent. In fact, the contiguous, sterically congested, and unsymmetrical 1,2,3,4-tetrasubstituted arenes, i.e., each substituent being different, are nontrivial to access via conventional methods. Therefore, there is a continuous and increasing interest in developing new methods for the synthesis of 1,2,3,4-tetrasubstituted arenes. Tanaka and co-workers developed a method for the synthesis of 1,2,3,4-tetrasubstituted benzenes from enol ether and two alkynes via [2+2+2] cycloaddition in the presence of a rhodium catalyst (Scheme 1a).^13^ Tsui and Nakamura, and Kuninobu and Takai research groups also reported a [2+2+2] cycloaddition between enol of 1,3-dicarbonyl compound and two moles of terminal alkynes using a manganese catalyst, leading to 1,2,3,4-tetrasubstituted benzenes (Scheme 1b).^14^ Dong and co-workers described a rapid entry to 1,2,3,4-tetrasubstituted benzenes through the elaboration of 2-iodo-1,2,4-trisubstituted arenes by Catellani reaction using a palladium/substituted-norbornene (Pd/subst-NBE) catalyst (Scheme 1c).^15^ Similarly, Lumb and Luan demonstrated that meta-substituted iodides are also effective coupling partners in Catellani reaction, which, upon treatment with a palladium/norbornene (Pd/NBE) catalyst, provides 1,2,3,4-tetrasubstituted benzenes through C–H bond functionalization (Scheme 1d).^16^ Cheong and Stuart research groups developed a strategy to synthesize 1,2,3,4-tetrasubstituted benzenes from 1,2,4-trisubstituted aryl­(Mes)­iodonium salts by vicinal difunctionalization of in situ formed aryne intermediates (Scheme 1e),^17^ complementary to Catellani chemistry.^18^ Ruffoni, Caldora, Leonori, and co-workers reported a three-step method for the conversion of 4-substituted pyridines into 2-cyano-1,2,3,4-tetrasubstituted benzenes (Scheme 1f).^19^ N-oxidation of pyridine, followed by photochemical deconstruction in the presence of an amine, yields a nitrile-bearing butadiene. Finally, its Diels–Alder reaction with alkynes and alkenes produces 1,2,3,4-tetrasubstituted benzonitriles (Scheme 1f).^19^ Although some methods have been developed and new variants continue to appear, regiocontrolled synthesis of 1,2,3,4-tetrasubstituted benzenes remains a significant challenge for organic chemists.

**Scheme 1:**
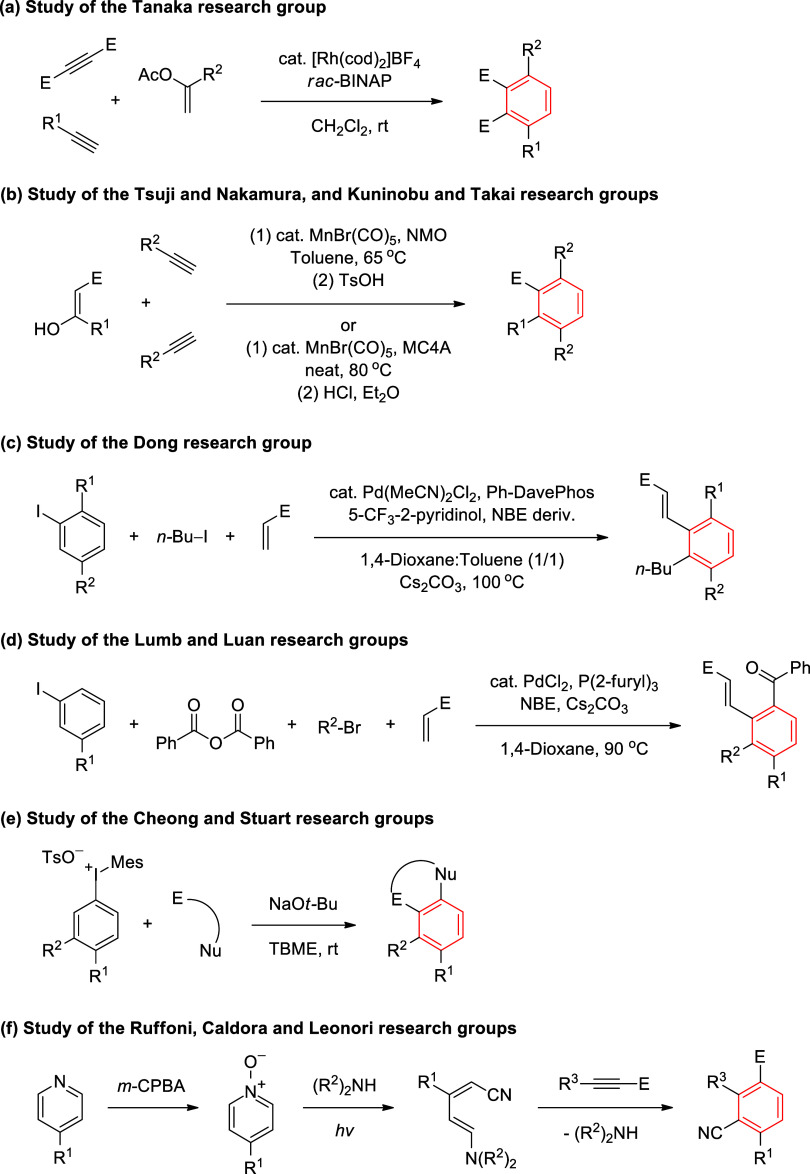
Synthetic Approaches for the Synthesis of 1,2,3,4-Tetrasubstituted Benzenes

1,2-Dihydropyridines are valuable scaffolds and reactive intermediates in medicinal chemistry since they are frequently used as the precursors to synthesize pyridines and piperidines through their oxidation and reduction reactions, respectively.^20^ Notably, pyridines and piperidines are among the most common structural components of pharmaceuticals and agrochemicals.^21^ In fact, as compared to their regioisomer 1,4-dihydropyridines,^22^ 1,2-dihydropyridines are less studied, possibly due to the limited availability of synthetic methods for their preparation.^20^ So, the potential of 1,2-dihydropyridines has been underrepresented in medicinal and pharmaceutical chemistry. Recently, their value as critical synthones (cyclic azadienes) for the synthesis of some alkaloids and drugs has been recognized.^20^ Diels–Alder reaction of 1,2-dihydropyridines with dienophiles affords isoquinuclidine (2-azabicyclo[2.2.2]­octane) ring systems, which are widely found in the structures of alkaloids such as *Ibogaine* (an antiaddictive and anticraving agent) and *Dioscorine* (a toxic central nervous system depressant and a nicotinic acetylcholine receptor modulator) (Figure 2).^23^ The anti-influenza drug *Oseltamivir phosphate* (Tamiflu) has been synthesized as well from a 1,2-dihydropyridine derivative through an isoquinuclidine intermediate (Figure 2).^24^ Notably, 1,2-dihydropyridines exhibit important biological activities,^22^ such as antiproliferative^25^ and anticancer^26^ properties. In general, 1,2-dihydropyridines are synthesized by Hantzsch reaction,^25^ reduction of *N*-activated pyridinium salts,^27^ and 6π-aza-electrocyclization of 1-azatrienes.^28^ Although many fruitful methods have recently been developed,^29^ there is a continuous need to develop new methodologies for the synthesis of 1,2-dihydropyridines since it may increase their potential and significance in the related fields.

**Figure 2:**
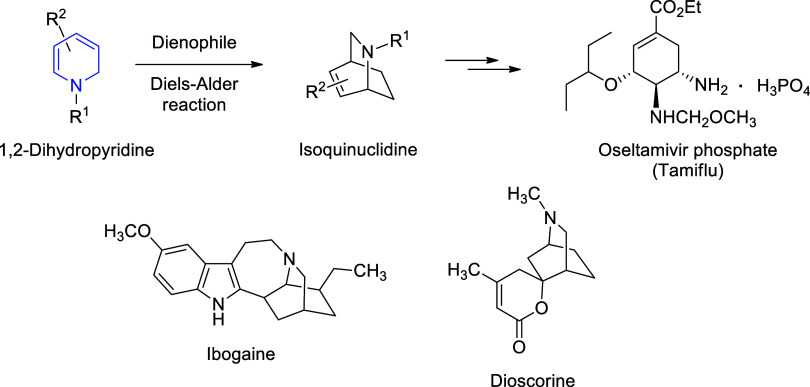
Synthesis of isoquinuclidines from 1,2-dihydropyridines via Diels–Alder reaction.

*N*-Propargylic β-enaminones have recently attracted attention as the reactive intermediates because, under suitable conditions, they readily produce five-, six-, and seven-membered heterocyclic compounds,^30^ including pyrroles, spiro-pyrroles, pyrrolines, pyridines, 1,4-oxazepines, and 1,4-thiazepines.^31^ When reacted with suitable reagents, *N*-propargylic β-enaminones, as well as *N*-propargylic β-enaminoesters, also produced 1,2-dihydropyridines via one-pot cyclization, as shown in Scheme 2. Saito, Hanzawa, and co-workers developed a method for the Au-catalyzed synthesis of 1,2-dihydropyridines from *N*-propargylic β-enaminones (Scheme 2a).^32^ Martins research group achieved the synthesis of 1,2-dihydropyridines under Ag catalysis (Scheme 2b).^33^ Wan research group reported the synthesis of 1,2-dihydropyridines and iodo-substituted derivatives from *N*-propargylic β-enaminoesters under thermal conditions (Scheme 2c).^34^ Oguri and co-workers demonstrated the synthesis of 1,2-dihydropyridines from both *N*-propargylic β-enaminones and β-enaminoesters under Cu catalysis (Scheme 2d).^35^ Pour research group described a rapid entry to 1,2-dihydropyridines from *N*-propargylic β-enaminoesters by using an Au/Ag cocatalyst system (Scheme 2e).^36^ An increasingly important aspect of these studies is to find new methods for synthesizing novel derivatives that may provide a new mode of action for the treatment of a specific disease.

**Scheme 2:**
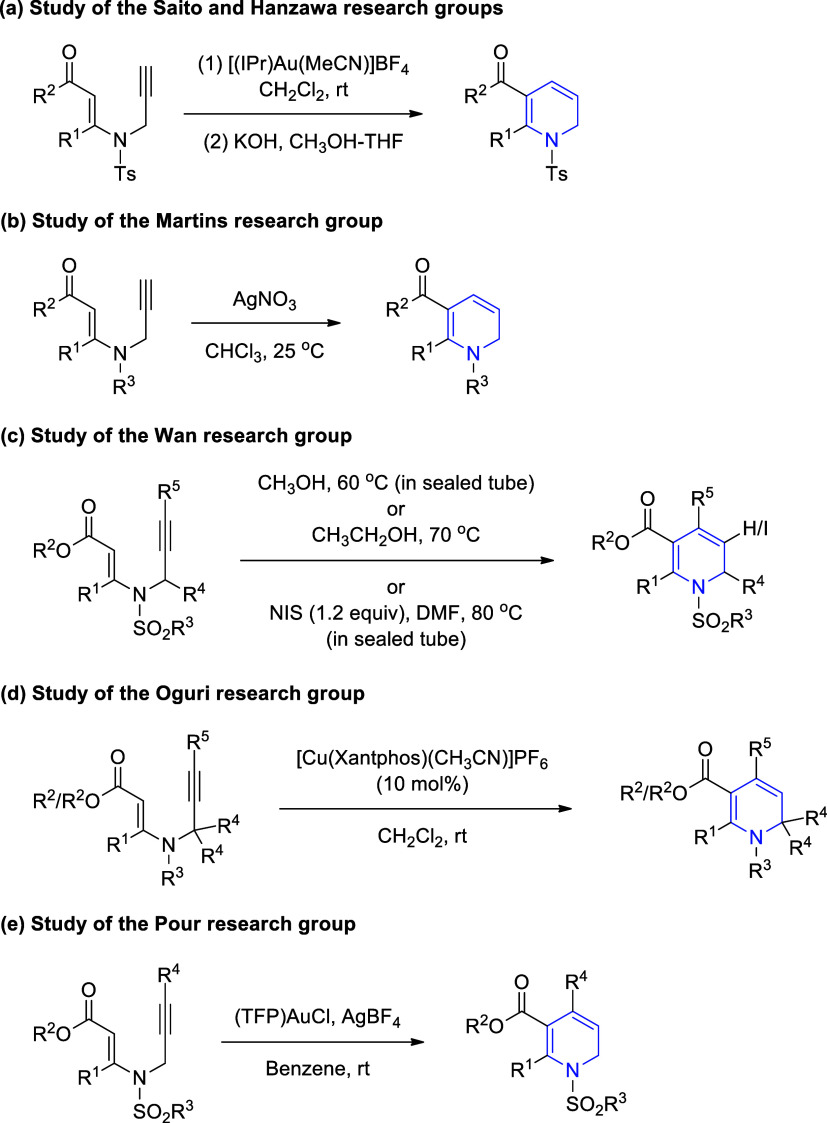
Synthesis of 1,2-Dihydropyridines from *N*-Propargylic β-Enaminones and β-Enaminoesters

Recently, Karunakar and co-workers investigated the reaction of *N*-propargylic β-enaminones with dialkyl acetylenedicarboxylates, which led to one-pot formation of 3-azabicyclo[4.1.0]­hepta-2,4-dienes, a cyclopropane-fused pyridine system (Scheme 3a).^37^ Sometimes, altering the backbone substitution pattern of the starting materials may influence the outcome of the reaction and new products can result from such reactions.^38^ Interestingly, the reaction of *N*-methyl-*N*-propargyl β-enaminones with dialkyl acetylenedicarboxylates was not studied. As part of our ongoing interest in developing new methods for the synthesis of potentially bioactive compounds, we have investigated the reaction between *N*-methyl-*N*-propargyl β-enaminones and dialkyl acetylenedicarboxylates (Scheme 3b). Interestingly, this reaction led to the formation of 1,2,3,4-tetrasubstituted benzenes and/or 3-alkenyl-1,2-dihydropyridines under the gold and copper catalysis. To the best of our knowledge, a carbocyclic product, 1,2,3,4-tetrasubstituted arene, resulted for the first time from the reactions of *N*-propargylic β-enaminones.^39^ Formation of 1,2-dihydropyridines from *N*-propargylic β-enaminones is well known, but the formation of 3-alkenyl-1,2-dihydropyridines is without precedent. Herein, we report the preliminary results of this study.

**Scheme 3:**
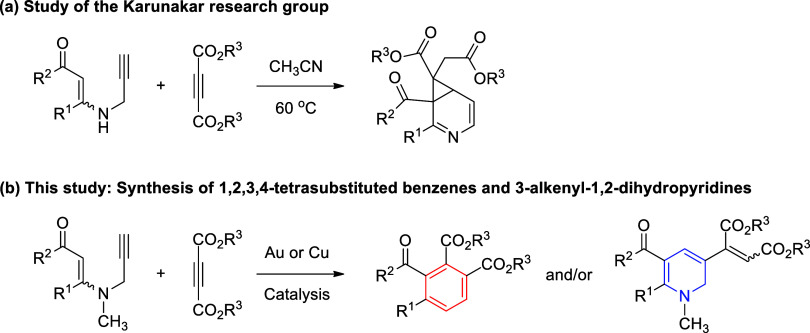
Strategies for the Synthesis of 3-Azabicyclo[4.1.0]­hepta-2,4-dienes, 1,2,3,4-Tetrasubstituted Benzenes, and 3-Alkenyl-1,2-dihydropyridines

## Results and Discussion

First, we synthesized the necessary starting materials, *N*-methyl-*N*-propargyl β-enaminone **3** derivatives, according to similar literature methods (Table 1).^31a,31c^ The reaction of α,β-alkynic ketones **1** with *N*-methylpropargylamine (**2**) in refluxing methanol yielded *N*-methyl-*N*-propargyl β-enaminones **3** (Table 1). By employing these reactions, we prepared 21 derivatives of *N*-methyl-*N*-propargyl β-enaminones **3**, the yields of which ranged between 8 and 69%. Notably, as compared with propargylamine, *N*-methylpropargylamine was less reactive and its reaction with α,β-alkynic ketones **1** took about twice as long. Moreover, it afforded the corresponding β-enaminones in relatively lower yields, along with some decomposition and reaction byproducts with no UV absorption, which could not be totally removed from the reaction’s target product. Furthermore, using a similar literature protocol,^31a,31c^ we synthesized one example of *N*-methyl-*N*-propargylic β-enaminone that contains internal alkyne functionality, specifically **3v**, from β-enaminone **3a** by the Sonogashira cross-coupling with iodobenzene under Pd catalysis (Table 1) (see the Experimental Section for details).

**Table 1:**
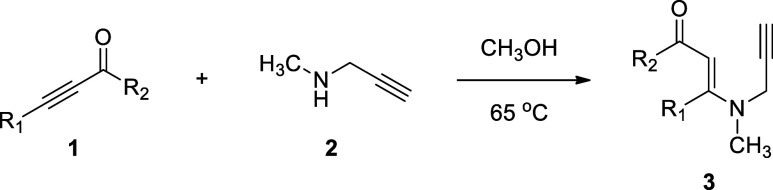

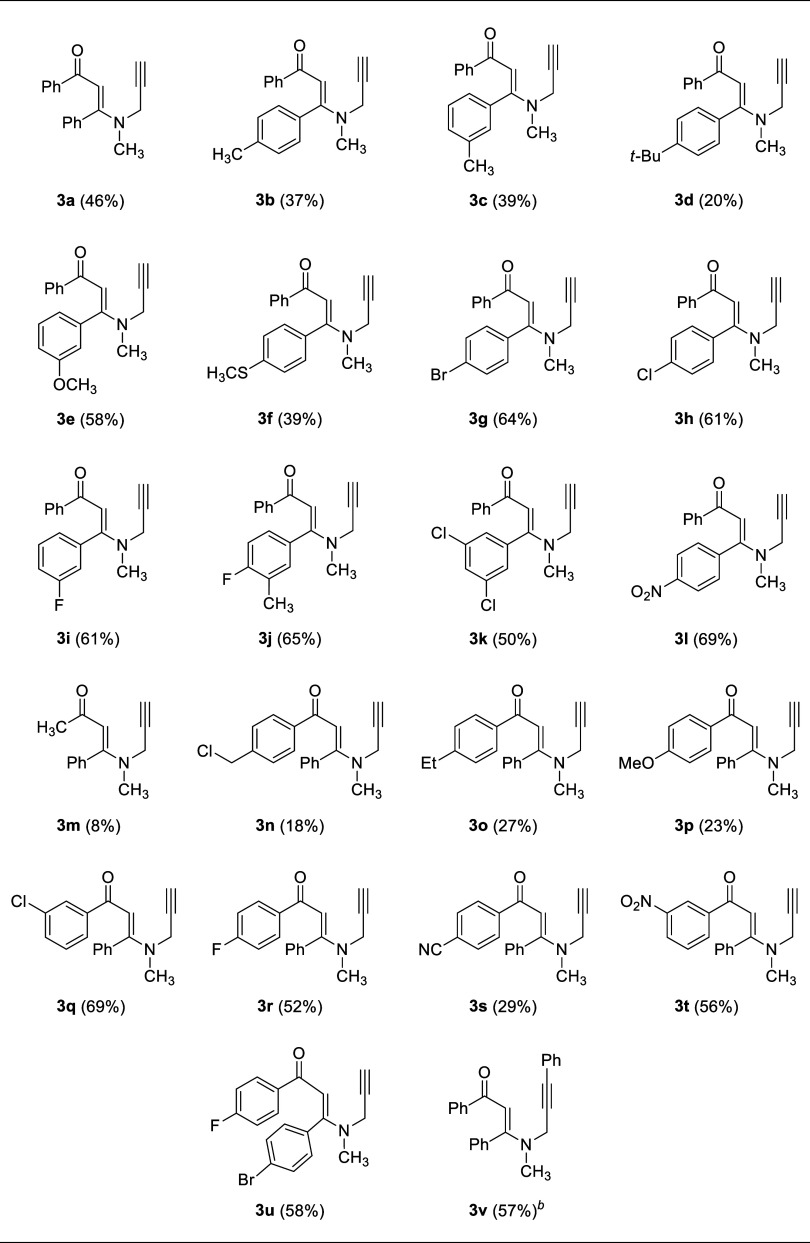
Synthesis of *N-*Methyl-*N-*propargyl β-enaminones **3** ^a^

a Isolated yields.

b This product was synthesized by the Sonogashira cross-coupling of β-enaminone **3a** with iodobenzene under Pd catalysis. For details, see the Experimental Section.

In general, *N*-propargylic β-enaminones form as the *Z* isomer because of an intramolecular hydrogen bond (N–H*···*O=C) in these compounds, which affects their tautomeric equilibria and stability. However, due to the absence of an N–H moiety, *N*-methyl-*N*-propargyl β-enaminones **3** can result from these reactions as either *E* or *Z* isomer, or a mixture of both isomers. In the ^1^H NMR spectra of β-enaminones **3**, only one singlet peak for vinylic hydrogen (C=C*H*) was observed around 6.0 ppm, indicating the formation of β-enaminones **3** as the single isomer. If β-enaminones **3** had formed as a mixture, there should have been another singlet peak in this region, belonging to the vinylic hydrogen of the other isomer, but such a peak was not observed. To figure out which isomer was formed, we took the NOESY spectrum of β-enaminone **3a** (see the Supporting Information). In the spectrum, a nuclear overhauser effect (NOE) was observed between vinylic hydrogen (C=C*H*) and methylene hydrogens (N–C*H*_2_-) as depicted in Figure 3a. A similar NOE correlation was also observed between vinylic hydrogen (C=C*H*) and methyl hydrogens (N–C*H*_3_) (Figure 3b). On the basis of these NOE interactions, the configuration of the double bond of β-enaminones **3** was assigned as the *E* stereochemistry. It is noteworthy that if a β-enaminone lacks an intramolecular hydrogen bond affecting its configuration, as in *N*-methyl-*N*-propargyl β-enaminones **3**, its *E* isomer is expected to be thermodynamically more stable than the corresponding *Z* isomer. Therefore, the formation of *N*-methyl-*N*-propargyl β-enaminones **3** as the *E* isomer is an expected result and is consistent with the observed NOE interactions.

**Figure 3:**
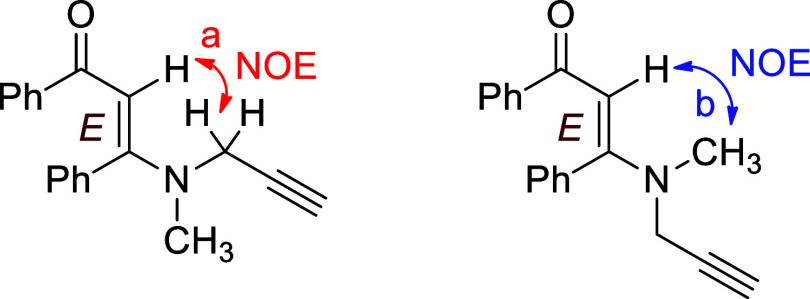
NOE Correlations Observed in the NOESY NMR Spectrum of β-Enaminone **3a**.

Next, we investigated the reaction of *N*-methyl-*N*-propargyl β-enaminone **3a** with dimethyl acetylenedicarboxylate (DMAD, **4a**) as a model reaction in various conditions (Table 2). In these reactions, a slightly excess amount of DMAD (1.3 equiv) was employed. Initially, we tried the reaction in refluxing acetonitrile, which produced a new product, namely, 1,2,3,4-tetrasubstituted benzene derivative **5a**, in very low yield (5%), along with recovered β-enaminone **3a** (15%) with some decomposition (Table 2, entry 1). This reaction generated few byproducts as well, but in very low amounts, one of which was tentatively assigned as the conjugate addition product of β-enaminone **3a** to DMAD (**4a**). Interestingly, this reaction produced a carbocyclic product, i.e., 1,2,3,4-tetrasubstituted benzene derivative **5a**. The structure of **5a** was further verified by the comparison of its spectral data with those reported previously for this compound.^40^ To our knowledge, the formation of such a product (**5a**) from these reactions is unprecedented. It is well known that the reactions of β-enaminones furnish the nitrogen-based heterocycles. Possibly, during the course of the reaction, a nitrogen-containing intermediate or compound is first produced, which then undergoes skeletal editing by the removal of the nitrogen atom moiety from the molecule to give a carbocyclic product, as it will be discussed in the mechanism. Subsequently, in order to increase the product yield, we tested the reaction in the presence of various gold catalysts. The reaction in the presence of AuCl_3_ (0.10 equiv) afforded product **5a** in 16% yield (Table 2, entry 2). The reaction was repeated using AgSbF_6_ (0.15 equiv) in addition to AuCl_3_, but the yield (19%) of product **5a** increased only slightly (Table 2, entry 3). Interestingly, there was no change in yield when AgOTf was used instead of AgSbF_6_ (Table 2, entry 4). The reaction with AuCl_3_/AgSbF_6_ was carried out in 1,2-dichloroethane (DCE) and dioxane as well, providing **5a** in 27 and 25% yields, respectively (Table 2, entries 5 and 6). Then, we turned our attention to the AuCl catalyst. First of all, we tested the reaction with AuCl (0.10 equiv) in acetonitrile, DCE, dioxane, toluene, and *p*-xylene (Table 2, entries 7–11), which afforded product **5a** in 21–32% yields, where the highest yield (32%) was obtained in toluene (Table 2, entry 10). The same reaction was also tested using AgSbF_6_ in addition to AuCl in acetonitrile, dioxane, and toluene (Table 2, entries 12–14). Of these, the reaction with toluene gave product **5a** in the highest yield (35%) (Table 2, entry 14). Since the highest yield so far was obtained with toluene, subsequent reactions were carried out with this solvent. The same reaction was carried out at lower temperatures as well. The reaction at room temperature did not produce any product, and the starting β-enaminone **3a** was recovered in 26% yield (Table 2, entry 15). However, the reaction at 50 °C yielded benzene derivative **5a** in 11% yield, along with recovered β-enaminone **3a** (25%) (Table 2, entry 16). The reaction was also performed with a lower AuCl/AgSbF_6_ ratio (0.05/0.10 equiv), but product **5a** from this reaction was obtained in a lower yield (32%) (Table 2, entry 17). The reaction was then tested using 2.0 and 3.0 equiv of DMAD while keeping the AuCl/AgSbF_6_ ratio constant, but these reactions formed product **5a** with 29 and 25% yields, respectively (Table 2, entries 18 and 19). Obviously, increasing the amount of DMAD reduced the product yield. Therefore, subsequent reactions were carried out using 1.3 equiv of DMAD. The reaction was also tried with AuI/AgSbF_6_, but the product was obtained with a 31% yield (Table 2, entry 20). Subsequently, the reaction was carried out in toluene at reflux with other gold catalysts, in the presence or absence of AgSbF_6_. For this purpose, C_18_H_33_AuClP, HAuCl_4_·3H_2_O, [(IMes)­AuCl], [(IPr)­AuCl], Et_3_PAuCl, Ph_3_PAuCH_3_, and JohnPhos AuCl catalysts were used (Table 2, entries 21–28). These reactions gave the expected product **5a** with yields ranging from 20 to 32%. In summary, the highest yield of 1,2,3,4-tetrasubstituted benzene **5a** was obtained in refluxing toluene with 1.3 equiv of DMAD in the presence of AuCl/AgSbF_6_ (0.10/0.15 equiv) catalyst system (Table 2, entry 14). Therefore, derivatization of 1,2,3,4-tetrasubstituted benzenes **5** was carried out under these conditions.

**Table 2:**
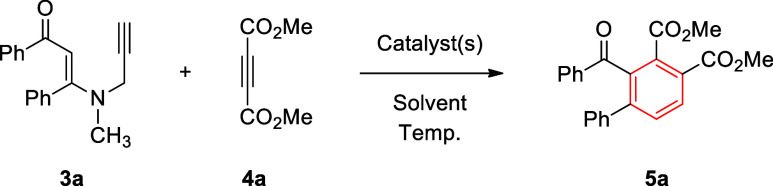
Optimization of the Reaction Conditions under Gold Catalysis^a^

entry	catalyst(s) (equiv)	DMAD (**4a**) (equiv)	solvent	temp. (°C)	yield of **5a** (%)
1	–	1.3	CH_3_CN	82	5
2	AuCl_3_ (0.10)	1.3	CH_3_CN	82	16
3	AuCl_3_/AgSbF_6_ (0.10/0.15)	1.3	CH_3_CN	82	19
4	AuCl_3_/AgOTf (0.10/0.15)	1.3	CH_3_CN	82	19
5	AuCl_3_/AgSbF_6_ (0.10/0.15)	1.3	DCE	83	27
6	AuCl_3_/AgSbF_6_ (0.10/0.15)	1.3	Dioxane	101	25
7	AuCl (0.10)	1.3	CH_3_CN	82	21
8	AuCl (0.10)	1.3	DCE	83	28
9	AuCl (0.10)	1.3	Dioxane	101	30
10	AuCl (0.10)	1.3	Toluene	110	32
11	AuCl (0.10)	1.3	*p*-Xylene	138	30
12	AuCl/AgSbF_6_ (0.10/0.15)	1.3	CH_3_CN	82	15
13	AuCl/AgSbF_6_ (0.10/0.15)	1.3	Dioxane	101	27
**14**	**AuCl/AgSbF**_**6**_ (0.10/0.15)	**1.3**	**Toluene**	**110**	**35**
15^b^	AuCl/AgSbF_6_ (0.10/0.15)	1.3	Toluene	rt	
16^c^	AuCl/AgSbF_6_ (0.10/0.15)	1.3	Toluene	50	11
17	AuCl/AgSbF_6_ (0.05/0.10)	1.3	Toluene	110	32
18	AuCl/AgSbF_6_ (0.10/0.15)	2.0	Toluene	110	29
19	AuCl/AgSbF_6_ (0.10/0.15)	3.0	Toluene	110	25
20	AuI/AgSbF_6_ (0.10/0.15)	1.3	Toluene	110	31
21	C_18_H_33_AuClP/AgSbF_6_ (0.10/0.15)	1.3	Toluene	110	32
22	HAuCl_4_·3H_2_O/AgSbF_6_ (0.10/0.15)	1.3	Toluene	110	28
23	[(IMes)AuCl]/AgSbF_6_ (0.10/0.15)	1.3	Toluene	110	29
24	[(IPr)AuCl]/AgSbF_6_ (0.10/0.15)	1.3	Toluene	110	31
25	Et_3_PAuCl/AgSbF_6_ (0.10/0.15)	1.3	Toluene	110	25
26	Ph_3_PAuCH_3_/AgSbF_6_ (0.10/0.15)	1.3	Toluene	110	24
27	JohnPhos AuCl (0.10)	1.3	Toluene	110	20
28	JohnPhos AuCl/AgSbF_6_ (0.10/0.15)	1.3	Toluene	110	30

a Isolated yields.

b The starting β-enaminone **3a** was recovered in 26% yield.

c The starting β-enaminone **3a** was recovered in 25% yield.

We also investigated the reaction of *N*-methyl-*N*-propargyl β-enaminone **3a** with DMAD (**4a**) in the presence of copper catalysts (Table 3). 1.3 equiv of DMAD was used in these reactions as well. The reaction with CuI (0.10 equiv) in refluxing acetonitrile afforded a new product, 3-alkenyl-1,2-dihydropyridine **6a**, in 16% yield, along with 1,2,3,4-tetrasubstituted benzene **5a** as a byproduct in 5% yield (Table 3, entry 1). When the same reaction was carried out with 1.0 equiv of CuI, the product yields increased slightly (23% for **6a** and 12% for **5a**) (Table 3, entry 2). When CuBr (0.10 equiv) was employed, 1,2-dihydropyridine **6a** was obtained in 29% yield, while benzene **5a** was formed in 23% yield (Table 3, entry 3). When CuCl (0.10 equiv) was used, only benzene **5a** was obtained with a 16% yield (Table 3, entry 4). The reaction was also tested with Cu­(OAc) and Cu­(OAc)_2_, but these reactions produced 1,2-dihydropyridine **6a** in 17 and 24% yields, respectively (Table 3, entries 5 and 6). In the reaction with [(iPr)­CuCl], 1,2-dihydropyridine **6a** and benzene **5a** resulted in 15 and 14% yields, respectively (Table 3, entry 7). So far, the highest yield (29%) of 3-alkenyl-1,2-dihydropyridine **6a** was obtained with CuBr; therefore, subsequent reactions were carried out with this catalyst. The reaction with CuBr was also tested with 2.0 and 3.0 equiv of DMAD. These reactions generated 1,2-dihydropyridine **6a** in 39% yields, along with benzene **5a** in 8–9% yields (Table 3, Entries 8 and 9). Since an excess amount (3.0 equiv) of DMAD did not increase the product yield (39%), subsequent reactions were carried out with 2.0 equiv of DMAD. We performed the same reaction at lower temperatures as well. The reaction at room temperature yielded 1,2-dihydropyridine **6a** in 19% yield (Table 2, entry 10). The reaction at 50 °C afforded 1,2-dihydropyridine **6a** and benzene **5a** in 10 and 2% yields, respectively, along with the recovered starting β-enaminone **3a** (11%) (Table 2, entry 11). The reaction in DMF and DMSO at 80 °C produced only benzene **5a** but in very low yields (2 and 4%, respectively) (Table 3, entries 12 and 13). The reaction was also tested in THF, DCE, dioxane, and toluene (Table 3, entries 14–17). In these reactions, benzene **5a** was obtained as the major product in 11–23% yields, while 1,2-dihydropyridine **6a** was formed as a byproduct in 7–9% yields. Finally, the reaction was also carried out with lower (0.50 equiv) and higher (1.00 equiv) amounts of CuBr, but a dramatic decrease in product yields was observed (Table 2, entries 18 and 19). In summary, the highest yield of 3-alkenyl-1,2-dihydropyridine **6a** (39%) was obtained in the presence of 0.10 equiv of CuBr catalyst using either 2.0 or 3.0 equiv of DMAD (Table 3, entries 8 and 9); however, derivatization studies of 1,2-dihydropyridines **6** have been performed under the condition using 2.0 equiv of DMAD (Table 3, entry 8).

**Table 3:**
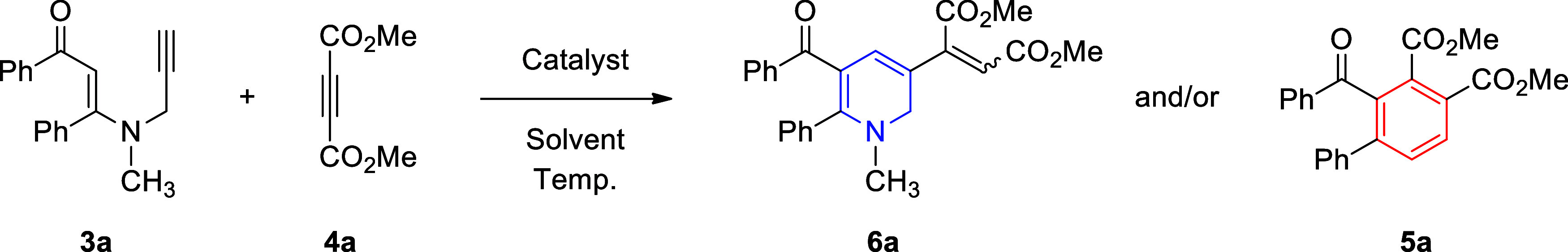
Optimization of the Reaction Conditions under Copper Catalysis^a^

entry	catalyst (equiv)	DMAD (**4a**) (equiv)	solvent	temp. (°C)	yields of products (%)
1	CuI (0.10)	1.3	CH_3_CN	82	**6a** (16) + **5a** (5)
2	CuI (1.00)	1.3	CH_3_CN	82	**6a** (23) + **5a** (12)
3	CuBr (0.10)	1.3	CH_3_CN	82	**6a** (29) + **5a** (23)
4	CuCl (0.10)	1.3	CH_3_CN	82	**5a** (16)
5	Cu(OAc) (0.10)	1.3	CH_3_CN	82	**6a** (17)
6	Cu(OAc)_2_ (0.10)	1.3	CH_3_CN	82	**6a** (24)
7	[(iPr)CuCl] (0.10)	1.3	CH_3_CN	82	**6a** (15) + **5a** (14)
8	**CuBr** (0.10)	**2.0**	**CH**_**3**_ **CN**	82	**6a (39)** + **5a (9)**
9	CuBr (0.10)	3.0	CH_3_CN	82	**6a** (39) + **5a** (8)
10	CuBr (0.10)	2.0	CH_3_CN	rt	**6a** (19)
11^b^	CuBr (0.10)	2.0	CH_3_CN	50	**6a** (10) + **5a** (2)
12	CuBr (0.10)	2.0	DMF	80	**5a** (2)
13	CuBr (0.10)	2.0	DMSO	80	**5a** (4)
14	CuBr (0.10)	2.0	THF	65	**6a** (8) + **5a** (19)
15	CuBr (0.10)	2.0	DCE	83	**6a** (7) + **5a** (20)
16	CuBr (0.10)	2.0	Dioxane	101	**6a** (9) + **5a** (11)
17	CuBr (0.10)	2.0	Toluene	110	**6a** (7) + **5a** (23)
18	CuBr (0.50)	2.0	CH_3_CN	82	**6a** (7) + **5a** (2)
19	CuBr (1.00)	2.0	CH_3_CN	82	**6a** (3) + **5a** (13)

a Isolated yields.

b The starting β-enaminone **3a** was recovered in 11% yield.

After optimizing the reaction conditions, we first explored the substrate scope and limitations of the Au-catalyzed reaction. For this purpose, various *N*-methyl-*N*-propargyl β-enaminones **3** were subjected to the reaction with 1.3 equiv of dialkyl acetylenedicarboxylates (**4**) in the presence of AuCl/AgSbF_6_ (0.10/0.15 equiv) in toluene at 110 °C (Table 4). All reactions produced the expected 1,2,3,4-tetrasubstituted benzene **5** derivatives. By employing these reactions, 23 different benzene **5** derivatives were synthesized from the corresponding *N*-methyl-*N*-propargyl β-enaminones **3** with yields ranging from 16 to 37% (Table 4). Many drugs and drug candidate molecules in clinical trials contain halogen atoms in their structures because halogen bonds increase and improve drug-target binding affinity.^41^ Fluorinated drugs, in particular, hold a special place in the pharmaceutical industry because fluorination has positive effects on absorption, distribution, metabolism, and excretion.^42^ Therefore, in this study, 10 derivatives of benzenes **5** containing fluorine, chlorine, and/or bromine atoms in their structures were synthesized with yields ranging from 25 to 35% (Table 4).

**Table 4:**
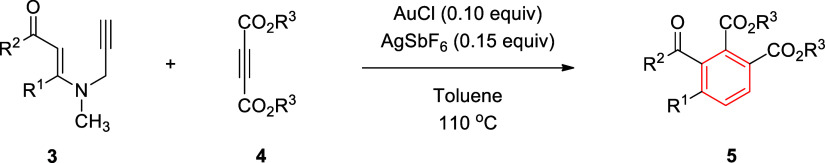

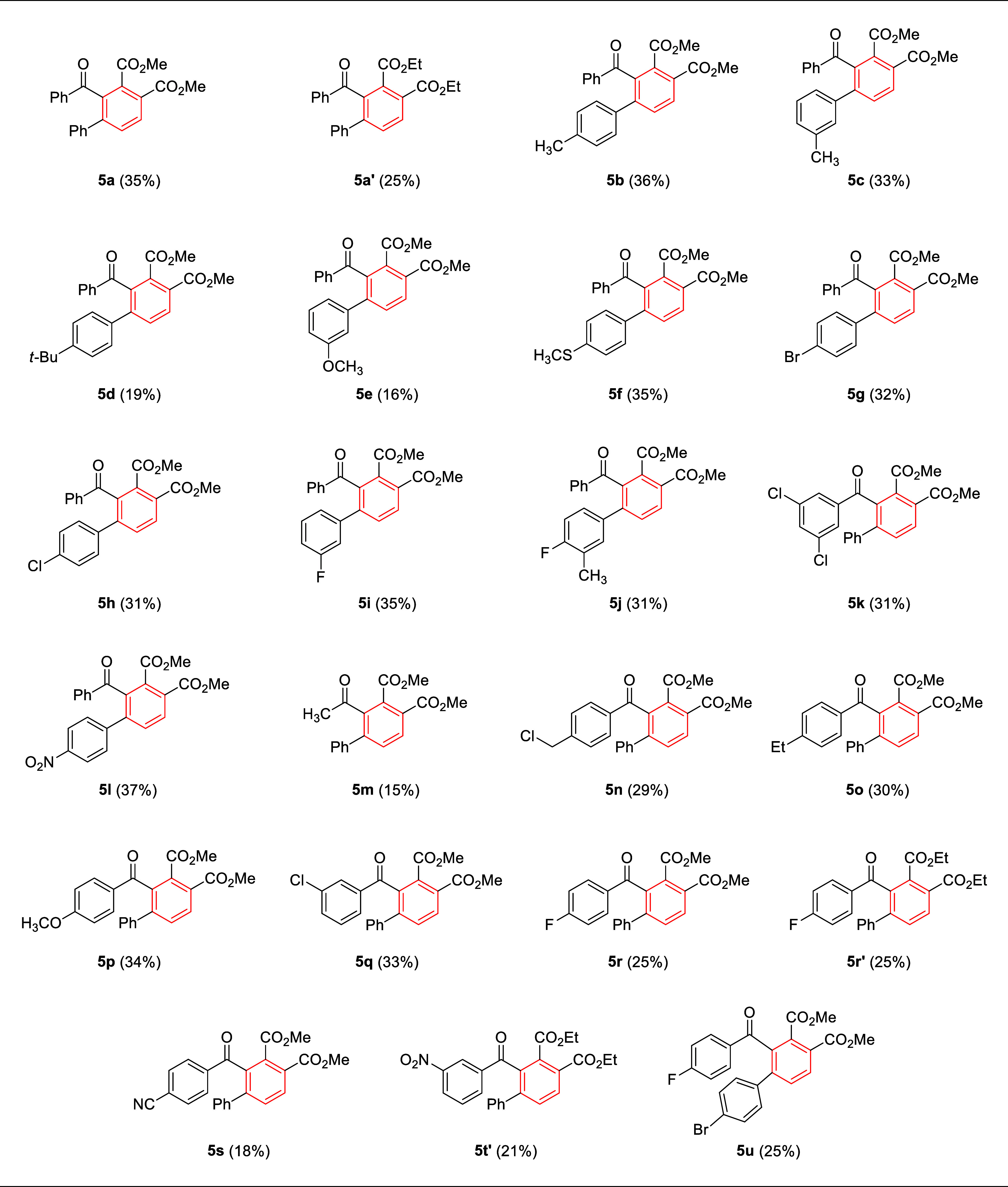
Synthesis of 1,2,3,4-Tetrasubstituted Benzenes **5** under Gold Catalysis^a^

a Isolated yields.

We examined the generality and substrate scope for the Cu-catalyzed reaction as well. Results are given in Table 5. Different *N*-methyl-*N*-propargyl β-enaminones **3** were reacted with 2.0 equiv of dialkyl acetylenedicarboxylates (**4**) in the presence of CuBr (0.10 equiv) in acetonitrile at 82 °C. These reactions furnished 3-alkenyl-1,2-dihydropyridines **6** as the major products. By utilizing these reactions, 12 different 3-alkenyl-1,2-dihydropyridine **6** derivatives were synthesized with yields ranging from 15 to 61%. As previously mentioned, halogen-containing organic compounds have gained importance in the pharmaceutical industry.^41,42^ That is why, 6 halogen-bearing 1,2-dihydropyridine **6** derivatives were prepared in 22–61% yields (Table 5). Notably, 1,2,3,4-tetrasubstituted benzenes **5** were also obtained from these reactions, but as the minor products between 3 and 15% yields, as depicted in Table 5. Unfortunately, their formation under copper catalysis could not be totally prevented and 12 derivatives were formed during these reactions, 6 of them being halogenated derivatives.

**Table 5:**
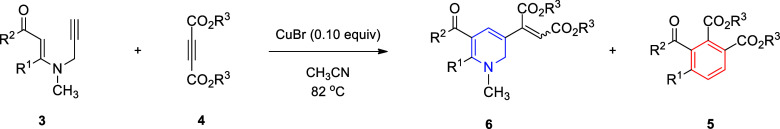

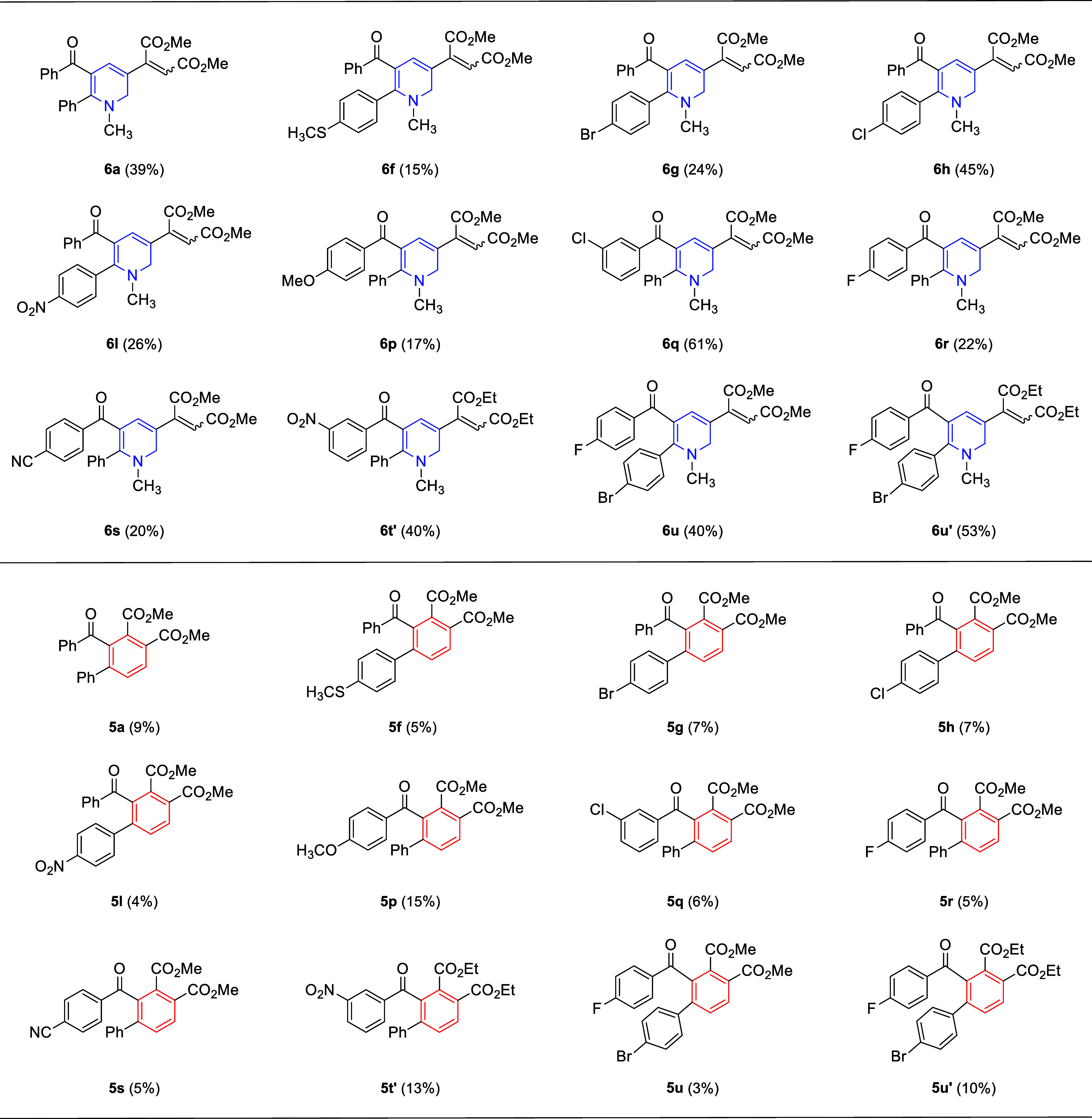
Synthesis of 3-Alkenyl-1,2-dihydropyridines **6** under Copper Catalysis^a^

a Isolated yields.

Lower yields of the products (1,2,3,4-tetrasubstituted benzenes **5** and 3-alkenyl-1,2-dihydropyridines **6**) may be attributed to the presence of a tertiary amine functionality in the structure of the starting *N*-methyl-*N*-propargyl β-enaminones **3**. The lack of N–H prevents the proton transfer steps (often rate-limiting), such as enamine/iminium equilibrium or interconversion. Although tertiary amines are electron-donating, their lone pair is often sterically hindered and exhibits poorer overlap with the π-system, which results in weaker delocalization, and they cannot donate electron density efficiently into the C=C–C=O system.^30^ All these factors presumably reduce the reactivity of *N*-methyl-*N*-propargyl β-enaminones **3**, lowering the yields of the products.

In order to further widen the array of products, we also examined two reactions of *N*-methyl-*N*-propargylic β-enaminone that contains internal alkyne functionality, namely, **3v** (Scheme 4). The reactions were conducted with 2.0 equiv of DMAD (**4a**) under gold and copper catalysis, which produced 1,2,3,4,5-pentasubstituted benzene **5v** in 13 and 12% yields, respectively. Presumably, the steric and/or electronic effects of the phenyl group on the alkyne moiety significantly diminished the product yield, which requires further optimization. Interestingly, in the reaction with CuBr, a 1,2-dihydropyridine type product did not form, or it formed in very low yield and escaped from the isolation. It is noteworthy that pentasubstituted arenes are also important scaffolds in medicinal and pharmaceutical chemistry since they provide rigid and sterically defined pharmacophores, improve receptor selectivity, and enhance metabolic stability in drugs.^1,43^ Moreover, they are building blocks for hexasubstituted arenes, ligands, and materials. They are nontrivial to access via conventional methods, and their syntheses often require tandem and multistep reactions. They are generally synthesized by sequential electrophilic aromatic substitution (EAS), cross-coupling, and C–H activation reactions.^43,44^ So, the reactions of internal alkyne-tethered *N*-methyl-*N*-propargyl β-enaminones with dialkyl acetylenedicarboxylates may provide a simple one-pot entry to pentasubstituted arenes.

**Scheme 4:**
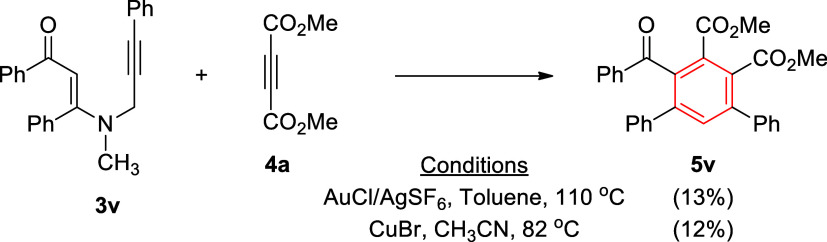
Reaction of Internal Alkyne-Tethered *N*-Methyl-*N*-propargyl β-Enaminone **3v** with DMAD (**4a**) under Gold and Copper Catalysis

We also tested the reaction of *N*-methyl-*N*-propargyl β-enaminone **3a** with ethyl propiolate (**7**) under both gold and copper catalysis (Scheme 5). Unfortunately, these reactions did not produce the expected products such as **5y** and/or **6y**; instead, they gave unidentified decomposition products. Noticeably, as compared with a dialkyl acetylenedicarboxylate (**4**), ethyl propiolate (**7**) is well known to be less electrophilic and reactive. Although it can act as a Michael acceptor, it usually requires stronger nucleophiles or harsher conditions. Moreover, ethyl propiolate has a terminal alkyne proton and can behave as a nucleophile after deprotonation. In the presence of metal salts, it can generate metalated propiolates with nucleophilic property as well.^45^ In brief, lower electrophilicity or polarity inversion (umpolung) may have inhibited the reaction of ethyl propiolate with β-enaminone **3a** under these conditions.

**Scheme 5:**
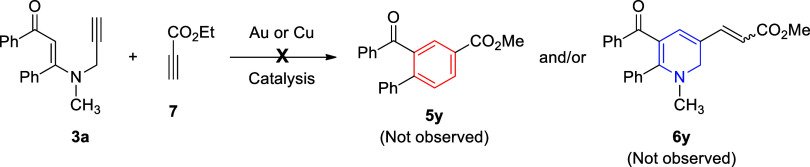
Reaction of *N*-Methyl-*N*-propargyl β-Enaminone **3a** with Ethyl Propiolate (**7**) under Gold and Copper Catalysis

Finally, we investigated the reaction of 1,2-dihydropyridine **8h** with DMAD (**4a**) under copper catalysis (Scheme 6) (see the Experimental Section for the synthesis of 1,2-dihydropyridine **8h**). Unfortunately, this reaction did not produce any products, such as **6h**, other than decomposition products. This clearly shows that no such C–H bond activation reaction occurs during the formation of 3-alkenyl-1,2-dihydropyridines **6**.^46^

**Scheme 6:**
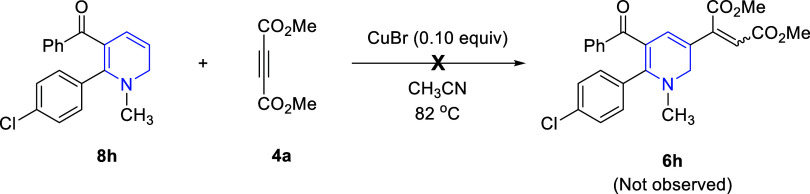
Reaction of 1,2-Dihydropyridine **8h** with DMAD (**4a**) under Copper Catalysis

The proposed mechanism for the formation of 1,2,3,4-tetrasubstituted benzenes **5** is given in Scheme 7. First, coordination of the alkyne group of dialkyl acetylenedicarboxylate (**4**) to the metal catalyst activates the alkyne moiety, forming intermediate **4′**. Then, conjugate addition of β-enaminone **3** to **4′** yields intermediate **9**. Subsequently, metal-catalyzed intramolecular cyclization generates intermediate **10**, which then converts into intermediate **11** by the displacement of the hydrogen atom. Reductive elimination forms intermediate **12**, which immediately undergoes 6π electrocyclization to give bicyclic compound **13**. Finally, retro [2+2] cycloaddition produces 1,2,3,4-tetrasubstituted benzene **5**, along with an imine compound (H_3_C–N=CH_2_) (Scheme 7). Unfortunately, we could not detect the formation of this imine during the reactions. Notably, imine product should be volatile (having a very low boiling point). If not, it is thought to react with water during extraction to form (methylamino)­methanol (H_3_C-NH–CH_2_–OH), passing into the aqueous phase.

**Scheme 7:**
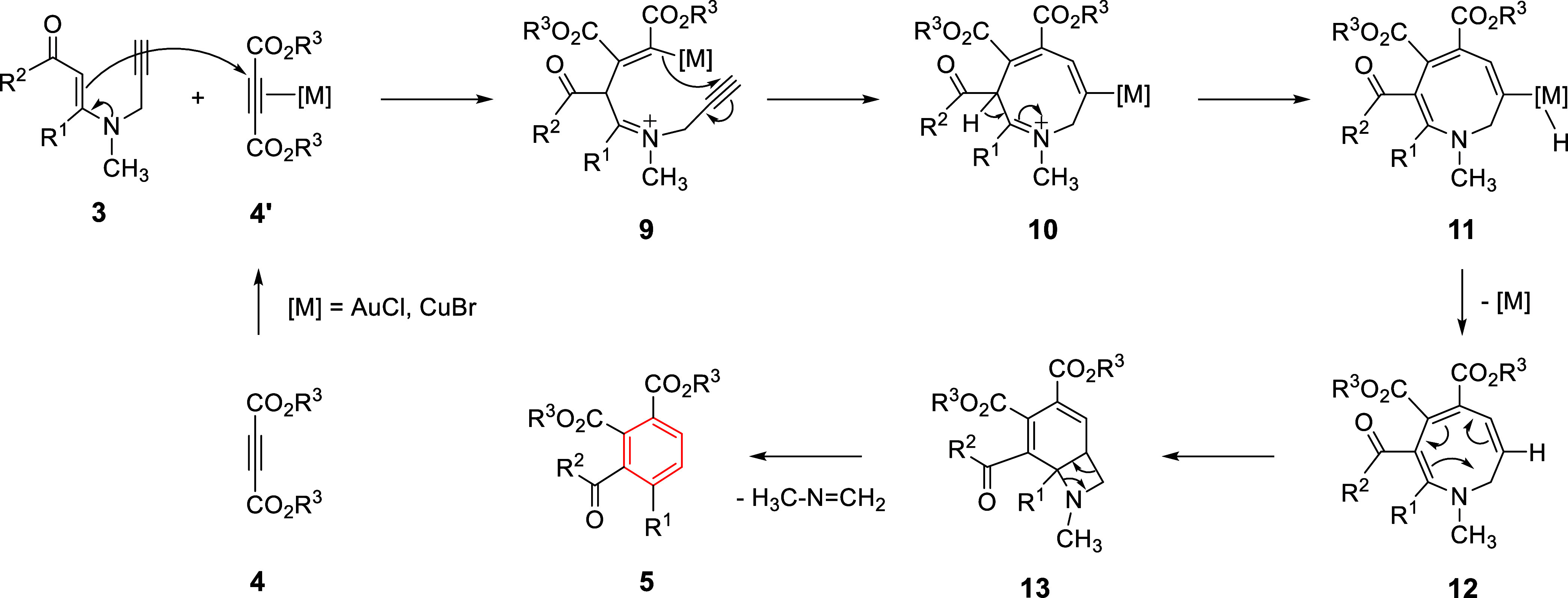
Proposed Mechanism for the Formation of 1,2,3,4-Tetrasubstituted Benzenes **5**

The mechanism proposed for the formation of 3-alkenyl-1,2-dihydropyridine **6** derivatives is shown in Scheme 8. First, coordination of the alkyne moiety of β-enaminone **3** to the metal catalyst activates the alkyne group, giving intermediate **3′**. In fact, activation of the alkyne moiety initiates 6-*endo-dig* cyclization, which produces the cyclic intermediate **14**. Subsequently, metal-catalyzed conjugate addition of **14** to dialkyl acetylenedicarboxylate (**4**) yields intermediate **15**, which then converts into intermediate **16** by the displacement of the hydrogen atom. Finally, reductive elimination affords 3-alkenyl-1,2-dihydropyridine **6** (Scheme 8). When the mechanism is examined, it is seen that two processes occur consecutively. First, dihydropyridine intermediate **14** forms from the starting material by cyclization, and then its conjugate addition to dialkyl acetylenedicarboxylate (**4**) affords the final product, 3-alkenyl-1,2-dihydropyridine **6** (Scheme 8). As concluded from the control experiment in Scheme 6, no neutral 1,2-dihydropyridine product, such as **8**, forms during the formation of 3-alkenyl-1,2-dihydropyridine product **6**, and no C–H bond activation reaction takes place. In brief, the second process, i.e., introduction of an alkenyl group into the ring, goes through the conjugated addition of the copper-dihydropyridine intermediate **14** to dialkyl acetylenedicarboxylate **4**, instead of a C–H bond activation reaction.^46^

**Scheme 8:**
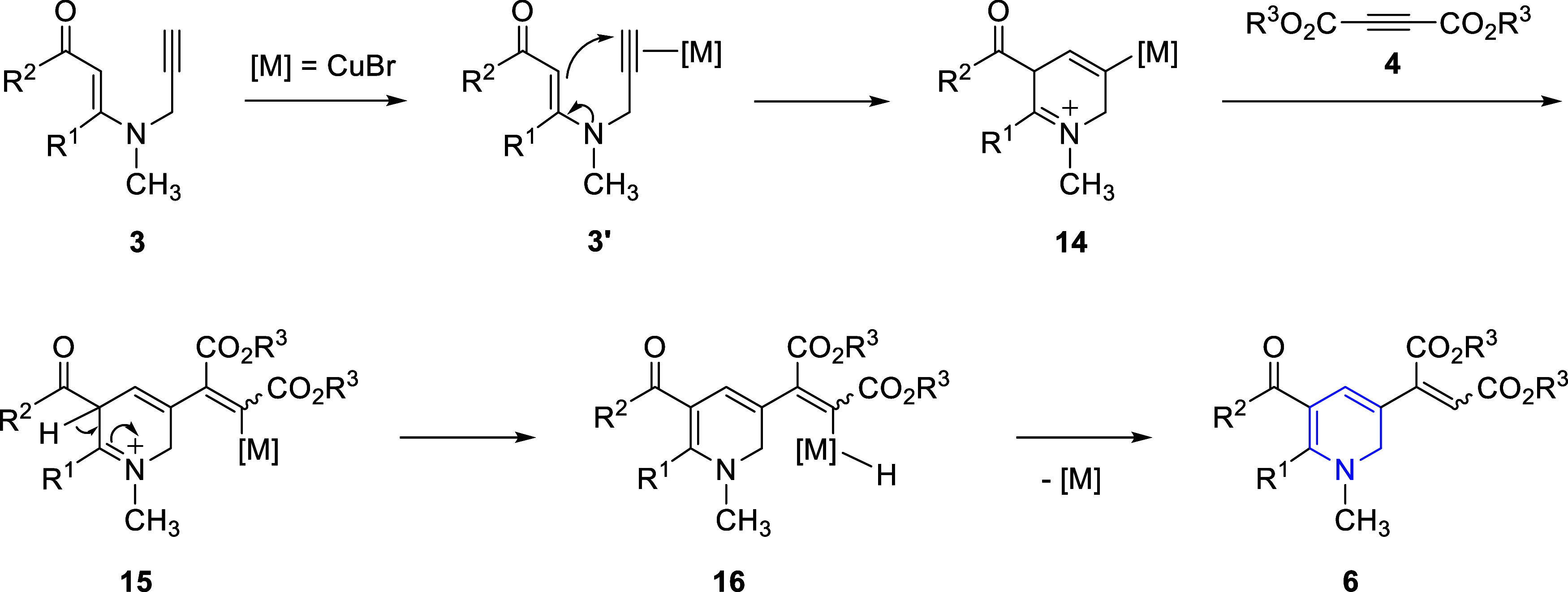
Proposed Mechanism for the Formation of 3-Alkenyl-1,2-dihydropyridines **6**

## Conclusions

In summary, we have uncovered new and novel reactions of *N*-methyl-*N*-propargyl β-enaminones with dialkyl acetylenedicarboxylates. When treated with dialkyl acetylenedicarboxylates in toluene at reflux under AuCl/AgSbF_6_ catalysis, *N*-methyl-*N*-propargyl β-enaminones produced 1,2,3,4-tetrasubstituted benzenes. During the course of the reaction, the conjugate addition of β-enaminone to dialkyl acetylenedicarboxylate, followed by cyclization of the resulting molecule, forms an eight-membered 1,2-dihydroazocine intermediate, which undergoes a series of cycloaddition reactions to yield a 1,2,3,4-tetrasubstituted benzene derivative with the ejection of an imine moiety (*N*-methylenemethanamine) from the molecule. The reaction was general for a variety of *N*-methyl-*N*-propargyl β-enaminones and tolerated the presence of a range of substituents. However, the same reaction in acetonitrile at reflux under CuBr catalysis furnished 3-alkenyl-1,2-dihydropyridines as the major product, along with 1,2,3,4-tetrasubstituted benzenes as the minor product. Formation of 3-alkenyl-1,2-dihydropyridine involves intramolecular cyclization followed by the conjugate addition of the resulting six-membered dihydropyridine intermediate to dialkyl acetylenedicarboxylate. When an internal alkyne-tethered *N*-methyl-*N*-propargyl β-enaminone was employed as the starting material, a pentasubstituted benzene derivative was obtained from these reactions under both gold and copper catalysis. The diversity of the synthesized 1,2,3,4-tetrasubstituted benzenes and 3-alkenyl-1,2-dihydropyridines may provide many new carbocyclic and nitrogen-based heterocyclic systems, respectively, for drug discovery and development.

## Experimental Section

### General Information

^1^H and ^13^C NMR spectra were recorded at 400 and 100 MHz, respectively. Chemical shifts were given in parts per million (ppm) relative to CDCl_3_ (7.26 and 77.16 ppm) in ^1^H and ^13^C NMR, respectively. Coupling constants (*J*) were given in hertz (Hz), and spin multiplicities were shown by the following symbols: s (singlet), d (doublet), t (triplet), q (quartet), p (pentet), and m (multiplet). Infrared (IR) spectra were obtained using attenuated total reflection (ATR). Band positions were recorded in reciprocal centimeters (cm^–1^). Mass spectra (MS) and high-resolution MS (HRMS) were obtained using electrospray ionization (ESI) with micro-Tof; *m*/*z* values are reported (for each measurement, the mass scale was recalibrated with sodium formate clusters, and samples were dissolved and measured in MeOH or CH_3_CN). Flash chromatography was performed using thick-walled glass columns and “flash-grade” silica gel (230–400 mesh). Thin-layer chromatography (TLC) was accomplished by using commercially prepared 0.25 mm silica gel plates. TLC plates were visualized by exposing them to UV light or iodine vapors. The relative proportions of solvents in chromatography solvent mixtures refer to the volume/volume ratio. All commercially available reagents were used directly without purification unless otherwise stated. All solvents used in reactions and chromatography were distilled and/or dried properly for purity. The inert atmosphere was created using a slight positive pressure (ca. 0.1 psi) of argon. All glassware was dried in an oven prior to use.

### General Procedure for the Synthesis of *N*-Methyl-*N*-propargyl β-Enaminones **3** (Table 1)

To a stirred solution of the corresponding α,β-alkynic ketone **1** (1.00 mmol) in methanol (10 mL) under argon was added *N*-methylpropargylamine (**2**) (1.20 mmol) and the resulting mixture was refluxed for approximately 6 h in an oil bath (The progress of the reaction was monitored by routine TLC analysis for the disappearance of α,β-alkynic ketone **1** using hexane/ethyl acetate (4:1) as the eluent). After the reaction was over, the solvent was removed on a rotary evaporator, and ethyl acetate (30 mL) and a saturated aqueous solution of NaCl (15 mL) were added. After the layers were separated, the aqueous layer was extracted with ethyl acetate (2 × 30 mL) again. The combined organic layers were dried over MgSO_4_ and evaporated on a rotary evaporator to give the crude product, which was purified by flash chromatography on silica gel using hexane/ethyl acetate (9:1 followed by 4:1) as the eluent to afford the corresponding *N*-methyl-*N*-propargyl β-enaminone **3**.

#### 3-(Methyl­(prop-2-yn-1-yl)­amino)-1,3-diphenylprop-2-en-1-one (**3a**)

1,3-Diphenylprop-2-yn-1-one (**1a**) (781.0 mg, 3.80 mmol) and *N*-methylpropargylamine (**2**) (320.0 mg, 4.56 mmol) were employed to afford 480.6 mg (46%) of the indicated product **3a** as a yellow oil (*R*_f_ = 0.30 in 4:1 hexane/ethyl acetate).

**3a:** ^1^H NMR (400 MHz, CDCl_3_) δ 7.90 (d, *J* = 7.0 Hz, 2H), 7.45 (m, 4H), 7.40 (d, *J* = 7.5 Hz, 2H), 7.38 (t, *J* = 1.5 Hz, 1H), 7.33 (t, *J* = 1.2 Hz, 1H), 6.09 (s, 1H), 3.88 (s, 2H), 3.05 (s, 3H), 2.37 (s, 1H); ^13^C NMR (100 MHz, CDCl_3_) δ 188.3 (C), 163.6 (C), 141.3 (C), 136.2 (C), 131.1 (CH), 129.0 (CH), 128.7 (CH), 128.4 (CH), 128.1 (CH), 127.9 (CH), 96.9 (CH), 78.1 (C), 73.2 (CH), 42.1 (CH_3_), 38.0 (CH_2_); IR­(neat): 3291, 3059, 2951, 1723, 1667, 1595, 1524, 1446, 1434, 1411, 1295, 1269, 1226, 1200, 1170, 1132, 1061, 1000, 937, 827, 751, 695, 673, 608, 569, 555, 520 cm^–1^; MS (ESI, *m*/*z*): 276.14 [M + H]^+^; HRMS (ESI, *m*/*z*): calcd. for C_19_H_18_NO: 276.1383 [M + H]^+^, found: 276.1390.

#### 3-(Methyl­(prop-2-yn-1-yl)­amino)-1-phenyl-3-(*p*-tolyl)­prop-2-en-1-one (**3b**)

1-Phenyl-3-(*p*-tolyl)­prop-2-yn-1-one (**1b**) (812.0 mg, 3.69 mmol) and *N*-methylpropargylamine (**2**) (310.0 mg, 4.43 mmol) were employed to afford 394.6 mg (37%) of the indicated product **3b** as an orange solid (*R*_f_ = 0.25 in 4:1 hexane/ethyl acetate; mp 80.6–81.8 °C).

**3b:** ^1^H NMR (400 MHz, CDCl_3_) δ 7.91 (d, *J* = 7.3 Hz, 2H), 7.46–7.37 (m, 3H), 7.26 (d, *J* = 7.9 Hz, 2H), 7.21 (d, *J* = 8.0 Hz, 2H), 6.07 (s, 1H), 3.89 (s, 2H), 3.05 (s, 3H), 2.42 (s, 3H), 2.37 (s, 1H); ^13^C NMR (100 MHz, CDCl_3_) δ 188.3 (C), 163.9 (C), 141.3 (C), 138.9 (C), 133.1 (C), 131.0 (CH), 129.5 (CH), 128.3 (CH), 128.1 (CH), 127.9 (CH), 96.9 (CH), 78.2 (C), 73.1 (CH), 42.1 (CH_3_), 37.9 (CH_2_), 21.6 (CH_3_); IR (neat): 3235, 2862, 2805, 1625, 1611, 1574, 1509, 1434, 1417, 1339, 1246, 1209, 1051, 1019, 940, 910, 826, 768, 698, 669, 584, 559, 506, 468, 424 cm^–1^; MS (ESI, *m*/*z*): 290.15 [M + H]^+^; HRMS (ESI, *m*/*z*): calcd. for C_20_H_20_NO: 290.1539 [M + H]^+^, found: 290.1533.

#### 3-(Methyl­(prop-2-yn-1-yl)­amino)-1-phenyl-3-(*m*-tolyl)­prop-2-en-1-one (**3c**)

1-Phenyl-3-(*m*-tolyl)­prop-2-yn-1-one (**1c**) (850.0 mg, 3.86 mmol) and *N*-methylpropargylamine (**2**) (320.0 mg, 4.63 mmol) were employed to afford 439.2 mg (39%) of the indicated product **3c** as a red oil (*R*_f_ = 0.26 in 4:1 hexane/ethyl acetate).

**3c:** ^1^H NMR (400 MHz, CDCl_3_) δ 7.89 (d, *J* = 7.1 Hz, 2H), 7.43 (d, *J* = 7.1 Hz, 1H), 7.40 (d, *J* = 7.6 Hz, 2H), 7.33 (t, *J* = 3.8 Hz, 1H), 7.24 (d, *J* = 7.6 Hz, 1H), 7.12 (bs, 2H), 6.06 (s, 1H), 3.88 (s, 2H), 3.04 (s, 3H), 2.47 (s, 1H), 2.38 (s, 3H); ^13^C NMR (100 MHz, CDCl_3_) δ 188.4 (C), 163.8 (C), 141.3 (C), 138.2 (C), 136.1 (C), 131.0 (CH), 129.8 (CH), 128.9 (CH), 128.6 (CH), 128.1 (CH), 127.8 (CH), 125.5 (CH), 96.9 (CH), 78.2 (C), 73.1 (CH), 42.1 (CH_3_), 38.0 (CH_2_), 21.6 (CH_3_); IR (neat): 3285, 3220, 3055, 2920, 1733, 1671, 1596, 1516, 1399, 1341, 1219, 1197, 1049, 978, 914, 770, 700, 665, 590, 472, 439 cm^–1^; MS (ESI, *m*/*z*): 288.14 [M-H]^−^; HRMS (ESI, *m*/*z*): calcd. for C_20_H_18_NO: 288.1388 [M-H]^−^, found: 288.1382.

#### 3-(4-(*tert*-Butyl)­phenyl)-3-(methyl­(prop-2-yn-1-yl)­amino)-1-phenylprop-2-en-1-one (**3d**)

3-(4-(*tert*-Butyl)­phenyl)-1-phenylprop-2-yn-1-one (**1d**) (753.0 mg, 2.87 mmol) and *N*-methylpropargylamine (**2**) (240.0 mg, 3.43 mmol) were employed to afford 189.8 mg (20%) of the indicated product **3d** as an orange oil (*R*_f_ = 0.32 in 4:1 hexane/ethyl acetate).

**3d:** ^1^H NMR (400 MHz, CDCl_3_) δ 7.86 (d, *J* = 7.4 Hz, 2H), 7.45–7.41 (m, 3H), 7.37 (d, *J* = 7.6 Hz, 2H), 7.24 (d, *J* = 8.1 Hz, 2H), 6.04 (s, 1H), 3.86 (s, 2H), 3.03 (s, 3H), 2.37 (s, 1H), 1.36 (s, 9H); ^13^C NMR (100 MHz, CDCl_3_) δ 188.7 (C), 163.8 (C), 151.7 (C), 141.4 (C), 132.8 (C), 130.8 (CH), 128.1 (CH), 127.9 (CH), 127.7 (CH), 125.5 (CH), 97.2 (CH), 78.2 (C), 73.1 (CH), 42.1 (CH_3_), 37.9 (C), 34.7 (CH_2_), 31.3 (CH_3_); IR (neat): 3285, 3230, 2960, 2903, 2867, 1735, 1597, 1508, 1403, 1362, 1341, 1266, 1204, 1109, 1046, 1017, 976, 929, 832, 774, 703, 648, 588, 559, 478 cm^–1^; MS (ESI, *m*/*z*): 332.20 [M + H]^+^; HRMS (ESI, *m*/*z*): calcd. for C_23_H_26_NO: 332.2009 [M + H]^+^, found: 332.2019.

#### 3-(3-Methoxyphenyl)-3-(methyl­(prop-2-yn-1-yl)­amino)-1-phenylprop-2-en-1-one (**3e**)

3-(3-Methoxyphenyl)-1-phenylprop-2-yn-1-one (**1e**) (827.0 mg, 3.54 mmol) and *N*-methylpropargylamine (**2**) (290.0 mg, 4.25 mmol) were employed to afford 631.0 mg (58%) of the indicated product **3e** as a red oil (*R*_f_ = 0.10 in 4:1 hexane/ethyl acetate).

**3e:** ^1^H NMR (400 MHz, CDCl_3_) δ 7.88 (d, *J* = 6.9 Hz, 2H), 7.43–7.36 (m, 4H), 7.15 (dd, *J* = 7.4, 1.7 Hz, 1H), 7.03 (d, *J* = 7.4 Hz, 1H), 6.98 (d, *J* = 8.3 Hz, 1H), 6.08 (s, 1H), 3.92 (s, 2H), 3.83 (s, 3H), 3.09 (s, 3H), 2.34 (s, 1H); ^13^C NMR (100 MHz, CDCl_3_) δ 187.7 (C), 160.1 (C), 156.5­(C), 141.6 (C), 130.7 (CH), 130.3 (CH), 129.6 (CH), 128.0 (CH), 127.8 (CH), 125.2 (C), 121.1 (CH), 111.1 (CH), 95.4 (CH), 78.3 (C), 72.8 (CH), 55.8 (CH_3_), 41.5 (CH_3_), 37.4 (CH_2_); IR (neat): 3280, 3227, 3058, 2934, 2836, 1732, 1632, 1598, 1577, 1491, 1461, 1407, 1341, 1280, 1243, 1114, 1040, 1022, 976, 934, 830, 798, 700, 664, 582, 536 cm^–1^; MS (ESI, *m*/*z*): 304.13 [M-H]^−^; HRMS (ESI, *m*/*z*): calcd. for C_20_H_18_NO_2_: 304.1338 [M-H]^−^, found: 304.1342.

#### 3-(Methyl­(prop-2-yn-1-yl)­amino)-3-(4-(methylthio)­phenyl)-1-phenylprop-2-en-1-one (**3f**)

3-(4-(Methylthio)­phenyl)-1-phenylprop-2-yn-1-one (**1f**) (756.0 mg, 3.00 mmol) and *N*-methylpropargylamine (**2**) (250.0 mg, 3.60 mmol) were employed to afford 372.2 mg (39%) of the indicated product **3f** as an orange oil (*R*_f_ = 0.10 in 4:1 hexane/ethyl acetate).

**3f:** ^1^H NMR (400 MHz, CDCl_3_) δ 7.83 (d, *J* = 8.5 Hz, 2H), 7.46–7.42 (m, 3H), 7.32–7.29 (m, 2H), 7.22 (d, *J* = 8.5 Hz, 2H), 6.05 (s, 1H), 3.87 (s, 2H), 3.04 (s, 3H), 2.55 (s, 1H), 2.50 (s, 3H); ^13^C NMR (100 MHz, CDCl_3_) δ 187.2 (C), 163.4 (C), 142.7 (C), 137.7 (C), 136.2 (C), 129.0 (CH), 128.7 (CH), 128.3 (CH), 127.2 (CH), 125.0 (CH), 96.6 (CH), 78.1 (C), 73.2 (CH), 42.1 (CH_3_), 38.0 (CH_3_), 15.1 (CH_2_); IR (neat): 3270, 3236, 2919, 2890, 1733, 1623, 1587, 1514, 1488, 1398, 1340, 1204, 1182, 1093, 1047, 902, 829, 768, 698, 670, 629, 580, 475 cm^–1^; MS (ESI, *m*/*z*): 322.13 [M + H]^+^; HRMS (ESI, *m*/*z*): calcd. for C_20_H_20_NOS: 322.1260 [M + H]^+^, found: 322.1261.

#### 3-(4-Bromophenyl)-3-(methyl­(prop-2-yn-1-yl)­amino)-1-phenylprop-2-en-1-one (**3g**)

3-(4-Bromophenyl)-1-phenylprop-2-yn-1-one (**1g**) (654.0 mg, 2.29 mmol) and *N*-methylpropargylamine (**2**) (190.0 mg, 2.75 mmol) were employed to afford 517.5 mg (64%) of the indicated product **3g** as an orange solid (*R*_f_ = 0.25 in 4:1 hexane/ethyl acetate; mp 78.7–81.3 °C).

**3g:** ^1^H NMR (400 MHz, CDCl_3_) δ 7.89 (d, *J* = 7.0 Hz, 2H), 7.58 (d, *J* = 8.5 Hz, 2H), 7.47 (t, *J* = 2.5 Hz, 1H), 7.40 (t, *J* = 7.3 Hz, 2H), 7.21 (d, *J* = 8.4 Hz, 2H), 6.09 (s, 1H), 3.86 (s, 2H), 3.04 (s, 3H), 2.38 (s, 1H); ^13^C NMR (100 MHz, CDCl_3_) δ 188.2 (C), 162.3 (C), 141.0 (C), 135.2 (C), 132.0 (CH), 131.3 (CH), 130.1 (CH), 128.2 (CH), 127.8 (CH), 123.3 (C), 97.2 (CH), 77.9 (C), 73.5 (CH), 42.2 (CH_3_), 38.1 (CH_2_); IR (neat): 3243, 2891, 2844, 1683, 1625, 1575, 1516, 1487, 1435, 1416, 1340, 1210, 1177, 1066, 1047, 1010, 910, 828, 766, 700, 670, 580, 506, 465, 406 cm^–1^; MS (ESI, *m*/*z*): 354.05 [M + H]^+^; HRMS (ESI, *m*/*z*): calcd. for C_19_H_17_BrNO: 354.0488 [M + H]^+^, found: 354.0493.

#### 3-(4-Chlorophenyl)-3-(methyl­(prop-2-yn-1-yl)­amino)-1-phenylprop-2-en-1-one (**3h**)

3-(4-Chlorophenyl)-1-phenylprop-2-yn-1-one (**1h**) (689.0 mg, 2.86 mmol) and *N*-methylpropargylamine (**2**) (240.0 mg, 3.43 mmol) were employed to afford 542.8 mg (61%) of the indicated product **3h** as a dark orange oil (*R*_f_ = 0.37 in 4:1 hexane/ethyl acetate).

**3h:** ^1^H NMR (400 MHz, CDCl_3_) δ 7.92 (d, *J* = 7.0 Hz, 2H), 7.46–7.39 (m, 5H), 7.27 (d, *J* = 8.4 Hz, 2H), 6.10 (s, 1H), 3.82 (s, 2H), 3.00 (s, 3H), 2.41 (s, 1H); ^13^C NMR (100 MHz, CDCl_3_) δ 187.7 (C), 161.9 (C), 140.7 (C), 134.5 (C), 134.4 (C), 131.0 (CH), 129.6 (CH), 128.7 (CH), 127.9 (CH), 127.4 (CH), 96.6 (CH), 77.6 (C), 73.4 (CH), 41.8 (CH_3_), 37.7 (CH_2_); IR (neat): 3294, 3061, 2943, 1671, 1591, 1541, 1521, 1397, 1340, 1268, 1224, 1175, 1089, 1012, 907, 832, 764, 699, 687, 513, 476 cm^–1^; MS (ESI, *m*/*z*): 310.10 [M + H]^+^; HRMS (ESI, *m*/*z*): calcd. for C_19_H_17_ClNO: 310.0993 [M + H]^+^, found: 310.0991.

#### 3-(3-Fluorophenyl)-3-(methyl­(prop-2-yn-1-yl)­amino)-1-phenylprop-2-en-1-one (**3i**)

3-(3-Fluorophenyl)-1-phenylprop-2-yn-1-one (**1i**) (707.0 mg, 3.15 mmol) and *N*-methylpropargylamine (**2**) (260.0 mg, 3.78 mmol) were employed to afford 561.0 mg (61%) of the indicated product **3i** as an orange oil (*R*_f_ = 0.21 in 4:1 hexane/ethyl acetate).

**3i:** ^1^H NMR (400 MHz, CDCl_3_) δ 7.88 (d, *J* = 7.0 Hz, 2H), 7.47 (t, *J* = 2.5 Hz, 1H), 7.45 (t, *J* = 1.4 Hz, 1H), 7.40 (t, *J* = 5.8 Hz, 2H), 7.15 (ddd, *J* = 8.5, 2.6, 0.9 Hz, 1H), 7.12–7.09 (m, 1H), 7.04 (ddd, *J* = 9.2, 2.5, 1.5 Hz, 1H), 6.09 (s, 1H), 3.87 (s, 2H), 3.04 (s, 3H), 2.38 (t, *J* = 2.2 Hz, 1H); ^13^C NMR (100 MHz, CDCl_3_) δ 188.3 (C), 162.9 (d, ^1^ *J* = 247.0 Hz, C), 161.8 (C), 141.0 (C), 138.4 (d, ^4^ *J* = 2.8 Hz, CH), 131.3 (CH), 130.4 (d, ^3^ *J* = 8.1 Hz, C), 128.2 (CH), 127.8 (CH), 124.2 (d, ^3^ *J* = 8.2 Hz, CH), 116.0 (d, ^2^ *J* = 22.3 Hz, CH), 115.6 (d, ^2^ *J* = 21.2 Hz, CH), 97.2 (CH), 77.8 (C), 73.5 (CH), 42.2 (CH_3_), 38.1 (CH_2_); IR (neat): 3295, 3232, 3062, 2989, 1732, 1613, 1576, 1519, 1488, 1445, 1404, 1342, 1222, 1196, 1175, 1132, 1046, 982, 916, 891, 768, 697, 521 cm^–1^; MS (ESI, *m*/*z*): 294.13 [M + H]^+^; HRMS (ESI, *m*/*z*): calcd. for C_19_H_17_FNO: 294.1289 [M + H]^+^, found: 294.1281.

#### 3-(4-Fluoro-3-methylphenyl)-3-(methyl­(prop-2-yn-1-yl)­amino)-1-phenylprop-2-en-1-one (**3j**)

3-(4-Fluoro-3-methylphenyl)-1-phenylprop-2-yn-1-one (**1j**) (740.0 mg, 3.11 mmol) and *N*-methylpropargylamine (**2**) (260.0 mg, 3.73 mmol) were employed to afford 617.5 mg (65%) of the indicated product **3j** as an orange solid (*R*_f_ = 0.21 in 4:1 hexane/ethyl acetate; mp 86.1–87.4 °C).

**3j:** ^1^H NMR (400 MHz, CDCl_3_) δ 7.89 (d, *J* = 6.9 Hz, 2H), 7.45 (d, *J* = 7.1 Hz, 1H), 7.41 (s, 1H), 7.39 (t, *J* = 3.4 Hz, 1H), 7.13 (t, *J* = 6.6 Hz, 2H), 7.08 (d, *J* = 9.2 Hz, 1H), 6.06 (s, 1H), 3.87 (s, 2H), 3.03 (s, 3H), 2.38 (s, 1H), 2.30 (s, 3H); ^13^C NMR (100 MHz, CDCl_3_) δ 188.4 (C), 162.8 (C), 161.7 (d, ^1^ *J* = 246.8 Hz, C), 141.2 (C), 131.6 (d, ^4^ *J* = 3.8 Hz, C), 131.5 (d, ^3^ *J* = 5.4 Hz, CH), 131.1 (CH), 128.1 (CH), 127.8 (CH), 127.7 (d, ^3^ *J* = 8.4 Hz, CH), 125.3 (d, ^2^ *J* = 17.7 Hz,C), 115.4 (d, ^2^ *J* = 22.8 Hz, CH), 97.2 (CH), 78.0 (C), 73.3 (CH), 42.1 (CH_3_), 38.0 (CH_2_), 14.8 (d, ^3^ *J* = 3.0 Hz, CH_3_); IR (neat): 3208, 3054, 2963, 2925, 1615, 1595, 1526, 1450, 1486, 1413, 1206, 1178, 1113, 1047, 979, 929, 909, 889, 814, 780, 758, 705, 685, 664, 548, 536, 439 cm^–1^; MS (ESI, *m*/*z*): 308.14 [M + H]^+^; HRMS (ESI, *m*/*z*): calcd. for C_20_H_19_FNO: 308.1445 [M + H]^+^, found: 308.1439.

#### 3-(3,5-Dichlorophenyl)-3-(methyl­(prop-2-yn-1-yl)­amino)-1-phenylprop-2-en-1-one (**3k**)

3-(3,5-Dichlorophenyl)-1-phenylprop-2-yn-1-one (**1k**) (613.0 mg, 2.23 mmol) and *N*-methylpropargylamine (**2**) (190.0 mg, 2.68 mmol) were employed to afford 385.0 mg (50%) of the indicated product **3k** as a yellow solid (*R*_f_ = 0.34 in 4:1 hexane/ethyl acetate; mp 129.8–131.0 °C).

**3k:** ^1^H NMR (400 MHz, CDCl_3_) δ 7.70 (s, 2H), 7.46 (d, *J* = 2.7 Hz, 3H), 7.39 (s, 1H), 7.29 (bs, 2H), 5.91 (s, 1H), 3.89 (s, 2H), 3.10 (s, 3H), 2.39 (s, 1H); ^13^C NMR (100 MHz, CDCl_3_) δ 185.0 (C), 164.9 (C), 144.1 (C), 135.6 (C), 134.8 (CH), 130.4 (CH), 129.2 (C), 128.8 (CH), 128.1 (CH), 126.3 (CH), 95.5 (CH), 77.7 (C), 73.5 (CH), 42.2 (CH_3_), 38.1 (CH_2_); IR (neat): 3220, 2917, 2893, 1632, 1560, 1520, 1429, 1411, 1398, 1341, 1259, 1241, 1161, 1121, 1087, 1064, 990, 940, 905, 857, 781, 748, 696, 671, 560, 519, 469, 429 cm^–1^; MS (ESI, *m*/*z*): 344.06 [M + H]^+^; HRMS (ESI, *m*/*z*): calcd. for C_19_H_16_Cl_2_NO: 344.0604 [M + H]^+^, found: 344.0602.

#### 3-(Methyl­(prop-2-yn-1-yl)­amino)-3-(4-nitrophenyl)-1-phenylprop-2-en-1-one (**3l**)

3-(4-Nitrophenyl)-1-phenylprop-2-yn-1-one (**1l**) (682.0 mg, 2.71 mmol) and *N*-methylpropargylamine (**2**) (220.0 mg, 3.25 mmol) were employed to afford 601.4 mg (69%) of the indicated product **3l** as a yellow solid (*R*_f_ = 0.18 in 4:1 hexane/ethyl acetate; mp 112.7–114.0 °C).

**3l:** ^1^H NMR (400 MHz, CDCl_3_) δ 8.33 (d, *J* = 8.8 Hz, 2H), 7.88 (d, *J* = 7.1 Hz, 2H), 7.51 (d, *J* = 8.6 Hz, 2H), 7.47 (t, *J* = 1.4 Hz, 1H), 7.42 (t, *J* = 7.4 Hz, 2H), 6.17 (s, 1H), 3.85 (s, 2H), 3.06 (s, 3H), 2.41 (s, 1H); ^13^C NMR (100 MHz, CDCl_3_) δ 188.1 (C), 160.9 (C), 148.1 (C), 143.5 (C), 140.5 (C), 131.7 (CH), 129.5 (CH), 128.4 (CH), 127.8 (CH), 124.1 (CH), 97.5 (CH), 77.6 (C), 73.9 (CH), 42.4 (CH_3_), 38.3 (CH_2_); IR (neat): 3231, 3077, 2782, 2709, 1623, 1558, 1507, 1445, 1408, 1341, 1230, 1199, 1105, 1049, 939, 910, 856, 745, 710, 677, 614, 568, 500, 448 cm^–1^; MS (ESI, *m*/*z*): 321.12 [M + H]^+^; HRMS (ESI, *m*/*z*): calcd. for C_19_H_17_N_2_O_3_: 321.1234 [M + H]^+^, found: 321.1236.

#### 4-(Methyl­(prop-2-yn-1-yl)­amino)-4-phenylbut-3-en-2-one (**3m**)

4-Phenylbut-3-yn-2-one (**1m**) (800.0 mg, 5.55 mmol) and *N*-methylpropargylamine (**2**) (460.0 mg, 6.66 mmol) were employed to afford 94.6 mg (8%) of the indicated product **3m** as a dark orange oil (*R*_f_ = 0.23 in 4:1 hexane/ethyl acetate).

**3m:** ^1^H NMR (400 MHz, CDCl_3_) δ 7.43–7.41 (m, 3H), 7.26 (dd, *J* = 6.5, 3.0 Hz, 2H), 5.40 (s, 1H), 3.71 (s, 2H), 2.91 (s, 3H), 2.28 (t, *J* = 2.3 Hz, 1H), 1.69 (s, 3H); ^13^C NMR (100 MHz, CDCl_3_) δ 196.3 (C), 161.6 (C), 135.9 (C), 129.3 (CH), 128.7 (CH), 128.6 (CH), 103.1 (CH), 78.1 (C), 73.0 (CH), 42.1 (CH_3_), 37.7 (CH_2_), 29.9 (CH_3_); IR (neat): 3381, 3294, 3056, 2927, 2862, 1705, 1595, 1578, 1522, 1490, 1445, 1363, 1301, 1270, 1196, 1118, 1073, 1024, 953, 833, 765, 702, 618 cm^–1^; MS (ESI, *m*/*z*): 212.11 [M-H]^−^; HRMS (ESI, *m*/*z*): calcd. for C_14_H_14_NO: 212.1075 [M-H]^−^, found: 212.1075.

#### 1-(4-(Chloromethyl)­phenyl)-3-(methyl­(prop-2-yn-1-yl)­amino)-3-phenylprop-2-en-1-one (**3n**)

1-(4-(Chloromethyl)­phenyl)-3-phenylprop-2-yn-1-one (**1n**) (861.0 mg, 3.38 mmol) and *N*-methylpropargylamine (**2**) (280.0 mg, 4.06 mmol) were employed to afford 197.7 mg (18%) of the indicated product **3n** as a red oil (*R*_f_ = 0.10 in 4:1 hexane/ethyl acetate).

**3n:** ^1^H NMR (400 MHz, CDCl_3_) δ 7.88 (d, *J* = 8.2 Hz, 2H), 7.46–7.43 (m, 3H), 7.39 (d, *J* = 8.2 Hz, 2H), 7.31 (dd, *J* = 6.5, 2.9 Hz, 2H), 6.06 (s, 1H), 4.59 (s, 2H), 3.87 (s, 2H), 3.04 (s, 3H), 2.39 (s, 1H);^13^C NMR (100 MHz, CDCl_3_) δ 187.3 (C), 163.7 (C), 141.2 (C), 140.0 (C), 135.9 (C), 128.9 (CH), 128.6 (CH), 128.2 (CH), 128.1 (CH), 96.4 (CH), 77.9 (C), 73.2 (CH), 45.7 (CH_2_), 42.0 (CH_3_), 37.9 (CH_2_) (Note that two CH peaks overlap on each other); IR (neat): 3290, 3199, 3054, 2942, 1670, 1606, 1519, 1493, 1444, 1405, 1341, 1265, 1219, 1178, 1112, 1052, 1016, 976, 918, 852, 770, 728, 698, 572, 487 cm^–1^; MS (ESI, *m*/*z*): 322.10 [M-H]^−^; HRMS (ESI, *m*/*z*): calcd. for C_20_H_17_ClNO: 322.0999 [M-H]^−^, found: 322.1000.

#### 1-(4-Ethylphenyl)-3-(methyl­(prop-2-yn-1-yl)­amino)-3-phenylprop-2-en-1-one (**3o**)

1-(4-Ethylphenyl)-3-phenylprop-2-yn-1-one (**1o**) (130.0 mg, 5.55 mmol) and *N*-methylpropargylamine (**2**) (460.0 mg, 6.66 mmol) were employed to afford 456.5 mg (27%) of the indicated product **3o** as an orange oil (*R*_f_ = 0.21 in 4:1 hexane/ethyl acetate).

**3o:** ^1^H NMR (400 MHz, CDCl_3_) δ 7.85 (d, *J* = 8.0 Hz, 2H), 7.48–7.42 (m, 3H), 7.32 (dd, *J* = 6.2, 2.7 Hz, 2H), 7.23 (d, *J* = 8.0 Hz, 2H), 6.10 (s, 1H), 3.87 (s, 2H), 3.04 (s, 3H), 2.69 (q, *J* = 7.6 Hz, 2H), 2.37 (s, 1H), 1.26 (t, *J* = 7.6 Hz, 3H); ^13^C NMR (100 MHz, CDCl_3_) δ 188.0 (C), 163.2 (C), 147.7 (C), 138.8 (C), 136.3 (C), 128.9 (CH), 128.6 (CH), 128.4 (CH), 128.0 (CH), 127.6 (CH), 97.0 (CH), 78.2 (C), 73.1 (CH), 42.1 (CH_3_), 37.9 (CH_2_), 28.9 (CH_3_), 15.5 (CH_2_); IR (neat): 3279, 3242, 3082, 2964, 2931, 1606, 1518, 1398, 1340, 1212, 1178, 1115, 1045, 1011, 903, 831, 765, 697, 670, 569, 507, 469 cm^–1^; MS (ESI, *m*/*z*): 304.17 [M + H]^+^; HRMS (ESI, *m*/*z*): calcd. for C_21_H_22_NO: 304.1696 [M + H]^+^, found: 304.1700.

#### 1-(4-Methoxyphenyl)-3-(methyl­(prop-2-yn-1-yl)­amino)-3-phenylprop-2-en-1-one (**3p**)

1-(4-Methoxyphenyl)-3-phenylprop-2-yn-1-one (**1p**) (794.0 mg, 3.36 mmol) and *N*-methylpropargylamine (**2**) (280.0 mg, 4.03 mmol) were employed to afford 236.5 mg (23%) of the indicated product **3p** as an orange oil (*R*_f_ = 0.11 in 4:1 hexane/ethyl acetate).

**3p:** ^1^H NMR (400 MHz, CDCl_3_) δ 7.89 (d, *J* = 8.8 Hz, 2H), 7.44–7.42 (m, 3H), 7.31 (dd, *J* = 6.6, 2.9 Hz, 2H), 6.89 (d, *J* = 8.8 Hz, 2H), 6.07 (s, 1H), 3.90 (s, 2H), 3.84 (s, 3H), 3.03 (s, 3H), 2.36 (s, 1H); ^13^C NMR (100 MHz, CDCl_3_) δ 187.2 (C), 163.0 (C), 162.0 (C), 136.3 (C), 133.9 (C), 129.8 (CH), 128.8 (CH), 128.6 (CH), 128.3 (CH), 113.2 (CH), 96.9 (CH), 78.2 (C), 73.1 (CH), 55.3 (CH_3_), 42.1 (CH_3_), 37.9 (CH_2_); IR (neat): 3283, 3231, 2934, 2837, 1642, 1597, 1523, 1494, 1401, 1341, 1303, 1251, 1204, 1167, 1110, 1050, 1022, 919, 902, 843, 770, 749, 697, 670, 633, 569, 505, 468 cm^–1^; MS (ESI, *m*/*z*): 306.15 [M + H]^+^; HRMS (ESI, *m*/*z*): calcd. for C_20_H_20_NO_2_: 306.1489 [M + H]^+^, found: 306.1490.

#### 1-(3-Chlorophenyl)-3-(methyl­(prop-2-yn-1-yl)­amino)-3-phenylprop-2-en-1-one (**3q**)

1-(3-Chlorophenyl)-3-phenylprop-2-yn-1-one (**1q**) (897.0 mg, 3.73 mmol) and *N*-methylpropargylamine (**2**) (310.0 mg, 4.48 mmol) were employed to afford 791.0 mg (69%) of the indicated product **3q** as an orange solid (*R*_f_ = 0.23 in 4:1 hexane/ethyl acetate; mp 105.2–106.8 °C).

**3q:** ^1^H NMR (400 MHz, CDCl_3_) δ 7.85 (s, 1H), 7.75 (d, *J* = 7.6 Hz, 1H), 7.59–7.41 (m, 4H), 7.40 (d, *J* = 7.9 Hz, 1H), 7.32 (d, *J* = 7.7 Hz, 2H), 5.96 (s, 1H), 3.90 (s, 2H), 3.07 (s, 3H), 2.39 (s, 1H); ^13^C NMR (100 MHz, CDCl_3_) δ 186.2 (C), 164.3 (C), 142.9 (C), 136.0 (C), 134.0 (CH), 130.7 (CH), 129.3 (CH), 129.2 (C), 128.6 (CH), 128.2 (CH), 127.9 (CH), 125.8 (CH), 96.1 (CH), 77.9 (C), 73.4 (CH), 42.3 (CH_3_), 38.4 (CH_2_); IR (neat): 3270, 3060, 2931, 2862, 1670, 1621, 1559, 1463, 1397, 1327, 1296, 1226, 1200, 1147, 1087, 1052, 986, 936, 906, 762, 723, 697, 672, 648, 564, 514, 470, 457 cm^–1^; MS (ESI, *m*/*z*): 310.10 [M + H]^+^; HRMS (ESI, *m*/*z*): calcd. for C_19_H_17_ClNO: 310.0999 [M + H]^+^, found: 310.0997.

#### 1-(4-Fluorophenyl)-3-(methyl­(prop-2-yn-1-yl)­amino)-3-phenylprop-2-en-1-one (**3r**)

1-(4-Fluorophenyl)-3-phenylprop-2-yn-1-one (**1r**) (966.0 mg, 4.31 mmol) and *N*-methylpropargylamine (**2**) (360.0 mg, 5.17 mmol) were employed to afford 652.0 mg (52%) of the indicated product **3r** as a red oil (*R*_f_ = 0.16 in 4:1 hexane/ethyl acetate).

**3r:** ^1^H NMR (400 MHz, CDCl_3_) δ 7.83–7.75 (m, 2H), 7.37–7.31 (m, 3H), 7.22–7.19 (m, 2H), 6.97–6.93 (m, 2H), 5.93 (s, 1H), 3.78 (s, 2H), 2.94 (s, 3H), 2.28 (s, 1H); ^13^C NMR (100 MHz, CDCl_3_) δ 186.7 (C), 164.5 (d, ^1^ *J* = 250.9 Hz, C), 163.7 (C), 137.4 (d, ^4^ *J* = 2.7 Hz, C), 136.0 (C), 130.1 (d, ^3^ *J* = 8.8 Hz, CH), 129.0 (CH), 128.7 (CH), 128.2 (CH), 114.9 (d, ^2^ *J* = 21.6 Hz, CH), 96.3 (CH), 78.0 (C), 73.2 (CH), 42.1 (CH_3_), 37.9 (CH_2_); IR (neat): 3294, 3233, 3060, 2926, 1733, 1631, 1596, 1523, 1498, 1444, 1412, 1398, 1342, 1222, 1201, 1152, 1092, 1048, 976, 918, 848, 789, 772, 749, 699, 671, 598, 570, 508 cm^–1^; MS (ESI, *m*/*z*): 294.13 [M + H]^+^; HRMS (ESI, *m*/*z*): calcd. for C_19_H_17_FNO: 294.1294 [M + H]^+^, found: 294.1294.

#### 4-(3-(Methyl­(prop-2-yn-1-yl)­amino)-3-phenylacryloyl)­benzonitrile (**3s**)

4-(3-Phenylpropioloyl)­benzonitrile (**1s**) (600.4 mg, 2.60 mmol) and *N*-methylpropargylamine (**2**) (220.0 mg, 3.12 mmol) were employed to afford 224.0 mg (29%) of the indicated product **3s** as an orange solid (*R*_f_ = 0.06 in 4:1 hexane/ethyl acetate; mp 102.9–103.7 °C).

**3s:** ^1^H NMR (400 MHz, CDCl_3_) δ 7.81 (d, *J* = 6.9 Hz, 2H), 7.56 (d, *J* = 6.9 Hz, 2H), 7.37–7.34 (m, 3H), 7.21–7.18 (m, 2H), 5.89 (s, 1H), 3.81 (s, 2H), 3.00 (s, 3H), 2.29 (s, 1H);^13^C NMR (100 MHz, CDCl_3_) δ 186.3 (C), 164.9 (C), 145.2 (C), 135.7 (C), 132.0 (CH), 129.4 (CH), 128.9 (CH), 128.3 (CH), 128.2 (CH), 118.7 (C), 114.0 (C), 95.9 (CH), 77.2 (C), 73.6 (CH), 42.3 (CH_3_), 38.1 (CH_2_); IR (neat): 3262, 3061, 2981, 2228, 1633, 1518, 1489, 1415, 1397, 1376, 1340, 1289, 1208, 1177, 1108, 1054, 1017, 975, 927, 902, 857, 791, 777, 673, 614i 593, 566, 538, 508, 466, 432 cm^–1^; MS (ESI, *m*/*z*): 301.13 [M + H]^+^; HRMS (ESI, *m*/*z*): calcd. for C_20_H_17_N_2_O: 301.1341 [M + H]^+^, found: 301.1335.

#### 3-(Methyl­(prop-2-yn-1-yl)­amino)-1-(3-nitrophenyl)-3-phenylprop-2-en-1-one (**3t**)

1-(3-Nitrophenyl)-3-phenylprop-2-yn-1-one (**1t**) (564.0 mg, 2.24 mmol) and *N*-methylpropargylamine (**2**) (190.0 mg, 2.69 mmol) were employed to afford 401.0 mg (56%) of the indicated product **3t** as a yellow solid (*R*_f_ = 0.03 in 4:1 hexane/ethyl acetate; mp 109.2–110.5 °C).

**3t:** ^1^H NMR (400 MHz, CDCl_3_) δ 8.57 (s, 1H), 8.15 (d, *J* = 8.2 Hz, 1H), 8.05 (d, *J* = 7.7 Hz, 1H), 7.43 (t, *J* = 7.94 Hz, 1H), 7.37–7.32 (m, 3H), 7.20 (dd, *J* = 6.0, 3.3 Hz, 2H), 5.93 (s, 1H), 3.81 (s, 2H), 3.01 (s, 3H), 2.31 (s, 1H); ^13^C NMR (100 MHz, CDCl_3_) δ 185.4 (C), 165.0 (C), 148.0 (C), 142.9 (C), 135.7 (C), 133.6 (CH), 129.3 (CH), 129.2 (CH), 128.8 (CH), 128.2 (CH), 125.2 (CH), 122.7 (CH), 95.6 (CH), 77.6 (C), 73.6 (CH), 42.3 (CH_3_), 38.2 (CH_2_); IR (neat): 3229, 3072, 3059, 2913, 1633, 1607, 1509, 1411, 1372, 1346, 1315, 1260, 1205, 1157, 1112, 1077, 1044, 980, 922, 877, 820, 792, 772, 708, 676, 614, 576, 508, 473 cm^–1^; MS (ESI, *m*/*z*): 321.12 [M + H]^+^; HRMS (ESI, *m*/*z*): calcd. for C_19_H_17_N_2_O_3_: 321.1239 [M + H]^+^, found: 321.1238.

#### 3-(4-Bromophenyl)-1-(4-fluorophenyl)-3-(methyl­(prop-2-yn-1-yl)­amino)­prop-2-en-1-one (**3u**)

3-(4-Bromophenyl)-1-(4-fluorophenyl)­prop-2-yn-1-one (**1u**) (633.0 mg, 2.09 mmol) and *N*-methylpropargylamine (**2**) (170.0 mg, 2.51 mmol) were employed to afford 454.0 mg (58%) of the indicated product **3u** as a yellow oil (*R*_f_ = 0.37 in 4:1 hexane/ethyl acetate).

**3u:** ^1^H NMR (400 MHz, CDCl_3_) δ 7.89 (dd, *J* = 8.6, 5.6 Hz, 2H), 7.58 (d, *J* = 8.3 Hz, 2H), 7.19 (d, *J* = 8.3 Hz, 2H), 7.06 (t, *J* = 8.6 Hz, 2H), 6.03 (s, 1H), 3.87 (s, 2H), 3.04 (s, 3H), 2.38 (s, 1H);^13^C NMR (100 MHz, CDCl_3_) δ 186.7 (C), 164.8 (d, ^1^ *J* = 251.5 Hz, C), 162.5 (C), 137.3 (d, ^4^ *J* = 2.5 Hz, C), 135.1 (C), 132.1 (CH), 130.2 (d, ^3^ *J* = 8.9 Hz, CH), 130.0 (CH), 123.4 (C), 115.1 (d, ^2^ *J* = 21.7 Hz, CH), 96.7 (CH), 77.8 (C), 73.6 (CH), 42.2 (CH_3_), 38.1 (CH_2_); IR (neat): 3295, 3225, 3066, 2925, 1631, 1594, 1520, 1500, 1489, 1397, 1341, 1221, 1199, 1151, 1069, 1043, 906, 848, 823, 786, 720, 673, 612, 566, 501, 474 cm^–1^; MS (ESI, *m*/*z*): 372.04 [M + H]^+^; HRMS (ESI, *m*/*z*): calcd. for C_19_H_16_BrFNO: 372.0399 [M + H]^+^, found: 372.0381.

#### Synthesis of 3-(Methyl­(3-phenylprop-2-yn-1-yl)­amino)-1,3-diphenylprop-2-en-1-one (**3v**)

To a stirred solution of *N*-methyl-*N*-propargyl β-enaminone **3a** (463.0 mg, 1.68 mmol) in DMF (2.50 mL) at room temperature under argon was added (*i*-Pr)_2_NH (3.40 mL), PdCl_2_(PPh_3_)_2_ (24.0 mg, 0.03 mmol), CuI (6.0 mg, 0.03 mmol), and iodobenzene (215.0 mg, 1.05 mmol). The resulting mixture was then stirred at room temperature for approximately 6 h (The progress of the reaction was followed by routine TLC for the disappearance of β-enaminone **3a** using hexane/ethyl acetate (4:1) as the eluent). After the reaction was over, without removing DMF, ethyl acetate (30 mL) and saturated aqueous NH_4_Cl solution (30 mL) were added. After the layers were separated, the aqueous phase was extracted with ethyl acetate (2 × 30 mL). The combined organic phases were dried over MgSO_4_ and evaporated on a rotary evaporator to give a crude product, which was purified by flash chromatography on silica gel using hexane, followed by hexane/ethyl acetate (4:1), as the eluent to afford 334.0 mg (57%) of the indicated internal alkyne-tethered *N*-methyl-*N*-propargyl β-enaminone **3v** as a yellowish-brown oil (*R*_f_ = 0.28 in 4:1 hexane/ethyl acetate).

**3v:** ^1^H NMR (400 MHz, CDCl_3_) δ 7.95 (d, *J* = 7.5 Hz, 2H), 7.53–7.47 (m, 6H), 7.44–7.38 (m, 7H), 6.17 (s, 1H), 4.12 (s, 2H), 3.13 (s, 3H); ^13^C NMR (100 MHz, CDCl_3_) δ 188.1 (C), 163.6 (C), 141.3 (C), 136.3 (C), 131.8 (CH), 131.0 (CH), 128.9 (CH), 128.7 (CH), 128.4 (CH), 128.4 (CH), 128.0 (CH), 127.8 (CH), 127.2 (CH), 122.5 (C), 96.7 (CH), 85.0 (C), 83.5 (C), 43.0 (CH_3_), 38.1 (CH_2_); IR (neat): 3056, 3028, 2926, 1684, 1634, 1596, 1576, 1518, 1487, 1442, 1405, 1339, 1256, 1202, 1176, 1157, 1115, 1070, 1047, 1022, 1000, 978, 919, 905, 827, 756, 690, 665, 632, 613, 579, 528, 507 cm^–1^; MS (ESI, *m*/*z*): 352.17 [M + H]^+^; HRMS (ESI, *m*/*z*): calcd. for C_25_H_22_NO: 352.1701 [M + H]^+^, found: 352.1697.

### General Procedure for the Synthesis of 1,2,3,4-Tetrasubstituted Benzenes 5 under Gold Catalysis (Table 4)

To a stirred solution of the corresponding *N-*methyl*-N*-propargyl β-enaminone **3** (1.00 mmol) in toluene (6 mL) under argon was added dialkyl acetylenedicarboxylate **4** (1.3 mmol), AuCl (0.10 mmol) ve AgSbF_6_ (0.15 mmol), and the resulting mixture was refluxed for approximately 5 h in an oil bath (The progress of the reaction was monitored by routine TLC analysis for the disappearance of β-enaminone **3** using hexane/ethyl acetate (4:1) as the eluent). After the reaction was over, the solvent was removed on a rotary evaporator, and ethyl acetate (30 mL) and a saturated aqueous solution of NaCl (15 mL) were added. After the layers were separated, the aqueous layer was extracted with ethyl acetate (2 × 30 mL) again. The combined organic layers were dried over MgSO_4_ and evaporated on a rotary evaporator to give the crude product, which was purified by flash chromatography on silica gel using hexane/ethyl acetate (4:1) as the eluent to afford the corresponding 1,2,3,4-tetrasubstituted benzene derivative **5**.

#### Dimethyl 2-Benzoyl-[1,1′-biphenyl]-3,4-dicarboxylate (**5a**)

3-(Methyl­(prop-2-yn-1-yl)­amino)-1,3-diphenylprop-2-en-1-one (**3a**) (110.0 mg, 0.40 mmol), dimethyl acetylenedicarboxylate (**4a**) (74.0 mg, 0.52 mmol), AuCl (9.0 mg, 0.04 mmol), and AgSbF_6_ (21.0 mg, 0.06 mmol) were employed to afford 54.7 mg (35%) of the indicated product **3a** as a yellowish-orange oil (*R*_f_ = 0.33 in 4:1 hexane/ethyl acetate).

**5a:** ^1^H NMR (400 MHz) δ 8.05 (d, *J* = 8.0 Hz, 1H), 7.50 (d, *J* = 8.0 Hz, 1H), 7.47 (d, *J* = 6.8 Hz, 2H), 7.37 (t, *J* = 7.4 Hz, 1H), 7.21 (t, *J* = 7.6 Hz, 2H), 7.07 (s, 5H), 3.87 (s, 3H), 3.51 (s, 3H); ^13^C NMR (100 MHz, CDCl_3_) δ 196.9 (C), 168.5 (C), 165.6 (C), 144.3 (C), 138.7 (C), 136.7 (C), 136.5 (C), 135.9 (CH), 133.5 (CH), 129.8 (CH), 129.7 (CH), 129.0 (CH), 128.9 (C), 128.3 (CH), 128.2 (C), 127.9 (CH), 53.0 (CH_3_), 52.5 (CH_3_) (Note that two CH peaks overlap on each other); IR (neat): 2950, 1724, 1669, 1596, 1579, 1463, 1434, 1409, 1295, 1263, 1237, 1196, 1168, 1132, 1002, 968, 942, 845, 799, 763, 742, 713, 699, 671, 559 cm^–1^; MS (ESI, *m*/*z*): 375.12 [M + H]^+^; HRMS (ESI, *m*/*z*): calcd. for C_23_H_19_O_5_: 375.1232 [M + H]^+^, found: 375.1238. The spectral data are in agreement with those reported previously for this compound.^40^

#### Diethyl 2-Benzoyl-[1,1′-biphenyl]-3,4-dicarboxylate (**5a′**)

3-(Methyl­(prop-2-yn-1-yl)­amino)-1,3-diphenylprop-2-en-1-one (**3a**) (204.0 mg, 0.74 mmol), diethyl acetylenedicarboxylate (**4b**) (160.0 mg, 0.96 mmol), AuCl (17.0 mg, 0.07 mmol), and AgSbF_6_ (38.0 mg, 0.11 mmol) were employed to afford 74.3 mg (25%) of the indicated product **5a′** as a yellow oil (*R*_f_ = 0.49 in 4:1 hexane/ethyl acetate).

**5a′:** ^1^H NMR (400 MHz, CDCl_3_) δ 8.04 (d, *J* = 8.0 Hz, 1H), 7.48 (d, *J* = 8.0 Hz, 2H), 7.45 (d, *J* = 1.4 Hz, 1H), 7.36 (t, *J* = 7.4 Hz, 1H), 7.20 (t, *J* = 7.8 Hz, 2H), 7.06 (s, 5H), 4.32 (q, *J* = 7.1 Hz, 2H), 3.96 (q, *J* = 7.2 Hz, 2H), 1.31 (t, *J* = 7.1 Hz, 3H), 0.86 (t, *J* = 7.2 Hz, 3H); ^13^C NMR (100 MHz, CDCl_3_) δ 197.0 (C), 167.9 (C), 165.3 (C), 144.1 (C), 138.6 (C), 136.8 (C), 136.5 (C), 136.0 (C), 133.4 (CH), 129.8 (CH), 129.8 (CH), 129.3 (C), 129.0 (CH), 128.3 (CH), 128.1 (CH), 128.1 (CH), 127.8 (CH), 62.0 (CH_2_), 61.5 (CH_2_), 14.1 (CH_3_), 13.6 (CH_3_); IR (neat): 3057, 2981, 2935, 2903, 1721, 1671, 1596, 1566, 1465, 1446, 1414, 1388, 1366, 1293, 1260, 1173, 1134, 1067, 1017, 973, 749, 800, 764, 742, 701, 672, 567, 440 cm^–1^; MS (ESI, *m*/*z*): 425.14 [M + Na]^+^; HRMS (ESI, *m*/*z*): calcd. for C_25_H_22_O_5_Na: 425.1365 [M + Na]^+^, found: 425.1365.

#### Dimethyl 2-Benzoyl-4′-methyl-[1,1′-biphenyl]-3,4-dicarboxylate (**5b**)

3-(Methyl­(prop-2-yn-1-yl)­amino)-1-phenyl-3-(p-tolyl)­prop-2-en-1-one (**3b**) (143.0 mg, 0.49 mmol), dimethyl acetylenedicarboxylate (**4a**) (90.0 mg, 0.64 mmol), AuCl (11.0 mg, 0.05 mmol), and AgSbF_6_ (30.0 mg, 0.07 mmol) were employed to afford 67.5 mg (36%) of the indicated product **5b** as a dark yellow solid (*R*_f_ = 0.24 in 4:1 hexane/ethyl acetate; mp 76.3–77.8 °C).

**5b:** ^1^H NMR (400 MHz, CDCl_3_) δ 8.12 (d, *J* = 8.0 Hz, 1H), 7.57 (t, *J* = 7.8 Hz, 3H), 7.47 (t, *J* = 7.4 Hz, 1H), 7.31 (t, *J* = 7.7 Hz, 2H), 7.04 (d, *J* = 7.9 Hz, 2H), 6.96 (d, *J* = 7.8 Hz, 2H), 3.96 (s, 3H), 3.63 (s, 3H), 2.23 (s, 3H); ^13^C NMR (100 MHz, CDCl_3_) δ 197.0 (C), 168.6 (C), 165.6 (C), 144.4 (C), 138.8 (C), 137.9 (C), 136.7 (C), 136.6 (C), 133.5 (C), 132.9 (C), 129.8 (CH), 129.5 (CH), 128.8 (CH), 128.7 (CH), 128.6 (CH), 128.3 (CH), 128.2 (CH), 52.9 (CH_3_), 52.4 (CH_3_), 21.2 (CH_3_); IR (neat): 3058, 2993, 2947, 1740, 1726, 1663, 1590, 1575, 1433, 1298, 1260, 1237, 1195, 1168, 1132, 1065, 1000, 949, 840, 820, 796, 743, 713, 676, 562, 515 cm^–1^; MS (ESI, *m*/*z*): 389.14 [M + H]^+^; HRMS (ESI, *m*/*z*): calcd. for C_24_H_21_O_5_: 389.1389 [M + H]^+^, found: 389.1389.

#### Dimethyl 2-Benzoyl-3′-methyl-[1,1′-biphenyl]-3,4-dicarboxylate (**5c**)

3-(Methyl­(prop-2-yn-1-yl)­amino)-1-phenyl-3-(m-tolyl)­prop-2-en-1-one (**3c**) (186.0 mg, 0.64 mmol), dimethyl acetylenedicarboxylate (**4a**) (120.0 mg, 0.83 mmol), AuCl (15.0 mg, 0.06 mmol), and AgSbF_6_ (33.0 mg, 0.10 mmol) were employed to afford 81.8 mg (33%) of the indicated product **5c** as a yellow oil (*R*_f_ = 0.34 in 4:1 hexane/ethyl acetate).

**5c:** ^1^H NMR (400 MHz, CDCl_3_) δ 8.12 (d, *J* = 8.0 Hz, 1H), 7.59 (d, *J* = 8.0 Hz, 1H), 7.55 (s, 1H), 7.54 (d, *J* = 1.4 Hz, 1H), 7.45 (t, *J* = 7.4 Hz, 1H), 7.29 (t, *J* = 7.8 Hz, 2H), 7.06–7.00 (m, 1H), 6.95 (s, 2H), 6.93 (d, *J* = 0.6 Hz, 1H), 3.96 (s, 3H), 3.62 (s, 3H), 2.18 (s, 3H); ^13^C NMR (100 MHz, CDCl_3_) δ 197.0 (C), 168.5 (C), 165.6 (C), 144.3 (C), 138.9 (C), 137.4 (C), 136.8 (C), 136.4 (C), 135.8 (C), 133.3 (C), 130.4 (CH), 129.7 (CH), 128.9 (CH), 128.4 (CH), 128.2 (CH), 127.8 (CH), 126.7 (CH), 52.9 (CH_3_), 52.4 (CH_3_), 21.2 (CH_3_) (Note that two CH peaks overlap on each other); IR (neat): 3027, 2951, 2924, 2864, 1726, 1670, 1596, 1580, 1566, 1460, 1435, 1297, 1265, 1242, 1195, 1166, 1131, 1097, 1072, 1002, 943, 891, 843, 799, 787, 715, 672, 571, 468 cm^–1^; MS (ESI, *m*/*z*): 411.12 [M + Na]^+^; HRMS (ESI, *m*/*z*): calcd. for C_24_H_20_O_5_Na: 411.1208 [M + Na]^+^, found: 411.1208.

#### Dimethyl 2-Benzoyl-4′-(*tert*-butyl)-[1,1′-biphenyl]-3,4-dicarboxylate (**5d**)

3-(4-(*tert*-Butyl)­phenyl)-3-(methyl­(prop-2-yn-1-yl)­amino)-1-phenylprop-2-en-1-one (**3d**) (56.0 mg, 0.17 mmol), dimethyl acetylenedicarboxylate (**4a**) (30.0 mg, 0.22 mmol), AuCl (4.0 mg, 0.02 mmol), and AgSbF_6_ (10.0 mg, 0.03 mmol) were employed to afford 14.0 mg (19%) of the indicated product **5d** as a yellow solid (*R*_f_ = 0.33 in 4:1 hexane/ethyl acetate; mp 71.8–73.0 °C).

**5d:** ^1^H NMR (400 MHz, CDCl_3_) δ 8.13 (d, *J* = 8.0 Hz, 1H), 7.61 (d, *J* = 8.0 Hz, 1H), 7.49 (d, *J* = 7.1 Hz, 2H), 7.42 (t, *J* = 7.4 Hz, 1H), 7.26 (t, *J* = 7.8 Hz, 2H), 7.13 (d, *J* = 8.4 Hz, 2H), 7.04 (d, *J* = 8.4 Hz, 2H), 3.97 (s, 3H), 3.61 (s, 3H), 1.20 (s, 9H); ^13^C NMR (100 MHz, CDCl_3_) δ 197.4 (C), 168.7 (C), 165.7 (C), 151.0 (C), 144.6 (C), 138.9 (C), 137.1 (C), 136.5 (C), 133.2 (C), 132.9 (C), 129.6 (CH), 129.5 (CH), 128.9 (CH), 128.9 (CH), 128.5 (CH), 128.2 (CH), 124.8 (CH), 53.0 (CH_3_), 52.5 (CH_3_), 34.6 (C), 31.2 (3CH_3_); IR (neat): 2906, 2870, 2957, 1739, 1723, 1672, 1596, 1580, 1460, 1398, 1366, 1298, 1264, 1194, 1166, 1135, 1064, 996, 964, 941, 841, 804, 710, 679, 585 cm^–1^; MS (ESI, *m*/*z*): 453.17 [M + Na]^+^; HRMS (ESI, *m*/*z*): calcd. for C_27_H_26_O_5_Na: 453.1673 [M + Na]^+^, found: 453.1650.

#### Dimethyl 2-Benzoyl-3′-methoxy-[1,1′-biphenyl]-3,4-dicarboxylate (**5e**)

3-(3-Methoxyphenyl)-3-(methyl­(prop-2-yn-1-yl)­amino)-1-phenylprop-2-en-1-one (**3e**) (272.0 mg, 0.89 mmol), dimethyl acetylenedicarboxylate (**4a**) (160.0 mg, 1.16 mmol), AuCl (21.0 mg, 0.09 mmol), and AgSbF_6_ (45.0 mg, 0.13 mmol) were employed to afford 56.3 mg (16%) of the indicated product **5e** as an orange solid (*R*_f_ = 0.23 in 4:1 hexane/ethyl acetate; mp 142.3–143.3 °C).

**5e:** ^1^H NMR (400 MHz, CDCl_3_) δ 8.07 (d, *J* = 8.0 Hz, 1H), 7.68 (d, *J* = 7.1 Hz, 2H), 7.57 (d, *J* = 8.0 Hz, 1H), 7.50 (t, *J* = 7.4 Hz, 1H), 7.35 (t, *J* = 7.8 Hz, 2H), 7.20 (s, 1H), 7.17 (d, *J* = 7.8 Hz, 1H), 6.87 (t, *J* = 7.1 Hz, 1H), 6.66 (d, *J* = 8.2 Hz, 1H), 3.94 (s, 3H), 3.60 (s, 3H), 3.41 (s, 3H); ^13^C NMR (100 MHz, CDCl_3_) δ 196.0 (C), 168.4 (C), 165.9 (C), 155.9 (C), 144.2 (C), 136.9 (C), 136.6 (C), 136.1 (C), 133.2 (CH), 131.3 (CH), 130.0 (CH), 129.6 (C), 128.6 (CH), 128.6 (CH), 128.1 (CH), 124.9 (C), 120.2 (CH), 110.3 (CH), 54.8 (OCH_3_), 52.8 (CH_3_), 52.3 (CH_3_) (Note that two CH peaks overlap on each other); IR (neat): 3101, 3062, 2944, 2851, 1726, 1671, 1593, 1579, 1498, 1463, 1436, 1410, 1319, 1302, 1273, 1198, 1168, 1067, 1026, 1002, 971, 944, 845, 798, 753, 718, 671, 626, 568, 543, 508 cm^–1^; MS (ESI, *m*/*z*): 427.12 [M + Na]^+^; HRMS (ESI, *m*/*z*): calcd. for C_24_H_20_O_6_Na: 427.1158 [M + Na]^+^, found: 427.1158.

#### Dimethyl 2-Benzoyl-4′-(methylthio)-[1,1′-biphenyl]-3,4-dicarboxylate (**5f**)

3-(Methyl­(prop-2-yn-1-yl)­amino)-3-(4-(methylthio)­phenyl)-1-phenylprop-2-en-1-one (**3f**) (139.0 mg, 0.43 mmol), dimethyl acetylenedicarboxylate (**4a**) (80.0 mg, 0.56 mmol), AuCl (10.0 mg, 0.04 mmol), and AgSbF_6_ (22.0 mg, 0.07 mmol) were employed to afford 63.0 mg (35%) of the indicated product **5f** as a yellow solid (*R*_f_ = 0.21 in 4:1 hexane/ethyl acetate; mp 97.3–98.6 °C).

**5f:** ^1^H NMR (400 MHz, CDCl_3_) δ 8.12 (d, *J* = 8.0 Hz, 1H), 7.54 (d, *J* = 8.0 Hz, 2H), 7.48 (d, *J* = 8.4 Hz, 2H), 7.17 (m, 5H), 7.10 (d, *J* = 8.4 Hz, 1H), 3.95 (s, 3H), 3.59 (s, 3H), 2.46 (s, 3H); ^13^C NMR (100 MHz, CDCl_3_) δ 195.7 (C), 168.5 (C), 165.6 (C), 146.9 (C), 144.3 (C), 138.5 (C), 136.5 (C), 135.9 (C), 132.9 (C), 130.2 (CH), 129.6 (CH), 128.9 (CH), 128.7 (C), 128.2 (CH), 128.1 (CH), 127.9 (CH), 124.6 (CH), 52.9 (CH_3_), 52.4 (CH_3_), 14.7 (CH_3_); IR (neat): 3106, 2952, 2922, 2871, 1720, 1663, 1583, 1433, 1340, 1239, 1168, 1135, 1087, 1069, 941, 839, 804, 752, 741, 698, 576, 503, 480 cm^–1^; MS (ESI, *m*/*z*): 421.11 [M + H]^+^; HRMS (ESI, *m*/*z*): calcd. for C_24_H_21_SO_5_: 421.1104 [M + H]^+^, found: 421.1088.

#### Dimethyl 2-Benzoyl-4′-bromo-[1,1′-biphenyl]-3,4-dicarboxylate (**5g**)

3-(4-Bromophenyl)-3-(methyl­(prop-2-yn-1-yl)­amino)-1-phenylprop-2-en-1-one (**3g**) (212.0 mg, 0.60 mmol), dimethyl acetylenedicarboxylate (**4a**) (110.0 mg, 0.78 mmol), AuCl (14.0 mg, 0.06 mmol), and AgSbF_6_ (31.0 mg, 0.09 mmol) were employed to afford 85.9 mg (32%) of the indicated product **5g** as a brown solid (*R*_f_ = 0.29 in 4:1 hexane/ethyl acetate; mp 119.1–120.3 °C).

**5g:** ^1^H NMR (400 MHz, CDCl_3_) δ 8.06 (d, *J* = 8.0 Hz, 1H), 7.50 (d, *J* = 3.5 Hz, 1H), 7.48–7.47 (m, 2H), 7.42 (t, *J* = 7.4 Hz, 1H), 7.26 (d, *J* = 7.8 Hz, 2H), 7.22 (d, *J* = 8.4 Hz, 2H), 6.95 (d, *J* = 8.4 Hz, 2H), 3.87 (s, 3H), 3.55 (s, 3H); ^13^C NMR (100 MHz, CDCl_3_) δ 196.5 (C), 168.2 (C), 165.4 (C), 144.1 (C), 137.5 (C), 136.6 (C), 136.5 (C), 134.8 (C), 133.8 (C), 131.3 (CH), 131.1 (CH), 129.8 (CH), 129.3 (CH), 129.0 (C), 128.5 (CH), 128.4 (CH), 122.7 (CH), 53.0 (CH_3_), 52.6 (CH_3_); IR (neat): 3001, 2950, 2924, 2849, 1748, 1722, 1668, 1585, 1488, 1434, 1392, 1252, 1170, 1131, 1060, 1009, 938, 826, 750, 706, 673, 555 cm^–1^; MS (ESI, *m*/*z*): 453.03 [M + H]^+^; HRMS (ESI, *m*/*z*): calcd. for C_23_H_18_BrO_5_: 453.0338 [M + H]^+^, found: 453.0338.

#### Dimethyl 2-Benzoyl-4′-chloro-[1,1′-biphenyl]-3,4-dicarboxylate (**5h**)

3-(4-Chlorophenyl)-3-(methyl­(prop-2-yn-1-yl)­amino)-1-phenylprop-2-en-1-one (**3h**) (188.3 mg, 0.61 mmol), dimethyl acetylenedicarboxylate (**4a**) (110.0 mg, 0.79 mmol), AuCl (14.2 mg, 0.06 mmol), and AgSbF_6_ (31.6 mg, 0.09 mmol) were employed to afford 77 mg (31%) of the indicated product **5h** as an orange solid (*R*_f_ = 0.37 in 4:1 hexane/ethyl acetate; mp 116.7–117.8 °C).

**5h:** ^1^H NMR (400 MHz, CDCl_3_) δ 8.06 (d, *J* = 8.0 Hz, 1H), 7.49 (d, *J* = 5.6 Hz, 1H), 7.47 (d, *J* = 5.0 Hz, 2H), 7.41 (t, *J* = 7.4 Hz, 1H), 7.24 (t, *J* = 7.8 Hz, 2H), 7.06 (d, *J* = 8.4 Hz, 2H), 7.01 (d, *J* = 8.5 Hz, 2H), 3.87 (s, 3H), 3.54 (s, 3H); ^13^C NMR (100 MHz, CDCl_3_) δ 196.5 (C), 168.3 (C), 165.4 (C), 144.2 (C), 137.4 (C), 136.6 (C), 136.6 (C), 134.4 (C), 134.4 (C), 133.8 (C), 131.0 (CH), 129.8 (CH), 129.3 (CH), 129.0 (CH), 128.5 (CH), 128.4 (CH), 128.1 (CH), 53.0 (CH_3_), 52.6 (CH_3_); IR (neat): 3008, 2956, 1724, 1662, 1596, 1495, 1293, 1269, 1196, 1137, 1065, 938, 852, 752, 723, 694, 674, 567, 525 cm^–1^; MS (ESI, *m*/*z*): 409.08 [M + H]^+^; HRMS (ESI, *m*/*z*): calcd. for C_23_H_18_ClO_5_: 409.0843 [M + H]^+^, found: 409.0842.

#### Dimethyl 2-Benzoyl-3′-fluoro-[1,1′-biphenyl]-3,4-dicarboxylate (**5i**)

3-(3-Fluorophenyl)-3-(methyl­(prop-2-yn-1-yl)­amino)-1-phenylprop-2-en-1-one (**3i**) (205.9 mg, 0.70 mmol), dimethyl acetylenedicarboxylate (**4a**) (130.0 mg, 0.91 mmol), AuCl (16.0 mg, 0.07 mmol), and AgSbF_6_ (38.0 mg, 0.11 mmol) were employed to afford 96.8 mg (35%) of the indicated product **5i** as an orange oil (*R*_f_ = 0.25 in 4:1 hexane/ethyl acetate).

**5i:** ^1^H NMR (400 MHz, CDCl_3_) δ 8.15 (d, *J* = 8.0 Hz, 1H), 7.59 (d, *J* = 8.0 Hz, 2H), 7.57 (s, 1H), 7.55 (d, *J* = 1.2 Hz, 1H), 7.48 (t, *J* = 7.4 Hz, 1H), 7.32 (t, *J* = 7.8 Hz, 2H), 7.13 (t, *J* = 7.8 Hz, 1H), 6.94 (d, *J* = 7.7 Hz, 1H), 6.87 (d, *J* = 1.8 Hz, 1H), 3.95 (s, 3H), 3.64 (s, 3H); ^13^C NMR (100 MHz, CDCl_3_) δ 196.5 (C), 168.2 (C), 165.4 (C), 161.9 (d, ^1^ *J* = 247.3 Hz, C), 144.1 (C), 138.0 (d, ^3^ *J* = 7.9 Hz, C), 137.3 (C), 137.2 (C), 136.5 (d, ^3^ *J* = 16.3 Hz, CH), 133.7 (CH), 129.7 (CH), 129.6 (C), 129.5 (C), 129.4 (CH), 128.5 (CH), 128.4 (CH), 125.6 (d, ^4^ *J* = 2.9 Hz, CH), 116.8 (d, ^2^ *J* = 21.0 Hz, CH), 115.2 (d, ^2^ *J* = 22.4 Hz, CH), 53.0 (CH_3_), 52.5 (CH_3_); IR (neat): 3029, 2996, 2952, 1726, 1657, 1579, 1459, 1401, 1297, 1151, 1129, 941, 907, 880, 847, 794, 750, 724, 697, 673, 646, 568, 523, 483 cm^–1^; MS (ESI, *m*/*z*): 393.11 [M + H]^+^; HRMS (ESI, *m*/*z*): calcd. for C_23_H_18_FO_5_: 393.1139 [M + H]^+^, found: 393.1138.

#### Dimethyl 2-Benzoyl-4′-fluoro-3′-methyl-[1,1′-biphenyl]-3,4-dicarboxylate (**5j**)

3-(4-Fluoro-3-methylphenyl)-3-(methyl­(prop-2-yn-1-yl)­amino)-1-phenylprop-2-en-1-one (**3j**) (280.0 mg, 0.91 mmol), dimethyl acetylenedicarboxylate (**4a**) (170.0 mg, 1.18 mmol), AuCl (21.0 mg, 0.09 mmol), and AgSbF_6_ (48.0 mg, 0.14 mmol) were employed to afford 115.0 mg (31%) of the indicated product **5j** as an orange oil (*R*_f_ = 0.29 in 4:1 hexane/ethyl acetate).

**5j:** ^1^H NMR (400 MHz, CDCl_3_) δ 8.12 (d, *J* = 8.0 Hz, 1H), 7.57 (d, *J* = 8.0 Hz, 1H), 7.53 (d, *J* = 7.2 Hz, 2H), 7.46 (t, *J* = 7.4 Hz, 1H), 7.30 (t, *J* = 7.7 Hz, 2H), 6.96 (d, *J* = 7.7 Hz, 1H), 6.92 (s, 1H), 6.76 (t, *J* = 8.9 Hz, 1H), 3.94 (s, 3H), 3.63 (s, 3H), 2.10 (s, 3H); ^13^C NMR (100 MHz, CDCl_3_) δ 196.9 (C), 168.4 (C), 165.5 (C), 161.0 (d, ^1^ *J* = 246.7 Hz, C), 144.4 (C), 137.8 (C), 136.7 (C), 136.5 (C), 133.5 (CH), 133.0 (d, ^3^ *J* = 5.5 Hz, CH), 131.5 (d, ^4^ *J* = 3.9 Hz, C), 129.6 (CH), 129.0 (CH), 128.8 (d, ^3^ *J* = 7.6 Hz, CH), 128.7 (C), 128.3 (CH), 128.3 (CH), 124.4 (d, ^2^ *J* = 17.6 Hz, C), 114.5 (d, ^2^ *J* = 22.7 Hz, CH), 52.9 (CH_3_), 52.4 (CH_3_), 14.3 (d, ^3^ *J* = 3.1 Hz, CH_3_); IR (neat): 3059, 2994, 2952, 1725, 1665, 1596, 1505, 1434, 1263, 1226, 1194, 1165, 1132, 1069, 944, 838, 796, 710, 673, 560, 514 cm^–1^; MS (ESI, *m*/*z*): 407.13 [M + H]^+^; HRMS (ESI, *m*/*z*): calcd. for C_24_H_20_FO_5_: 407.1295 [M + H]^+^, found: 407.1296.

#### Dimethyl 2-(3,5-Dichlorobenzoyl)-[1,1′-biphenyl]-3,4-dicarboxylate (**5k**)

3-(3,5-Dichlorophenyl)-3-(methyl­(prop-2-yn-1-yl)­amino)-1-phenylprop-2-en-1-one (**3k**) (166.3 mg, 0.48 mmol), dimethyl acetylenedicarboxylate (**4a**) (90.0 mg, 0.62 mmol), AuCl (11.0 mg, 0.05 mmol), and AgSbF_6_ (25.0 mg, 0.07 mmol) were employed to afford 65.0 mg (31%) of the indicated product **5k** as an orange solid (*R*_f_ = 0.21 in 4:1 hexane/ethyl acetate; mp 87.2–88.3 °C).

**5k:** ^1^H NMR (400 MHz, CDCl_3_) δ 8.17 (d, *J* = 8.0 Hz, 1H), 7.63 (d, *J* = 8.1 Hz, 1H), 7.37 (t, *J* = 1.79 Hz, 1H), 7.32 (d, *J* = 1.7 Hz, 2H), 7.21 (s, 1H), 7.20 (s, 1H), 7.19 (s, 1H), 7.13 (t, *J* = 4.6 Hz, 2H), 3.97 (s, 3H), 3.61 (s, 3H); ^13^C NMR (100 MHz, CDCl_3_) δ 194.6 (C), 168.2 (C), 165.4 (C), 142.8 (C), 139.1 (C), 138.8 (C), 136.6 (C), 135.7 (C), 135.2 (CH), 132.8 (CH), 129.7 (C), 129.7 (CH), 129.4 (CH), 128.8 (C), 128.6 (CH), 128.2 (CH), 127.7 (CH), 53.1 (CH_3_), 52.6 (CH_3_); IR (neat): 3081, 3031, 2996, 2950, 1730, 1664, 1563, 1463, 1429, 1295, 1261, 1234, 1179, 1134, 1071, 960, 872, 787, 758, 739, 705, 669, 570, 479 cm^–1^; MS (ESI, *m*/*z*): 443.05 [M + H]^+^; HRMS (ESI, *m*/*z*): calcd. for C_23_H_17_Cl_2_O_5_: 443.0453 [M + H]^+^, found: 443.0453.

#### Dimethyl 2-Benzoyl-4′-nitro-[1,1′-biphenyl]-3,4-dicarboxylate (**5l**)

3-(Methyl­(prop-2-yn-1-yl)­amino)-3-(4-nitrophenyl)-1-phenylprop-2-en-1-one (**3l**) (278.0 mg, 0.87 mmol), dimethyl acetylenedicarboxylate (**4a**) (160.0 mg, 1.13 mmol), AuCl (20.0 mg, 0.09 mmol), and AgSbF_6_ (45.0 mg, 0.13 mmol) were employed to afford 134.7 mg (37%) of the indicated product **5l** as an orange solid (*R*_f_ = 0.18 in 4:1 hexane/ethyl acetate; mp 141.1–142.8 °C).

**5l:** ^1^H NMR (400 MHz, CDCl_3_) δ 8.12 (d, *J* = 8.0 Hz, 1H), 7.98 (d, *J* = 8.8 Hz, 2H), 7.55 (d, *J* = 8.0 Hz, 1H), 7.51 (d, *J* = 7.1 Hz, 2H), 7.45 (t, *J* = 6.3 Hz, 1H), 7.30 (t, *J* = 2.2 Hz, 2H), 7.27 (d, *J* = 4.0 Hz, 2H), 3.89 (s, 3H), 3.54 (s, 3H); ^13^C NMR (100 MHz, CDCl_3_) δ 195.9 (C), 167.9 (C), 165.2 (C), 147.5 (C), 143.6 (C), 142.9 (C), 136.6 (C), 136.4 (C), 136.3 (C), 134.2 (CH), 130.8 (CH), 129.9 (CH), 129.4 (CH), 128.8 (C), 128.7 (CH), 123.0 (CH), 53.1 (CH_3_), 52.8 (CH_3_) (Note that two CH peaks overlap on each other); IR (neat): 3007, 2955, 2848, 1726, 1657, 1595, 1517, 1432, 1349, 1294, 1268, 1196, 1137, 1065, 989, 938, 867, 848, 796, 743, 718, 964, 673, 573 cm^–1^; MS (ESI, *m*/*z*): 420.11 [M + H]^+^; HRMS (ESI, *m*/*z*): calcd. for C_23_H_18_NO_7_: 420.1083 [M + H]^+^, found: 420.1083.

#### Dimethyl 2-Acetyl-[1,1′-biphenyl]-3,4-dicarboxylate (**5m**)

4-(Methyl­(prop-2-yn-1-yl)­amino)-4-phenylbut-3-en-2-one (**3m**) (239.0 mg, 1.12 mmol), dimethyl acetylenedicarboxylate (**4a**) (210.0 mg, 1.46 mmol), AuCl (26.0 mg, 0.11 mmol), and AgSbF_6_ (58.0 mg, 0.17 mmol) were employed to afford 52.0 mg (15%) of the indicated product **5m** as an orange oil (*R*_f_ = 0.31 in 4:1 hexane/ethyl acetate).

**5m:** ^1^H NMR (400 MHz, CDCl_3_) δ 8.09 (d, *J* = 8.1 Hz, 1H), 7.63 (d, *J* = 8.1 Hz, 1H), 7.43 (d, *J* = 2.2 Hz, 2H), 7.42 (t, *J* = 2.1 Hz, 1H), 7.31–7.29 (m, 2H), 3.94 (s, 3H), 3.61 (s, 3H), 1.89 (s, 3H); ^13^C NMR (100 MHz, CDCl_3_) δ 203.3 (C), 168.4 (C), 165.5 (C), 145.6 (C), 137.9 (C), 136.7 (C), 136.6 (C), 129.5 (CH), 129.4 (CH), 129.2 (C), 128.9 (CH), 128.5 (CH), 128.1 (CH), 53.0 (CH_3_), 52.5 (CH_3_), 30.4 (CH_3_); IR (neat): 3232, 3192, 3005, 2951, 2918, 1726, 1692, 1634, 1566, 1496, 1462, 1407, 1351, 1294, 1258, 1195, 1155, 1093, 1069, 1046, 963, 922, 877, 846, 786, 773, 701, 646, 612, 576, 541 cm^–1^; MS (ESI, *m*/*z*): 335.09 [M + Na]^+^; HRMS (ESI, *m*/*z*): calcd. for C_18_H_16_O_5_Na: 335.0895 [M + Na]^+^, found: 335.0896.

#### Dimethyl 2-(4-(Chloromethyl)­benzoyl)-[1,1′-biphenyl]-3,4-dicarboxylate (**5n**)

1-(4-(Chloromethyl)­phenyl)-3-(methyl­(prop-2-yn-1-yl)­amino)-3-phenylprop-2-en-1-one (**3n**) (72.0 mg, 0.22 mmol), dimethyl acetylenedicarboxylate (**4a**) (41.0 mg, 0.29 mmol), AuCl (5.0 mg, 0.02 mmol), and AgSbF_6_ (11.0 mg, 0.03 mmol) were employed to afford 27.0 mg (29%) of the indicated product **5n** as a yellow solid (*R*_f_ = 0.29 in 4:1 hexane/ethyl acetate; mp 130.5–132.7 °C).

**5n:** ^1^H NMR (400 MHz, CDCl_3_) δ 8.05 (d, *J* = 8.0 Hz, 1H), 7.49 (d, *J* = 8.0 Hz, 1H), 7.46 (d, *J* = 8.0 Hz, 2H), 7.23 (d, *J* = 8.1 Hz, 2H), 7.10–7.04 (m, 5H), 4.46 (s, 2H), 3.88 (s, 3H), 3.51 (s, 3H); ^13^C NMR (100 MHz, CDCl_3_) δ 196.3 (C), 168.4 (C), 165.6 (C), 144.1 (C), 142.8 (C), 138.8 (C), 136.7 (C), 136.6 (C), 135.9 (C), 130.2 (CH), 129.7 (CH), 129.1 (CH), 128.5 (CH), 128.4 (CH), 128.3 (CH), 128.0 (CH), 53.0 (CH_3_), 52.5 (CH_3_), 45.3 (CH_2_) (Note that two C peaks overlap on each other); IR (neat): 3058, 3002, 2952, 2851, 1737, 1668, 1605, 1572, 1464, 1435, 1412, 1296, 1266, 1240, 1167, 1135, 1069, 1006, 966, 943, 842, 801, 766, 753, 676, 626, 554, 535, 492 cm^–1^; MS (ESI, *m*/*z*): 445.08 [M + Na]^+^; HRMS (ESI, *m*/*z*): calcd. for C_24_H_19_ClO_5_Na: 445.0819 [M + Na]^+^, found: 445.0819.

#### Dimethyl 2-(4-Ethylbenzoyl)-[1,1′-biphenyl]-3,4-dicarboxylate (**5o**)

1-(4-Ethylphenyl)-3-(methyl­(prop-2-yn-1-yl)­amino)-3-phenylprop-2-en-1-one (**3o**) (179.0 mg, 0.59 mmol), dimethyl acetylenedicarboxylate (**4a**) (110.0 mg, 0.77 mmol), AuCl (14.0 mg, 0.06 mmol), and AgSbF_6_ (31.0 mg, 0.09 mmol) were employed to afford 71.5 mg (30%) of the indicated product **5o** as a dark yellow solid (*R*_f_ = 0.28 in 4:1 hexane/ethyl acetate; mp 71.8–73.0 °C).

**5o:** ^1^H NMR (400 MHz, CDCl_3_) δ 8.03 (d, *J* = 8.0 Hz, 1H), 7.46 (d, *J* = 8.0 Hz, 1H), 7.42 (d, *J* = 8.2 Hz, 2H), 7.08 (s, 5H), 7.04 (d, *J* = 8.2 Hz, 2H), 3.86 (s, 3H), 3.50 (s, 3H), 2.55 (q, *J* = 7.6 Hz, 2H), 1.11 (t, *J* = 7.6 Hz, 3H); ^13^C NMR (100 MHz, CDCl_3_) δ 196.4 (C), 168.5 (C), 165.6 (C), 150.7 (C), 144.6 (C), 138.7 (C), 136.5 (CH), 136.0 (CH), 134.4 (CH), 130.1 (CH), 129.6 (CH), 128.7 (C), 128.9 (C), 128.2 (C), 128.1 (C), 127.9 (CH), 127.8 (CH), 52.9 (CH_3_), 52.4 (CH_3_), 29.0 (CH_3_), 15.1 (CH_2_); IR (neat): 3112, 2972, 2951, 2932, 1719, 1666, 1602, 1566, 1434, 1410, 1296, 1272, 1237, 1196, 1164, 1070, 1003, 966, 942, 840, 804, 763, 741, 699, 555, 504 cm^–1^; MS (ESI, *m*/*z*): 403.15 [M + H]^+^; HRMS (ESI, *m*/*z*): calcd. for C_25_H_23_O_5_: 403.1540 [M + H]^+^, found: 403.1533.

#### Dimethyl 2-(4-Methoxybenzoyl)-[1,1′-biphenyl]-3,4-dicarboxylate (**5p**)

1-(4-Methoxyphenyl)-3-(methyl­(prop-2-yn-1-yl)­amino)-3-phenylprop-2-en-1-one (**3p**) (80.0 mg, 0.26 mmol), dimethyl acetylenedicarboxylate (**4a**) (50.0 mg, 0.34 mmol), AuCl (6.0 mg, 0.03 mmol), and AgSbF_6_ (14.0 mg, 0.04 mmol) were employed to afford 39.1 mg (34%) of the indicated product **5p** as a yellow solid (*R*_f_ = 0.10 in 4:1 hexane/ethyl acetate; mp 129.3–131.0 °C).

**5p:** ^1^H NMR (400 MHz, CDCl_3_) δ 8.12 (d, *J* = 8.0 Hz, 1H), 7.58 (d, *J* = 8.8 Hz, 2H), 7.55 (d, *J* = 8.1 Hz, 1H), 7.18 (s, 5H), 6.79 (d, *J* = 8.8 Hz, 2H), 3.96 (s, 3H), 3.83 (s, 3H), 3.60 (s, 3H); ^13^C NMR (100 MHz, CDCl_3_) δ 195.3 (C), 168.6 (C), 165.7 (C), 163.9 (C), 144.7 (C), 138.5 (C), 136.6 (C), 136.0 (C), 132.3 (C), 129.7 (C), 129.6 (CH), 128.9 (CH), 128.6 (CH), 128.2 (CH), 128.1 (CH), 127.9 (CH), 113.7 (CH), 55.6 (CH_3_), 52.9 (CH_3_), 52.4 (CH_3_); IR (neat): 3079, 3009, 2956, 2838, 1720, 1660, 1597, 1510, 1432, 1297, 1240, 1165, 1142, 1068, 1022, 994, 943, 82, 754, 700, 678, 643, 558, 549 cm^–1^; MS (ESI, *m*/*z*): 405.13 [M + H]^+^; HRMS (ESI, *m*/*z*): calcd. for C_24_H_21_O_6_: 405.1333 [M + H]^+^, found: 405.1331.

#### Dimethyl 2-(3-Chlorobenzoyl)-[1,1′-biphenyl]-3,4-dicarboxylate (**5q**)

1-(3-Chlorophenyl)-3-(methyl­(prop-2-yn-1-yl)­amino)-3-phenylprop-2-en-1-one (**3q**) (364.0 mg, 1.17 mmol), dimethyl acetylenedicarboxylate (**4a**) (220.0 mg, 1.52 mmol), AuCl (27.0 mg, 0.12 mmol), and AgSbF_6_ (62.0 mg, 0.18 mmol) were employed to afford 158.1 mg (33%) of the indicated product **5q** as a yellow solid (*R*_f_ = 0.37 in 4:1 hexane/ethyl acetate; mp 127.5–128.3 °C).

**5q:** ^1^H NMR (400 MHz, CDCl_3_) δ 8.05 (d, *J* = 8.0 Hz, 1H), 7.51 (d, *J* = 8.0 Hz, 1H), 7.38 (s, 1H), 7.29 (d, *J* = 7.9 Hz, 2H), 7.12 (d, *J* = 7.8 Hz, 1H), 7.09–7.03 (m, 5H), 3.86 (s, 3H), 3.50 (s, 3H); ^13^C NMR (100 MHz, CDCl_3_) δ 195.6 (C), 168.2 (C), 165.4 (C), 143.5 (C), 138.6 (C), 138.2 (C), 136.5 (C), 135.7 (C), 134.5 (C), 133.2 (C), 129.6 (CH), 129.4 (CH), 129.2 (CH), 129.1 (CH), 128.5 (CH), 128.3 (CH), 127.9 (CH), 127.6 (CH), 52.9 (CH_3_), 52.4 (CH_3_) (Note that two CH peaks overlap on each other); IR (neat): 3212, 3154, 3075, 2958, 1730, 1678, 1588, 1566, 1466, 1437, 1371, 1307, 1293, 1275, 1235, 1173, 1135, 1068, 994, 965, 943, 887, 871, 812, 765, 742, 715, 700, 677, 557, 520, 489 cm^–1^; MS (ESI, *m*/*z*): 431.07 [M + Na]^+^; HRMS (ESI, *m*/*z*): calcd. for C_23_H_17_ClO_5_Na: 431.0662 [M + Na]^+^, found: 431.0662.

#### Dimethyl 2-(4-Fluorobenzoyl)-[1,1′-biphenyl]-3,4-dicarboxylate (**5r**)

1-(4-Fluorophenyl)-3-(methyl­(prop-2-yn-1-yl)­amino)-3-phenylprop-2-en-1-one (**3r**) (253.0 mg, 0.86 mmol), dimethyl acetylenedicarboxylate (**4a**) (160.0 mg, 1.12 mmol), AuCl (20.0 mg, 0.09 mmol), and AgSbF_6_ (45.0 mg, 0.13 mmol) were employed to afford 85.0 mg (25%) of the indicated product **5r** as an orange solid (*R*_f_ = 0.34 in 4:1 hexane/ethyl acetate; mp 94.5–95.8 °C).

**5r:** ^1^H NMR (400 MHz, CDCl_3_) δ 8.05 (d, *J* = 8.0 Hz, 1H), 7.50 (d, *J* = 8.1 Hz, 2H), 7.46 (t, *J* = 2.3 Hz, 1H), 7.10–7.06 (m, 4H), 7.04 (d, *J* = 3.5 Hz, 1H), 6.87 (t, *J* = 8.5 Hz, 2H), 3.87 (s, 3H), 3.51 (s, 3H); ^13^C NMR (100 MHz, CDCl_3_) δ 195.4 (C), 168.4 (C), 165.8 (d, ^1^ *J* = 256.1 Hz, C), 165.5 (C), 144.0 (C), 138.5 (C), 136.5 (C), 135.8 (C), 133.1 (d, ^4^ *J* = 2.6 Hz, C), 132.4 (d, ^3^ *J* = 9.5 Hz, CH), 129.6 (CH), 129.1 (CH), 129.0 (C), 128.3 (CH), 128.0 (CH), 115.6 (d, ^2^ *J* = 22.0 Hz, CH), 53.0 (CH_3_), 52.5 (CH_3_) (Note that two CH peaks overlap on each other); IR (neat): 3068, 3002, 2951, 2850, 1725, 1667, 1592, 1563, 1500, 1468, 1408, 1295, 1263, 1235, 1219, 1174, 1069, 992, 942, 858, 844, 814, 789, 756, 738, 636, 615, 580, 558 cm^–1^; MS (ESI, *m*/*z*): 415.10 [M + Na]^+^; HRMS (ESI, *m*/*z*): calcd. for C_23_H_17_FO_5_Na: 415.0958 [M + Na]^+^, found: 415.0958.

#### Diethyl 2-(4-Fluorobenzoyl)-[1,1′-biphenyl]-3,4-dicarboxylate (**5r′**)

1-(4-Fluorophenyl)-3-(methyl­(prop-2-yn-1-yl)­amino)-3-phenylprop-2-en-1-one (**3r**) (410.0 mg, 1.40 mmol), diethyl acetylenedicarboxylate (**4b**) (310.0 mg, 1.82 mmol), AuCl (33.0 mg, 0.14 mmol), and AgSbF_6_ (72.0 mg, 0.21 mmol) were employed to afford 149.3 mg (25%) of the indicated product **5r′** as a yellow solid (*R*_f_ = 0.51 in 4:1 hexane/ethyl acetate; mp 104.3–105.9 °C).

**5r′:** ^1^H NMR (400 MHz, CDCl_3_) δ 8.04 (d, *J* = 8.0 Hz, 1H), 7.47 (dt, *J* = 5.2, 2.9 Hz, 3H), 7.06 (s, 5H), 6.85 (t, *J* = 8.5 Hz, 2H), 4.31 (q, *J* = 7.1 Hz, 2H), 3.95 (q, *J* = 7.1 Hz, 2H), 1.30 (t, *J* = 7.1 Hz, 3H), 0.85 (t, *J* = 7.1 Hz, 3H); ^13^C NMR (100 MHz, CDCl_3_) δ 195.4 (C), 167.8 (C), 165.7 (d, ^1^ *J* = 256.0 Hz, C), 165.1 (C), 143.7 (C), 138.4 (C), 136.5 (C), 135.9 (C), 133.2 (d, ^4^ *J* = 2.7 Hz, C), 132.4 (CH), 132.3 (CH), 129.7 (CH), 129.5 (C), 129.1 (CH), 128.1 (d, ^3^ *J* = 12.5 Hz, CH), 127.8 (CH), 115.5 (d, ^2^ *J* = 22.1 Hz, CH), 61.9 (CH_2_), 61.5 (CH_2_), 14.1 (CH_3_), 13.6 (CH_3_); IR (neat): 3108, 3056, 2994, 2963, 2943, 1732, 1719, 1657, 1593, 1505, 1462, 1445, 1390, 1368, 1310, 1292, 1253, 1180, 1153, 1113, 1095, 1065, 1012, 974, 886, 851, 761, 738, 691, 641, 567, 527 cm^–1^; MS (ESI, *m*/*z*): 443.13 [M + Na]^+^; HRMS (ESI, *m*/*z*): calcd. for C_25_H_21_FO_5_Na: 443.1271 [M + Na]^+^, found: 443.1273.

#### Dimethyl 2-(4-Cyanobenzoyl)-[1,1′-biphenyl]-3,4-dicarboxylate (**5s**)

4-(3-(Methyl­(prop-2-yn-1-yl)­amino)-3-phenylacryloyl)­benzonitrile (**3s**) (92.8 mg, 0.32 mmol), dimethyl acetylenedicarboxylate (**4a**) (60.0 mg, 0.42 mmol), AuCl (7.0 mg, 0.03 mmol), and AgSbF_6_ (16.0 mg, 0.05 mmol) were employed to afford 23.0 mg (18%) of the indicated product **5s** as a yellowish-white solid (*R*_f_ = 0.23 in 4:1 hexane/ethyl acetate; mp 121.9–122.8 °C).

**5s:** ^1^H NMR (400 MHz, CDCl_3_) δ 8.18 (d, *J* = 8.0 Hz, 1H), 7.66 (d, *J* = 8.1 Hz, 1H), 7.54 (s, 4H), 7.16 (d, *J* = 2.0 Hz, 2H), 7.14 (t, *J* = 3.0 Hz, 1H), 7.10 (t, *J* = 2.6 Hz, 2H), 3.98 (s, 3H), 3.61 (s, 3H); ^13^C NMR (100 MHz, CDCl_3_) δ 196.0 (C), 168.2 (C), 165.4 (C), 143.1 (C), 140.1 (C), 138.7 (C), 136.6 (C), 135.7 (C), 132.1 (CH), 129.8 (CH), 129.6 (CH), 129.4 (CH), 128.7 (C), 128.6 (C), 128.2 (CH), 117.9 (C), 116.3 (CH), 53.1 (CH_3_), 52.6 (CH_3_) (Note that two CH peaks overlap on each other); IR (neat): 3090, 3010, 2954, 2852, 2232, 1720, 1674, 1602, 1587, 1564, 1466, 1434, 1406, 1293, 1271, 1235, 1196, 1165, 1069, 1018, 968, 941, 865, 841, 762, 743, 691, 665, 641, 544, 511, 450 cm^–1^; MS (ESI, *m*/*z*): 422.10 [M + Na]^+^; HRMS (ESI, *m*/*z*): calcd. for C_24_H_17_NO_5_Na: 422.1004 [M + Na]^+^, found: 422.1004.

#### Diethyl 2-(3-Nitrobenzoyl)-[1,1′-biphenyl]-3,4-dicarboxylate (**5t′**)

3-(Methyl­(prop-2-yn-1-yl)­amino)-1-(3-nitrophenyl)-3-phenylprop-2-en-1-one (**3t**) (141.0 mg, 0.44 mmol), diethyl acetylenedicarboxylate (**4b**) (97.0 mg, 0.57 mmol), AuCl (10.0 mg, 0.04 mmol), and AgSbF_6_ (23.0 mg, 0.07 mmol) were employed to afford 42.3 mg (21%) of the indicated product **5t′** as a yellow oil (*R*_f_ = 0.31 in 4:1 hexane/ethyl acetate).

**5t′:** ^1^H NMR (400 MHz, CDCl_3_) δ 8.19–8.14 (m, 2H), 8.12 (d, *J* = 8.0 Hz, 1H), 7.73 (d, *J* = 7.7 Hz, 1H), 7.59 (d, *J* = 8.0 Hz, 1H), 7.37 (t, *J* = 7.9 Hz, 1H), 7.04 (s, 5H), 4.35 (q, *J* = 7.1 Hz, 2H), 3.98 (q, *J* = 7.2 Hz, 2H), 1.34 (t, *J* = 7.1 Hz, 3H), 0.88 (t, *J* = 7.2 Hz, 3H); ^13^C NMR (100 MHz, CDCl_3_) δ 195.2 (C), 167.6 (C), 165.1 (C), 147.9 (C), 142.6 (C), 138.6 (C), 138.2 (C), 136.7 (C), 135.9 (C), 134.7 (CH), 130.4 (C), 129.9 (CH), 129.6 (CH), 129.5 (CH), 128.8 (CH), 128.5 (CH), 128.1 (CH), 127.3 (CH), 124.2 (CH), 62.2 (CH_2_), 61.7 (CH_2_), 14.3 (CH_3_), 13.7 (CH_3_); IR (neat): 3083, 2982, 2937, 2904, 2872, 1721, 1677, 1612, 1578, 1531, 1467, 1442, 1414, 1389, 1366, 1348, 1293, 1257, 1181, 1137, 1093, 1067, 909, 862, 802, 764, 740, 699, 670, 653, 570, 448 cm^–1^; MS (ESI, *m*/*z*): 470.12 [M + Na]^+^; HRMS (ESI, *m*/*z*): calcd. for C_25_H_21_NO_7_Na: 470.1216 [M + Na]^+^, found: 470.1216.

#### Dimethyl 4′-Bromo-2-(4-fluorobenzoyl)-[1,1′-biphenyl]-3,4-dicarboxylate (**5u**)

3-(4-Bromophenyl)-1-(4-fluorophenyl)-3-(methyl­(prop-2-yn-1-yl)­amino)­prop-2-en-1-one (**3u**) (140.0 mg, 0.38 mmol), dimethyl acetylenedicarboxylate (**4a**) (70.0 mg, 0.49 mmol), AuCl (9.0 mg, 0.04 mmol), and AgSbF_6_ (20.0 mg, 0.06 mmol) were employed to afford 44.3 mg (25%) of the indicated product **5u** as a yellow oil (*R*_f_ = 0.41 in 4:1 hexane/ethyl acetate).

**5u:** ^1^H NMR (400 MHz, CDCl_3_) δ 8.07 (d, *J* = 8.0 Hz, 1H), 7.54–7.50 (m, 2H), 7.48 (d, *J* = 8.1 Hz, 1H), 7.25 (d, *J* = 8.4 Hz, 2H), 6.97–6.89 (m, 4H), 3.88 (s, 3H), 3.56 (s, 3H); ^13^C NMR (100 MHz, CDCl_3_) δ 195.0 (C), 168.2 (C), 166.1 (d, ^1^ *J* = 256.9 Hz, C), 165.4 (C), 143.9 (C), 137.4 (C), 136.6 (C), 134.8 (C), 133.1 (d, ^4^ *J* = 2.8 Hz, C), 132.5 (d, ^3^ *J* = 9.6 Hz, CH), 131.3 (CH), 131.2 (CH), 129.4 (CH), 129.2 (C), 128.3 (CH), 122.9 (C), 115.9 (d, ^2^ *J* = 22.1 Hz, CH), 53.0 (CH_3_), 52.6 (CH_3_); IR (neat): 3002, 2952, 2845, 1715, 1668, 1594, 1568, 1504, 1487, 1463, 1410, 1277, 1259, 1238, 1198, 1150, 1061, 1011, 999, 966, 864, 844, 828, 801, 749, 719, 675, 616, 549, 517, 501, 468 cm^–1^; MS (ESI, *m*/*z*): 493.01 [M + Na]^+^; HRMS (ESI, *m*/*z*): calcd. for C_23_H_16_^79^BrFO_5_Na: 493.0063 [M + Na]^+^, found: 493.0057; MS (ESI, *m*/*z*): 495.00 [M + Na]^+^; HRMS (ESI, *m*/*z*): calcd. for C_23_H_16_^81^BrFO_5_Na: 495.0042 [M + Na]^+^, found: 495.0046.

### General Procedure for the Synthesis of 3-Alkenyl-1,2-dihydropyridines 6 under Copper Catalysis (Table 5)

To a stirred solution of the corresponding *N-*methyl*-N*-propargyl β-enaminone **3** (1.00 mmol) in acetonitrile (6 mL) under argon were added dialkyl acetylenedicarboxylate **4** (2.0 mmol) and CuBr (0.10 mmol), and the resulting mixture was refluxed for approximately 5 h in an oil bath (The progress of the reaction was monitored by routine TLC analysis for the disappearance of β-enaminone **3** using hexane/ethyl acetate (4:1) as the eluent). After the reaction was over, the solvent was removed on a rotary evaporator, and ethyl acetate (30 mL) and a saturated aqueous solution of NaCl (15 mL) were added. After the layers were separated, the aqueous layer was extracted with ethyl acetate (2 × 30 mL) again. The combined organic layers were dried over MgSO_4_ and evaporated on a rotary evaporator to give the crude product, which was purified by flash chromatography on silica gel using hexane/ethyl acetate (5:1, followed by 2:1) as the eluent to afford the corresponding 3-alkenyl-1,2-dihydropyridine **6** and 1,2,3,4-tetrasubstituted benzene **5**.

#### Dimethyl 2-(5-Benzoyl-1-methyl-6-phenyl-1,2-dihydropyridin-3-yl)­but-2-enedioate (6a) and Dimethyl 2-Benzoyl-[1,1′-biphenyl]-3,4-dicarboxylate (**5a**)

3-(Methyl­(prop-2-yn-1-yl)­amino)-1,3-diphenylprop-2-en-1-one (**3a**) (135.6 mg, 0.49 mmol), dimethyl acetylenedicarboxylate (**4a**) (140.0 mg, 0.98 mmol), and CuBr (7.0 mg, 0.05 mmol) were employed. Two fractions were isolated. The first fraction afforded 16.1 mg (9%) of the indicated product **5a** as a yellowish-orange oil (*R*_f_ = 0.33 in 4:1 hexane/ethyl acetate). The second fraction yielded 80.3 mg (39%) of the indicated product **6a** as an orange oil (*R*_f_ = 0.03 in 4:1 hexane/ethyl acetate).

**6a:** ^1^H NMR (400 MHz) δ 7.40 (d, *J* = 7.0 Hz, 2H), 7.25–7.21 (m, 4H), 7.17 (d, *J* = 7.6 Hz, 2H), 7.12 (dd, *J* = 6.5, 3.1 Hz, 2H), 6.96 (s, 1H), 5.41 (s, 1H), 4.37 (s, 2H), 3.87 (s, 3H), 3.76 (s, 3H), 2.88 (s, 3H).^13^C NMR (100 MHz, CDCl_3_) δ 193.1 (C), 168.3 (C), 166.2 (C), 160.9 (C), 147.6 (CH), 140.6 (C), 134.2 (C), 134.1 (CH), 130.6 (C), 129.6 (C), 129.1 (CH), 128.9 (CH), 128.6 (CH), 127.7 (CH), 111.7 (C), 110.9 (CH), 108.9 (CH), 52.8­(CH_3_), 52.3 (CH_2_), 51.9­(CH_3_), 41.6 (CH_3_); IR (neat): 3058, 3027, 2952, 2927, 1733, 1670, 1655, 1648, 1595, 1578, 1525, 1490, 1445, 1434, 1342, 1205, 1169, 1068, 1015, 956, 768, 728, 695, 657, 633, 613, 533, 491 cm^–1^; MS (ESI, *m*/*z*): 418.17 [M + H]^+^; HRMS (ESI, *m*/*z*): calcd. for C_25_H_24_NO_5_: 418.1654 [M + H]^+^, found: 418.1656.

#### Dimethyl 2-(5-Benzoyl-1-methyl-6-(4-(methylthio)­phenyl)-1,2-dihydropyridin-3-yl)­but-2-enedioate (6f) and Dimethyl 2-Benzoyl-4′-(methylthio)-[1,1′-biphenyl]-3,4-dicarboxylate (**5f**)

3-(Methyl­(prop-2-yn-1-yl)­amino)-3-(4-(methylthio)­phenyl)-1-phenylprop-2-en-1-one (**3f**) (138.0 mg, 0.43 mmol), dimethyl acetylenedicarboxylate (**4a**) (120.0 mg, 0.86 mmol), and CuBr (6.0 mg, 0.04 mmol) were employed. Two fractions were isolated. The first fraction afforded 8.3 mg (5%) of the indicated product **5f** as a yellow solid (*R*_f_ = 0.21 in 4:1 hexane/ethyl acetate; mp 97.3–98.6 °C). The second fraction yielded 29.3 mg (15%) of the indicated product **6f** as a dark red oil (*R*_f_ = 0.06 in 4:1 hexane/ethyl acetate).

**6f:** ^1^H NMR (400 MHz) δ 7.36 (d, *J* = 8.2 Hz, 2H), 7.29 (d, *J* = 0.5 Hz, 2H), 7.26 (bs, 1H), 7.14–7.12 (m, 2H), 7.02 (d, *J* = 8.2 Hz, 2H), 6.95 (s, 1H), 5.42 (s, 1H), 4.37 (s, 2H), 3.90 (s, 3H), 3.77 (s, 3H), 2.89 (s, 3H), 2.45 (s, 3H).^13^C NMR (100 MHz, CDCl_3_) δ 192.3 (C), 168.3 (C), 166.2 (C), 160.5 (C), 147.6 (C), 142.5 (C), 137.0 (C), 134.2 (C), 134.1 (C), 129.7 (C), 129.6 (CH), 129.2 (CH), 128.7 (CH), 125.0 (CH), 111.9 (CH), 110.9 (CH), 109.0 (CH), 52.9 (CH_3_), 52.4 (CH_3_), 51.9 (CH_3_), 41.6 (CH_2_), 15.4 (CH_3_); IR (neat): 3056, 2995, 2949, 2924, 1734, 1670, 1648, 1585, 1510, 1433, 1398, 1364, 1326, 1245, 1209, 1150, 1090, 1014, 996, 834, 762, 735, 700, 633, 652, 516, 473 cm^–1^; MS (ESI, *m*/*z*): 464.15 [M + H]^+^; HRMS (ESI, *m*/*z*): calcd. for C_26_H_26_NO_5_S: 464.1532 [M + H]^+^, found: 464.1532.

#### Dimethyl 2-(5-Benzoyl-6-(4-bromophenyl)-1-methyl-1,2-dihydropyridin-3-yl)­but-2-enedioate (6g) and Dimethyl 2-Benzoyl-4′-bromo-[1,1′-biphenyl]-3,4-dicarboxylate (**5g**)

3-(4-Bromophenyl)-3-(methyl­(prop-2-yn-1-yl)­amino)-1-phenylprop-2-en-1-one (**3g**) (217.0 mg, 0.61 mmol), dimethyl acetylenedicarboxylate (**4a**) (170.0 mg, 1.22 mmol), and CuBr (9.0 mg, 0.06 mmol) were employed. Two fractions were isolated. The first fraction afforded 20.3 mg (7%) of the indicated product **5g** as a brown solid (*R*_f_ = 0.29 in 4:1 hexane/ethyl acetate; mp 119.1–120.3 °C). The second fraction yielded 71.5 mg (24%) of the indicated product **6g** as a dark orange solid (*R*_f_ = 0.14 in 4:1 hexane/ethyl acetate; mp 79.5–80.3 °C).

**6g:** ^1^H NMR (400 MHz, CDCl_3_) δ 7.39 (d, *J* = 4.6 Hz, 2H), 7.37 (d, *J* = 6.2 Hz, 2H), 7.31 (t, *J* = 6.8 Hz, 1H), 7.21 (t, *J* = 7.5 Hz, 2H), 7.00 (d, *J* = 8.4 Hz, 2H), 6.87 (s, 1H), 5.39 (s, 1H), 4.35 (s, 2H), 3.83 (s, 3H), 3.74 (s, 3H), 2.86 (s, 3H); ^13^C NMR (100 MHz, CDCl_3_) δ 192.8 (C), 168.2 (C), 166.1 (C), 159.7 (C), 147.3 (C), 140.3 (C), 133.8 (C), 133.0 (C), 131.9 (CH), 130.8 (C), 130.4 (CH), 128.8 (CH), 127.8 (CH), 124.0 (C), 111.2 (CH), 111.1 (CH), 109.2 (CH), 52.7 (CH_3_), 52.3 (CH_2_), 51.9 (CH_3_), 41.5 (CH_3_); IR (neat): 3050, 3023, 2997, 2948, 1734, 1709, 1638, 1561, 1518, 1459, 1433, 1395, 1363, 1329, 1254, 1195, 1149, 1068, 1027, 1007, 824, 796, 734, 698, 661, 645, 619, 548, 517 cm^–1^; MS (ESI, *m*/*z*): 496.07 [M + H]^+^; HRMS (ESI, *m*/*z*): calcd. for C_25_H_23_BrNO_5_: 496.0760 [M + H]^+^, found: 496.0718.

#### Dimethyl 2-(5-Benzoyl-6-(4-chlorophenyl)-1-methyl-1,2-dihydropyridin-3-yl)­but-2-enedioate (6h) and Dimethyl 2-Benzoyl-4′-chloro-[1,1′-biphenyl]-3,4-dicarboxylate (**5h**)

3-(4-Chlorophenyl)-3-(methyl­(prop-2-yn-1-yl)­amino)-1-phenylprop-2-en-1-one (**3h**) (172.1 mg, 0.56 mmol), dimethyl acetylenedicarboxylate (**4a**) (160.0 mg, 1.12 mmol), and CuBr (8.0 mg, 0.06 mmol) were employed. Two fractions were isolated. The first fraction afforded 16.7 mg (7%) of the indicated product **5b** as a yellowish-orange oil (*R*_f_ = 0.37 in 4:1 hexane/ethyl acetate). The second fraction yielded 113.8 mg (45%) of the indicated product **6h** as a dark red oil (*R*_f_ = 0.10 in 4:1 hexane/ethyl acetate).

**6h:** ^1^H NMR (400 MHz, CDCl_3_) δ 7.37 (d, *J* = 7.2 Hz, 2H), 7.28 (t, *J* = 7.4 Hz, 1H), 7.20–7.16 (m, 4H), 7.04 (d, *J* = 8.4 Hz, 2H), 6.88 (s, 1H), 5.39 (s, 1H), 4.33 (s, 2H), 3.83 (s, 3H), 3.73 (s, 3H), 2.84 (s, 3H); ^13^C NMR (100 MHz, CDCl_3_) δ 192.7 (C), 168.1 (C), 166.0 (C), 159.6 (C), 147.2 (C), 140.3 (C), 135.6 (C), 133.6 (C), 132.5 (CH), 130.7 (CH), 130.3 (CH), 128.8 (CH), 128.7 (CH), 127.7 (CH), 111.2 (C), 111.1 (C), 109.2 (CH), 52.6 (CH_3_), 52.2 (CH_2_), 51.8 (CH_3_), 41.4 (CH_3_); IR (neat): 3064, 2998, 2950, 1733, 1673, 1647, 1594, 1578, 1527, 1486, 1434, 1398, 1371, 1238, 1204, 1089, 1069, 1011, 893, 833, 787, 731, 698, 660, 618, 536, 500 cm^–1^; MS (ESI, *m*/*z*): 452.12 [M + H]^+^; HRMS (ESI, *m*/*z*): calcd. for C_25_H_23_ClNO_5_: 452.1259 [M + H]^+^, found: 452.1246.

#### Dimethyl 2-(5-Benzoyl-1-methyl-6-(4-nitrophenyl)-1,2-dihydropyridin-3-yl)­but-2-enedioate (6l) and Dimethyl 2-Benzoyl-4′-nitro-[1,1′-biphenyl]-3,4-dicarboxylate (**5l**)

3-(Methyl­(prop-2-yn-1-yl)­amino)-3-(4-nitrophenyl)-1-phenylprop-2-en-1-one (**3l**) (272.0 mg, 0.85 mmol), dimethyl acetylenedicarboxylate (**4a**) (240.0 mg, 1.70 mmol), and CuBr (12.0 mg, 0.09 mmol) were employed. Two fractions were isolated. The first fraction afforded 13.6 mg (4%) of the indicated product **5l** as an orange solid (*R*_f_ = 0.18 in 4:1 hexane/ethyl acetate; mp 141.1–142.8 °C). The second fraction yielded 102.6 mg (26%) of the indicated product **6l** as a dark red oil (*R*_f_ = 0.03 in 4:1 hexane/ethyl acetate).

**6l:** ^1^H NMR (400 MHz, CDCl_3_) δ 8.20 (d, *J* = 8.4 Hz, 2H), 7.45 (d, *J* = 7.9 Hz, 2H), 7.39 (d, *J* = 8.6 Hz, 2H), 7.30–7.26 (m, 3H), 6.80 (s, 1H), 5.41 (s, 1H), 4.42 (s, 2H), 3.81 (s, 3H), 3.77 (s, 3H), 2.85 (s, 3H); ^13^C NMR (100 MHz, CDCl_3_) δ 192.2 (C), 168.0 (C), 165.9 (C), 158.4 (C), 148.2 (C), 147.1 (C), 141.3 (C), 139.7 (C), 133.3 (C), 131.4 (CH), 129.7 (CH), 128.9 (CH), 128.1 (CH), 124.1 (CH), 111.8 (CH), 110.5 (C), 110.1 (CH), 52.8 (CH_3_), 52.7 (CH_2_), 52.0 (CH_3_), 41.4 (CH_3_); IR (neat): 3073, 3039, 2996, 2861, 1729, 1672, 1594, 1578, 1561, 1459, 1436, 1344, 1248, 1198, 1154, 1068, 1026, 1002, 944, 852, 797, 754, 734, 698, 663, 649, 544, 509 cm^–1^; MS (ESI, *m*/*z*): 463.15 [M + H]^+^; HRMS (ESI, *m*/*z*): calcd. for C_25_H_23_N_2_O_7_: 463.1505 [M + H]^+^, found: 463.1505.

#### Dimethyl 2-(5-(4-Methoxybenzoyl)-1-methyl-6-phenyl-1,2-dihydropyridin-3-yl)­but-2-enedioate (6p) and Dimethyl 2-(4-Methoxybenzoyl)-[1,1′-biphenyl]-3,4-dicarboxylate (**5p**)

1-(4-Methoxyphenyl)-3-(methyl­(prop-2-yn-1-yl)­amino)-3-phenylprop-2-en-1-one (**3p**) (74.0 mg, 0.24 mmol), dimethyl acetylenedicarboxylate (**4a**) (70.0 mg, 0.48 mmol), and CuBr (3.0 mg, 0.02 mmol) were employed. Two fractions were isolated. The first fraction afforded 14.5 mg (15%) of the indicated product **5p** as a yellow solid (*R*_f_ = 0.10 in 4:1 hexane/ethyl acetate; mp 129.3–131.0 °C). The second fraction yielded 18.7 mg (17%) of the indicated product **6p** as a dark red oil (*R*_f_ = 0.03 in 4:1 hexane/ethyl acetate).

**6p:** ^1^H NMR (400 MHz, CDCl_3_) δ 7.46 (d, *J* = 8.8 Hz, 2H), 7.28 (bs, 1H), 7.26 (d, *J* = 2.3 Hz, 2H), 7.14 (dd, *J* = 6.5, 3.2 Hz, 2H), 6.93 (s, 1H), 6.69 (d, *J* = 8.8 Hz, 2H), 5.40 (s, 1H), 4.37 (s, 2H), 3.89 (s, 3H), 3.78 (s, 3H), 3.76 (s, 3H), 2.88 (s, 3H); ^13^C NMR (100 MHz, CDCl_3_) δ 192.2 (C), 171.3 (C), 168.4 (C), 166.2 (C), 161.9 (C), 160.1 (C), 147.6 (C), 134.4 (C), 134.2 (C), 132.9 (C), 131.2 (CH), 129.6 (CH), 129.1 (CH), 128.6 (CH), 113.0 (CH), 110.6 (CH), 108.7 (CH), 55.4 (OCH_3_), 52.8 (CH_3_), 52.3 (CH_2_), 51.9 (CH_3_), 41.6 (CH_3_); IR (neat): 3093, 2952, 2927, 2852, 1726, 1668, 1593, 1508, 1435, 1372, 1296, 1239, 1200, 1151, 1069, 1026, 1003, 943, 844, 789, 762, 735, 703, 637, 608, 509 cm^–1^; MS (ESI, *m*/*z*): 448.18 [M + H]^+^; HRMS (ESI, *m*/*z*): calcd. for C_26_H_26_NO_6_: 448.1760 [M + H]^+^, found: 448.1760.

#### Dimethyl 2-(5-(3-Chlorobenzoyl)-1-methyl-6-phenyl-1,2-dihydropyridin-3-yl)­but-2-enedioate (6q) and Dimethyl 2-(3-Chlorobenzoyl)-[1,1′-biphenyl]-3,4-dicarboxylate (**5q**)

1-(3-Chlorophenyl)-3-(methyl­(prop-2-yn-1-yl)­amino)-3-phenylprop-2-en-1-one (**3q**) (218.0 mg, 0.70 mmol), dimethyl acetylenedicarboxylate (**4a**) (200.0 mg, 1.40 mmol), and CuBr (10.0 mg, 0.07 mmol) were employed. Two fractions were isolated. The first fraction afforded 16.2 mg (6%) of the indicated product **5q** as a yellow solid (*R*_f_ = 0.37 in 4:1 hexane/ethyl acetate; mp 127.5–128.3 °C). The second fraction yielded 193.4 mg (61%) of the indicated product **6q** as a brown solid (*R*_f_ = 0.11 in 4:1 hexane/ethyl acetate; mp 126.4–127.0 °C).

**6q:** ^1^H NMR (400 MHz, CDCl_3_) δ 7.24 (t, *J* = 1.7 Hz, 1H), 7.21 (t, *J* = 1.3 Hz, 1H), 7.19 (d, *J* = 2.6 Hz, 2H), 7.13 (d, *J* = 1.2 Hz, 1H), 7.11 (d, *J* = 1.2 Hz, 1H), 7.06 (d, *J* = 2.2 Hz, 1H), 7.05–7.02 (m, 2H), 6.98 (s, 1H), 5.42 (s, 1H), 4.34 (s, 2H), 3.89 (s, 3H), 3.73 (s, 3H), 2.86 (s, 3H); ^13^C NMR (100 MHz, CDCl_3_) δ 191.2 (C), 168.2 (C), 166.0 (C), 161.2 (C), 147.3 (C), 142.4 (C), 133.6 (C), 133.4 (C), 133.2 (C), 130.1 (CH), 129.8 (CH), 129.1 (CH), 128.9 (CH), 128.8 (CH), 128.5 (CH), 126.6 (CH), 111.4 (CH), 111.2 (C), 109.3 (CH), 52.7 (CH_3_), 52.1 (CH_2_), 51.8 (CH_3_), 41.7 (CH_3_); IR (neat): 3053, 2997, 2949, 1728, 1705, 1597, 1567, 1557, 1523, 1499, 1460, 1432, 1412, 1394, 1370, 1342, 1250, 1195, 1173, 1151, 1075, 1035, 999, 982, 868, 805, 771, 729, 706, 654, 626, 551 cm^–1^; MS (ESI, *m*/*z*): 452.13 [M + H]^+^; HRMS (ESI, *m*/*z*): calcd. for C_25_H_23_ClNO_5_: 452.1265 [M + H]^+^, found: 452.1264.

#### Dimethyl 2-(5-(4-Fluorobenzoyl)-1-methyl-6-phenyl-1,2-dihydropyridin-3-yl)­but-2-enedioate (6r) and Dimethyl 2-(4-Fluorobenzoyl)-[1,1′-biphenyl]-3,4-dicarboxylate (**5r**)

1-(4-Fluorophenyl)-3-(methyl­(prop-2-yn-1-yl)­amino)-3-phenylprop-2-en-1-one (**3r**) (259.0 mg, 0.88 mmol), dimethyl acetylenedicarboxylate (**4a**) (250.0 mg, 1.76 mmol), and CuBr (13.0 mg, 0.09 mmol) were employed. Two fractions were isolated. The first fraction afforded 18.1 mg (5%) of the indicated product **5r** as an orange solid (*R*_f_ = 0.34 in 4:1 hexane/ethyl acetate; mp 94.5–95.8 °C). The second fraction yielded 85.7 mg (22%) of the indicated product **6r** as a dark red solid (*R*_f_ = 0.09 in 4:1 hexane/ethyl acetate; mp 160.8–161.7 °C).

**6r:** ^1^H NMR (400 MHz, CDCl_3_) δ 7.34–7.29 (m, 2H), 7.14 (d, *J* = 6.9 Hz, 3H), 7.03–6.99 (m, 2H), 6.88 (s, 1H), 6.73 (t, *J* = 8.6 Hz, 2H), 5.35 (s, 1H), 4.28 (s, 2H), 3.82 (s, 3H), 3.67 (s, 3H), 2.81 (s, 3H); ^13^C NMR (100 MHz, CDCl_3_) δ 191.8 (C), 168.3 (C), 166.1 (C), 164.0 (d, ^1^ *J* = 251.0 Hz, C), 160.7 (C), 147.4 (C), 136.8 (d, ^4^ *J* = 3.0 Hz, C), 133.8 (d, ^3^ *J* = 12.9 Hz, CH), 131.2 (C), 131.1 (C), 129.8 (C), 129.2 (CH), 128.6 (CH), 114.5 (d, ^2^ *J* = 21.8 Hz, CH), 111.6 (CH), 111.1 (CH), 109.1 (CH), 52.8 (CH_3_), 52.2 (CH_2_), 51.9 (CH_3_), 41.7 (CH_3_); IR (neat): 3065, 2998, 2952, 2868, 1731, 1709, 1592, 1566, 1502, 1436, 1407, 1389, 1356, 1323, 1292, 1267, 1221, 1196, 1167, 1073, 1014, 934, 900, 848, 785, 765, 717, 701, 634, 602, 518 cm^–1^; MS (ESI, *m*/*z*): 436.16 [M + H]^+^; HRMS (ESI, *m*/*z*): calcd. for C_25_H_23_FNO_5_: 436.1560 [M + H]^+^, found: 436.1559.

#### Dimethyl 2-(5-(4-Cyanobenzoyl)-1-methyl-6-phenyl-1,2-dihydropyridin-3-yl)­but-2-enedioate (6s) and Dimethyl 2-(4-Cyanobenzoyl)-[1,1′-biphenyl]-3,4-dicarboxylate (**5s**)

4-(3-(Methyl­(prop-2-yn-1-yl)­amino)-3-phenylacryloyl)­benzonitrile (**3s**) (88.0 mg, 0.29 mmol), dimethyl acetylenedicarboxylate (**4a**) (82.0 mg, 0.58 mmol), and CuBr (4.0 mg, 0.03 mmol) were employed. Two fractions were isolated. The first fraction afforded 6.0 mg (5%) of the indicated product **5s** as a yellowish-white solid (*R*_f_ = 0.23 in 4:1 hexane/ethyl acetate; mp 121.9–122.8 °C). The second fraction yielded 26.0 mg (20%) of the indicated product **6s** as an orange oil (*R*_f_ = 0.03 in 4:1 hexane/ethyl acetate).

**6s:** ^1^H NMR (400 MHz, CDCl_3_) δ 7.31–7.24 (m, 4H), 7.15 (t, *J* = 3.0 Hz, 1H), 7.11 (d, *J* = 7.7 Hz, 2H), 6.98–6.94 (m, 3H), 5.39 (s, 1H), 4.29 (s, 2H), 3.86 (s, 3H), 3.69 (s, 3H), 2.82 (s, 3H); ^13^C NMR (100 MHz, CDCl_3_) δ 191.2 (C), 168.2 (C), 166.0 (C), 161.4 (C), 147.3 (C), 145.2 (C), 133.5 (C), 132.9 (CH), 131.4 (CH), 130.3 (C), 129.5 (CH), 129.0 (CH), 128.8 (CH), 118.5 (C), 113.2 (C), 112.0 (CH), 111.6 (C), 110.1 (CH), 53.0 (CH_3_), 52.3 (CH_2_), 52.0 (CH_3_), 41.9 (CH_3_); IR (neat): 3088, 2998, 2951, 2225, 1736, 1706, 1621, 1568, 1519, 1466, 1434, 1399, 1362, 1332, 1242, 1196, 1168, 1148, 1071, 1016, 983, 914, 844, 778, 757, 722, 702, 643, 582, 541, 508 cm^–1^; MS (ESI, *m*/*z*): 443.16 [M + H]^+^; HRMS (ESI, *m*/*z*): calcd. for C_26_H_23_N_2_O_5_: 443.1607 [M + H]^+^, found: 443.1606.

#### Diethyl 2-(1-Methyl-5-(3-nitrobenzoyl)-6-phenyl-1,2-dihydropyridin-3-yl)­but-2-enedioate (**6t′**) and Diethyl 2-(3-Nitrobenzoyl)-[1,1′-biphenyl]-3,4-dicarboxylate (**5t′**)

3-(Methyl­(prop-2-yn-1-yl)­amino)-1-(3-nitrophenyl)-3-phenylprop-2-en-1-one (**3t**) (133.0 mg, 0.42 mmol), diethyl acetylenedicarboxylate (**4b**) (140.0 mg, 0.84 mmol), and CuBr (6.0 mg, 0.04 mmol) were employed. Two fractions were isolated. The first fraction afforded 24.6 mg (13%) of the indicated product **5t′** as a yellowish oil (*R*_f_ = 0.31 in 4:1 hexane/ethyl acetate). The second fraction yielded 82.3 mg (40%) of the indicated product **6t′** as a brown solid (*R*_f_ = 0.09 in 4:1 hexane/ethyl acetate; mp 167.4–168.2 °C).

**6t′:** ^1^H NMR (400 MHz, CDCl_3_) δ 8.07 (t, *J* = 1.6 Hz, 1H), 7.99 (d, *J* = 8.0 Hz, 1H), 7.67 (d, *J* = 7.7 Hz, 1H), 7.31–7.25 (m, 2H), 7.17–7.13 (m, 4H), 7.10 (t, *J* = 2.7 Hz, 1H), 5.49 (s, 1H), 4.47–4.41 (m, 4H), 4.23 (q, *J* = 7.1 Hz, 2H), 2.93 (s, 3H), 1.40 (t, *J* = 7.1 Hz, 3H), 1.32 (t, *J* = 7.1 Hz, 3H); ^13^C NMR (100 MHz, CDCl_3_) δ 190.1 (C), 167.6 (C), 165.4 (C), 161.1 (C), 147.0 (C), 146.9 (C), 142.5 (C), 134.2 (C), 133.5 (C), 132.4 (CH), 130.2 (CH), 129.6 (CH), 128.7 (C), 128.6 (CH), 124.4 (CH), 123.6 (CH), 112.5 (CH), 111.4 (CH), 110.6 (CH), 62.0 (CH_2_), 60.7 (CH_2_), 52.2 (CH_2_), 41.9 (CH_3_), 14.3 (CH_3_), 14.1 (CH_3_); IR (neat): 3075, 3049, 2982, 2937, 2902, 1743, 1705, 1600, 1582, 1566, 1525, 1456, 1439, 1404, 1383, 1368, 1348, 1305, 1257, 1181, 1156, 1097, 1023, 936, 870, 846, 836, 772, 715, 696, 651, 622, 552 cm^–1^; MS (ESI, *m*/*z*): 491.18 [M + H]^+^; HRMS (ESI, *m*/*z*): calcd. for C_27_H_27_N_2_O_7_: 491.1818 [M + H]^+^, found: 491.1818.

#### Dimethyl 2-(6-(4-Bromophenyl)-5-(4-fluorobenzoyl)-1-methyl-1,2-dihydropyridin-3-yl)­but-2-enedioate (6u) and Dimethyl 4′-Bromo-2-(4-fluorobenzoyl)-[1,1′-biphenyl]-3,4-dicarboxylate (**5u**)

3-(4-Bromophenyl)-1-(4-fluorophenyl)-3-(methyl­(prop-2-yn-1-yl)­amino)­prop-2-en-1-one (**3u**) (130.0 mg, 0.35 mmol), dimethyl acetylenedicarboxylate (**4a**) (100.0 mg, 0.70 mmol), and CuBr (5.0 mg, 0.04 mmol) were employed. Two fractions were isolated. The first fraction afforded 4.8 mg (3%) of the indicated product **5u** as a yellow oil (*R*_f_ = 0.41 in 4:1 hexane/ethyl acetate). The second fraction yielded 72.7 mg (40%) of the indicated product **6u** as a dark red solid (*R*_f_ = 0.08 in 4:1 hexane/ethyl acetate; mp 162.4–163.7 °C).

**6u:** ^1^H NMR (400 MHz, CDCl_3_) δ 7.44 (dd, *J* = 6.1, 2.7 Hz, 2H), 7.42 (dd, *J* = 6.3, 2.0 Hz, 2H), 7.01 (d, *J* = 8.4 Hz, 2H), 6.90 (t, *J* = 8.6 Hz, 2H), 6.86 (s, 1H), 5.42 (s, 1H), 4.36 (s, 2H), 3.87 (s, 3H), 3.76 (s, 3H), 2.88 (s, 3H); ^13^C NMR (100 MHz, CDCl_3_) δ 191.4 (C), 168.2 (C), 166.0 (C), 164.3 (d, ^1^ *J* = 252.1 Hz, C), 159.5 (C), 147.2 (C), 136.5 (d, ^4^ *J* = 2.3 Hz, C), 133.4 (CH), 133.0 (C), 132.0 (CH), 131.2 (d, ^3^ *J* = 8.8 Hz, CH), 130.5 (CH), 124.2 (C), 114.8 (d, ^2^ *J* = 21.5 Hz, CH), 111.4 (C), 111.2 (C), 109.6 (CH), 52.8 (CH_3_), 52.3 (CH_3_), 51.9 (CH_3_), 41.5 (CH_2_); IR (neat): 3059, 3023, 2995, 2944, 1734, 1693, 1614, 1588, 1566, 1506, 1455, 1433, 1393, 1362, 1297, 1266, 1193, 1150, 1105, 1065, 1034, 935, 901, 851, 833, 783, 766, 736, 677, 636, 605, 565, 522, 501 cm^–1^; MS (ESI, *m*/*z*): 514.07 [M + H]^+^; HRMS (ESI, *m*/*z*): calcd. for C_25_H_22_^79^BrFNO_5_: 514.0665 [M + H]^+^, found: 514.0666; MS (ESI, *m*/*z*): 516.07 [M + H]^+^; HRMS (ESI, *m*/*z*): calcd. for C_25_H_22_^81^BrFNO_5_: 516.0645 [M + H]^+^, found: 516.0671.

#### Diethyl 2-(6-(4-Bromophenyl)-5-(4-fluorobenzoyl)-1-methyl-1,2-dihydropyridin-3-yl)­but-2-enedioate (**6u′**) and Diethyl 4′-Bromo-2-(4-fluorobenzoyl)-[1,1′-biphenyl]-3,4-dicarboxylate (**5u′**)

3-(4-Bromophenyl)-1-(4-fluorophenyl)-3-(methyl­(prop-2-yn-1-yl)­amino)­prop-2-en-1-one (**3u**) (133.0 mg, 0.36 mmol), diethyl acetylenedicarboxylate (**4b**) (120.0 mg, 0.72 mmol), and CuBr (5.0 mg, 0.04 mmol) were employed. Two fractions were isolated. The first fraction afforded 18.3 mg (10%) of the indicated product **5u′** as a light yellow oil (*R*_f_ = 0.42 in 4:1 hexane/ethyl acetate). The second fraction yielded 104.1 mg (53%) of the indicated product **6u′** as a dark red oil (*R*_f_ = 0.14 in 4:1 hexane/ethyl acetate).

**5u′:** ^1^H NMR (400 MHz, CDCl_3_) δ 8.07 (d, *J* = 8.0 Hz, 1H), 7.54–7.49 (m, 2H), 7.47 (d, *J* = 8.0 Hz, 1H), 7.25 (d, *J* = 8.4 Hz, 2H), 6.97–6.90 (m, 4H), 4.34 (q, *J* = 7.1 Hz, 2H), 4.00 (q, *J* = 7.1 Hz, 2H), 1.33 (t, *J* = 7.1 Hz, 3H), 0.95 (t, *J* = 7.2 Hz, 3H); ^13^C NMR (100 MHz, CDCl_3_) δ 195.0 (C), 167.7 (C), 166.1 (d, ^1^ *J* = 256.8 Hz C), 165.1 (C), 143.7 (C), 137.3 (C), 136.7 (C), 134.9 (C), 133.2 (d, ^4^ *J* = 2.6 Hz, C), 132.5 (d, ^3^ *J* = 9.5 Hz, CH), 131.5 (C), 131.1 (C), 129.7 (CH), 129.4 (CH), 128.1 (CH), 122.8 (CH), 115.8 (d, ^2^ *J* = 22.1 Hz, CH), 62.1 (CH_2_), 61.8 (CH_2_), 14.3 (CH_3_), 13.8 (CH_3_); IR (neat): 3074, 2981, 2935, 2905, 1721, 1670, 1595, 1504, 1491, 1466, 1410, 1391, 1367, 1294, 1258, 1235, 1180, 1152, 1133, 1097, 1059, 1009, 975, 850, 829, 750, 716, 692, 669, 630, 615, 555, 507 cm^–1^; MS (ESI, *m*/*z*): 521.04 [M + Na]^+^; HRMS (ESI, *m*/*z*): calcd. for C_25_H_20_^79^BrFO_5_Na: 521.0370 [M + Na]^+^, found: 521.0373; MS (ESI, *m*/*z*): 523.04 [M + Na]^+^; HRMS (ESI, *m*/*z*): calcd. for C_25_H_20_^81^BrFO_5_Na: 523.0355 [M + Na]^+^, found: 523.0359.

**6u′:** ^1^H NMR (400 MHz, CDCl_3_) δ 7.35–7.28 (m, 4H), 6.93–6.89 (m, 2H), 6.81–6.78 (m, 1H), 6.78–6.73 (m, 2H), 5.29 (d, *J* = 4.5 Hz, 1H), 4.25 (s, 2H), 4.22 (dd, *J* = 7.1, 4.6 Hz, 2H), 4.09 (dd, *J* = 7.1, 4.6 Hz, 2H), 2.76 (s, 3H), 1.22–1.13 (m, 6H); ^13^C NMR (100 MHz, CDCl_3_) δ 191.4 (C), 167.6 (C), 165.4 (C), 164.2 (d, ^1^ *J* = 251.9 Hz, C), 159.4 (C), 146.9 (C), 136.6 (d, ^4^ *J* = 2.4 Hz, C), 133.04 (C), 132.95 (C), 131.9 (CH), 131.1 (d, ^3^ *J* = 8.98 Hz, CH), 130.4 (CH), 124.1 (C), 114.8 (d, ^2^ *J* = 21.7 Hz, CH), 111.5 (CH), 111.0 (C), 110.1 (CH), 61.9 (CH_2_), 60.7 (CH_2_), 52.3 (CH_3_), 41.4 (CH_2_), 14.3 (CH_3_), 14.0 (CH_3_); IR (neat): 3066, 2981, 2933, 2872, 1732, 1683, 1647, 1595, 1541, 1522, 1505, 1473, 1445, 1406, 1368, 1332, 1293, 1224, 1179, 1151, 1095, 1070, 1027, 1009, 912, 830, 768, 688, 606, 527, 502 cm^–1^; MS (ESI, *m*/*z*): 542.10 [M + H]^+^; HRMS (ESI, *m*/*z*): calcd. for C_27_H_26_^79^BrFNO_5_: 542.0978 [M + H]^+^, found: 542.0972; MS (ESI, *m*/*z*): 544.10 [M + H]^+^; HRMS (ESI, *m*/*z*): calcd. for C_27_H_26_^81^BrFNO_5_: 544.0958 [M + H]^+^, found: 544.0988.

#### Synthesis of 1,2,3,4,5-Pentasubstituted Benzene **5v** under Gold Catalysis

To a stirred solution of *N-*methyl*-N*-propargyl β-enaminone **3v** (1.00 mmol) in toluene (6 mL) under argon was added dimethyl acetylenedicarboxylate (**4a**) (90.0 mg, 0.66 mmol), AuCl (12.0 mg, 0.05 mmol) and AgSbF_6_ (26.0 mg, 0.08 mmol), and the resulting mixture was refluxed for approximately 5 h in an oil bath (The progress of the reaction was monitored by routine TLC analysis for the disappearance of β-enaminone **3v** using hexane/ethyl acetate (4:1) as the eluent). After the reaction was over, the solvent was removed on a rotary evaporator, and ethyl acetate (30 mL) and a saturated aqueous solution of NaCl (15 mL) were added. After the layers were separated, the aqueous layer was extracted with ethyl acetate (2 × 30 mL) again. The combined organic layers were dried over MgSO_4_ and evaporated on a rotary evaporator to give the crude product, which was purified by flash chromatography on silica gel using hexane/ethyl acetate (4:1) as the eluent to afford 30.0 mg (13%) of dimethyl 6′-benzoyl-[1,1′:3′,1″-terphenyl]-4′,5′-dicarboxylate (**5v**) as a yellowish-white solid (*R*_f_ = 0.35 in 4:1 hexane/ethyl acetate; mp 57.1–59.0 °C).

**5v:** ^1^H NMR (400 MHz, CDCl_3_) δ 8.05 (s, 1H), 7.34 (d, *J* = 7.5 Hz, 2H), 7.26 (t, *J* = 7.3 Hz, 2H), 7.23–7.18 (m, 3H), 7.17–7.12 (m, 3H), 7.09 (t, *J* = 7.6 Hz, 3H), 6.92 (s, 1H), 6.87 (s, 1H), 3.87 (s, 3H), 3.49 (s, 3H); ^13^C NMR (100 MHz, CDCl_3_) δ 196.8 (C), 168.6 (C), 165.7 (C), 143.3 (C), 141.3 (C), 138.9 (C), 138.5 (C), 137.4 (C), 135.7 (C), 135.2 (C), 133.3 (C), 130.8 (CH), 129.3 (CH), 129.3 (CH), 128.4 (CH), 128.2 (CH), 128.2 (CH), 128.1 (CH), 53.0 (CH_3_), 52.5 (CH_3_) (Note that three CH peaks overlap on each other); IR (neat): 3060, 3028, 2948, 2849, 1726, 1670, 1595, 1579, 1496, 1445, 1339, 1292, 1243, 1204, 1152, 1133, 1070, 1031, 1004, 972, 944, 875, 848, 801, 760, 699, 649, 602, 559, 530 cm^–1^; MS (ESI, *m*/*z*): 451.15 [M + H]^+^; HRMS (ESI, *m*/*z*): calcd. for C_29_H_23_O_5_: 451.1545 [M + H]^+^, found: 451.1545.

#### Synthesis of 1,2,3,4,5-Pentasubstituted Benzene **5v** under Copper Catalysis

To a stirred solution of *N-*methyl*-N*-propargyl β-enaminone **3v** (1.00 mmol) in acetonitrile (6 mL) under argon was added dimethyl acetylenedicarboxylate (**4a**) (130.0 mg, 0.90 mmol) and CuBr (7.0 mg, 0.05 mmol), and the resulting mixture was refluxed for approximately 5 h in an oil bath (The progress of the reaction was monitored by routine TLC analysis for the disappearance of β-enaminone **3v** using hexane/ethyl acetate (4:1) as the eluent). After the reaction was over, the solvent was removed on a rotary evaporator, and ethyl acetate (30 mL) and a saturated aqueous solution of NaCl (15 mL) were added. After the layers were separated, the aqueous layer was extracted with ethyl acetate (2 × 30 mL) again. The combined organic layers were dried over MgSO_4_ and evaporated on a rotary evaporator to give the crude product, which was purified by flash chromatography on silica gel using hexane/ethyl acetate (5:1 followed by 2:1) as the eluent to afford 24.7 mg (12%) of dimethyl 6′-benzoyl-[1,1′:3′,1″-terphenyl]-4′,5′-dicarboxylate (**5v**) as a yellowish-white solid (*R*_f_ = 0.35 in 4:1 hexane/ethyl acetate; mp 57.1–59.0 °C).

#### Synthesis of (2-(4-Chlorophenyl)-1-methyl-1,6-dihydropyridin-3-yl)­(phenyl)­methanone (**8h**)

To a stirred solution of the corresponding *N*-methyl-*N*-propargyl β-enaminone **3h** (127.0 mg, 0.41 mmol) in DMSO (5.00 mL) under argon was added CuBr (23.0 mg, 0.16 mmol) and the resulting mixture was stirred at 60 °C for approximately 1.5 h (The progress of the reaction was followed by routine TLC for the disappearance of β-enaminone **3h** using hexane/ethyl acetate (4:1) as the eluent). After the reaction was over, ethyl acetate (30 mL) and saturated aqueous NaCl solution (30 mL) were added to the reaction flask. After the layers were separated, the aqueous phase was extracted with ethyl acetate (2 × 30 mL). The combined organic phases were dried over MgSO_4_ and evaporated on a rotary evaporator to give a crude product, which was purified by flash chromatography on silica gel using hexane/ethyl acetate (4:1) as the eluent to afford 5.0 mg (5%) of the indicated product **8h** as dark orange solid (*R*_f_ = 0.29 in 4:1 hexane/ethyl acetate; mp 101.3–101.8 °C).

**8h:** ^1^H NMR (400 MHz, CDCl_3_) δ 7.36 (d, *J* = 7.1 Hz, 2H), 7.21 (t, *J* = 7.4 Hz, 1H), 7.14–7.11 (m, 4H), 7.04 (d, *J* = 8.4 Hz, 2H), 6.69 (d, *J* = 9.5 Hz, 1H), 5.14 (dt, *J* = 9.5, 3.7 Hz, 1H), 4.19 (dd, *J* = 3.6, 1.1 Hz, 2H), 2.70 (s, 3H); ^13^C NMR (100 MHz, CDCl_3_) δ 193.5 (C), 157.7 (C), 141.5 (C), 134.9 (C), 133.6 (C), 131.0 (CH), 129.8 (CH), 128.6 (CH), 128.4 (CH), 127.4 (CH), 126.7 (CH), 111.9 (C), 107.3 (CH), 52.3 (CH_3_), 40.6 (CH_2_); IR (neat): 3058, 2928, 1734, 1671, 1637, 1593, 1558, 1509, 1465, 1442, 1409, 1383, 1331, 1309, 1261, 1199, 1084, 1010, 970, 942, 921, 819, 762, 746, 723, 708, 665, 651, 618, 542, 519 cm^–1^; MS (ESI, *m*/*z*): 310.10 [M + H]^+^; HRMS (ESI, *m*/*z*): calcd. for C_19_H_17_NOCl: 310.0999 [M + H]^+^, found: 310.0999.

## Supplementary Material



## Notes

Copies of ^1^H and ^13^C NMR spectra for all starting materials and products (PDF)
