# Supplementary material for: Rapid Access to 1,2,3,4-Tetrasubstituted Benzenes and 3‑Alkenyl-1,2-dihydropyridines
Source: ACS Omega. 2026 May 4;11(19):29061–84. doi: 10.1021/acsomega.6c02327 (PMC13191555; doi:10.1021/acsomega.6c02327)
Supplement: Supplementary file 1 [file ao6c02327_si_001.pdf]

<u>SUPPORTING INFORMATION</u>

Rapid Access to 1,2,3,4-Tetrasubstituted Benzenes and 3-Alkenyl-1,2-dihydropyridines

Buse Aysen Dundar Ozdogan and Metin Zora*

Department of Chemistry, Faculty of Arts and Science, Middle East Technical University, 06800 Ankara, Turkey

<u>Table of Contents</u>

Copies of ^{1}H and $^{13}C\{^{1}H\}$ NMR Spectra for *N*-Methyl-*N*-propargyl β-Enaminones 3………………………………………… S2

Copies of ^{1}H and $^{13}C\{^{1}H\}$ NMR Spectra for 1,2,3,4-Tetrasubstituted Benzenes 5 ve 5'………………………………………… S25

Copies of ^{1}H and $^{13}C\{^{1}H\}$ NMR Spectra for 3-Alkenyl-1,2-dihydropyridines 6 ve 6'………………………………………… S53

* Corresponding Author. E-mail address: zora@metu.edu.tr (M. Zora)

Copies of ^{1}H and ^{13}C{^{1}H} NMR Spectra for *N*-Methyl-*N*-propargyl β-Enaminones 3

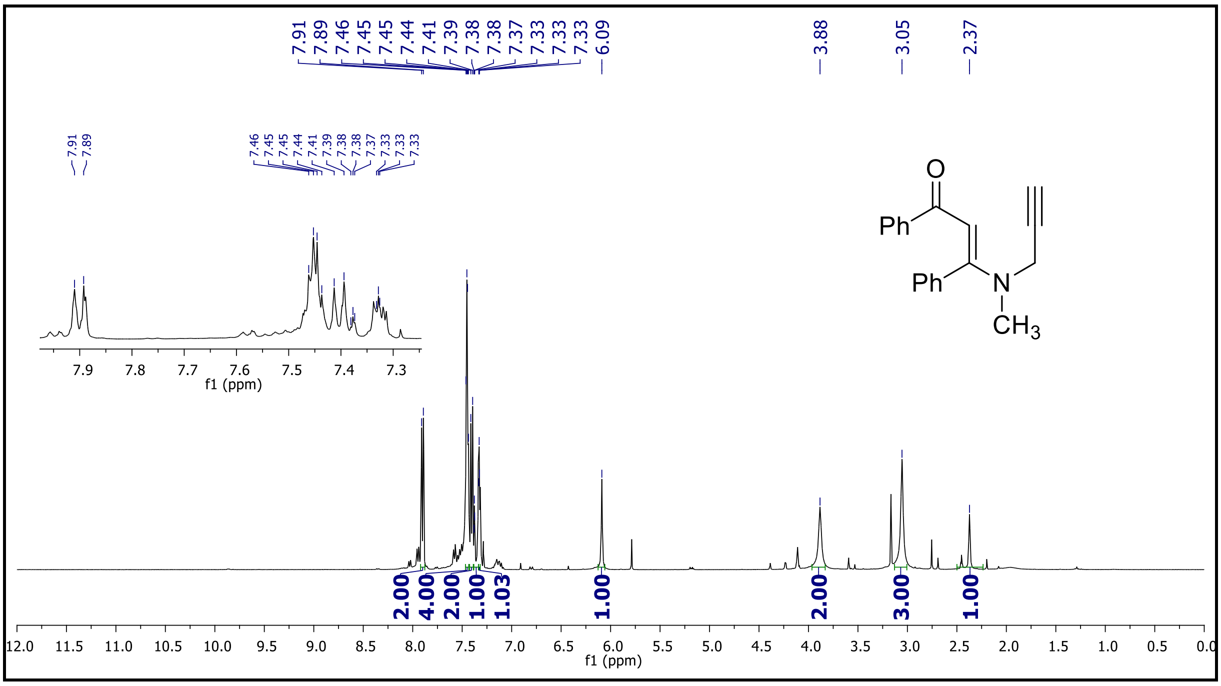

Figure S1. ^{1}H NMR (400 MHz, $CDCl_3$) spectrum of compound **3a**.

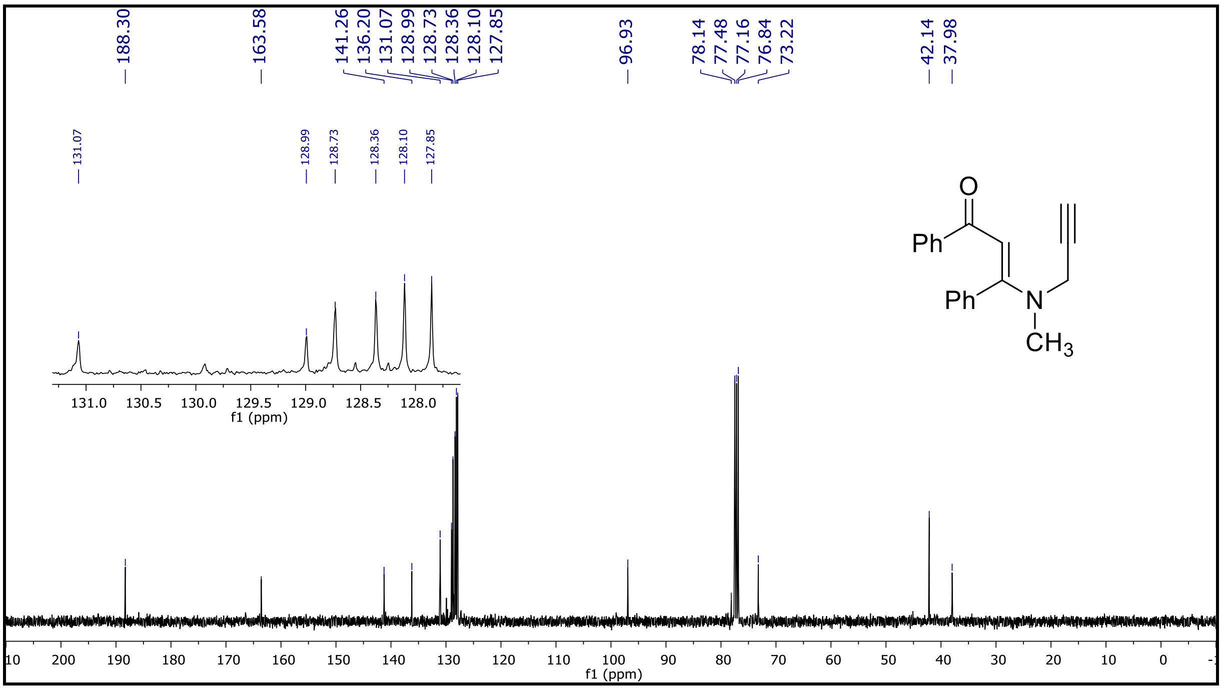

Figure S2. ^{13}C{^{1}H} NMR (100 MHz, $CDCl_3$) spectrum of compound **3a**.

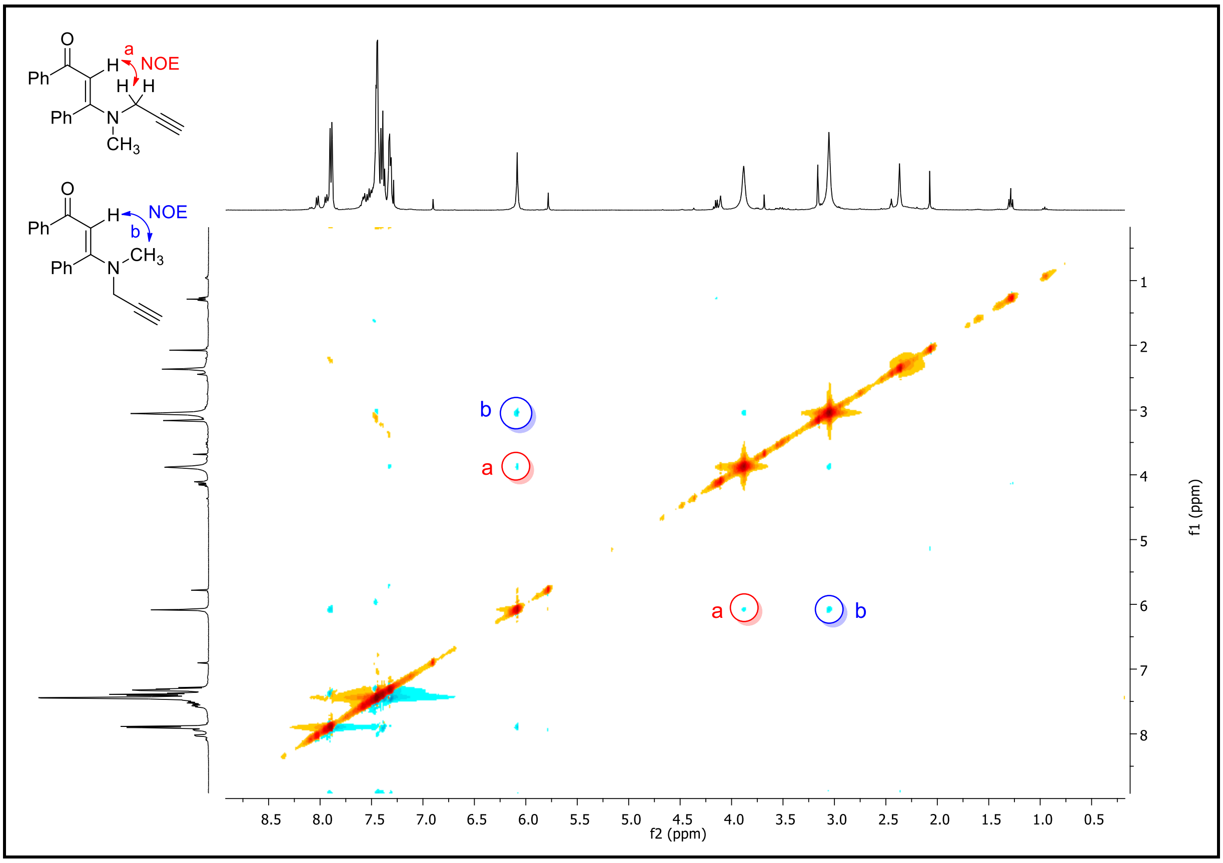

Figure S3. $^{1}H/^{1}H$ NOESY (400/400 MHz, $CDCl_3$) spectrum of compound **3a**.

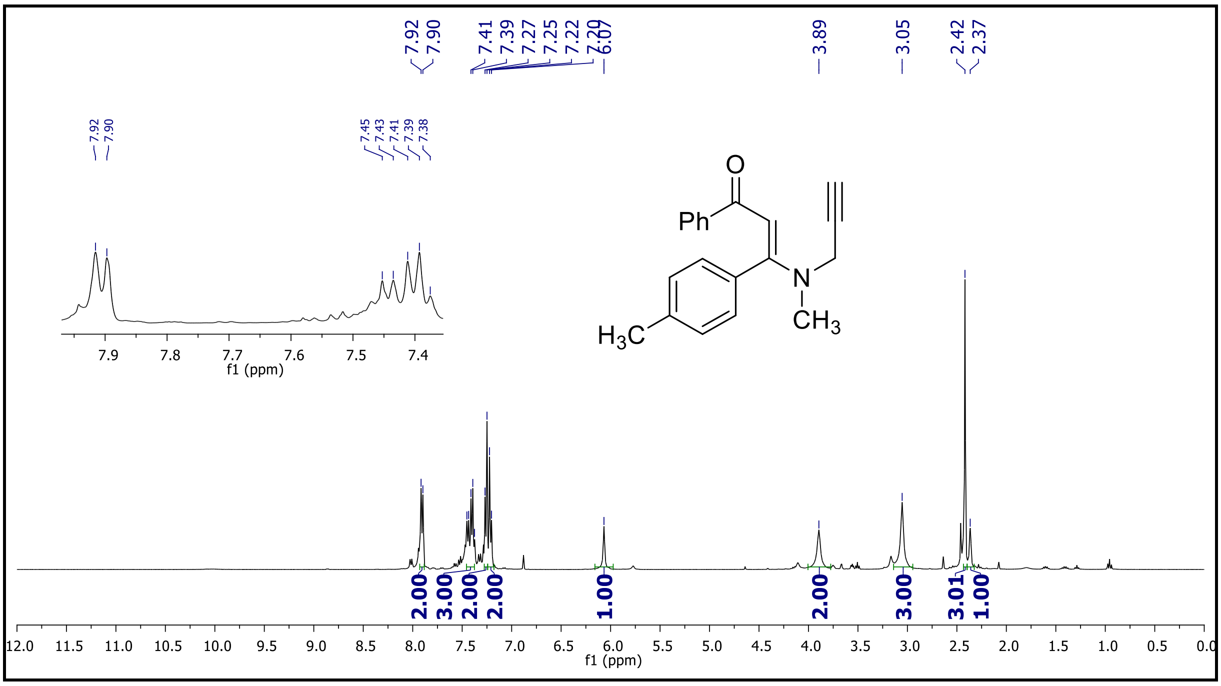

Figure S4. ^{1}H NMR (400 MHz, $CDCl_3$) spectrum of compound **3b**.

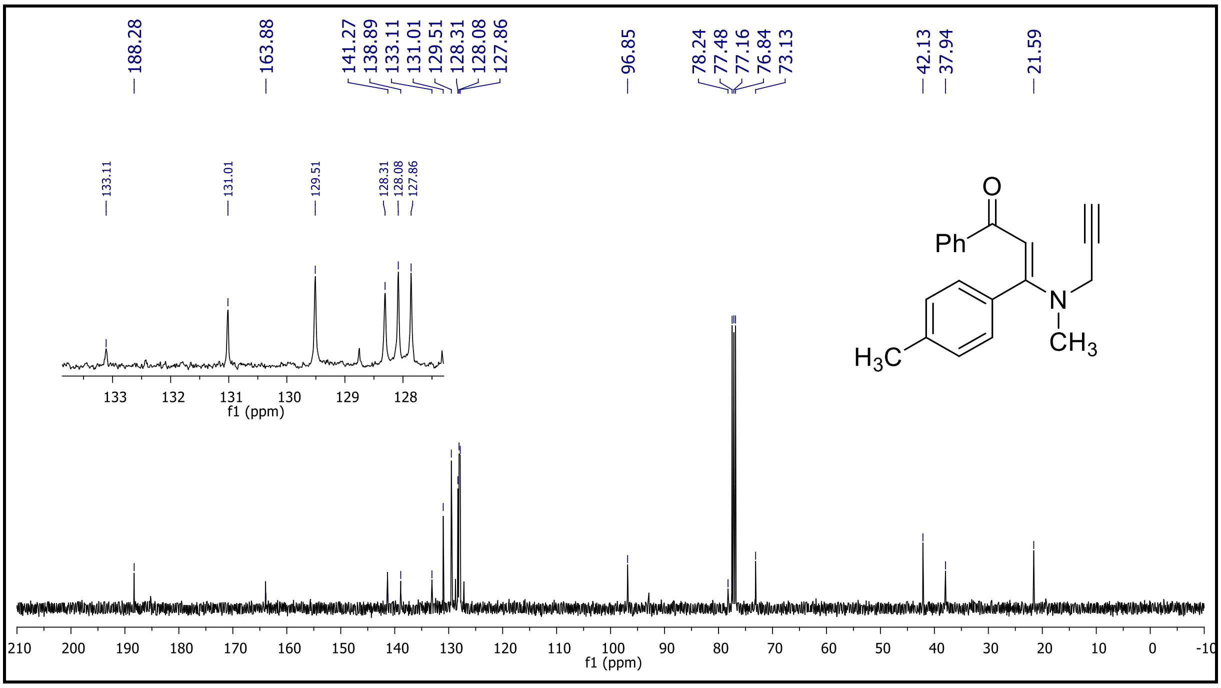

Figure S5. $^{13}C\{1H\}$ NMR (100 MHz, $CDCl_3$) spectrum of compound **3b**.

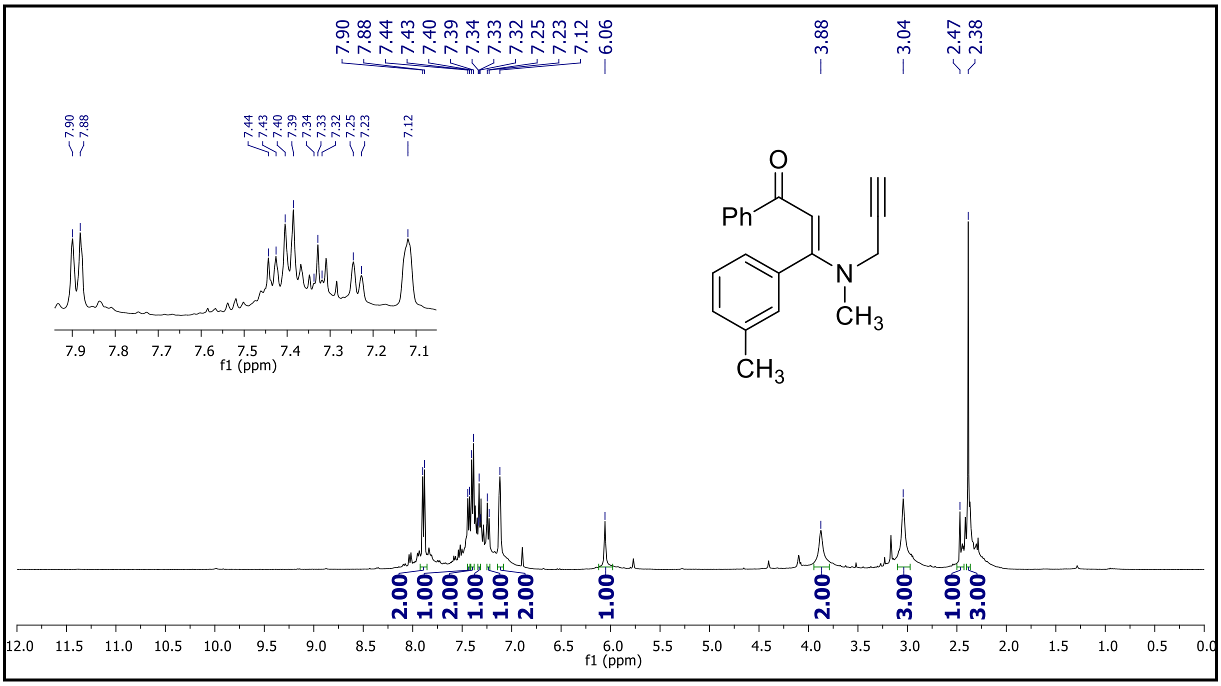

Figure S6. ^{1}H NMR (400 MHz, $CDCl_3$) spectrum of compound **3c**.

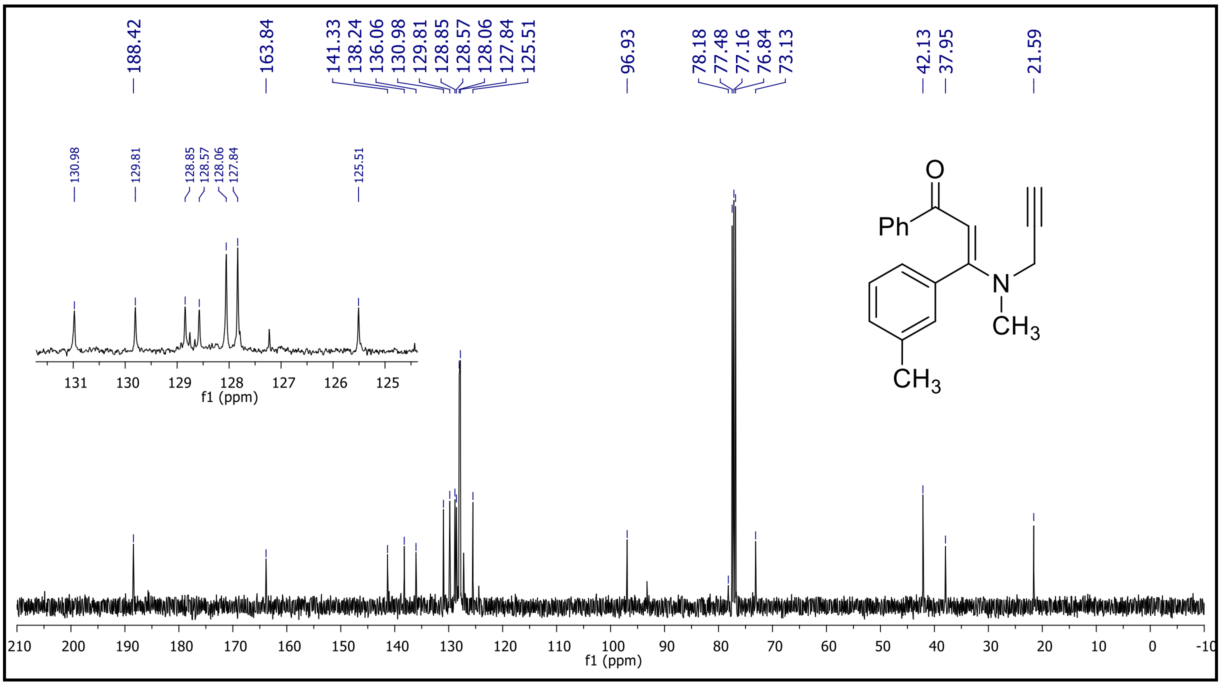

Figure S7. $^{13}C\{^{1}H\}$ NMR (100 MHz, $CDCl_3$) spectrum of compound **3c**.

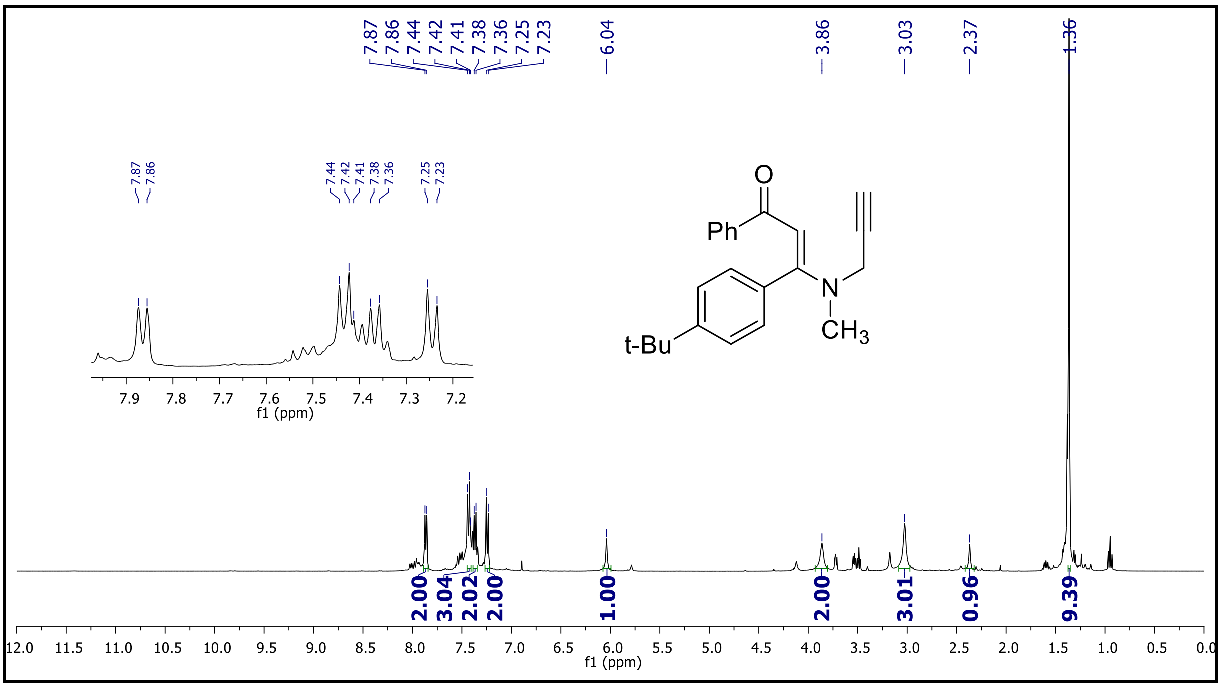

Figure S8. ^{1}H NMR (400 MHz, $CDCl_3$) spectrum of compound **3d**.

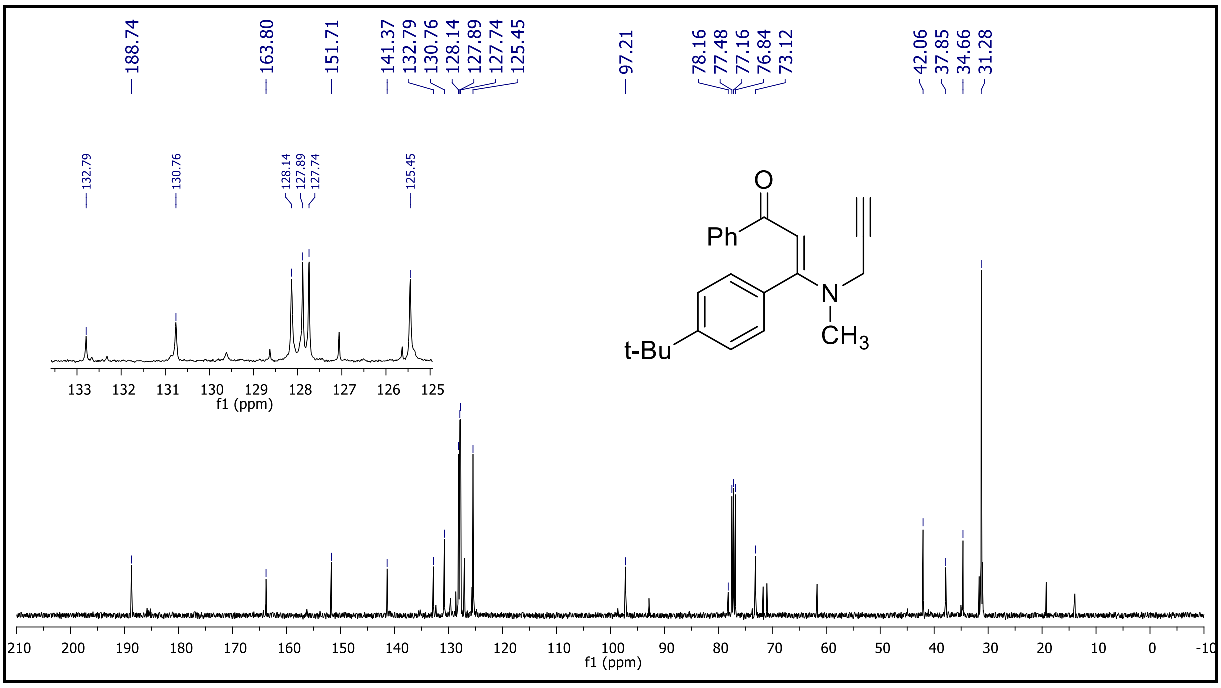

Figure S9. $^{13}C\{^{1}H\}$ NMR (100 MHz, $CDCl_3$) spectrum of compound **3d**.

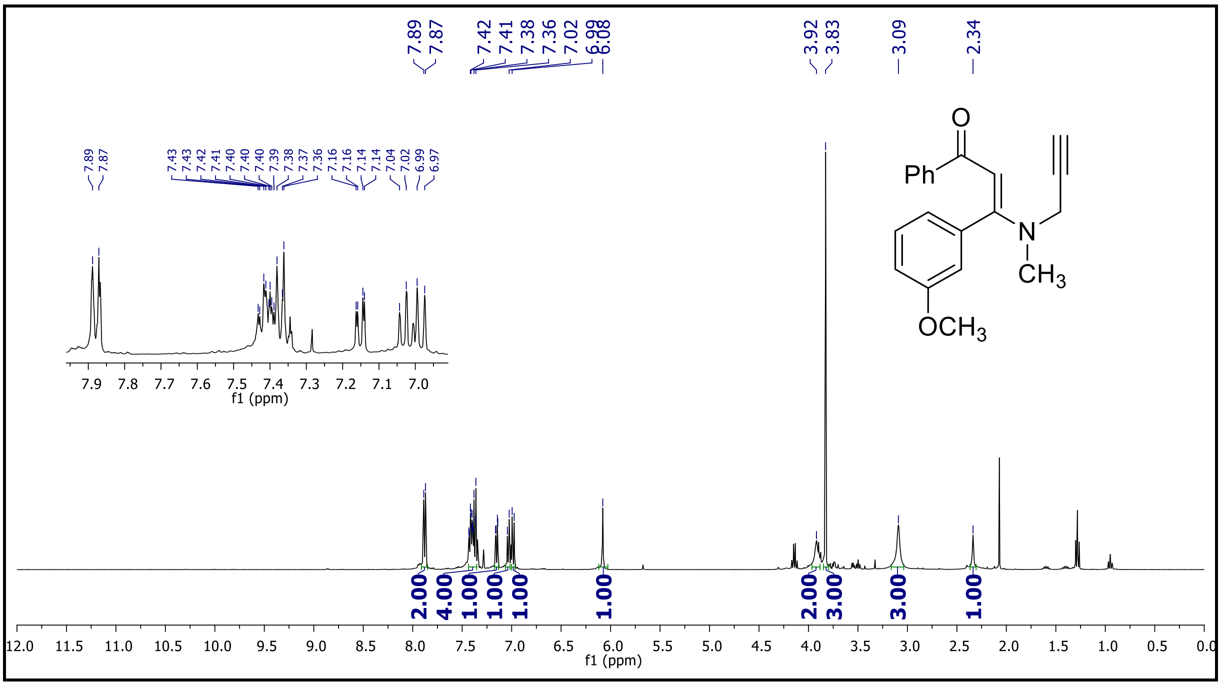

Figure S10. ^{1}H NMR (400 MHz, $CDCl_3$) spectrum of compound **3e**.

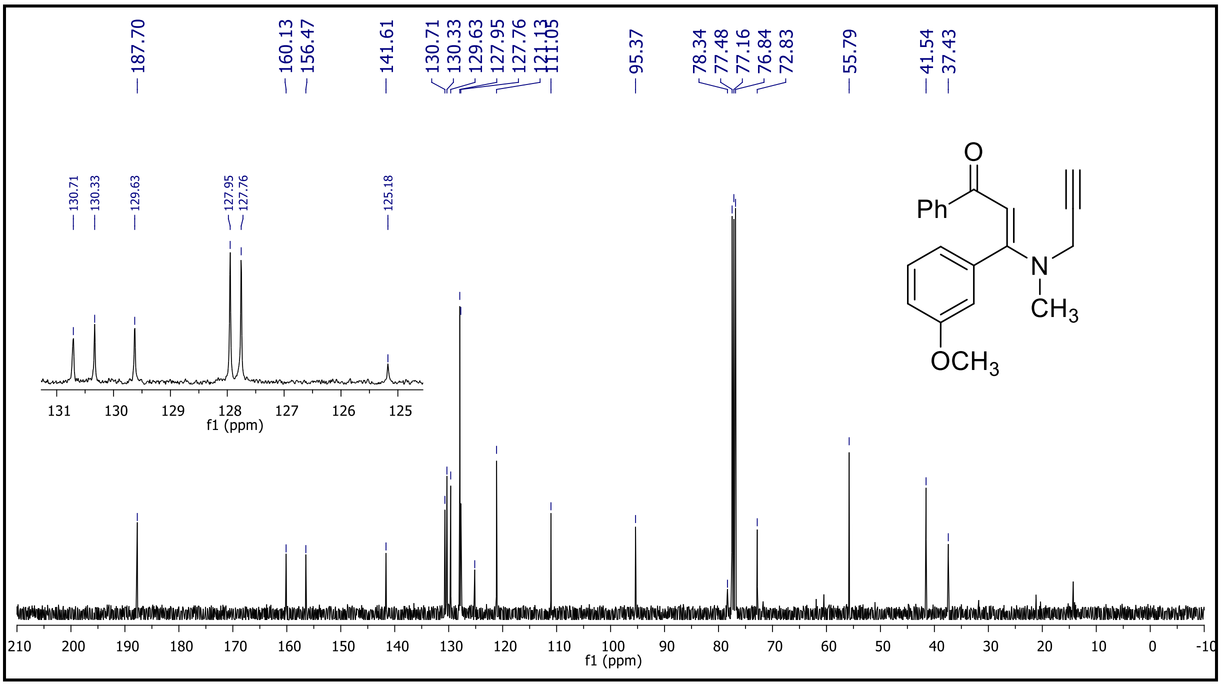

Figure S11. $^{13}C\{^1H\}$ NMR (100 MHz, $CDCl_3$) spectrum of compound **3e**.

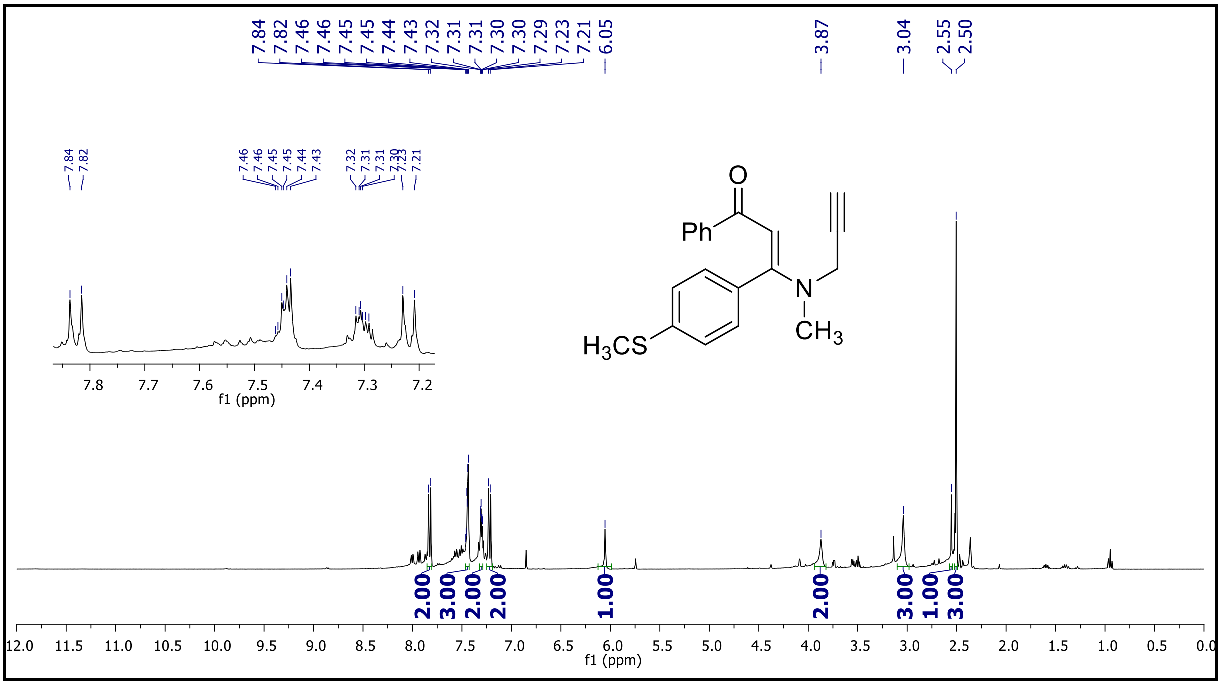

Figure S12. ^1H NMR (400 MHz, $CDCl_3$) spectrum of compound **3f**.

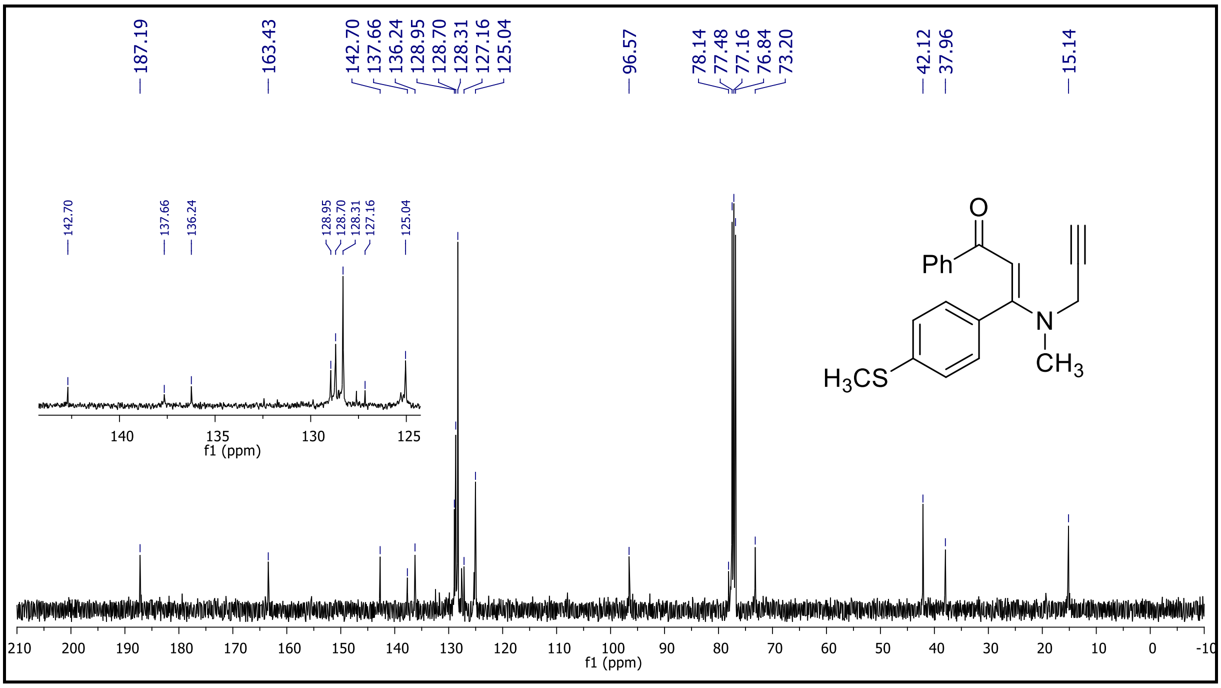

Figure S13. $^{13}C\{^{1}H\}$ NMR (100 MHz, $CDCl_3$) spectrum of compound **3f**.

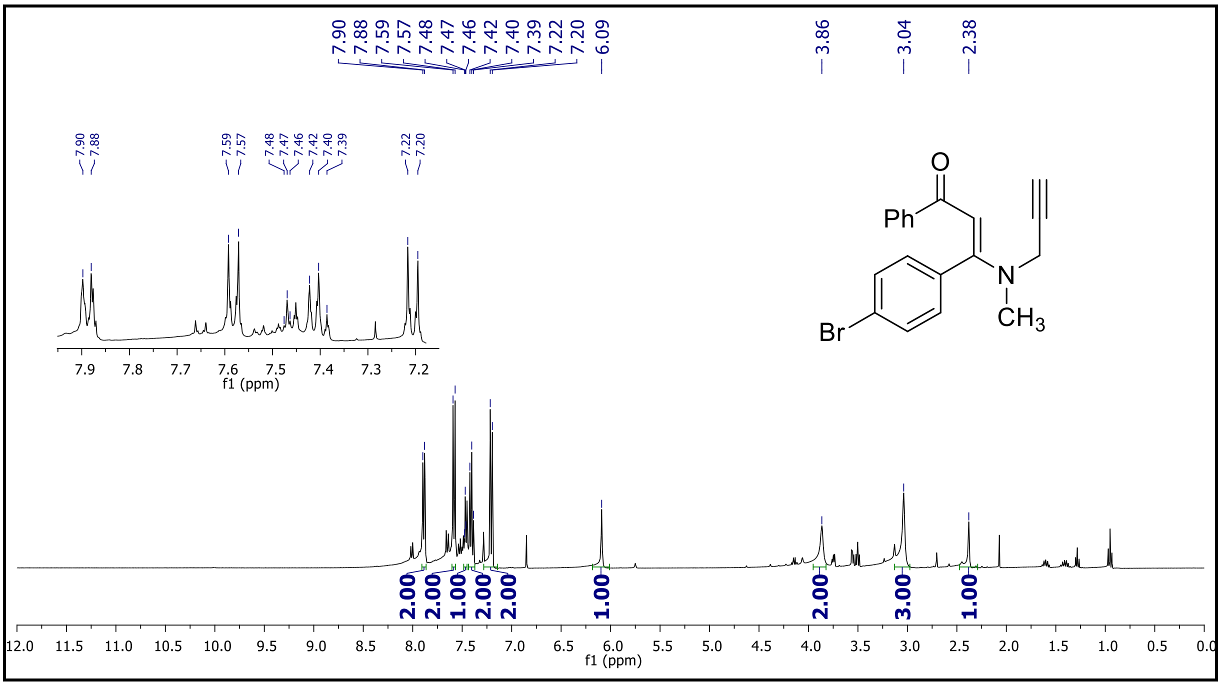

Figure S14. ^{1}H NMR (400 MHz, $CDCl_3$) spectrum of compound **3g**.

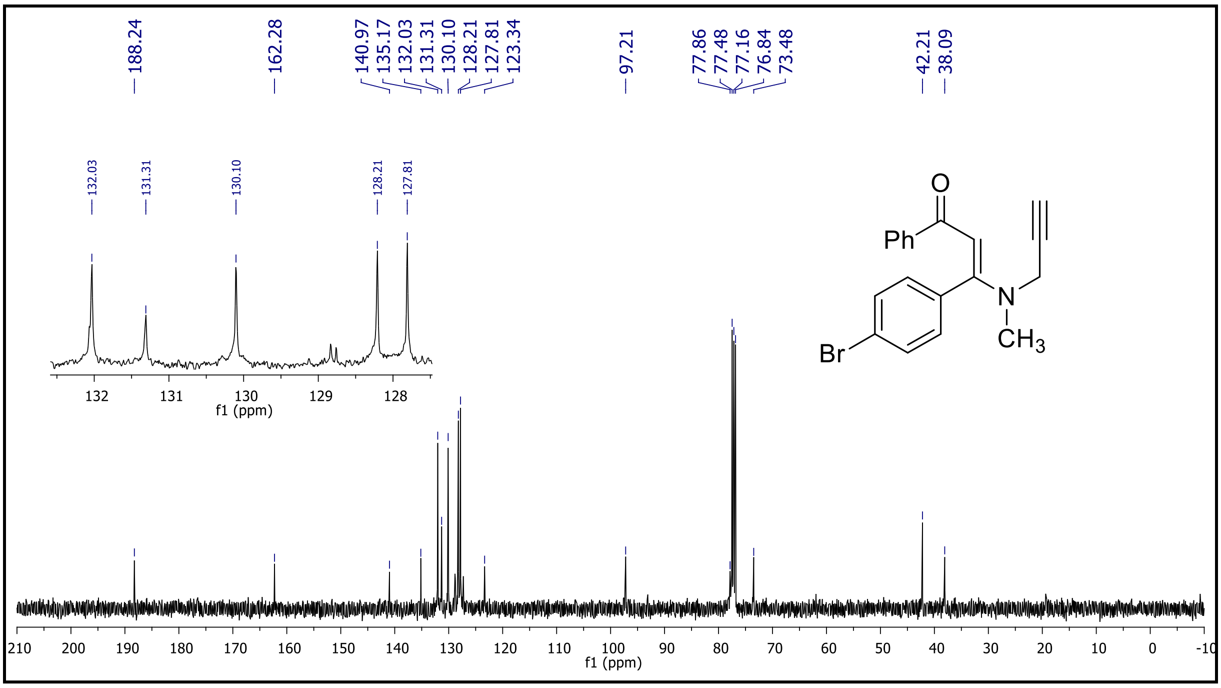

Figure S15. $^{13}C\{^1H\}$ NMR (100 MHz, $CDCl_3$) spectrum of compound **3g**.

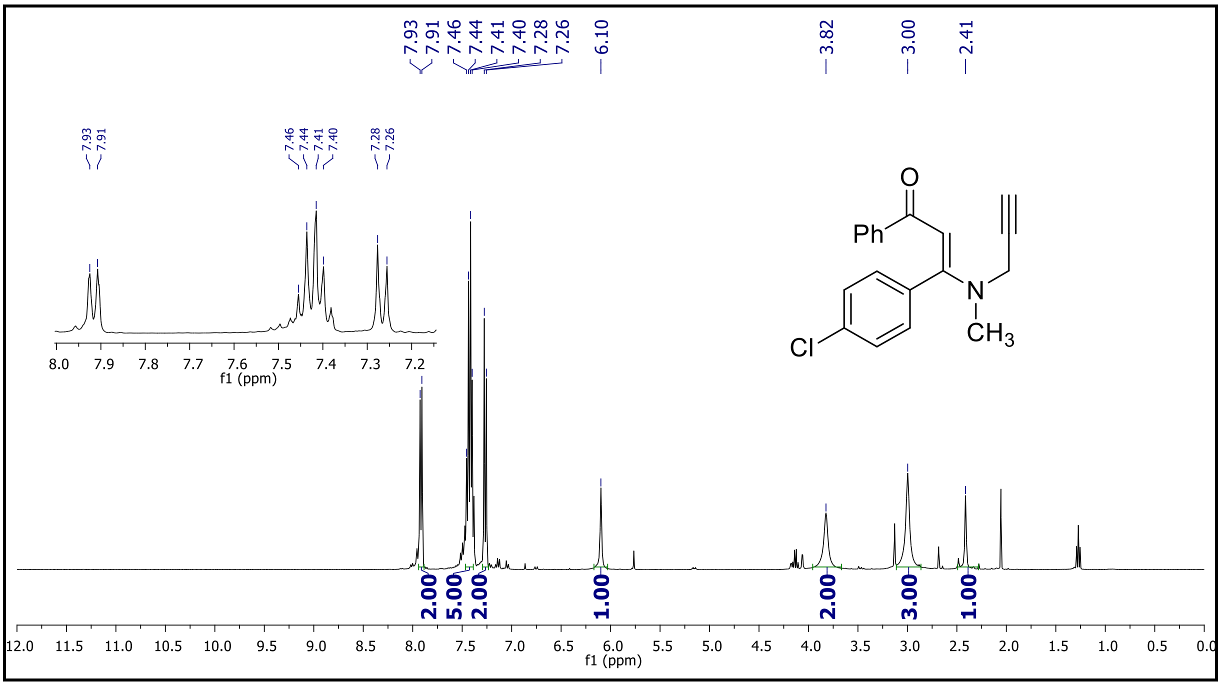

Figure S16. ^1H NMR (400 MHz, $CDCl_3$) spectrum of compound **3h**.

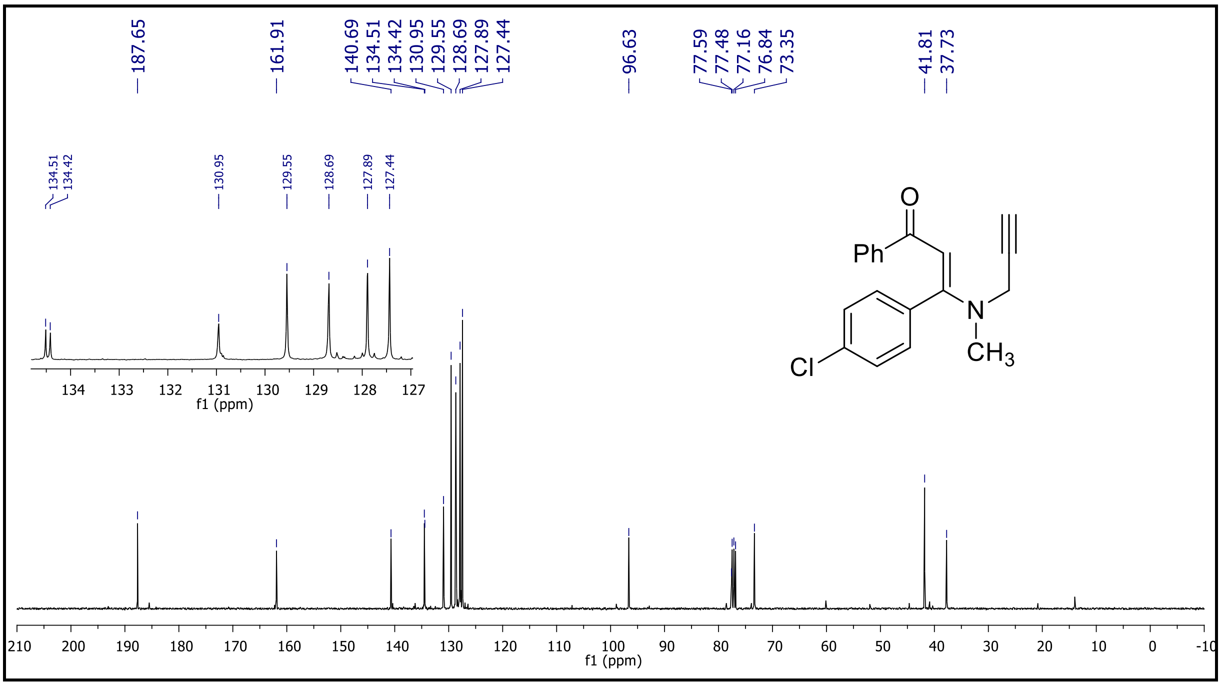

Figure S17. $^{13}C\{^{1}H\}$ NMR (100 MHz, $CDCl_3$) spectrum of compound **3h**.

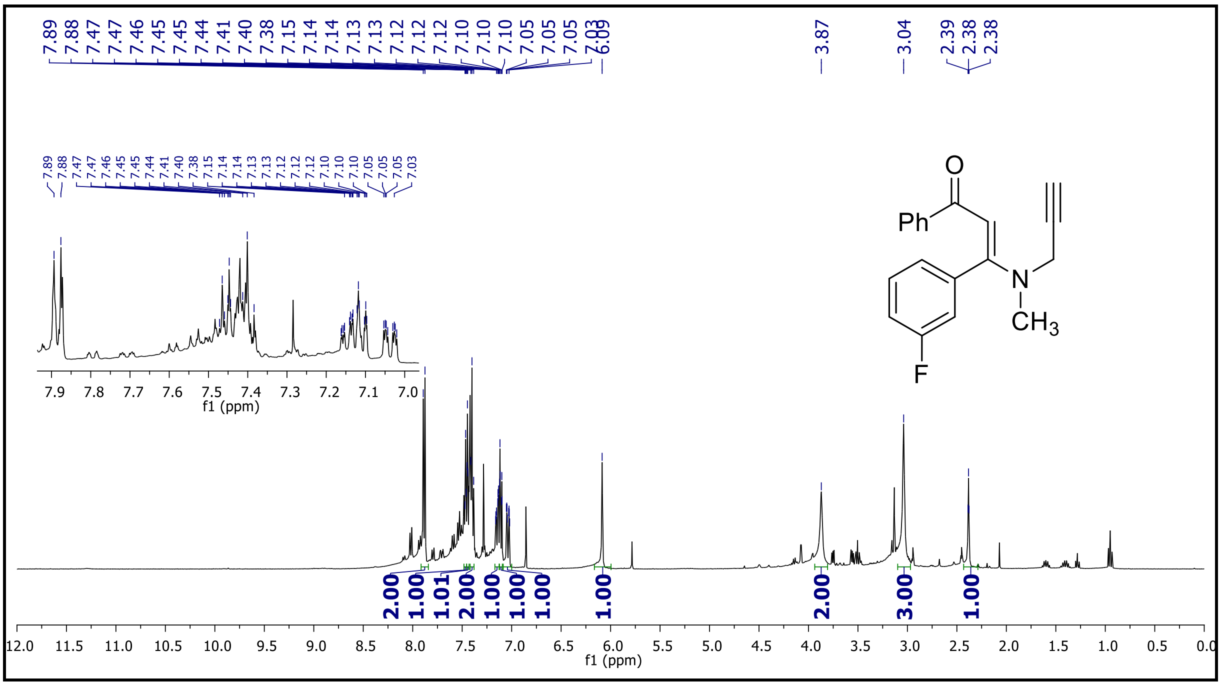

Figure S18. ^{1}H NMR (400 MHz, $CDCl_3$) spectrum of compound **3i**.

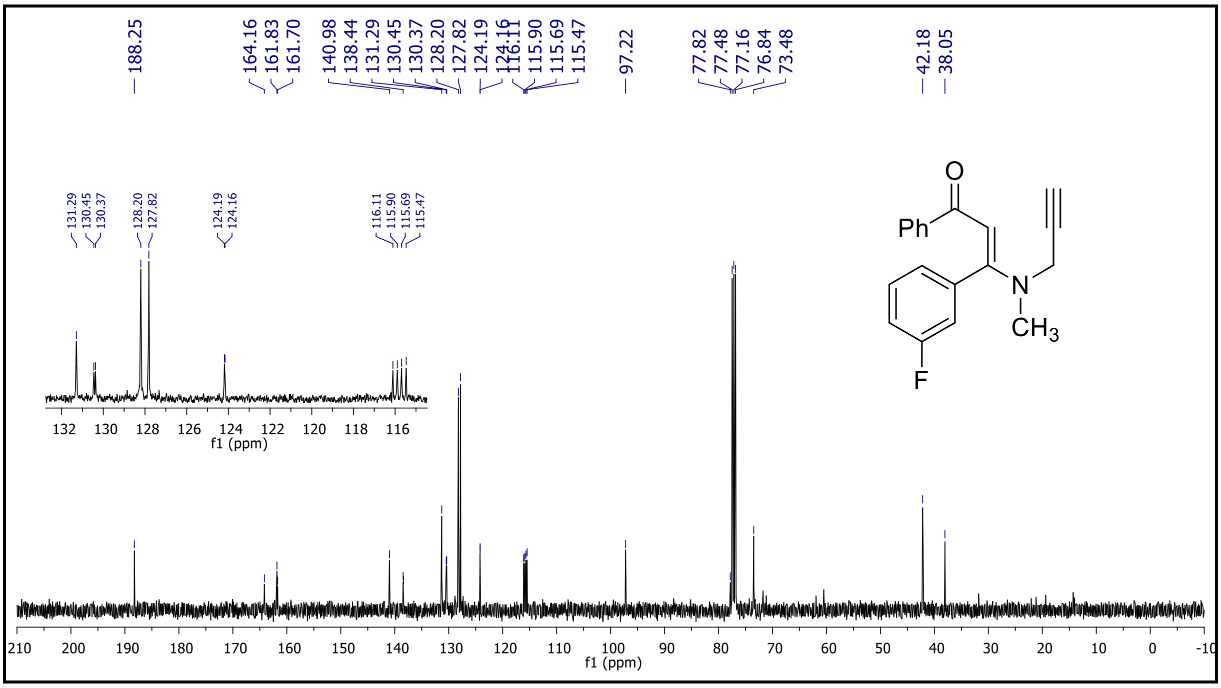

Figure S19. $^{13}C\{^{1}H\}$ NMR (100 MHz, $CDCl_3$) spectrum of compound **3i**.

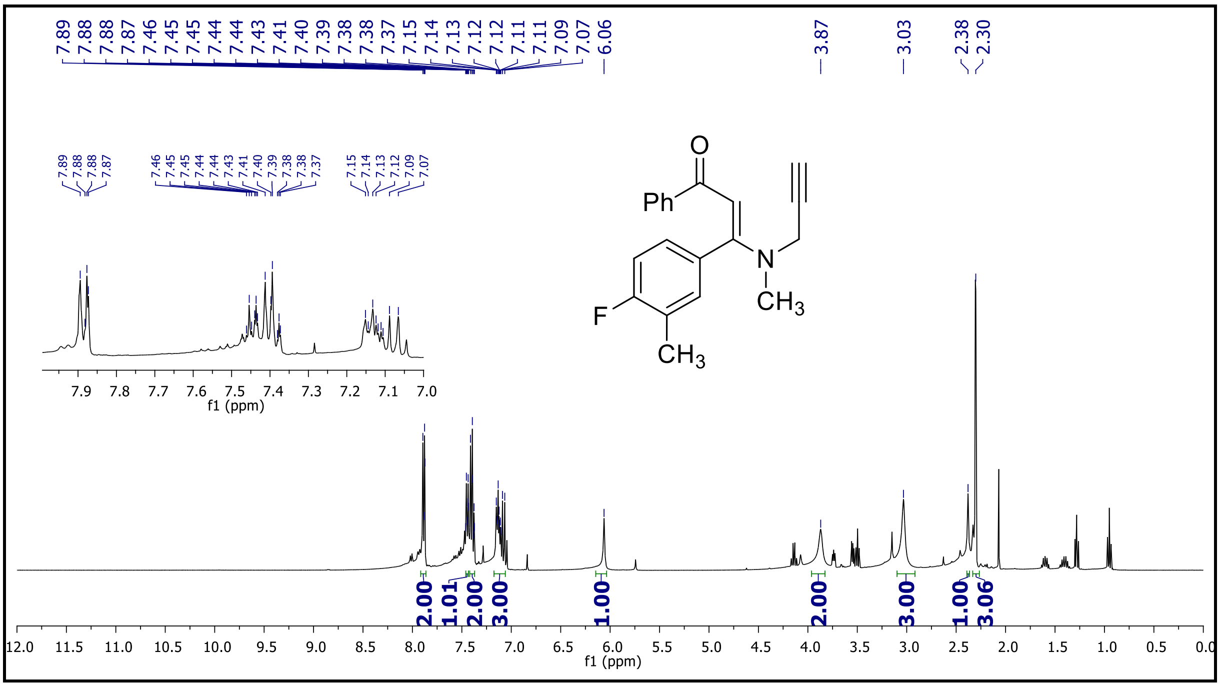

Figure S20. ^{1}H NMR (400 MHz, $CDCl_3$) spectrum of compound **3j**.

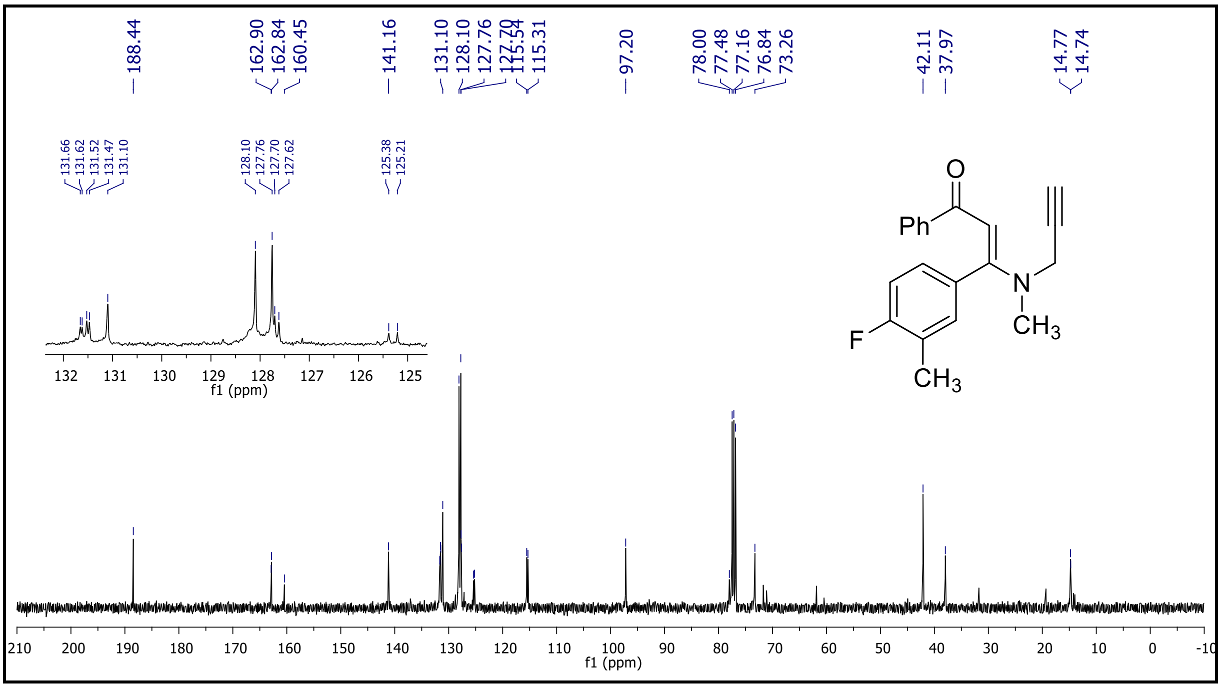

Figure S21. $^{13}C\{^1H\}$ NMR (100 MHz, $CDCl_3$) spectrum of compound **3j**.

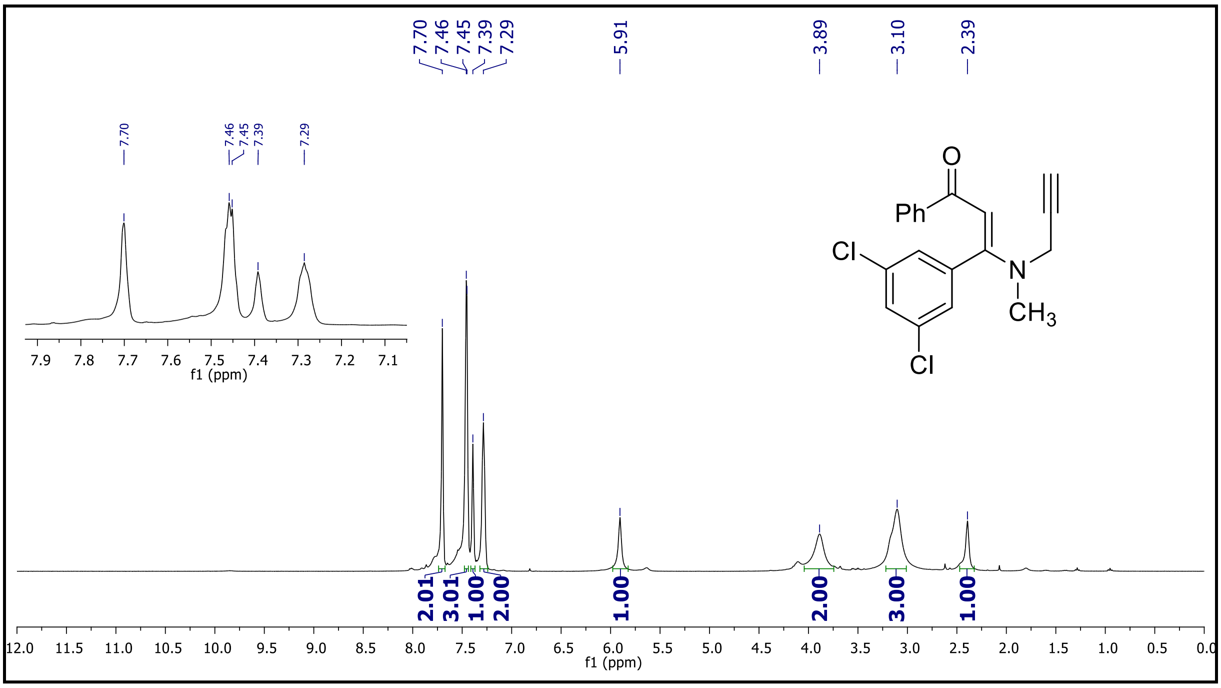

Figure S22. ^1H NMR (400 MHz, $CDCl_3$) spectrum of compound **3k**.

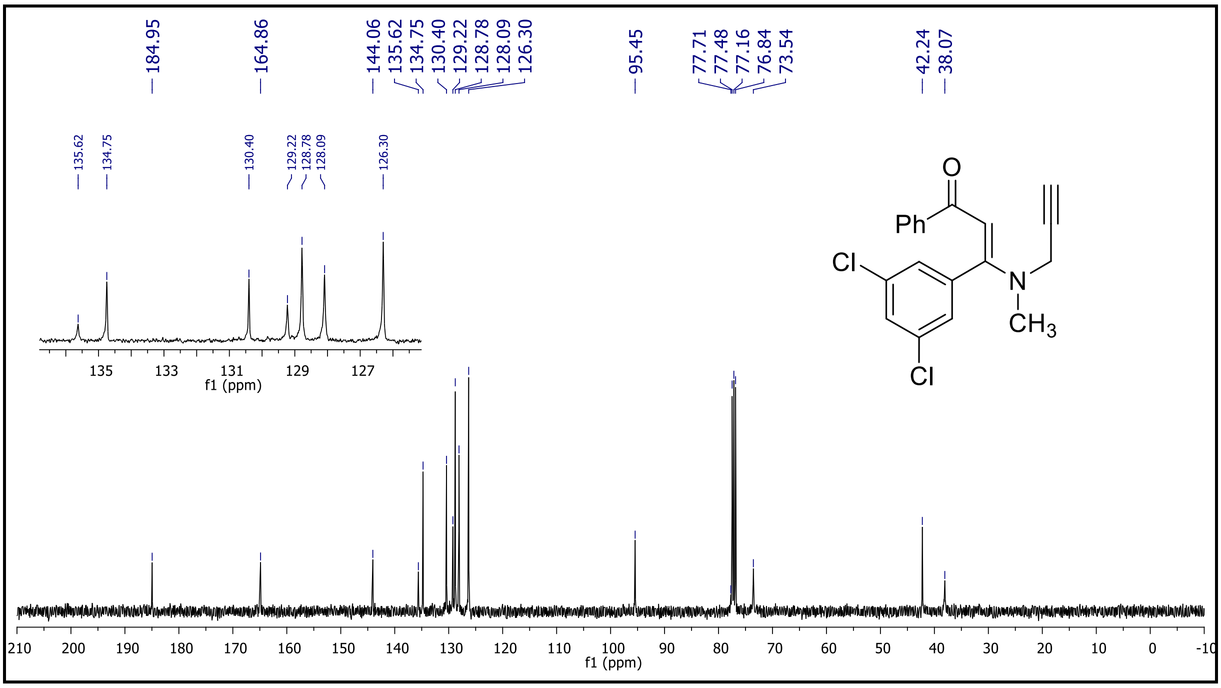

Figure S23. $^{13}C\{^{1}H\}$ NMR (100 MHz, $CDCl_3$) spectrum of compound **3k**.

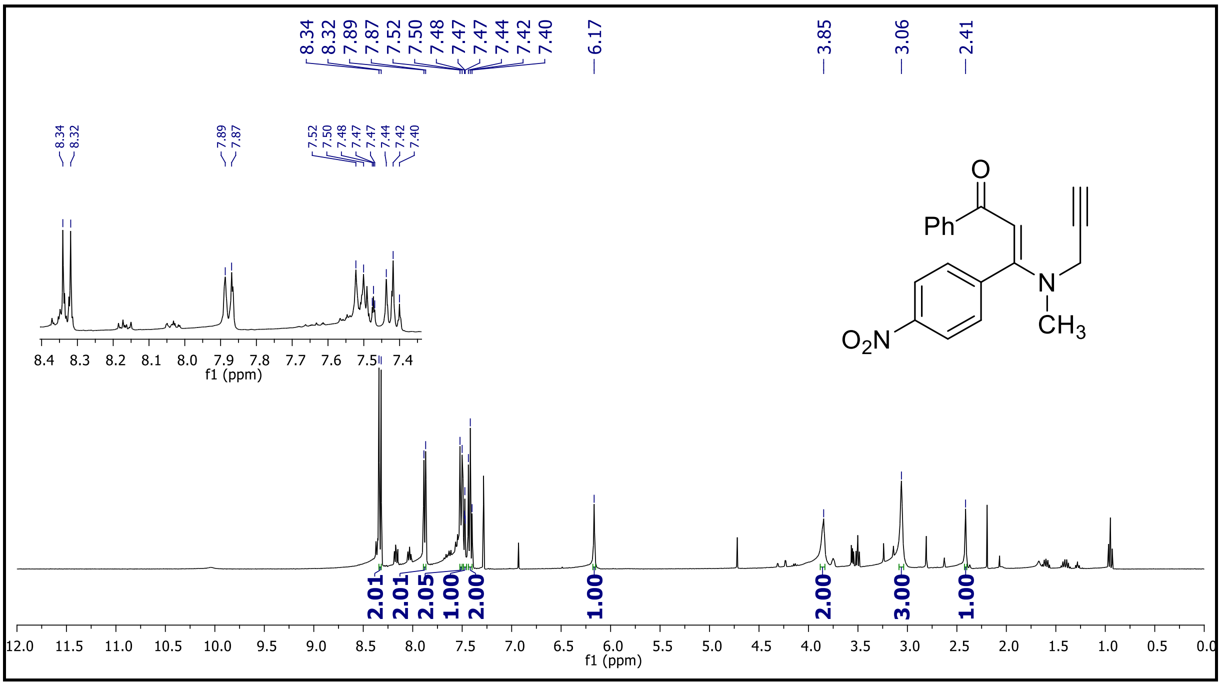

Figure S24. ^{1}H NMR (400 MHz, $CDCl_3$) spectrum of compound **3l**.

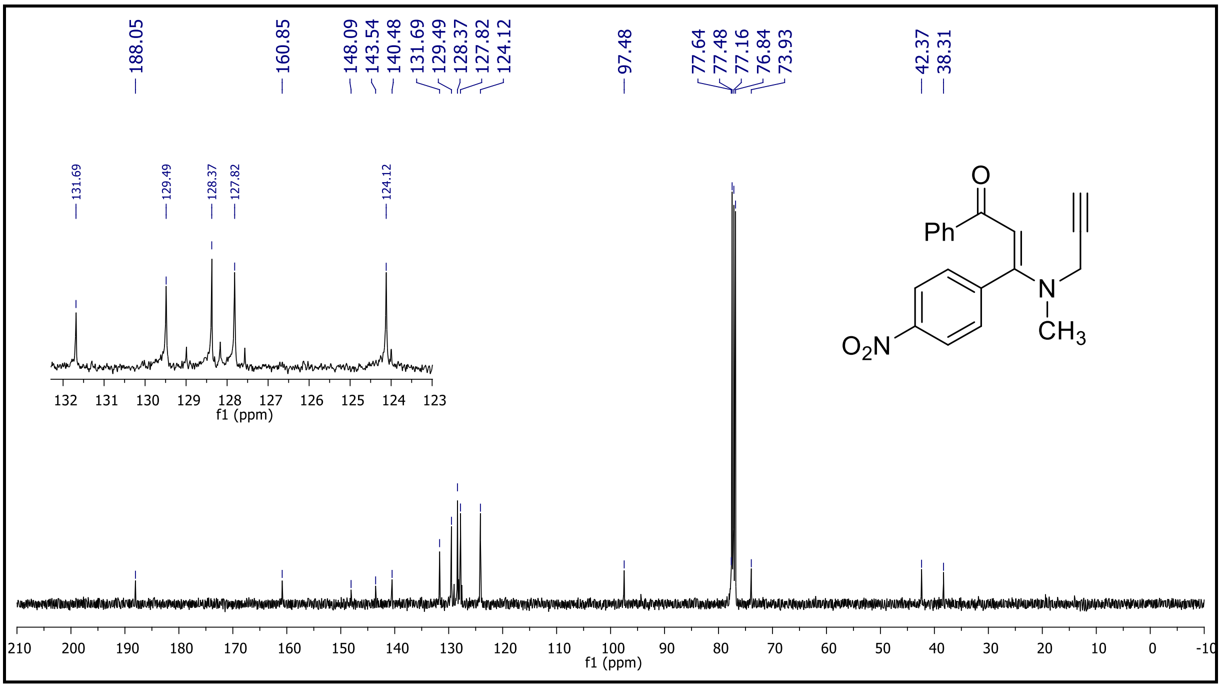

Figure S25. $^{13}C\{^1H\}$ NMR (100 MHz, $CDCl_3$) spectrum of compound **3l**.

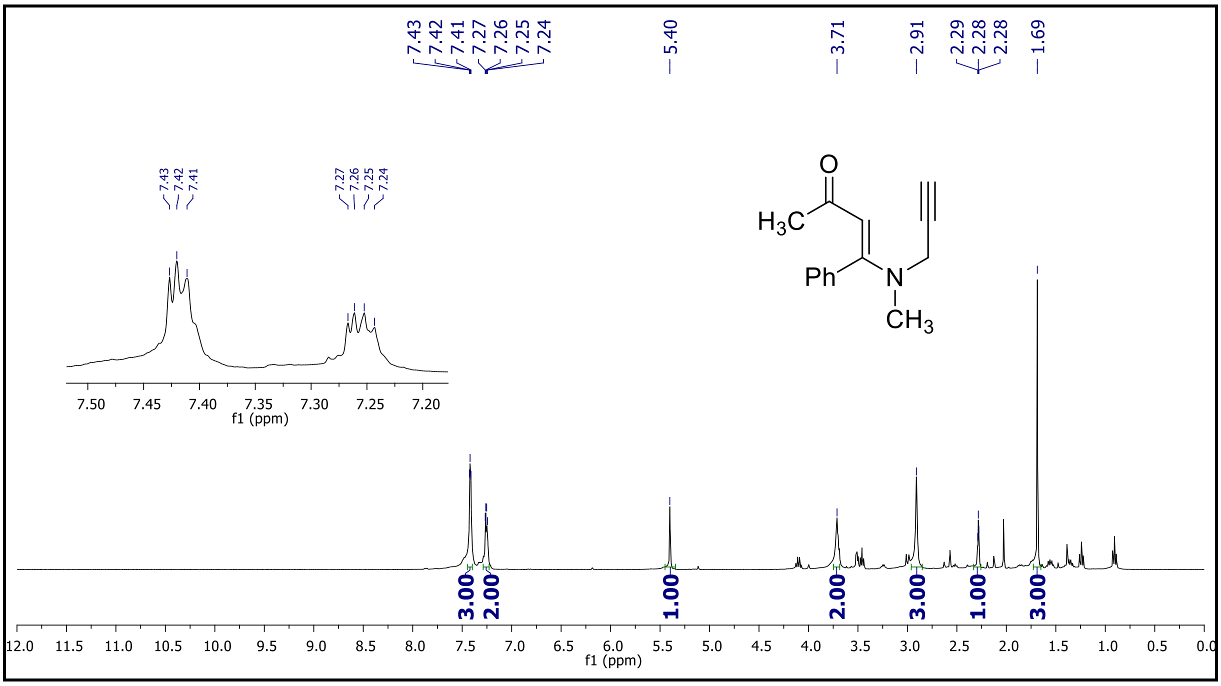

Figure S26. ^1H NMR (400 MHz, $CDCl_3$) spectrum of compound **3m**.

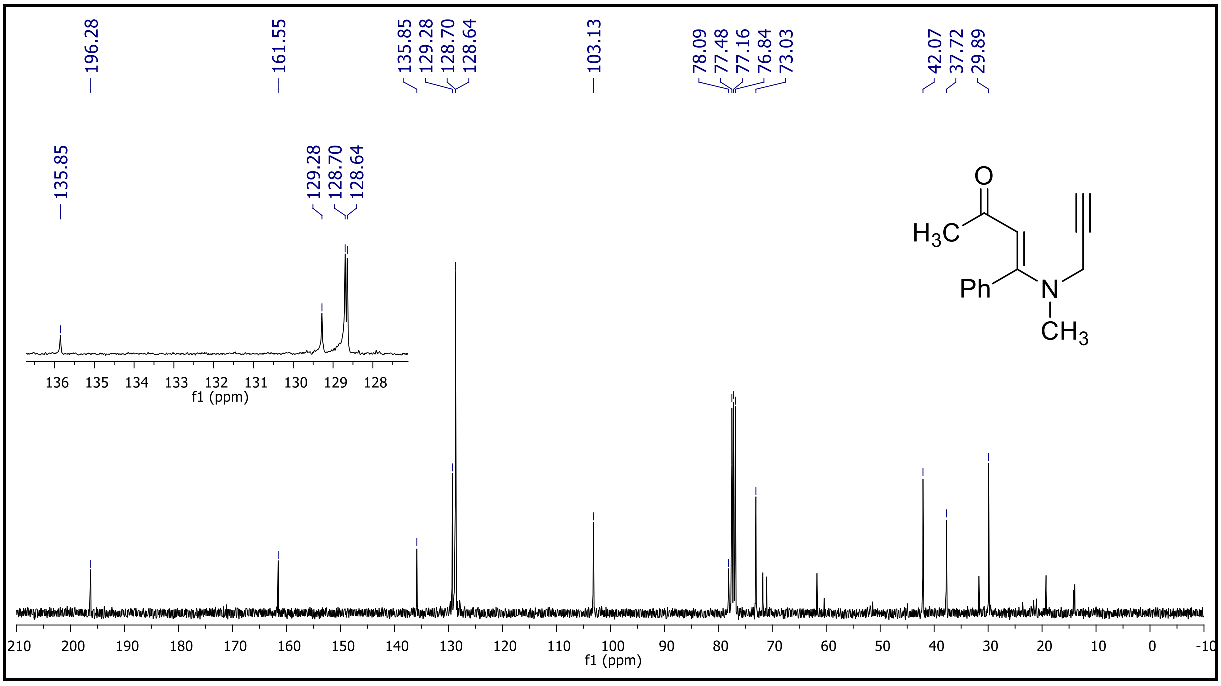

Figure S27. $^{13}C\{^1H\}$ NMR (100 MHz, $CDCl_3$) spectrum of compound **3m**.

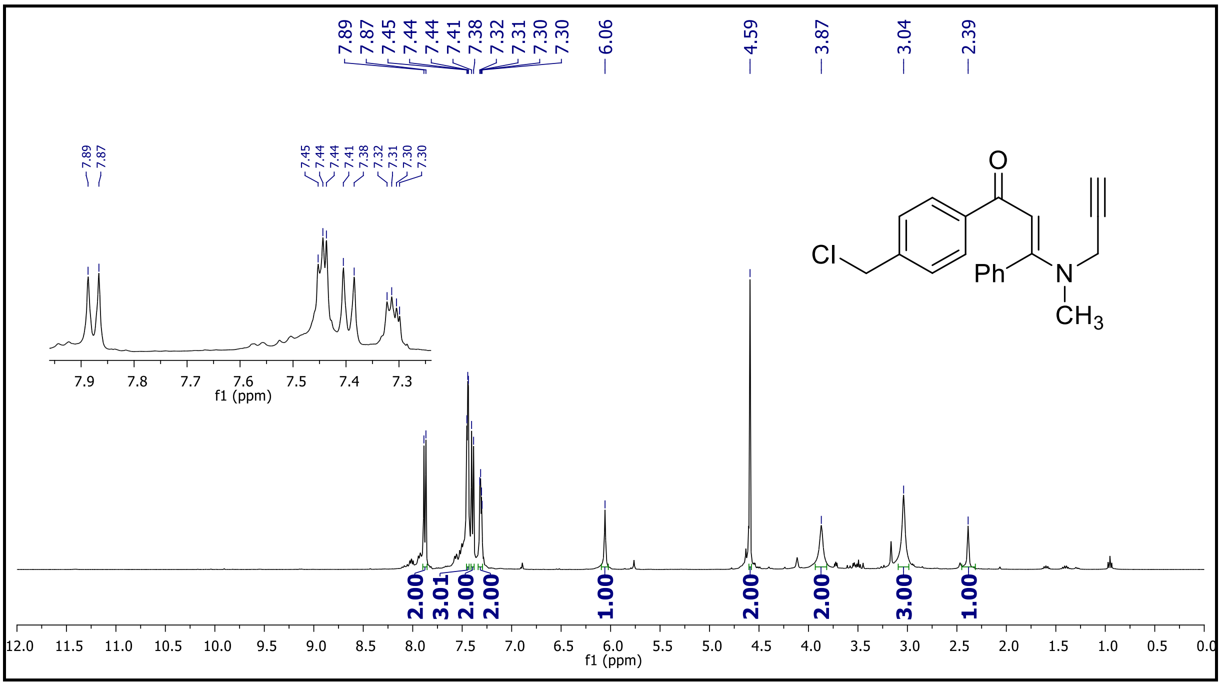

Figure S28. ^1H NMR (400 MHz, $CDCl_3$) spectrum of compound **3n**.

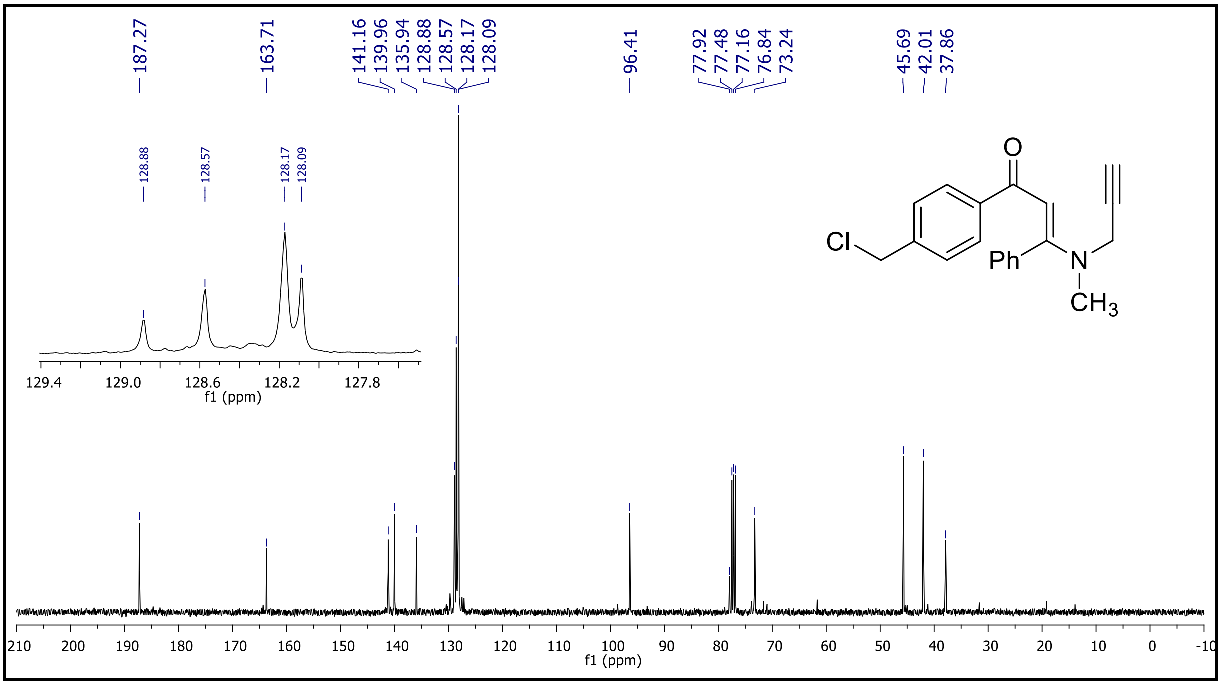

Figure S29. $^{13}C\{^1H\}$ NMR (100 MHz, $CDCl_3$) spectrum of compound **3n**.

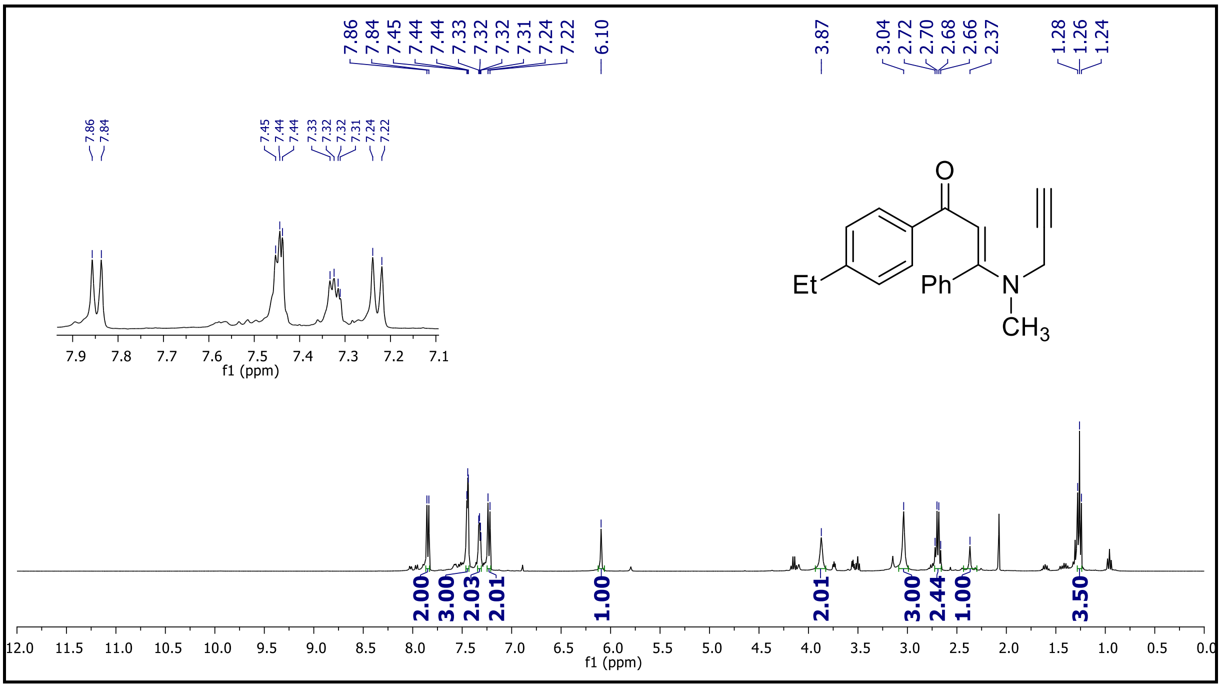

Figure S30. ^1H NMR (400 MHz, $CDCl_3$) spectrum of compound **3o**.

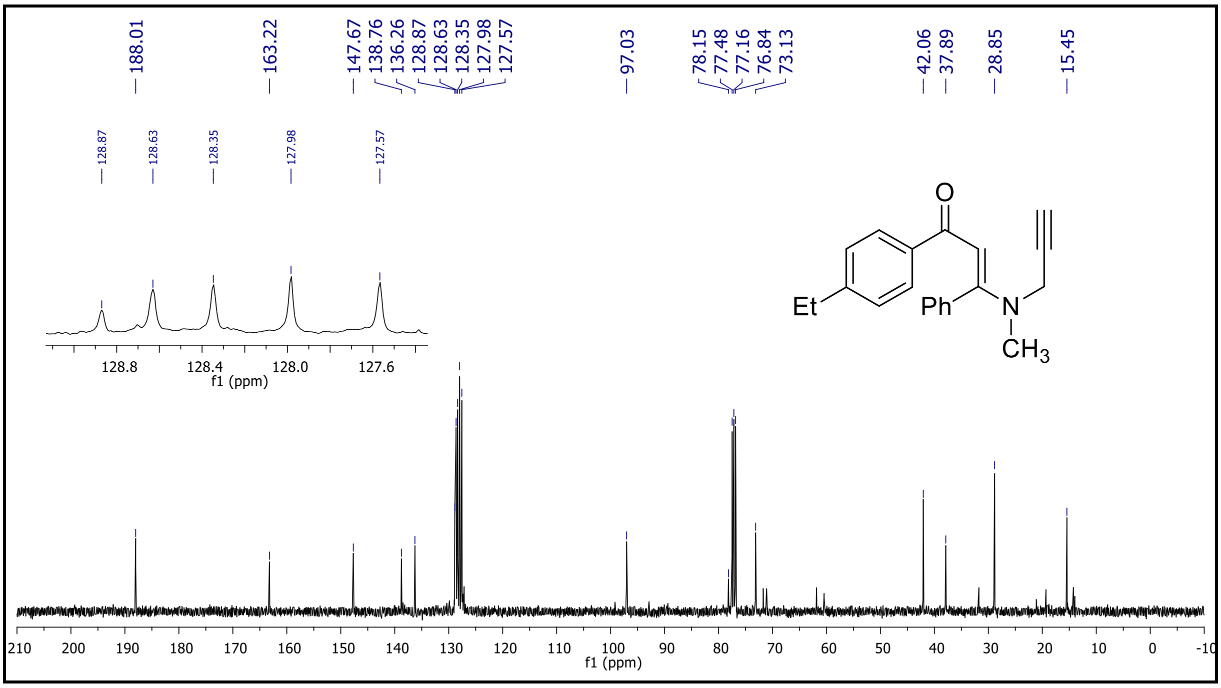

Figure S31. $^{13}C\{^1H\}$ NMR (100 MHz, $CDCl_3$) spectrum of compound **3o**.

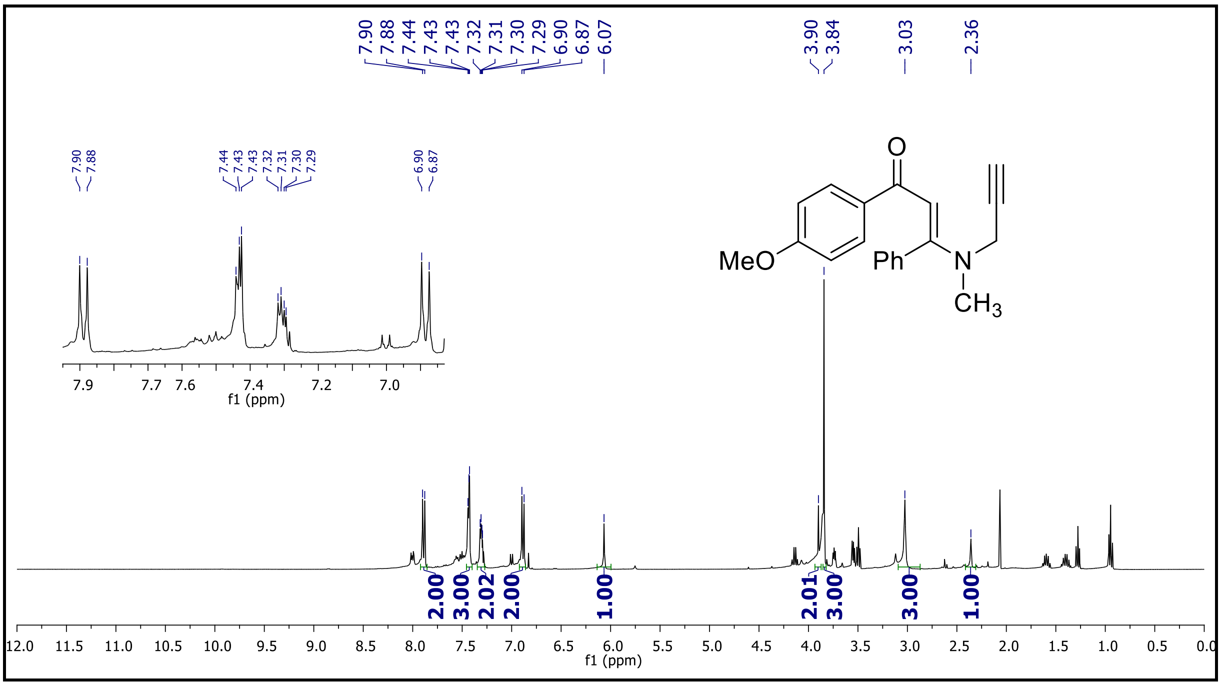

Figure S32. ^1H NMR (400 MHz, $CDCl_3$) spectrum of compound **3p**.

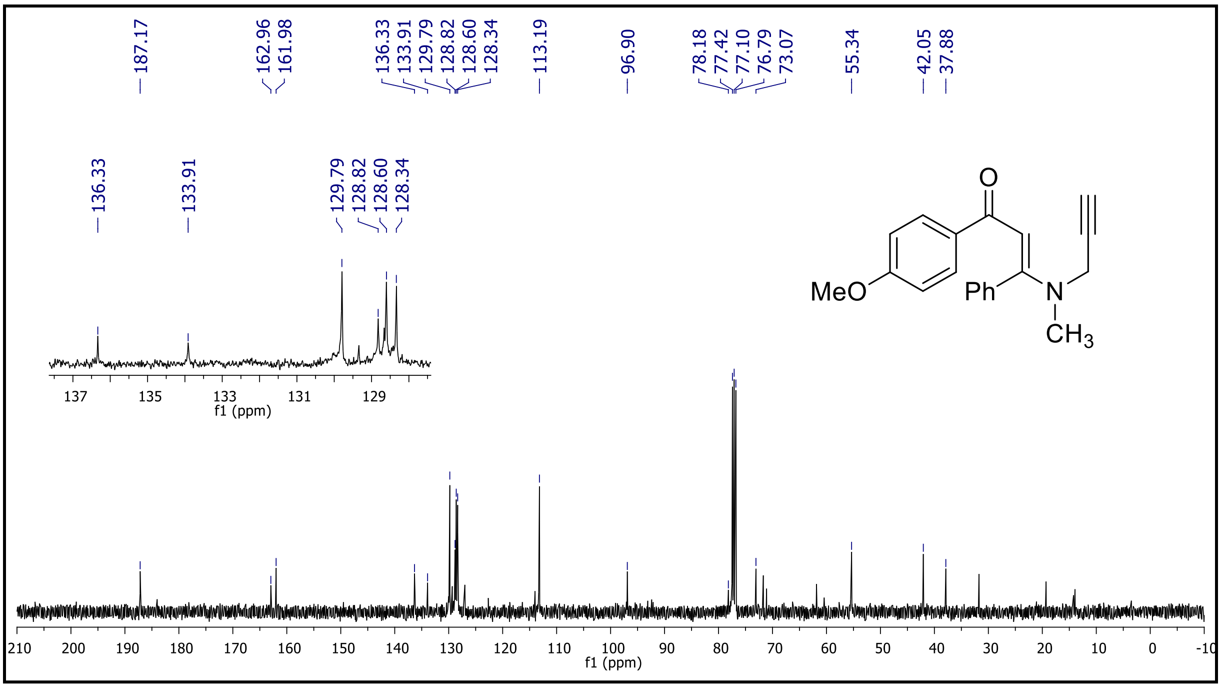

Figure S33. $^{13}C\{^{1}H\}$ NMR (100 MHz, $CDCl_3$) spectrum of compound **3p**.

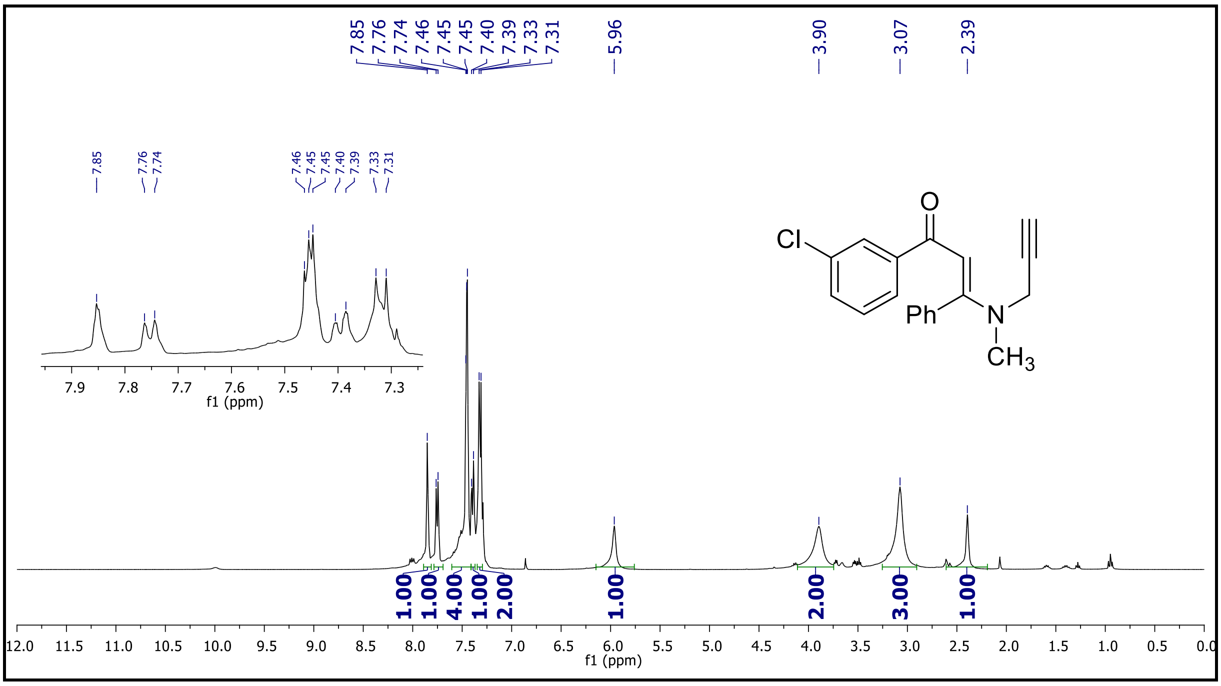

Figure S34. ^{1}H NMR (400 MHz, $CDCl_3$) spectrum of compound**3q** .

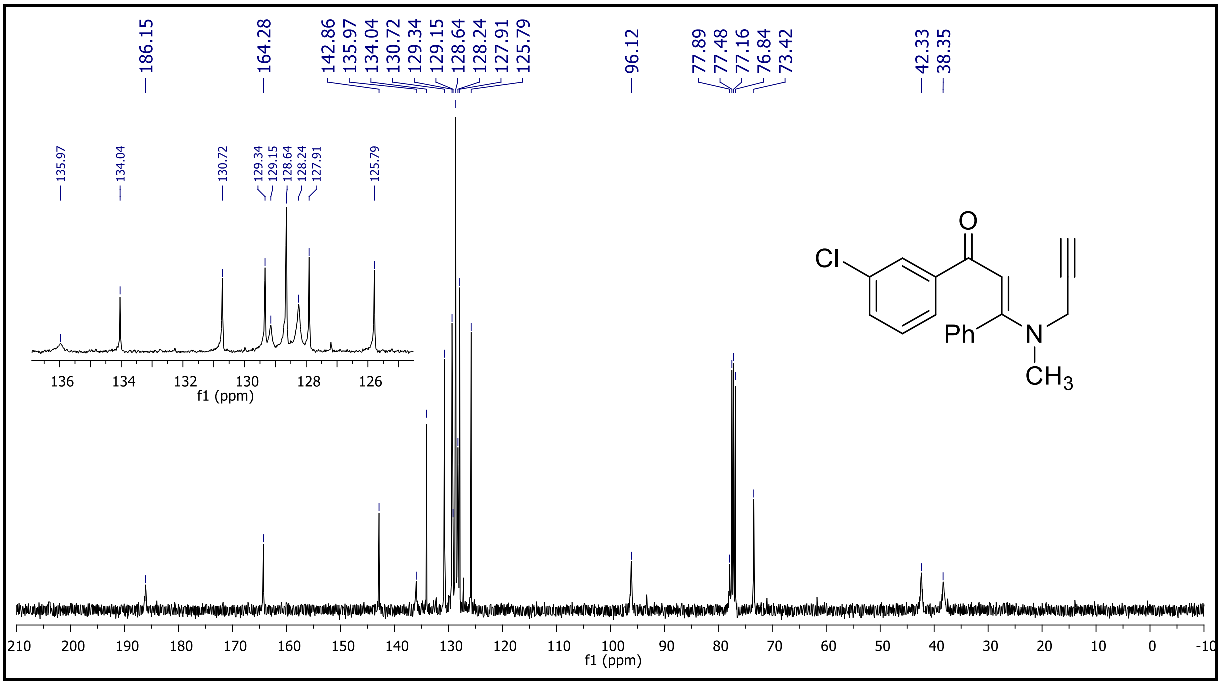

Figure S35. $^{13}C\{^{1}H\}$ NMR (100 MHz, $CDCl_3$) spectrum of compound **3q**.

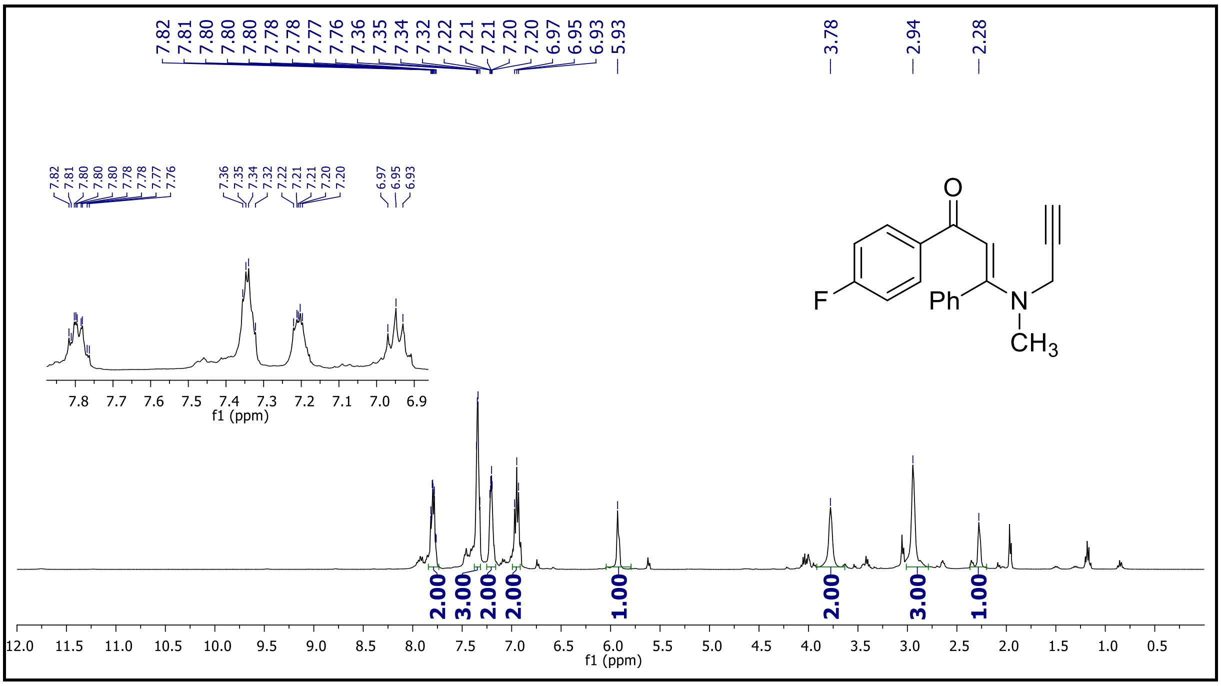

Figure S36. ^{1}H NMR (400 MHz, $CDCl_3$) spectrum of compound **3r**.

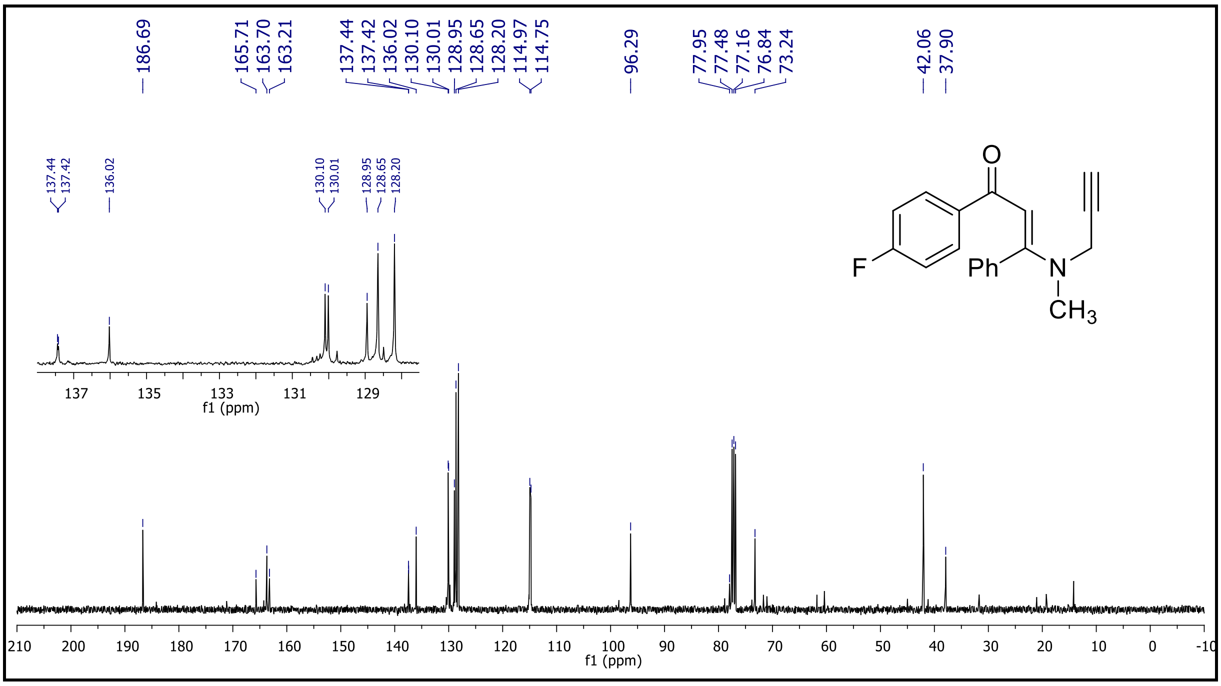

Figure S37. ^{13}C{^{1}H} NMR (100 MHz, $CDCl_3$) spectrum of compound **3r**.

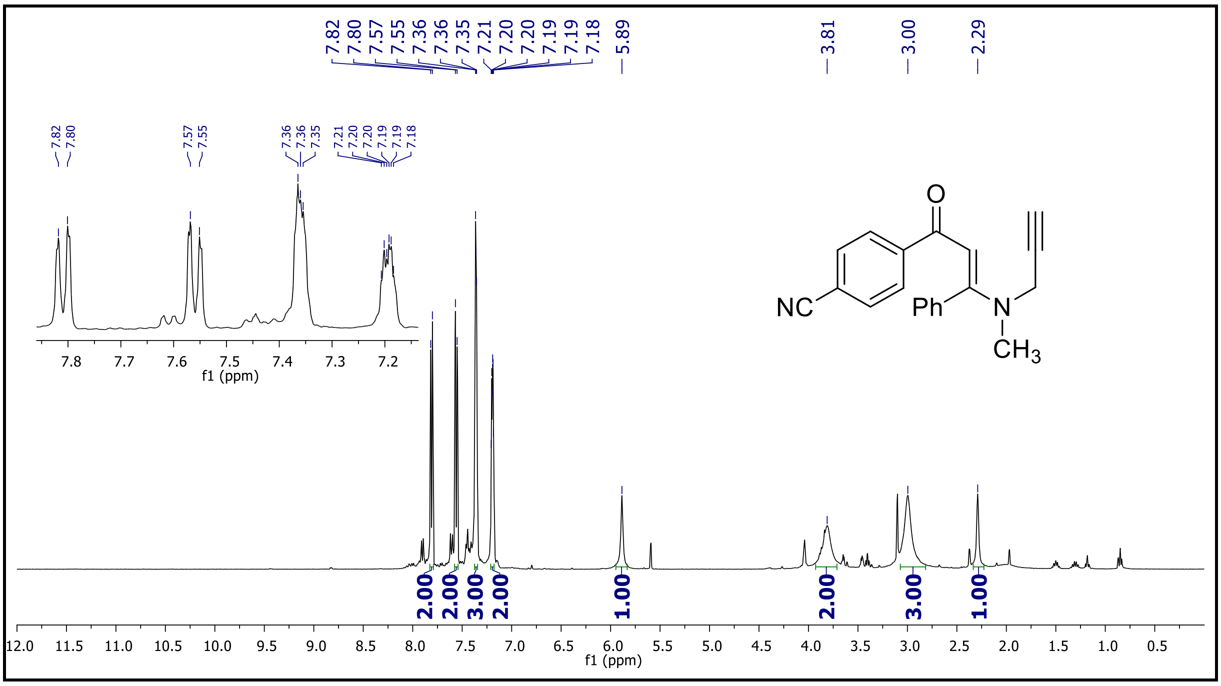

Figure S38. ^{1}H NMR (400 MHz, $CDCl_3$) spectrum of compound **3s**.

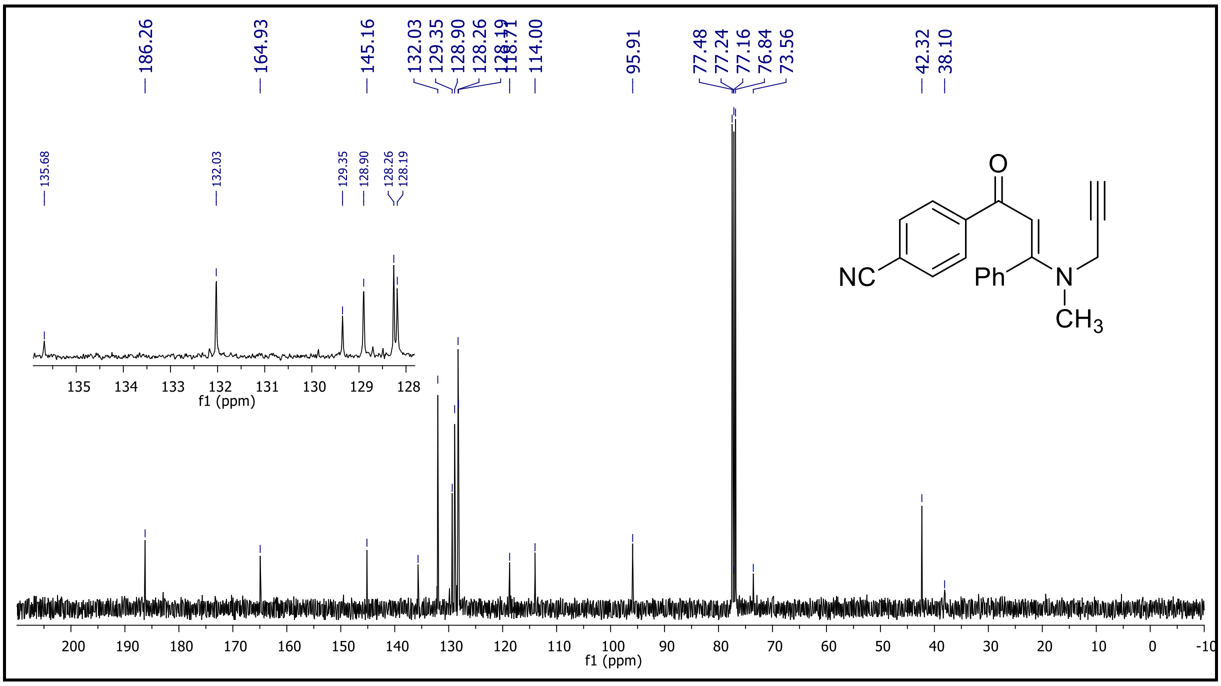

Figure S39. $^{13}C\{^{1}H\}$ NMR (100 MHz, $CDCl_3$) spectrum of compound **3s**.

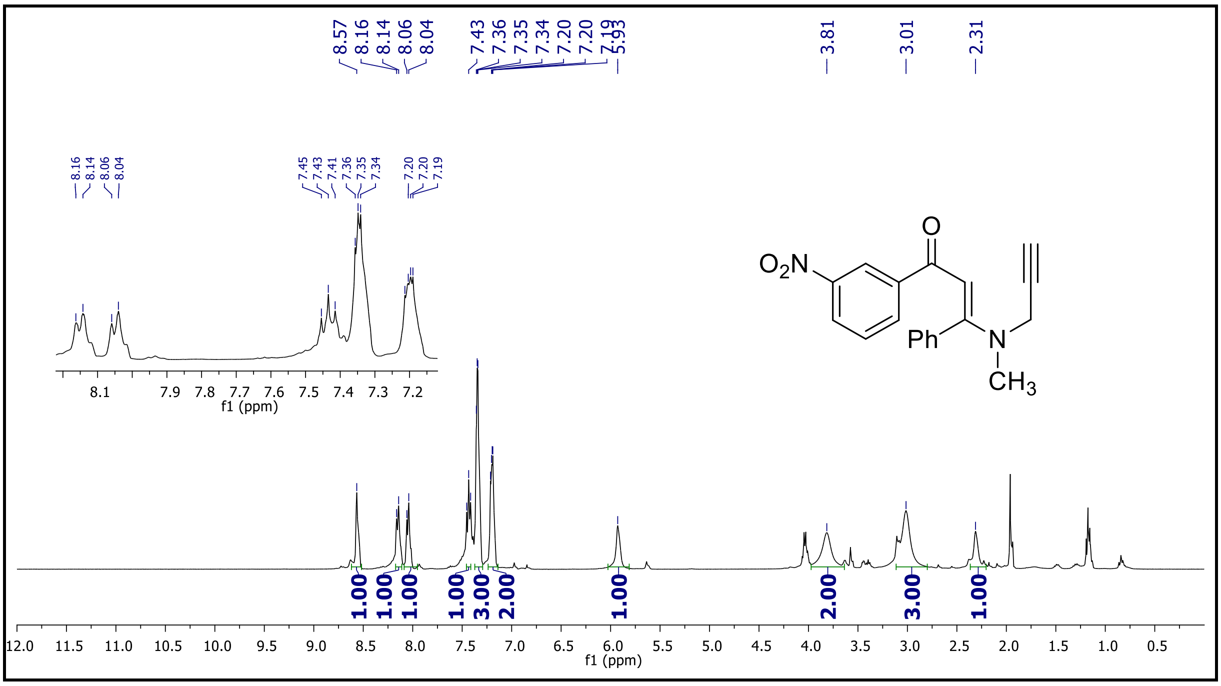

Figure S40. ^{1}H NMR (400 MHz, $CDCl_3$) spectrum of compound **3t**.

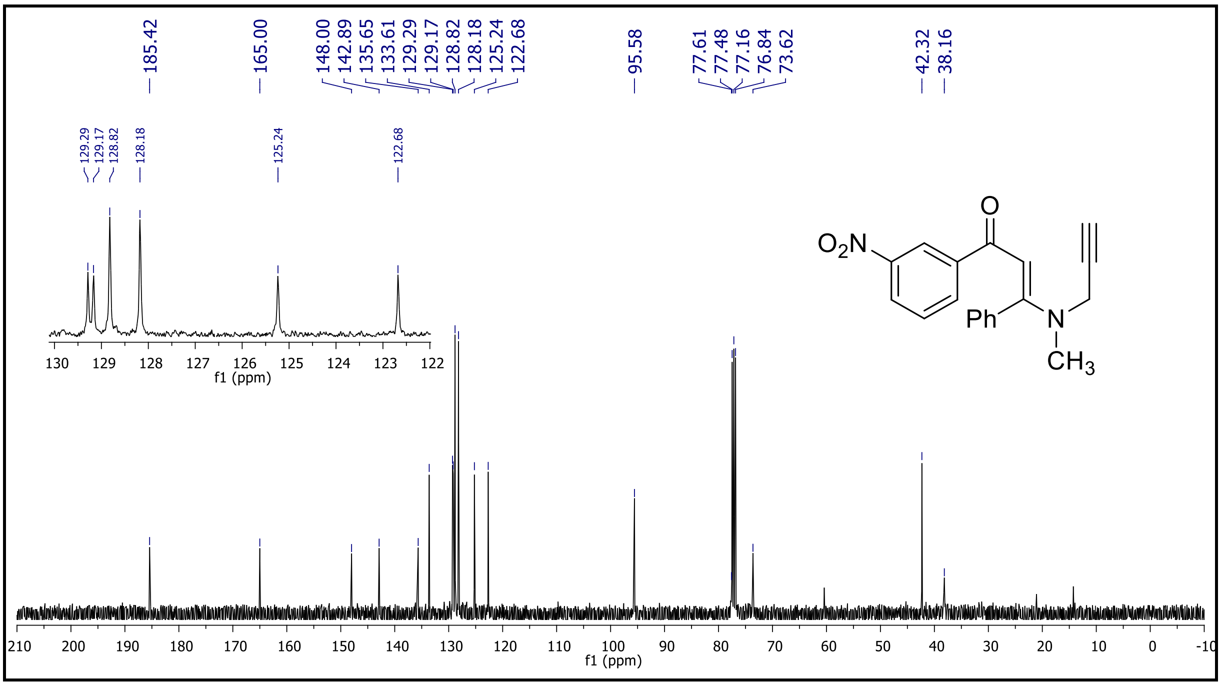

Figure S41. ^{13}C{1H} NMR (100 MHz, $CDCl_3$) spectrum of compound **3t**.

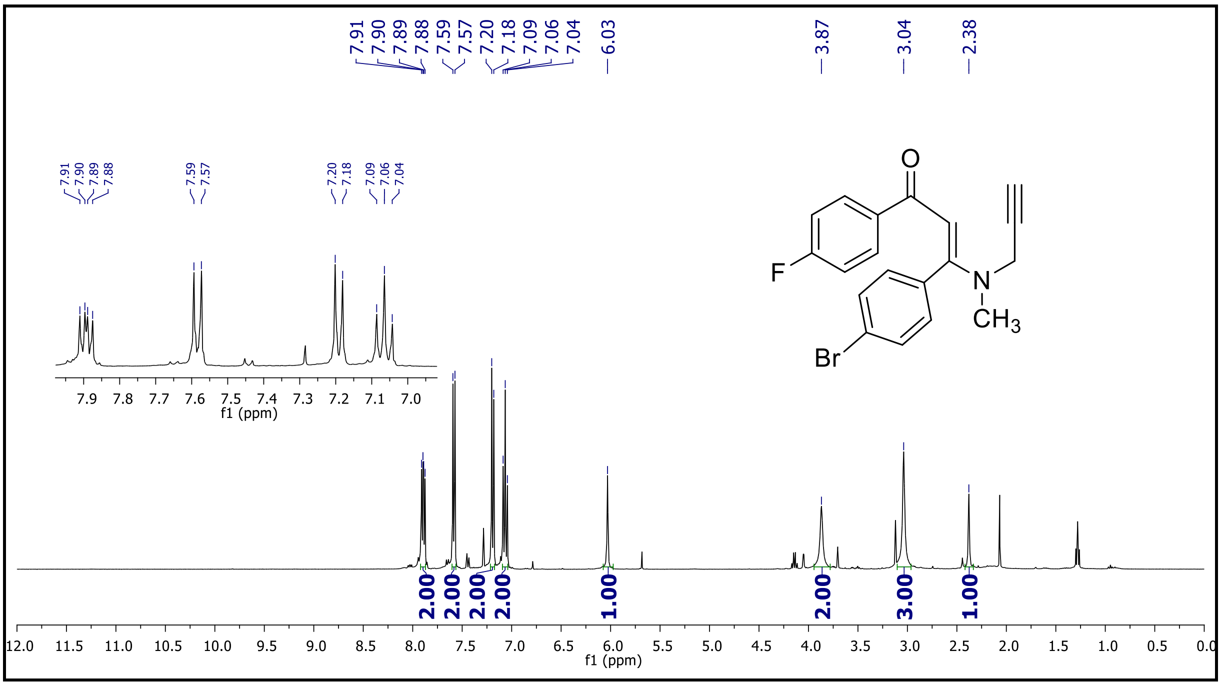

Figure S42. ^{1}H NMR (400 MHz, $CDCl_3$) spectrum of compound **3u**.

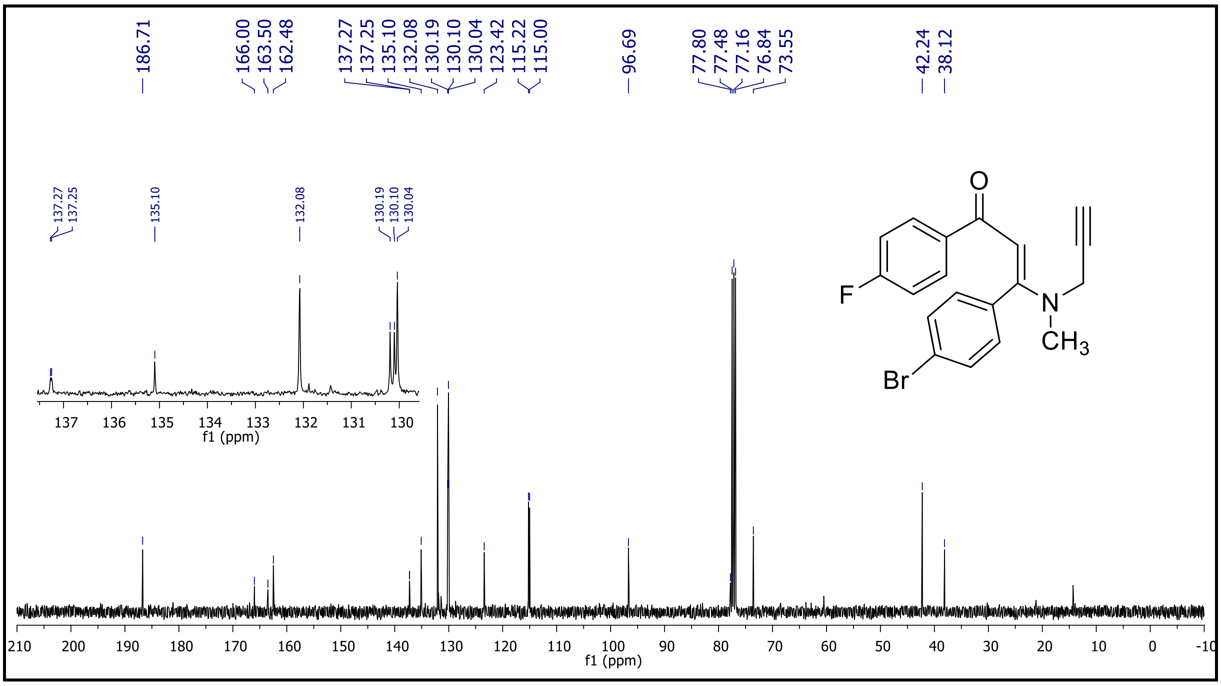

Figure S43. $^{13}C\{^{1}H\}$ NMR (100 MHz, $CDCl_3$) spectrum of compound **3u**.

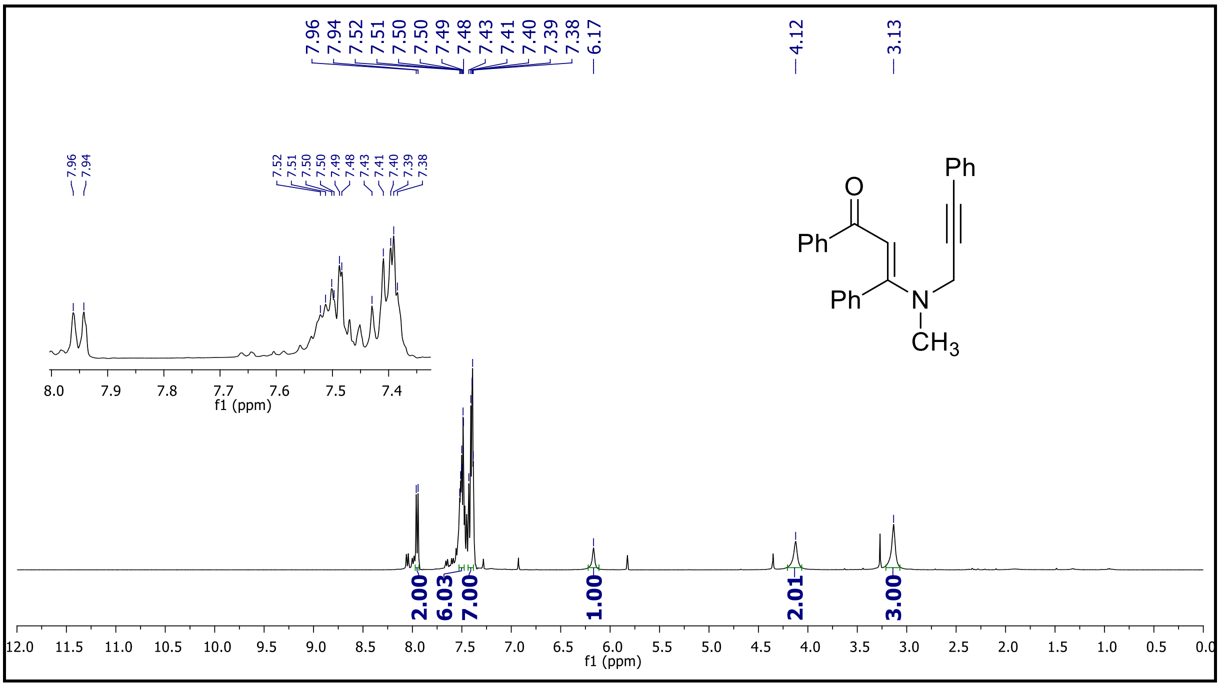

Figure 44. ^{1}H NMR (400 MHz, $CDCl_3$) spectrum of compound **3v**.

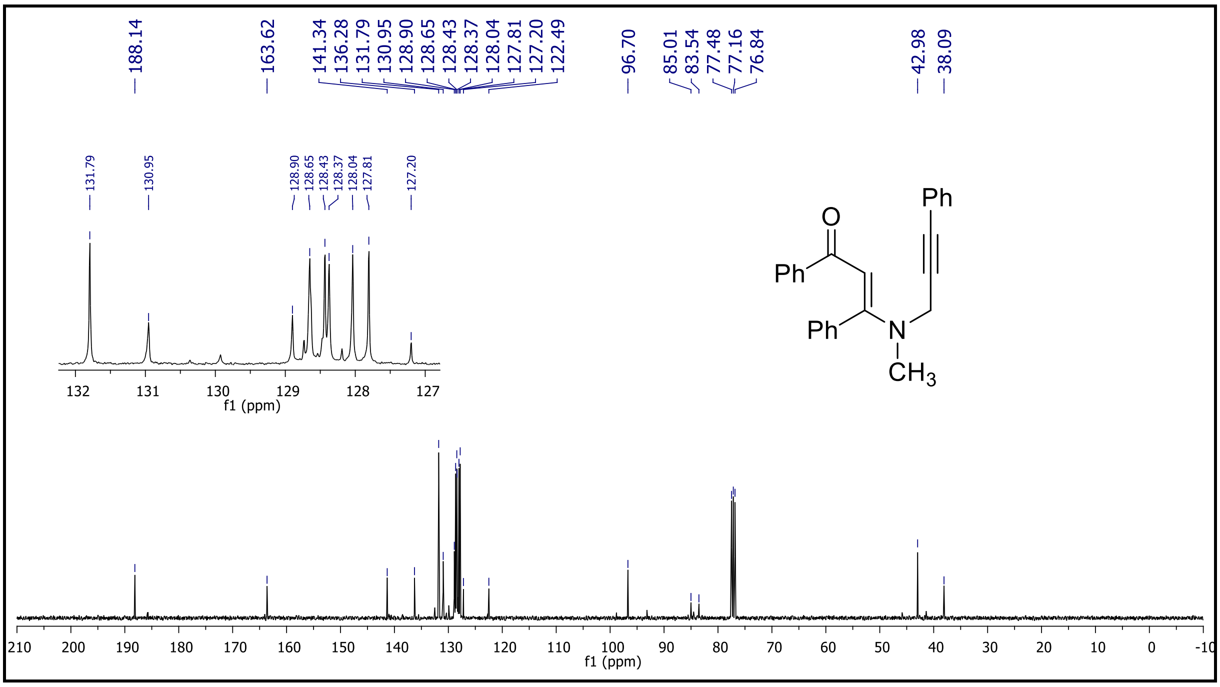

Figure 45. $^{13}C\{^{1}H\}$ NMR (100 MHz, $CDCl_3$) spectrum of compound **3v**.

Copies of ^{1}H and $^{13}C\{^{1}H\}$ NMR Spectra for 1,2,3,4-Tetrasubstituted Benzenes 5 ve 5'

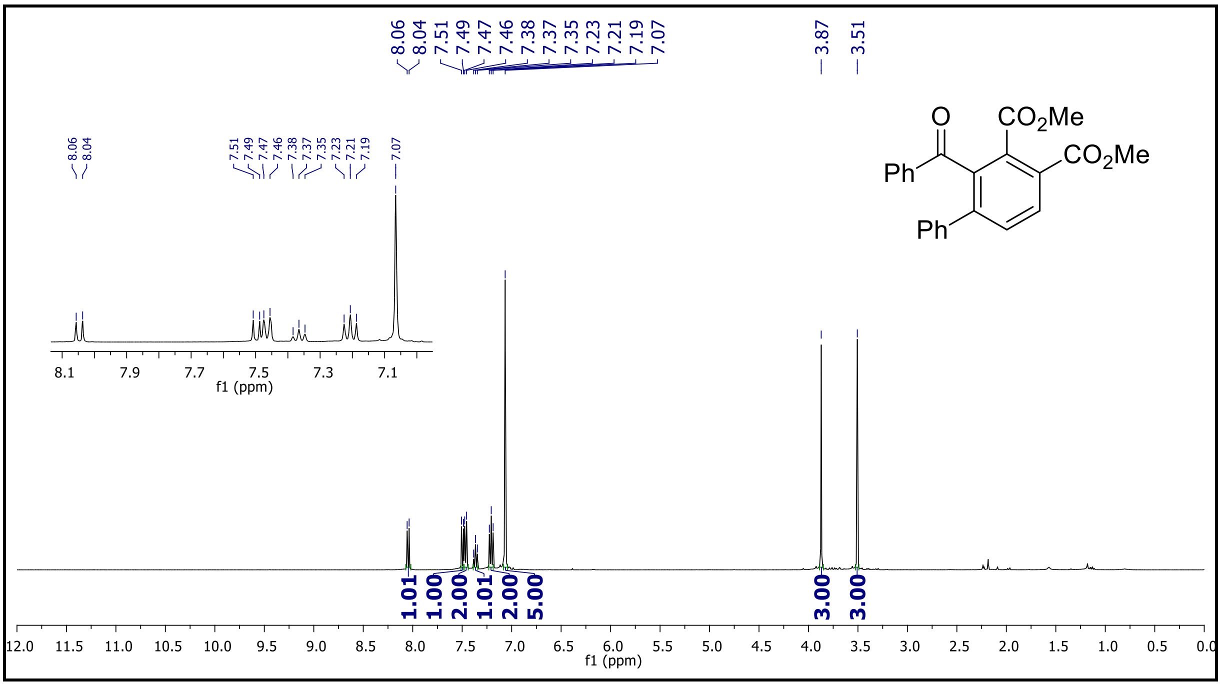

Figure S46. ^{1}H NMR (400 MHz, $CDCl_3$) spectrum of compound **5a**.

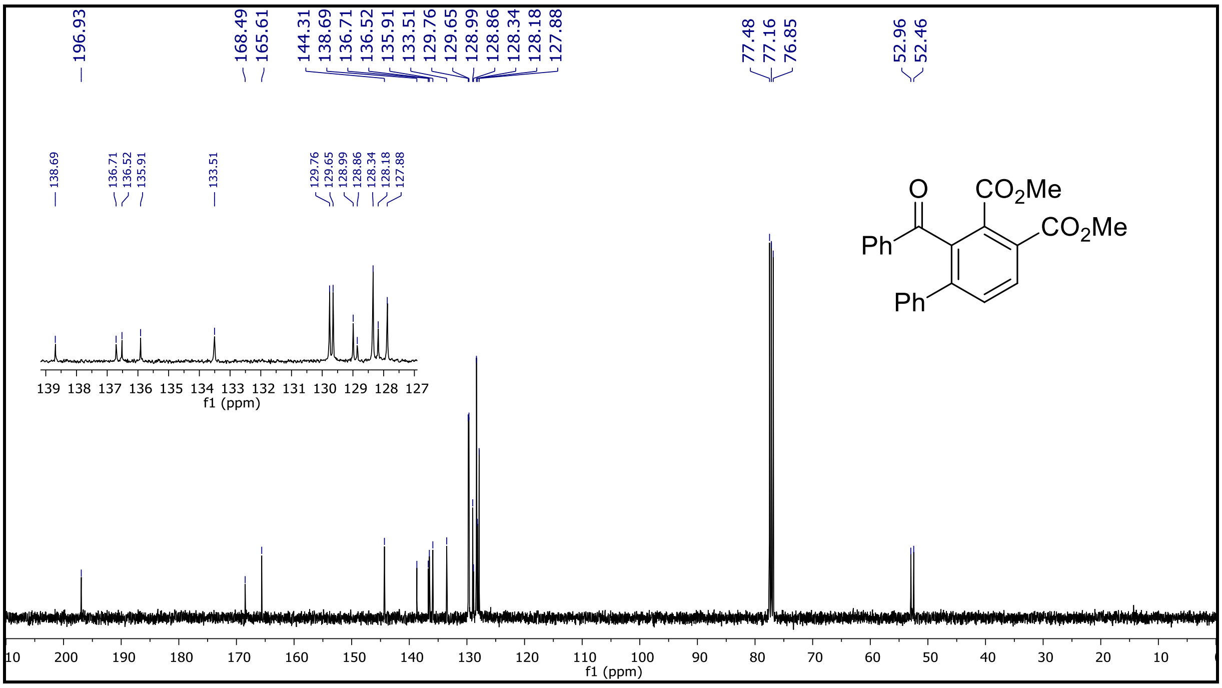

Figure S47. $^{13}C\{^{1}H\}$ NMR (100 MHz, $CDCl_3$) spectrum of compound **5a**.

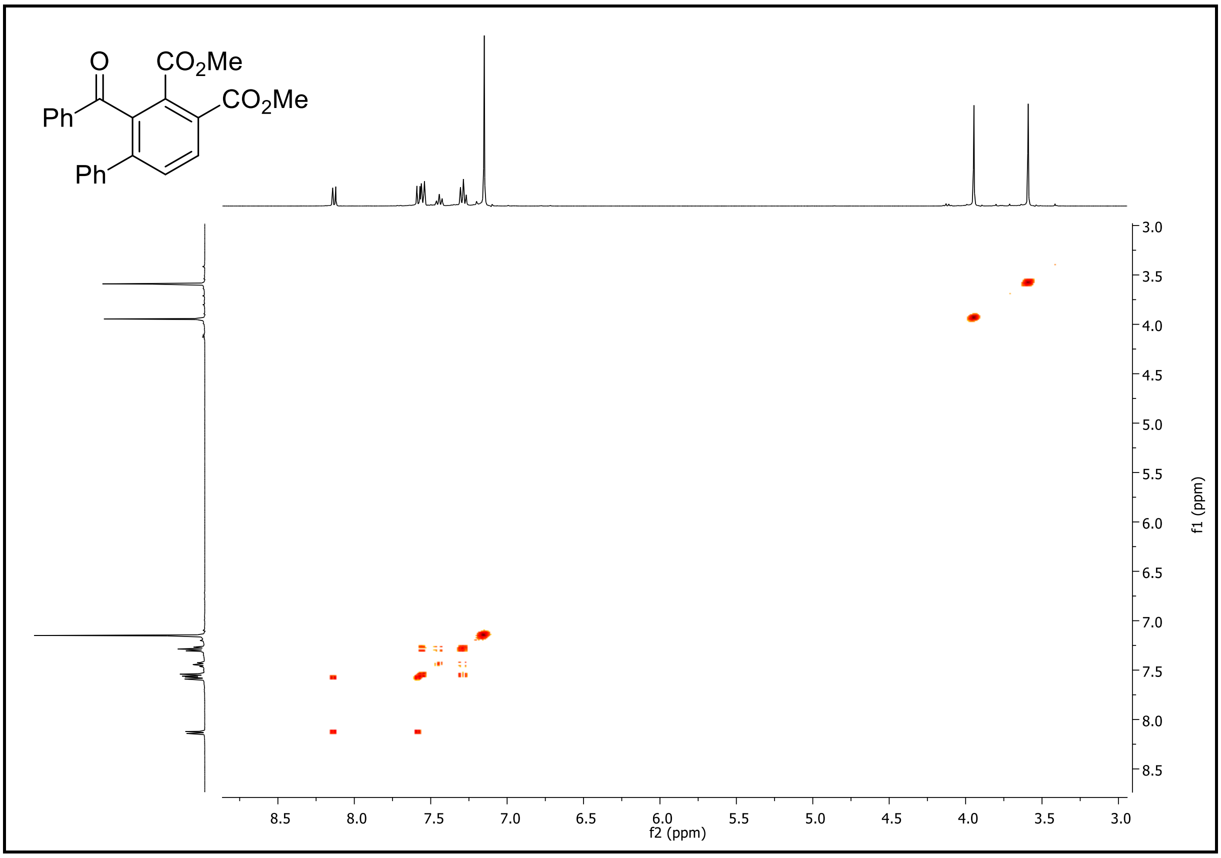

Figure S48. $^{1}H/^{1}H$ COSY (400/400 MHz, $CDCl_3$) spectrum of compound **5a**.

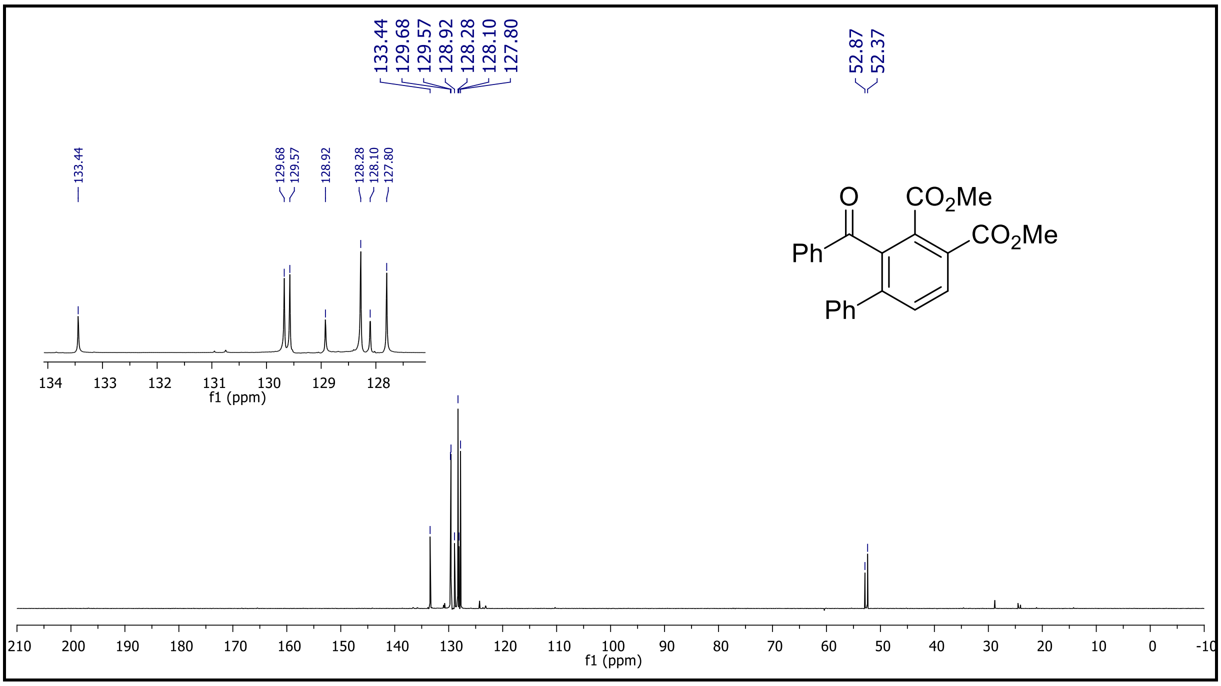

Figure S49. $^{13}C\{^{1}H\}$ DEPT–135 (100 MHz, $CDCl_3$) spectrum of compound **5a**.

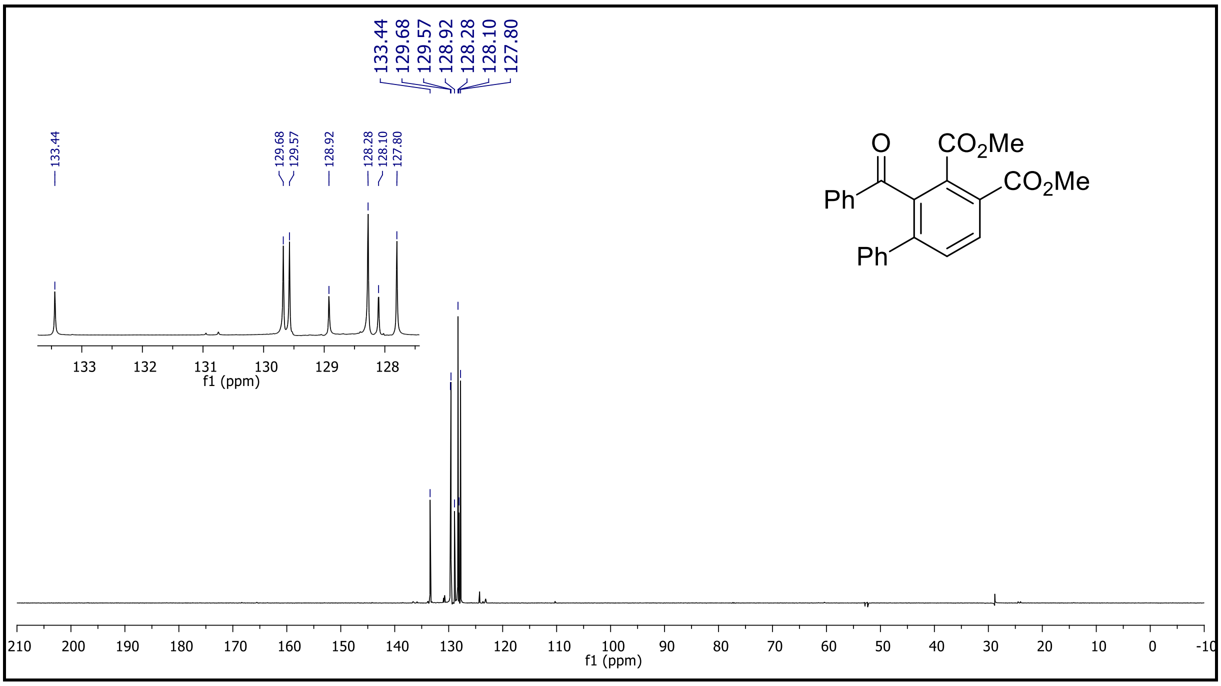

Figure S50. ^{13}C{^{1}H} DEPT–90 (100 MHz, $CDCl_3$) spectrum of compound **5a**.

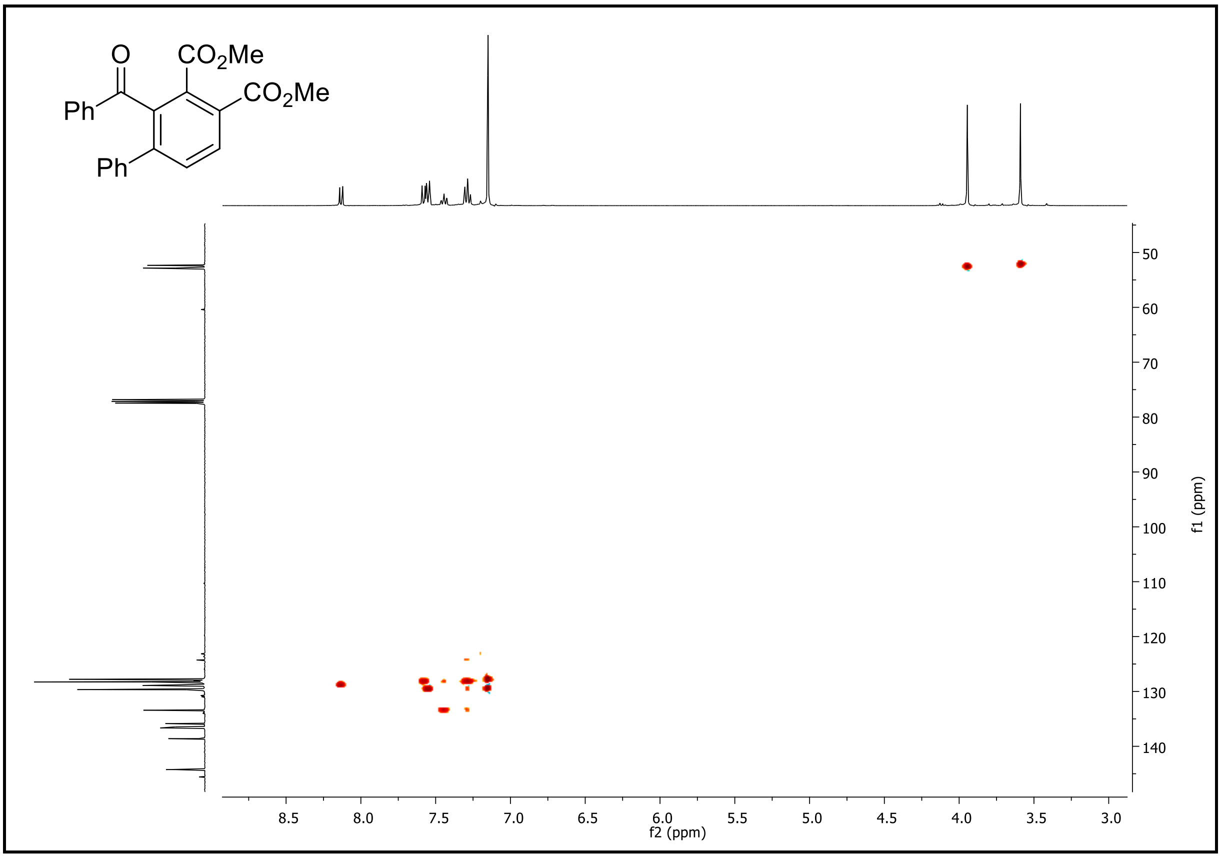

Figure S51. ^{1}H/^{13}C HSQC (400/100 MHz, $CDCl_3$) spectrum of compound **5a**.

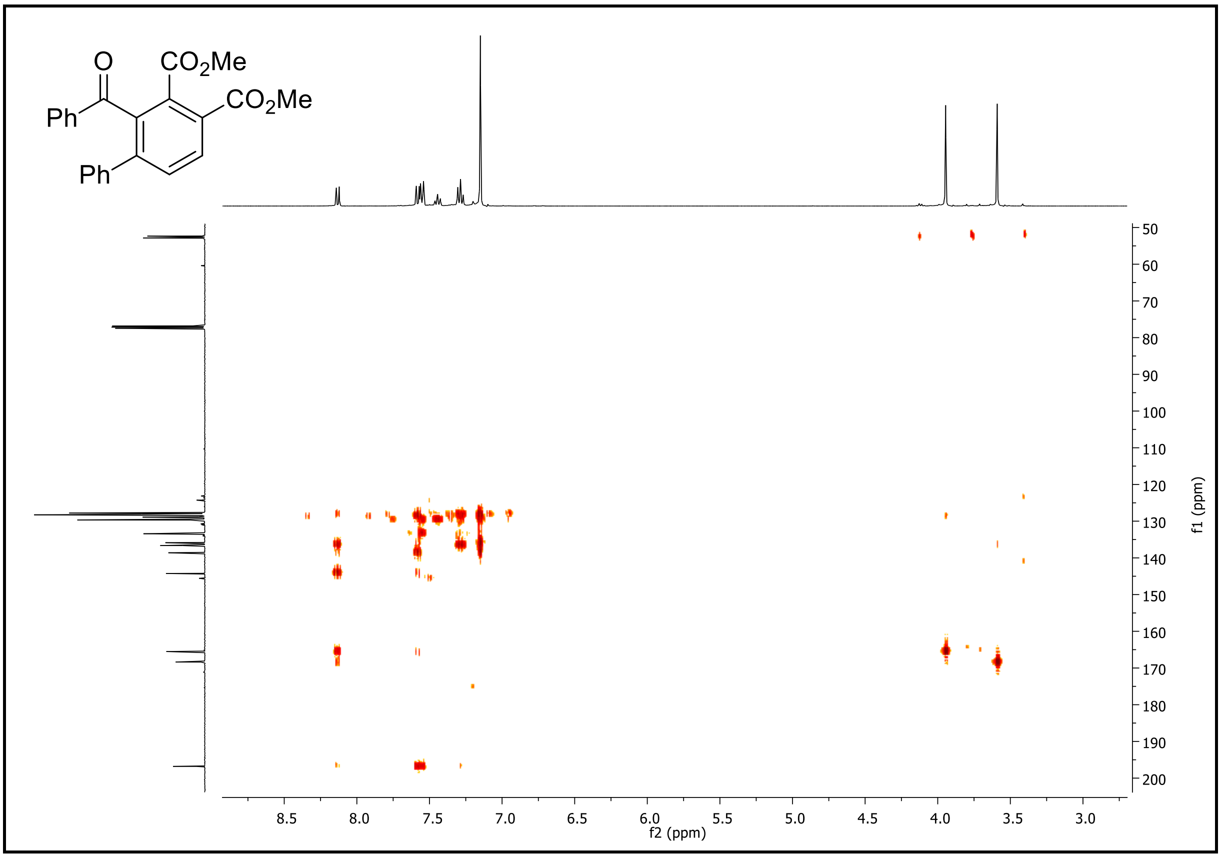

Figure S52. $^{1}H/^{13}C$ HMBC (400/100 MHz, $CDCl_3$) spectrum of compound **5a**.

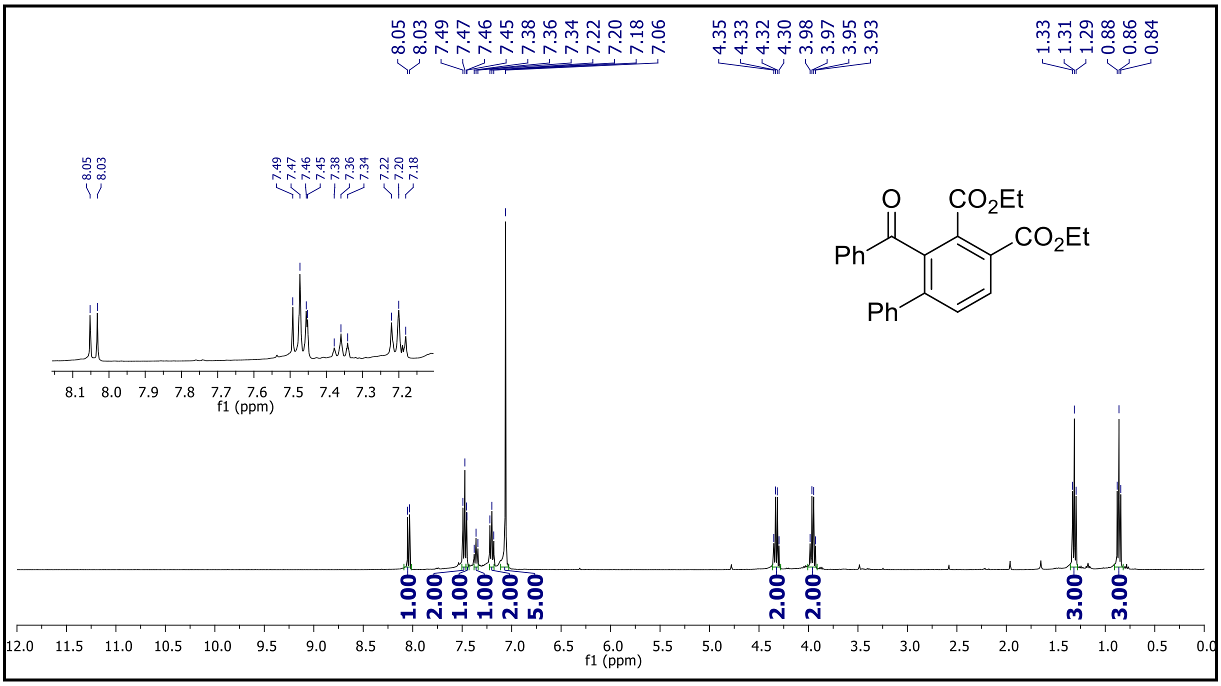

Figure S53. ^{1}H NMR (400 MHz, $CDCl_3$) spectrum of compound **5a'**.

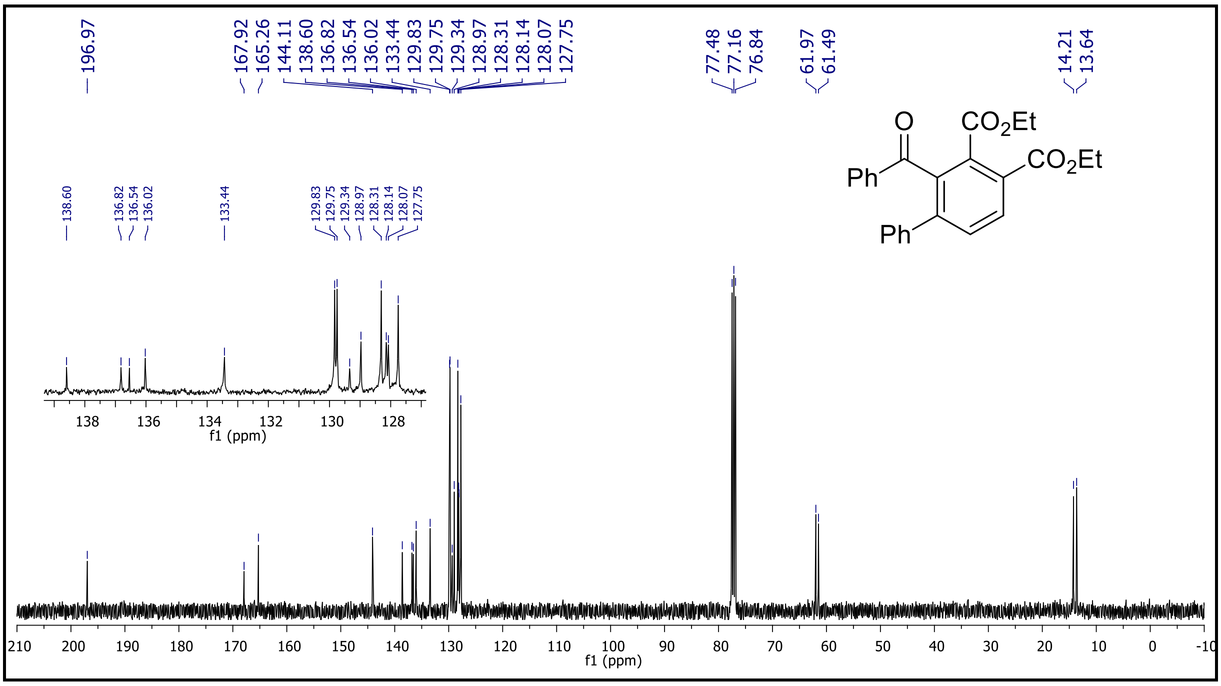

Figure S54. $^{13}C\{^{1}H\}$ NMR (100 MHz, $CDCl_3$) spectrum of compound **5a'**.

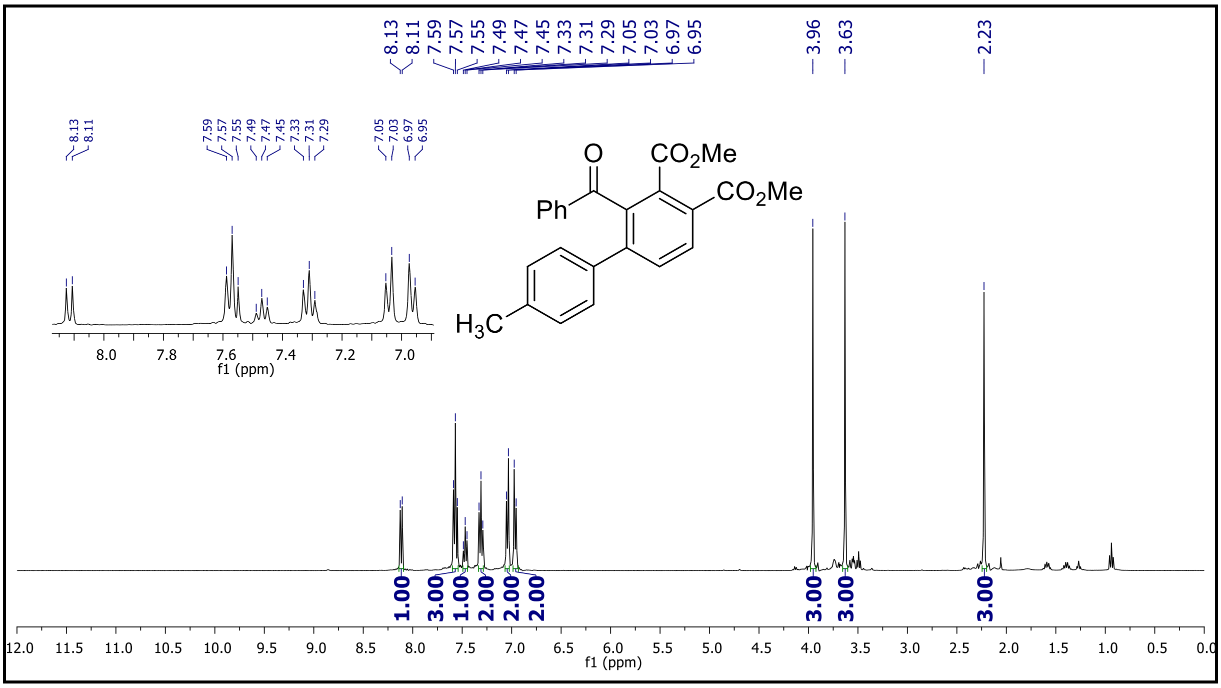

Figure S55. ^{1}H NMR (400 MHz, $CDCl_3$) spectrum of compound **5b**.

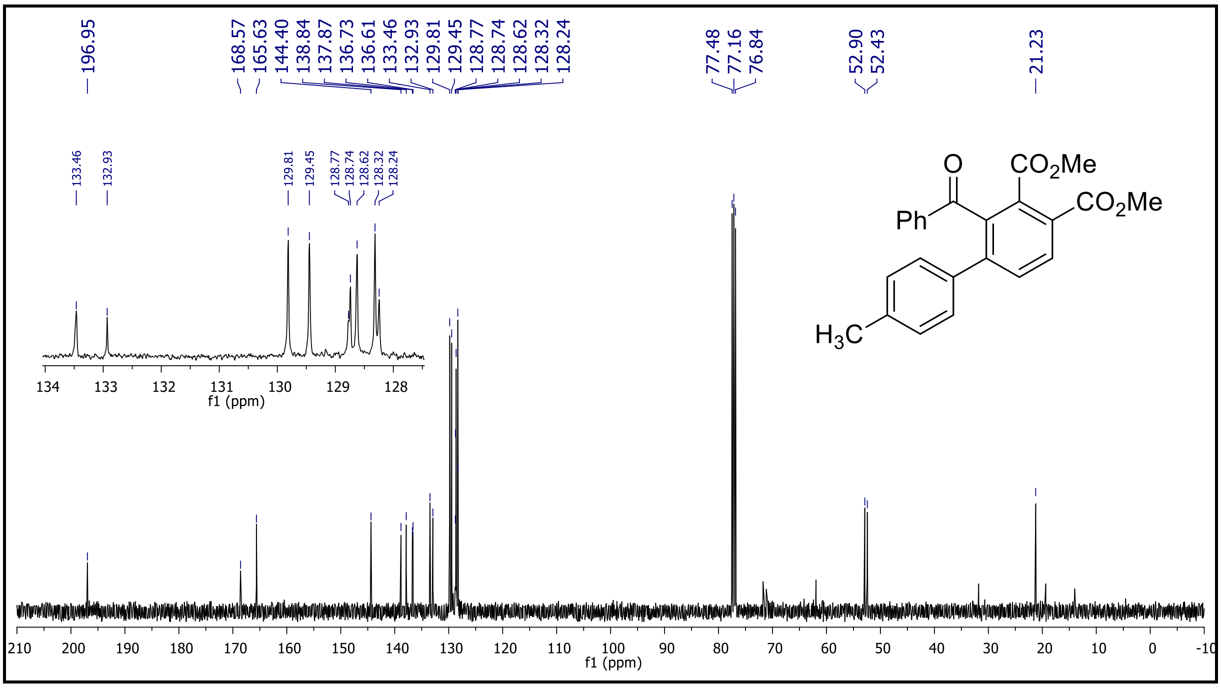

Figure S56. $^{13}C\{^{1}H\}$ NMR (100 MHz, $CDCl_3$) spectrum of compound **5b**.

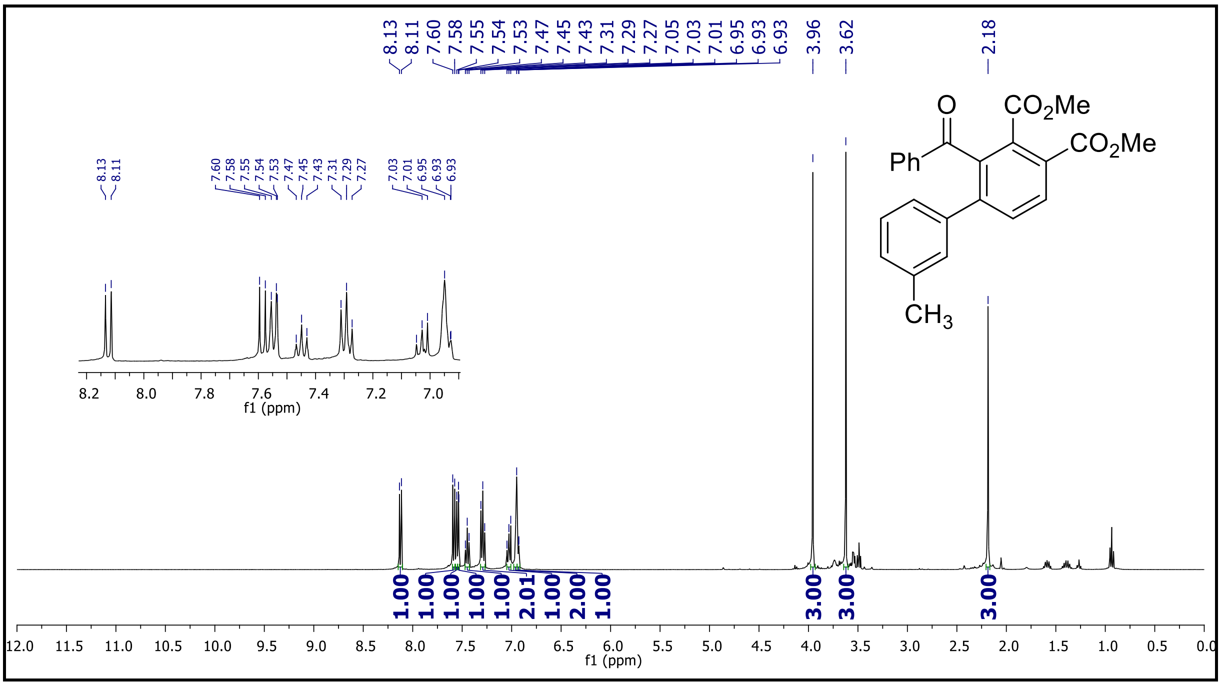

Figure S57. ^{1}H NMR (400 MHz, $CDCl_3$) spectrum of compound **5c**.

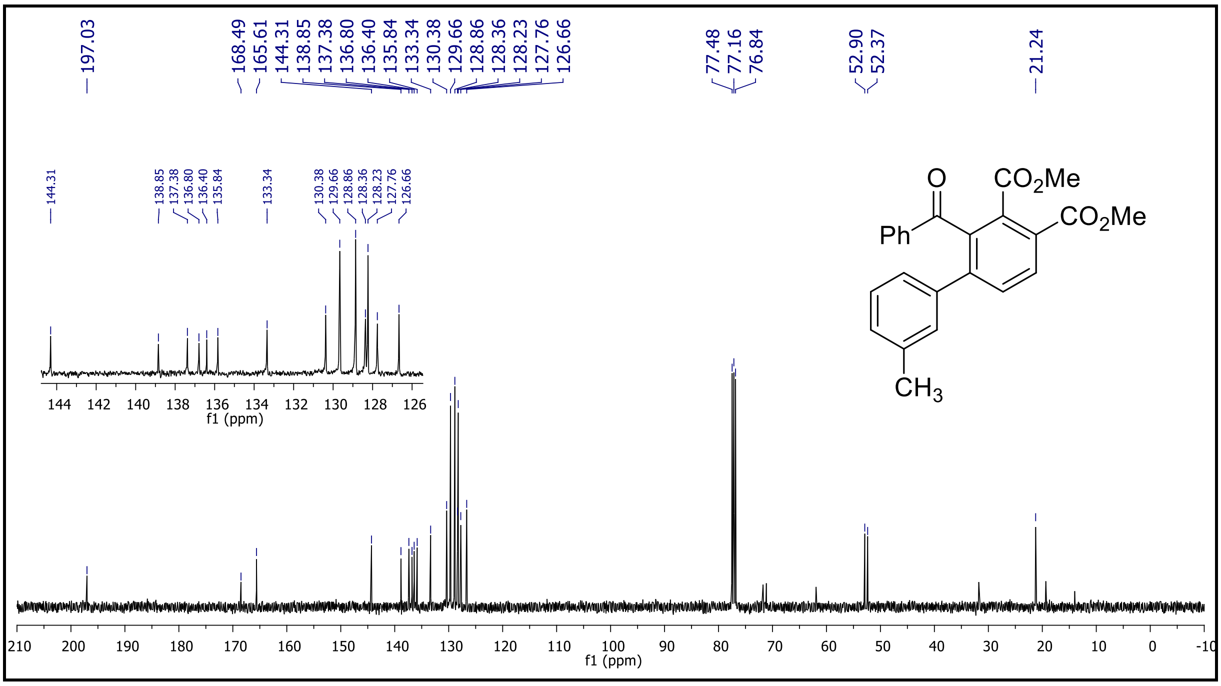

Figure S58. $^{13}C\{^1H\}$ NMR (100 MHz, $CDCl_3$) spectrum of compound **5c**.

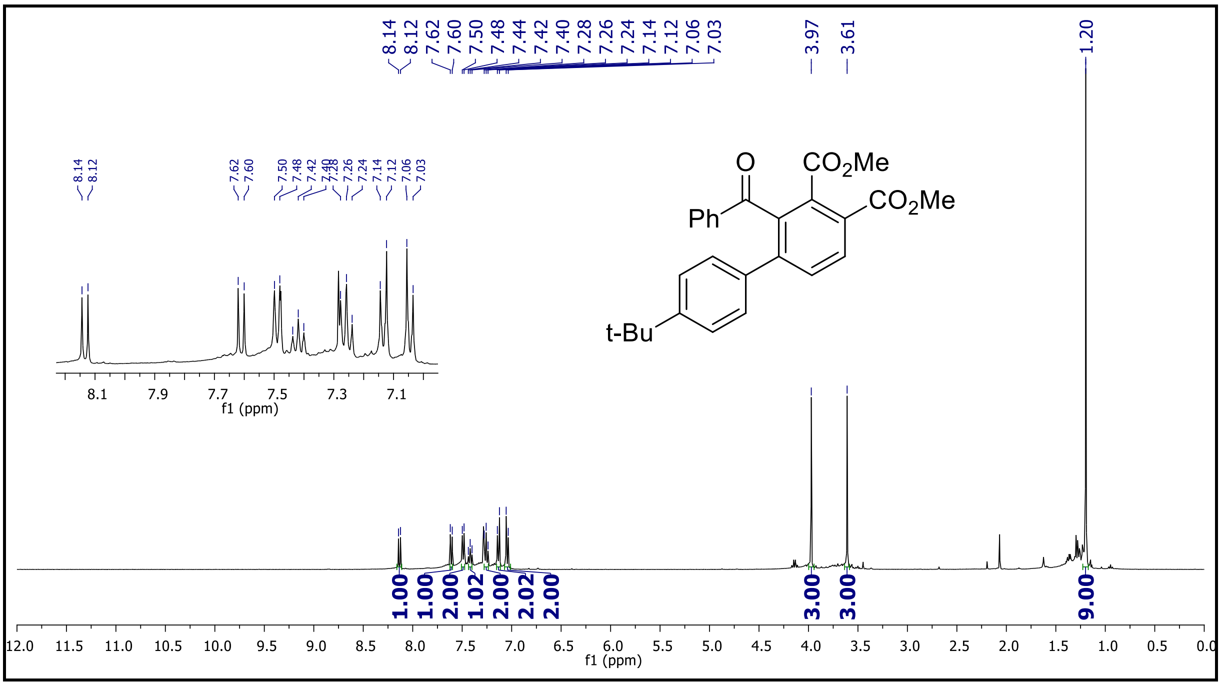

Figure S59. ^1H NMR (400 MHz, $CDCl_3$) spectrum of compound **5d**.

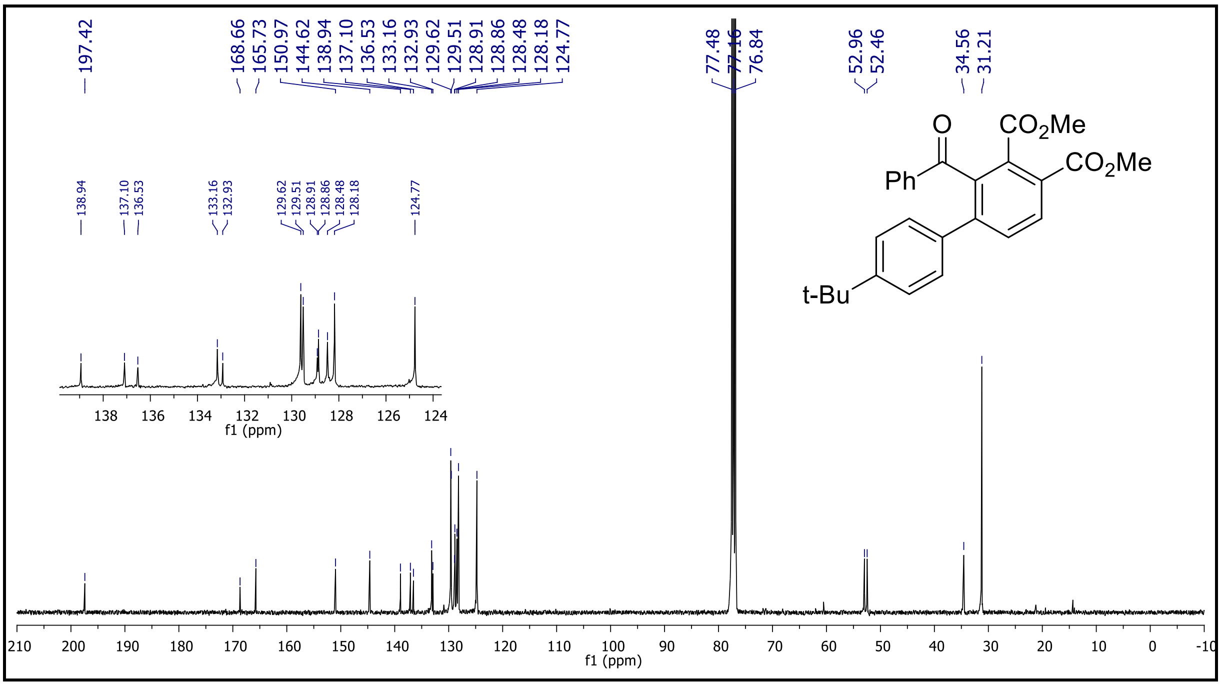

Figure S60. $^{13}C\{^{1}H\}$ NMR (100 MHz, $CDCl_3$) spectrum of compound **5d**.

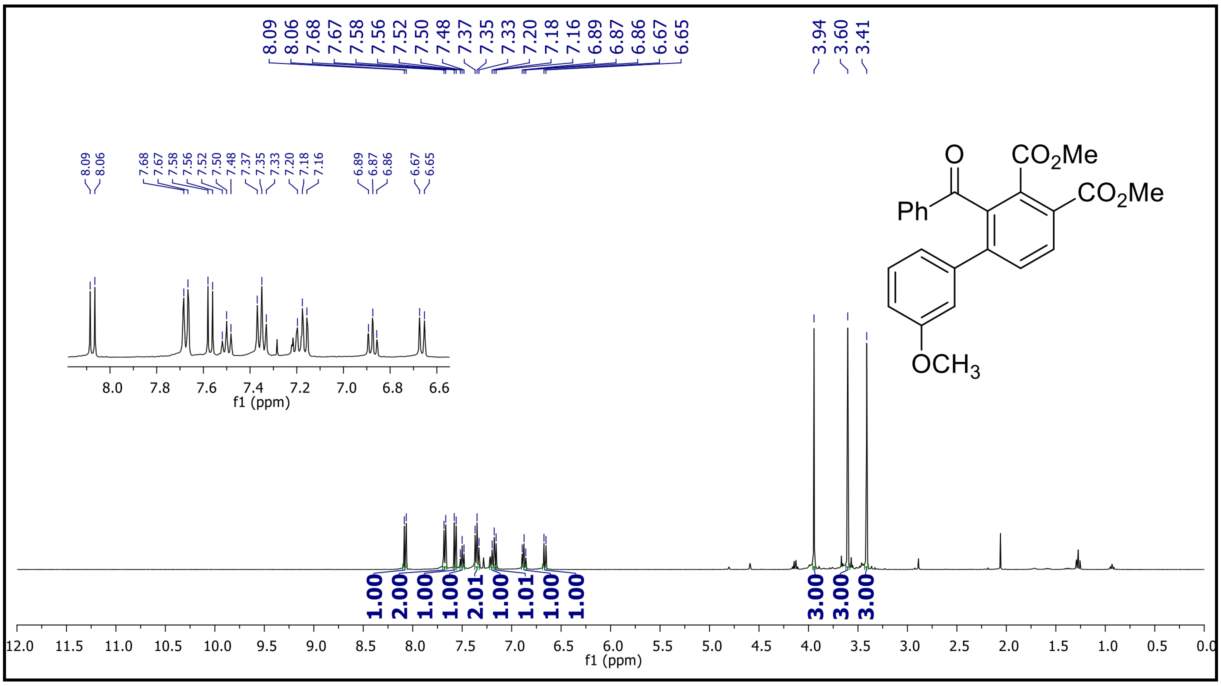

Figure S61. ^{1}H NMR (400 MHz, $CDCl_3$) spectrum of compound **5e**.

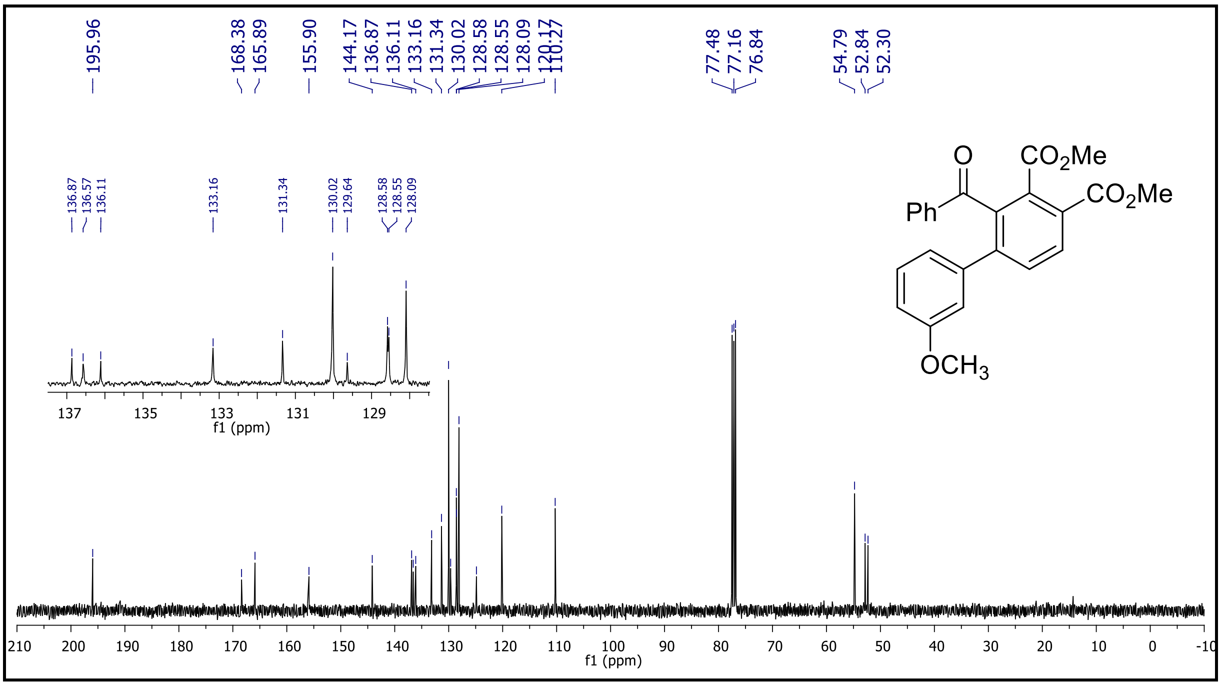

Figure S62. $^{13}C\{^{1}H\}$ NMR (100 MHz, $CDCl_3$) spectrum of compound **5e**.

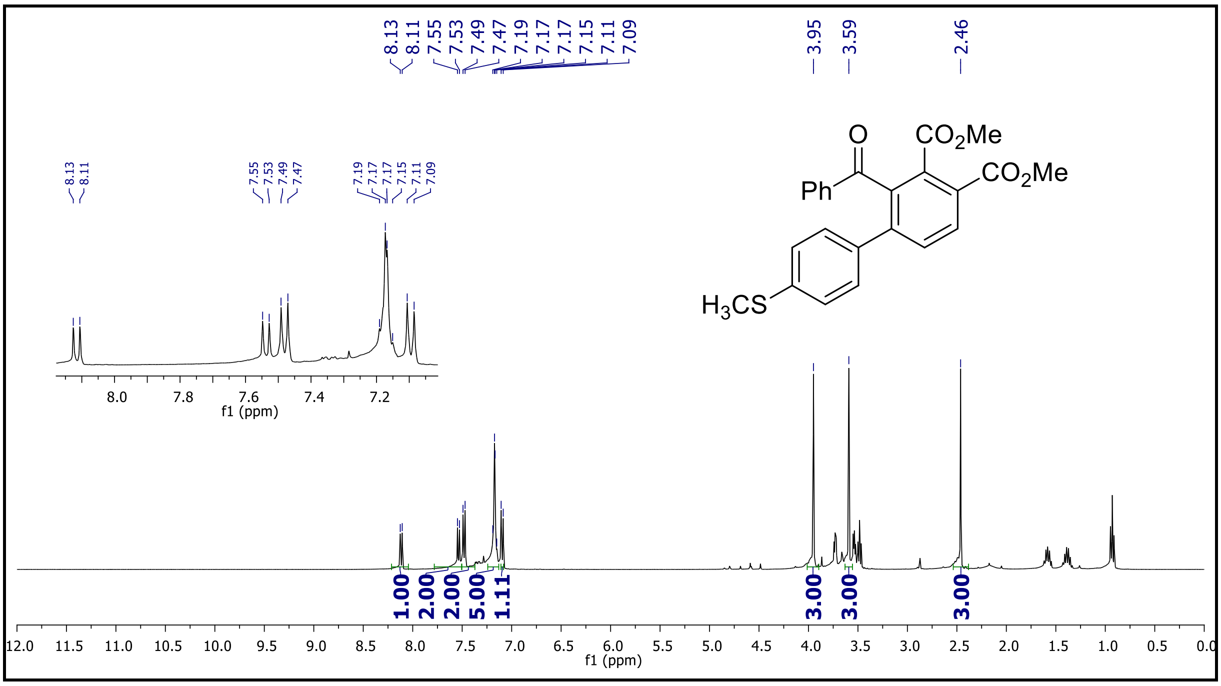

Figure S63. ^{1}H NMR (400 MHz, $CDCl_3$) spectrum of compound **5f**.

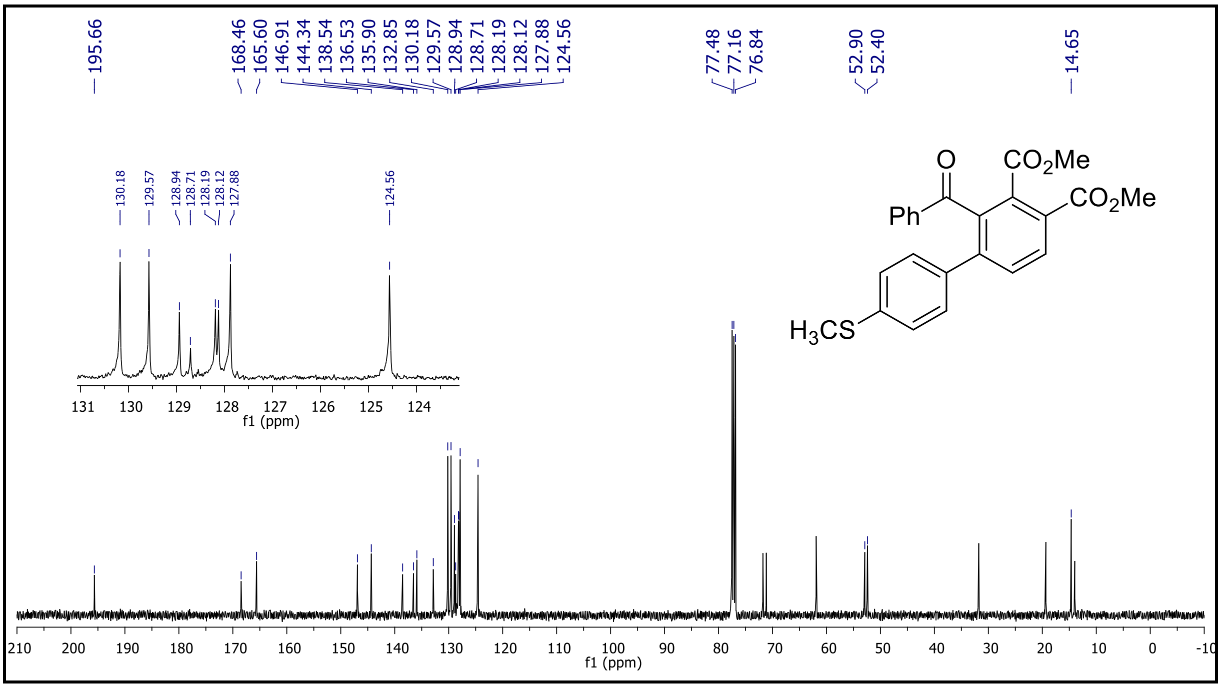

Figure S64. $^{13}C\{^{1}H\}$ NMR (100 MHz, $CDCl_3$) spectrum of compound **5f**.

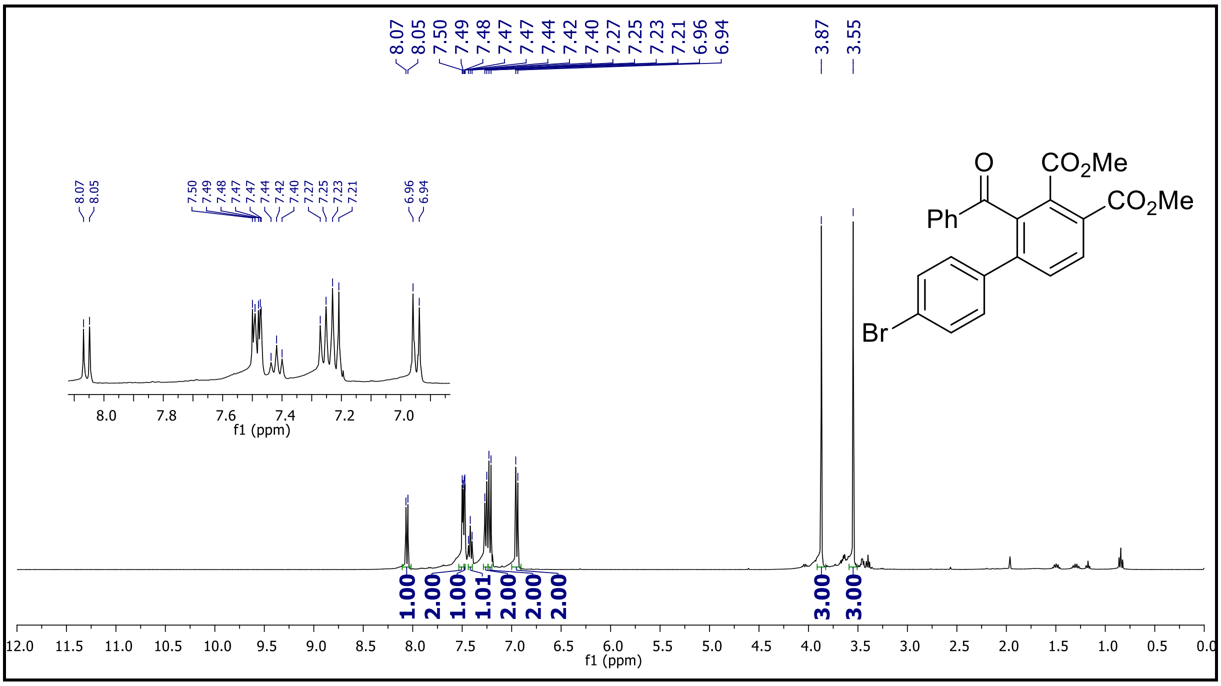

Figure S65. ^{1}H NMR (400 MHz, $CDCl_3$) spectrum of compound **5g**.

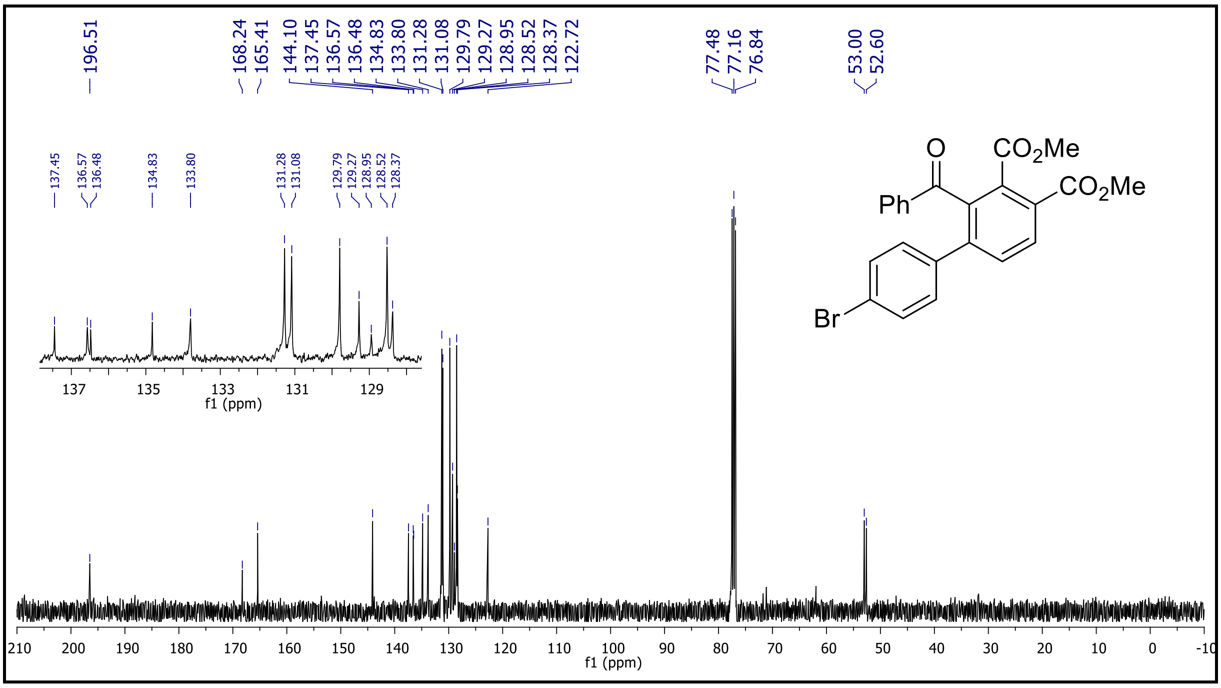

Figure S66. $^{13}C\{^{1}H\}$ NMR (100 MHz, CDCl3) spectrum of compound **5g**.

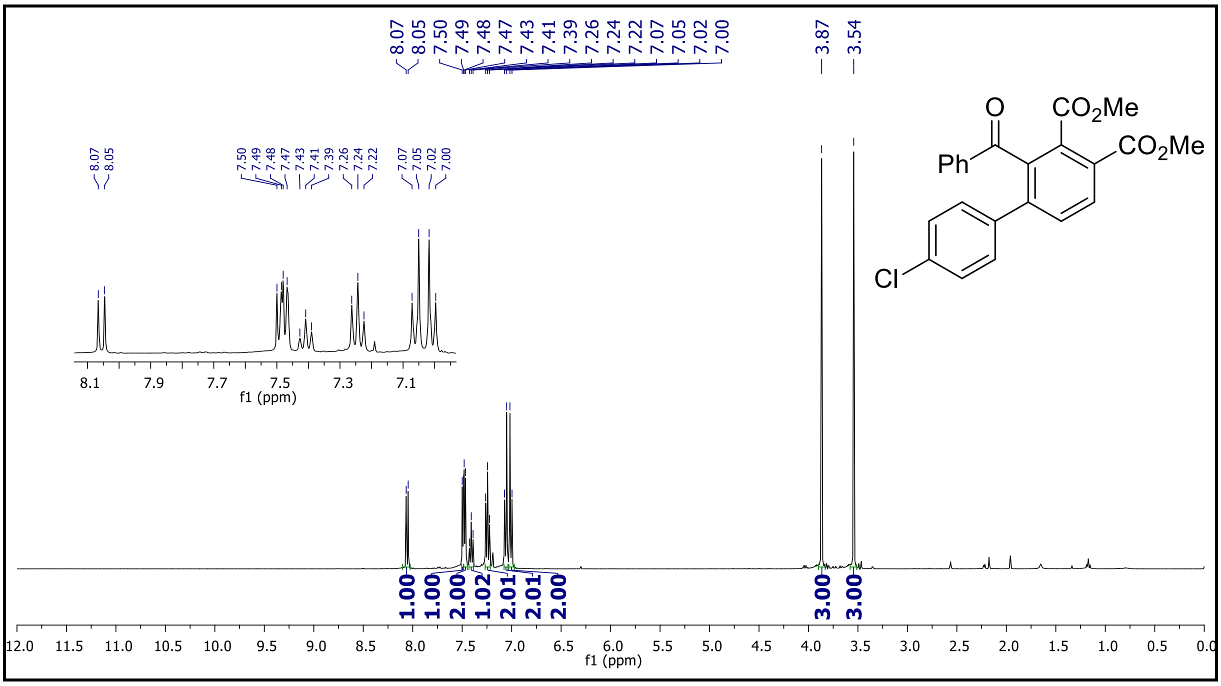

Figure S67. ^{1}H NMR (400 MHz, $CDCl_3$) spectrum of compound **5h**.

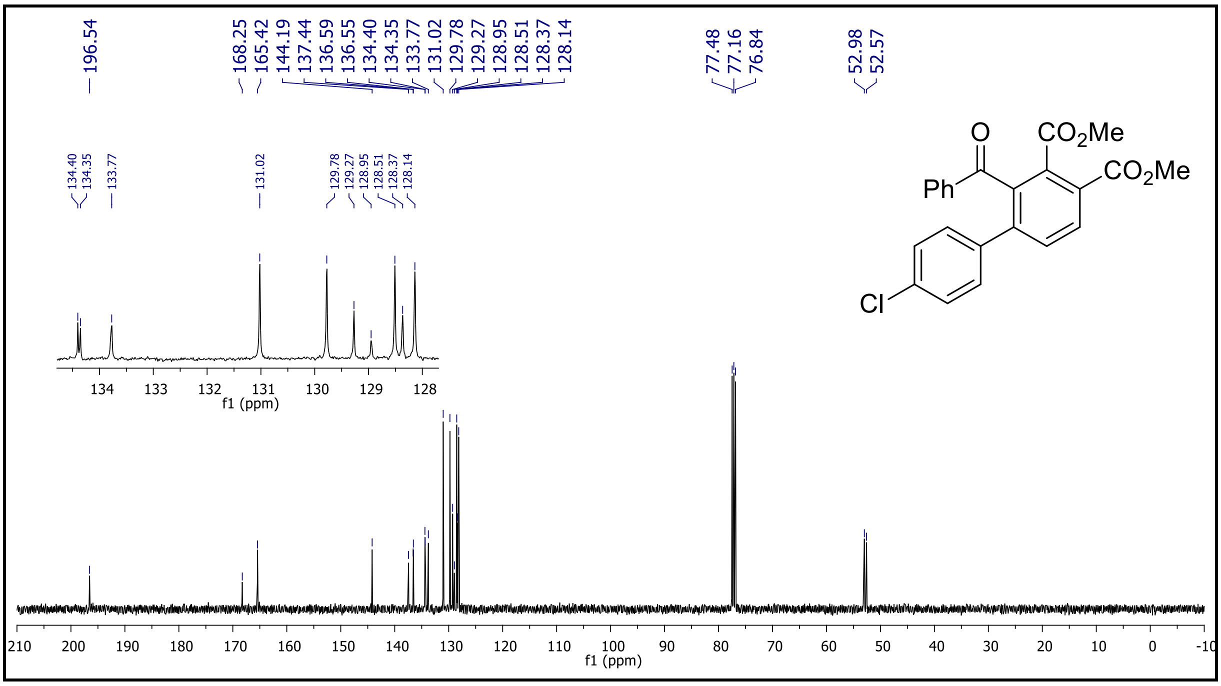

Figure S68. $^{13}C\{^{1}H\}$ NMR (100 MHz, $CDCl_3$) spectrum of compound **5h**.

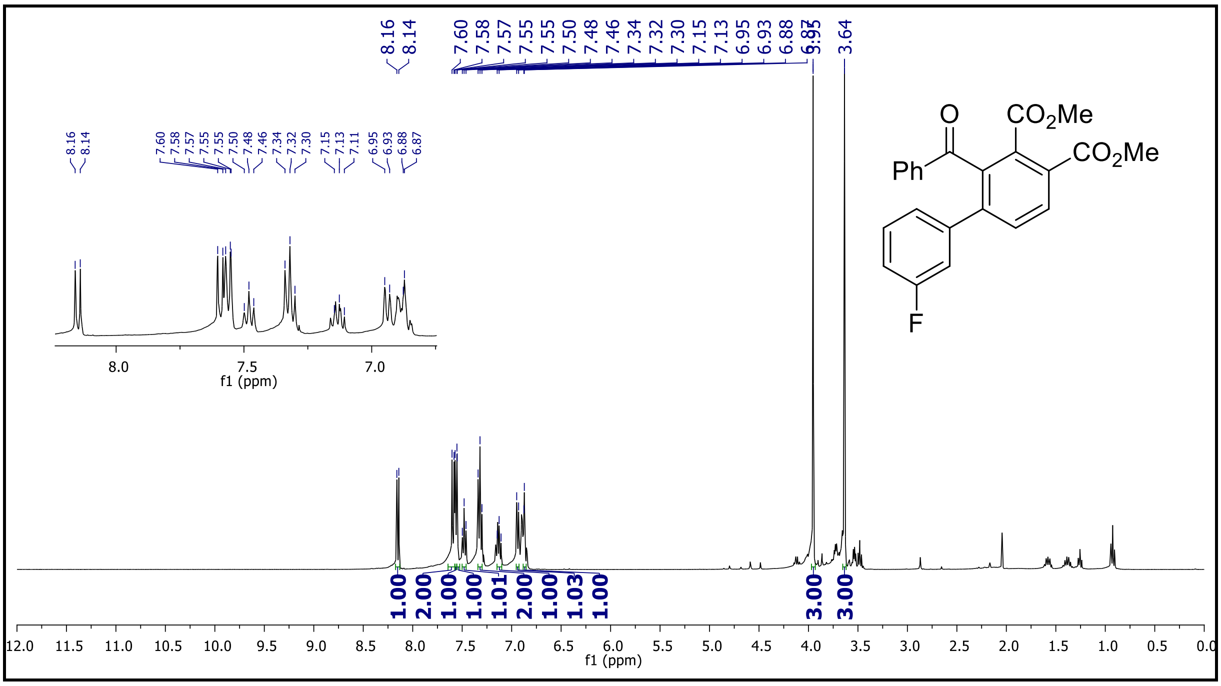

Figure S69. ^{1}H NMR (400 MHz, $CDCl_3$) spectrum of compound **5i**.

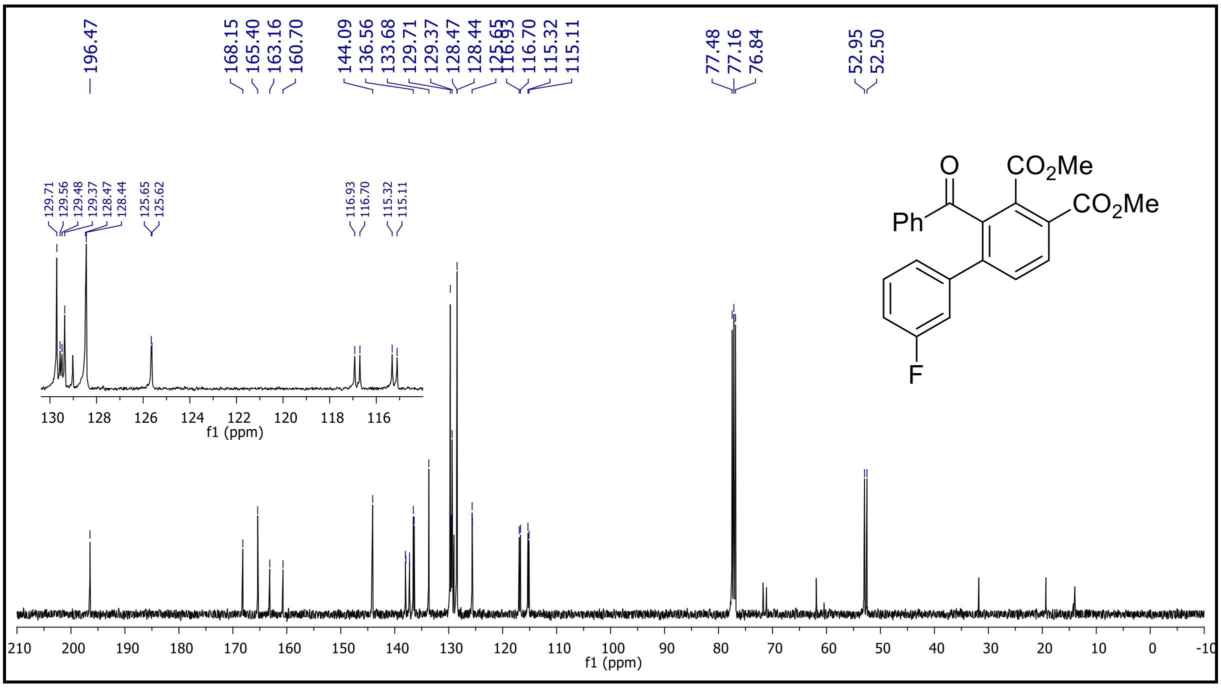

Figure S70. $^{13}C\{^1H\}$ NMR (100 MHz, $CDCl_3$) spectrum of compound **5i**.

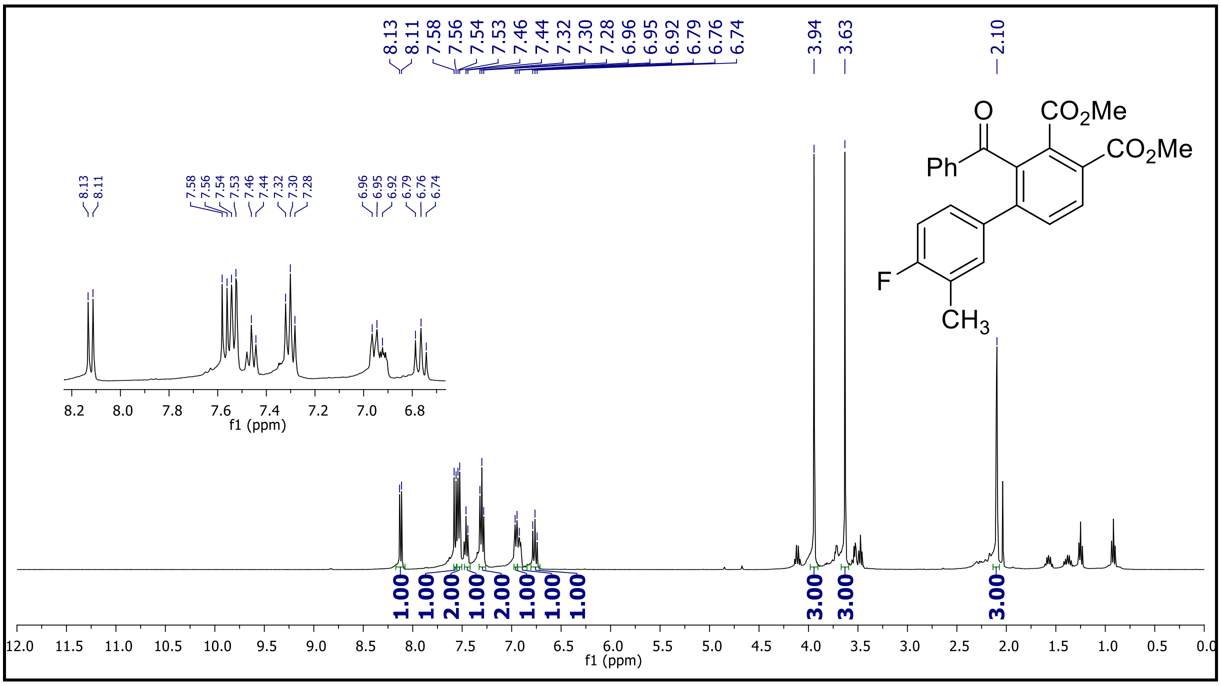

Figure S71. ^1H NMR (400 MHz, $CDCl_3$) spectrum of compound **5j**.

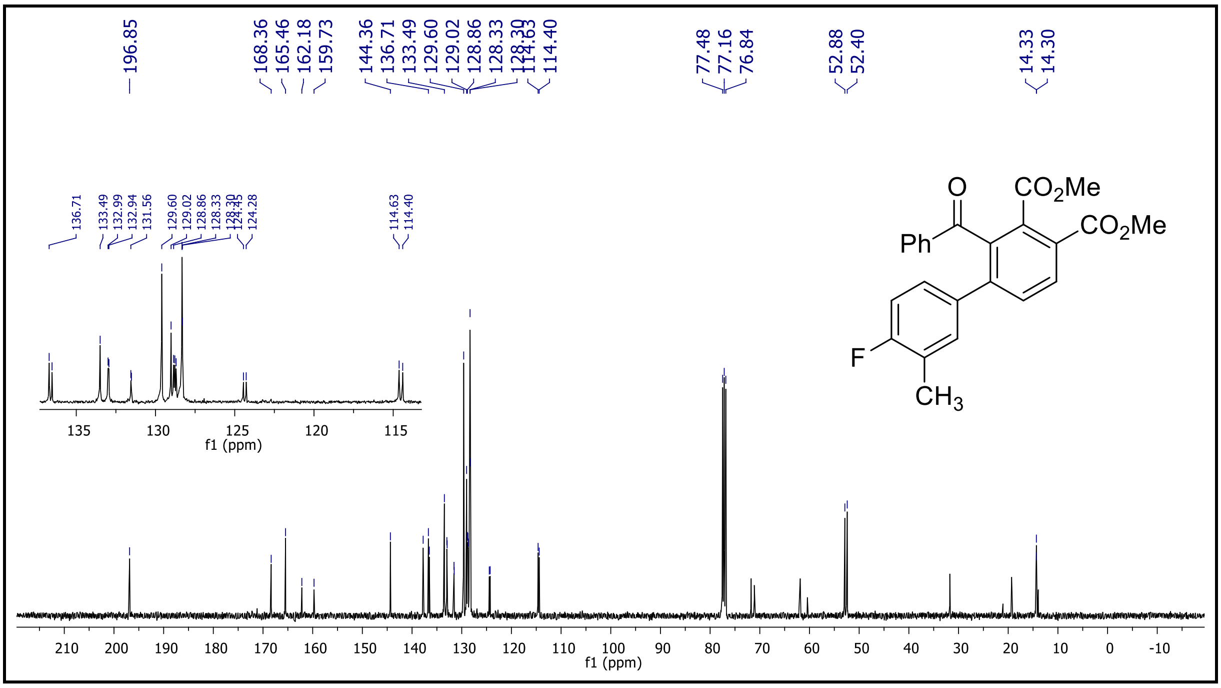

Figure S72. $^{13}C\{^{1}H\}$ NMR (100 MHz, $CDCl_3$) spectrum of compound **5j**.

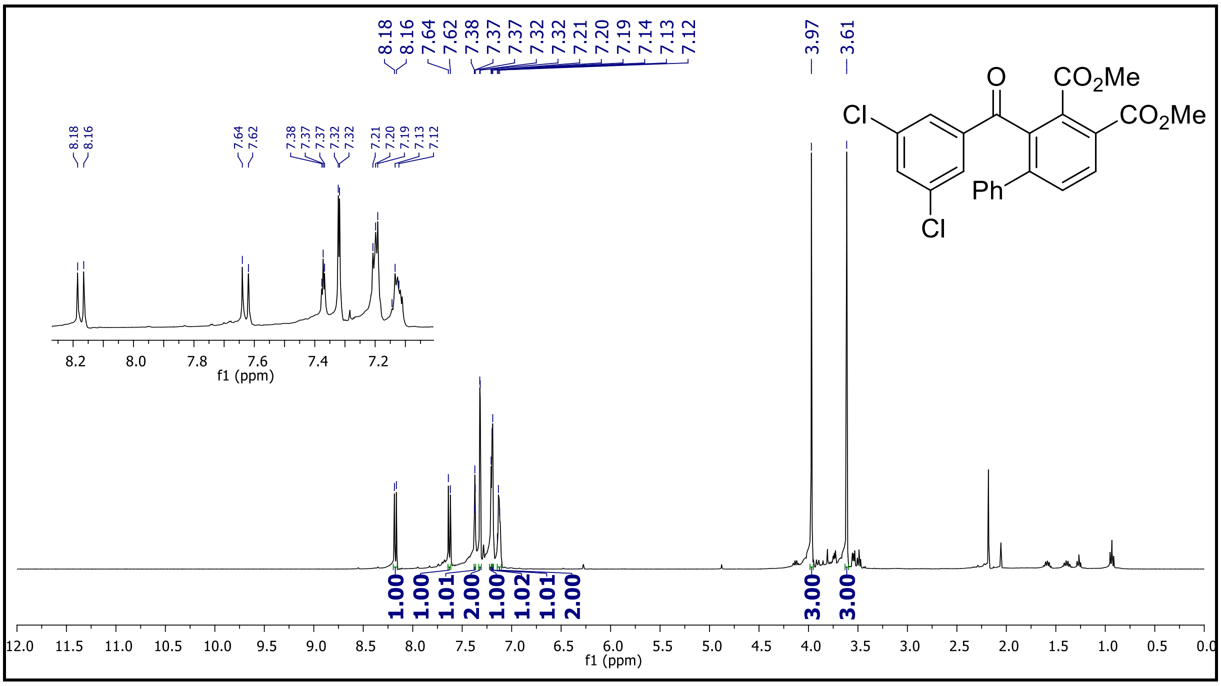

Figure S73. ^{1}H NMR (400 MHz, $CDCl_3$) spectrum of compound **5k**.

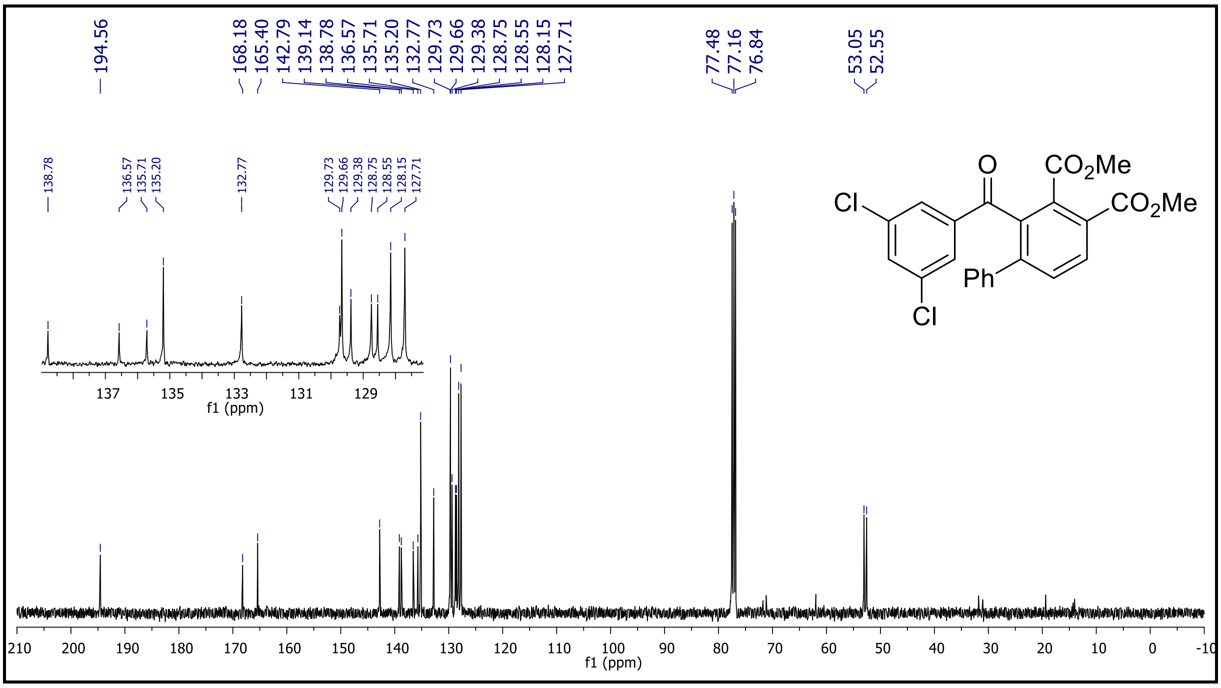

Figure S74. $^{13}C\{^{1}H\}$ NMR (100 MHz, $CDCl_3$) spectrum of compound **5k**.

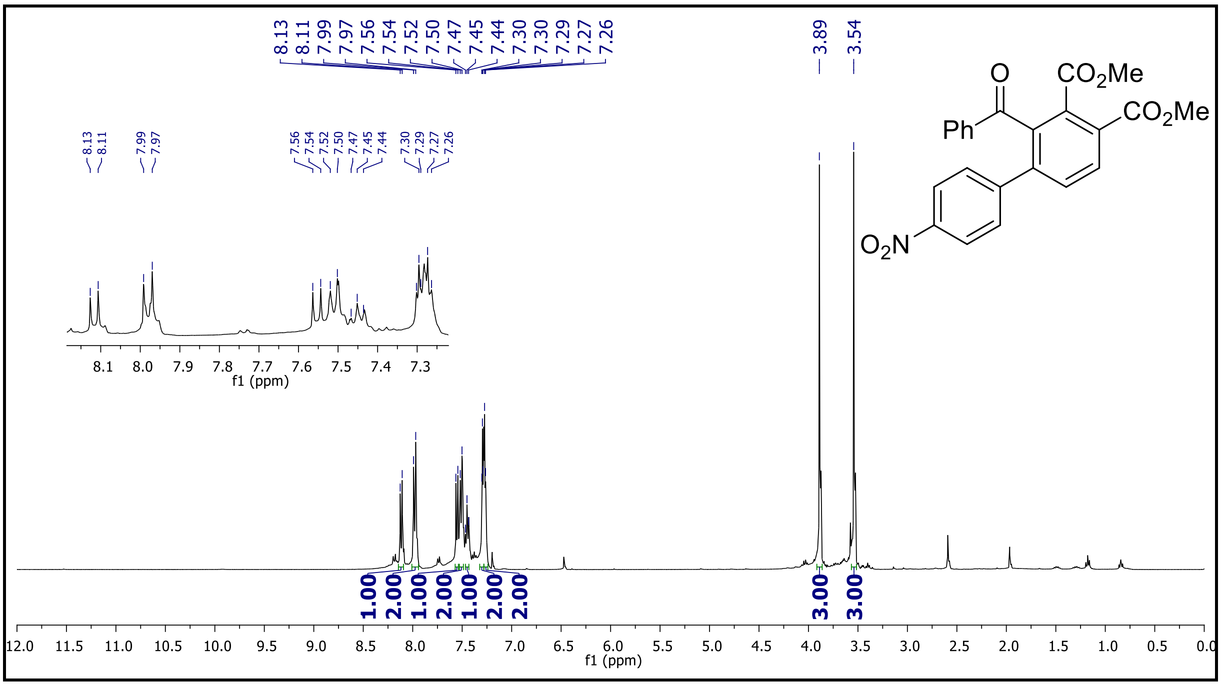

Figure S75. ^{1}H NMR (400 MHz, $CDCl_3$) spectrum of compound **5l**.

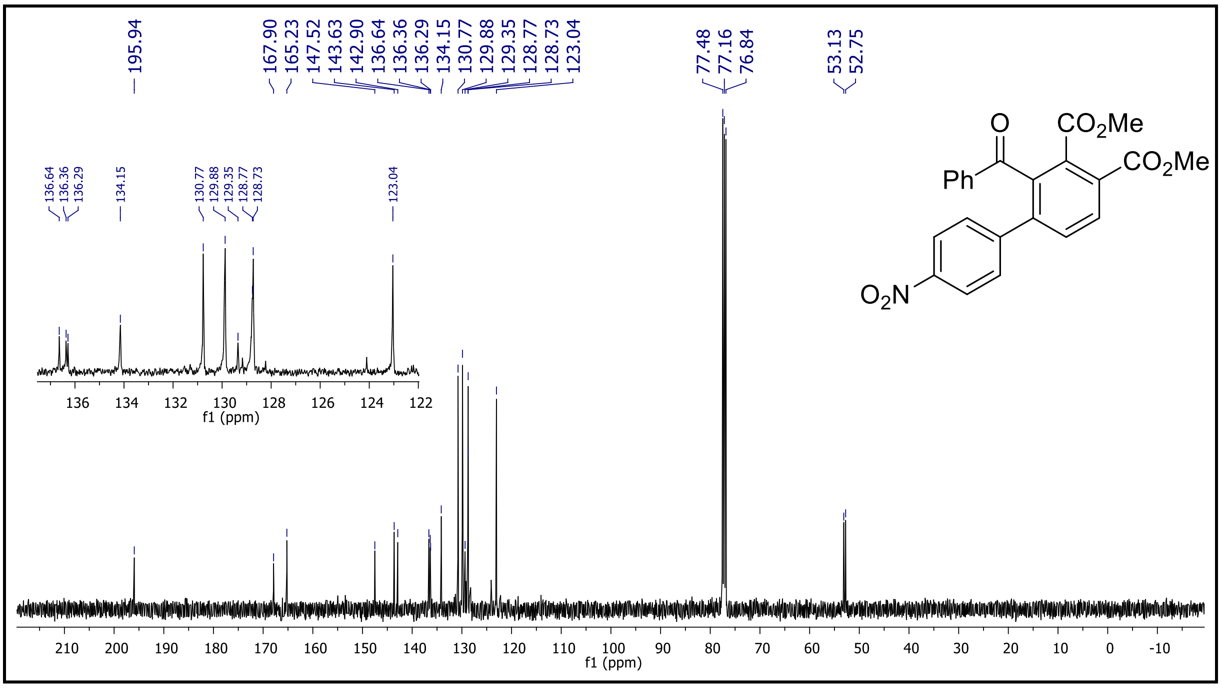

Figure S76. $^{13}C\{^1H\}$ NMR (100 MHz, $CDCl_3$) spectrum of compound **5l**.

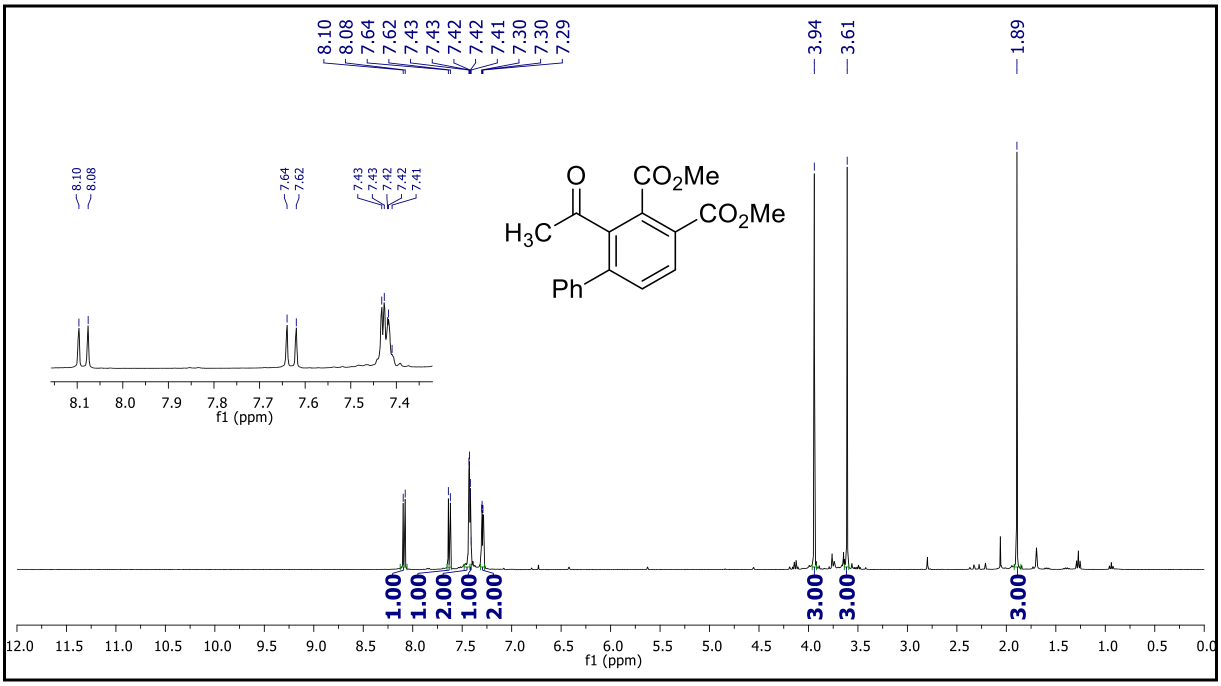

Figure S77. ^1H NMR (400 MHz, $CDCl_3$) spectrum of compound **5m**.

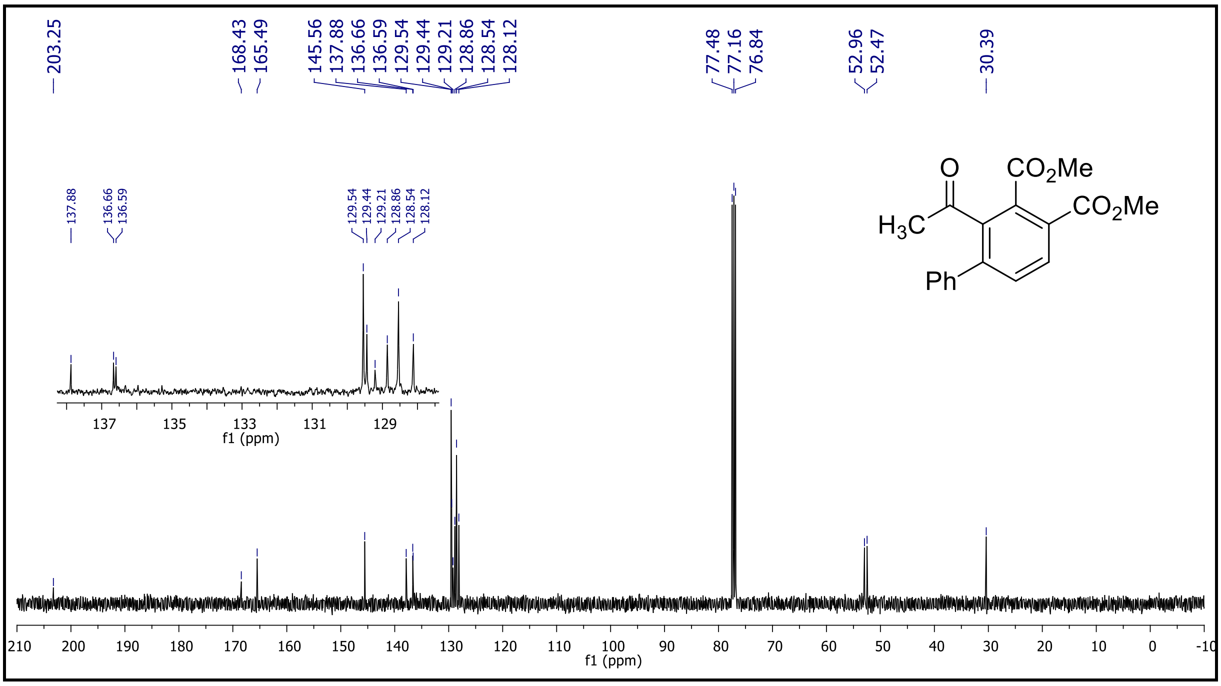

Figure S78. $^{13}C\{^1H\}$ NMR (100 MHz, $CDCl_3$) spectrum of compound **5m**.

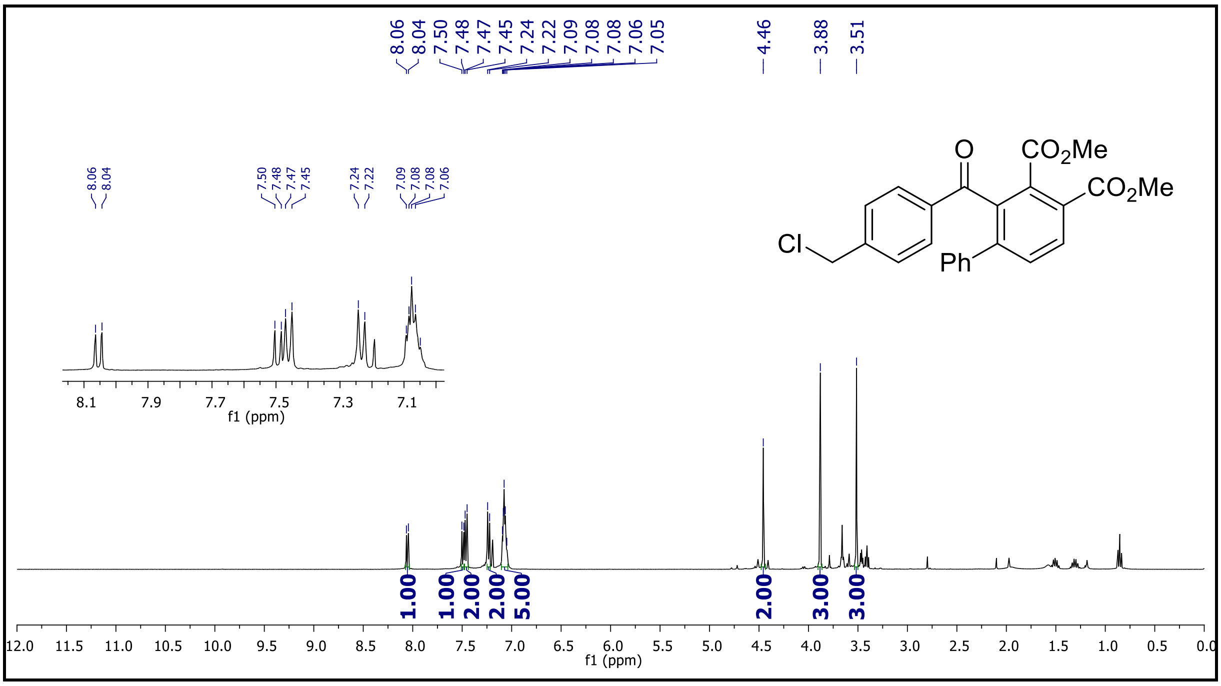

Figure S79. ^1H NMR (400 MHz, $CDCl_3$) spectrum of compound **5n**.

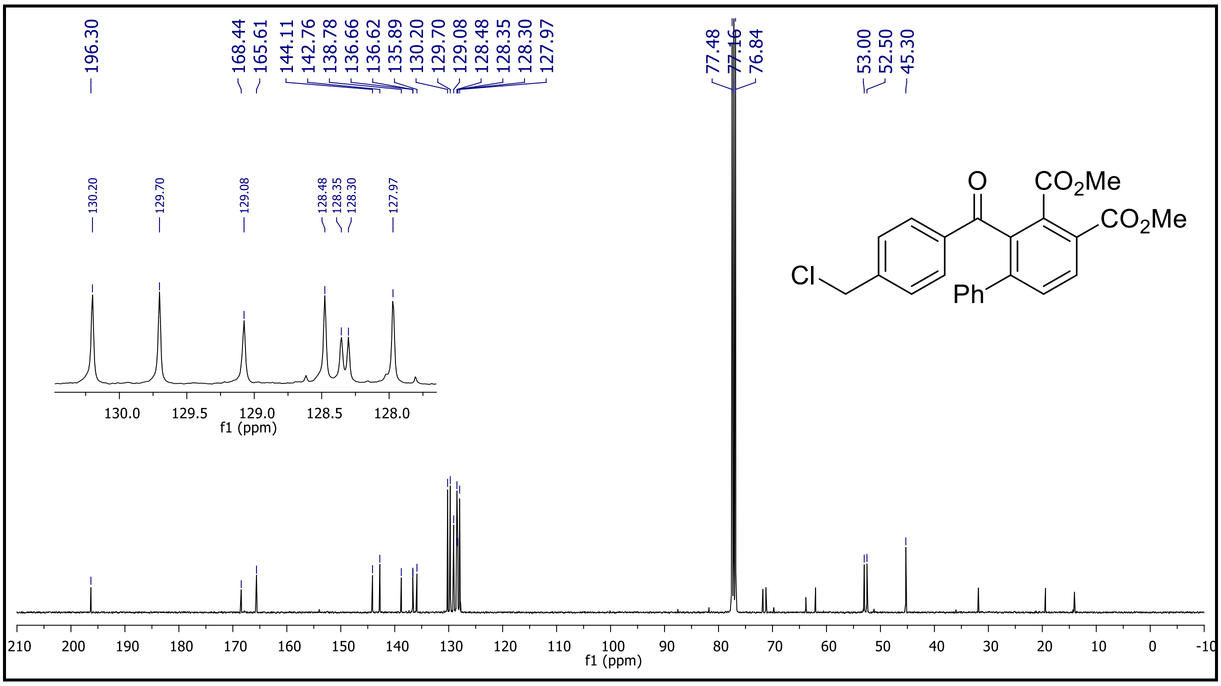

Figure S80. $^{13}C\{^{1}H\}$ NMR (100 MHz, $CDCl_3$) spectrum of compound **5n**.

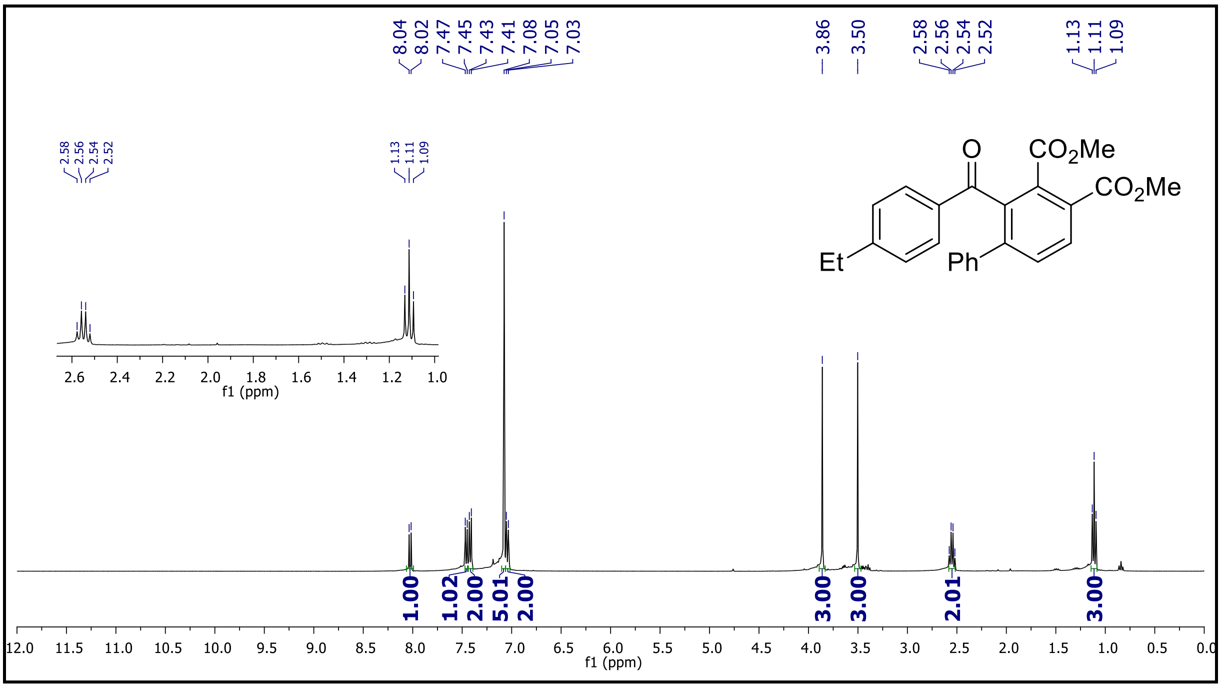

Figure S81. ^{1}H NMR (400 MHz, $CDCl_3$) spectrum of compound **5o**.

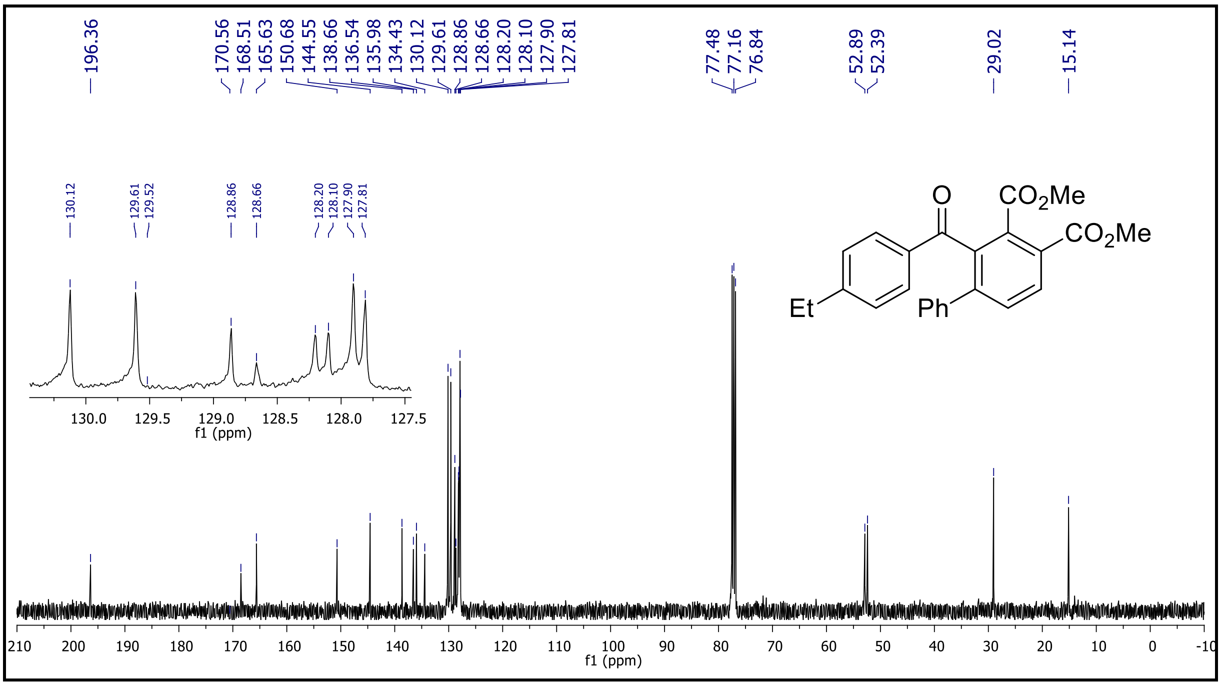

Figure S82. $^{13}C\{^1H\}$ NMR (100 MHz, $CDCl_3$) spectrum of compound **5o**.

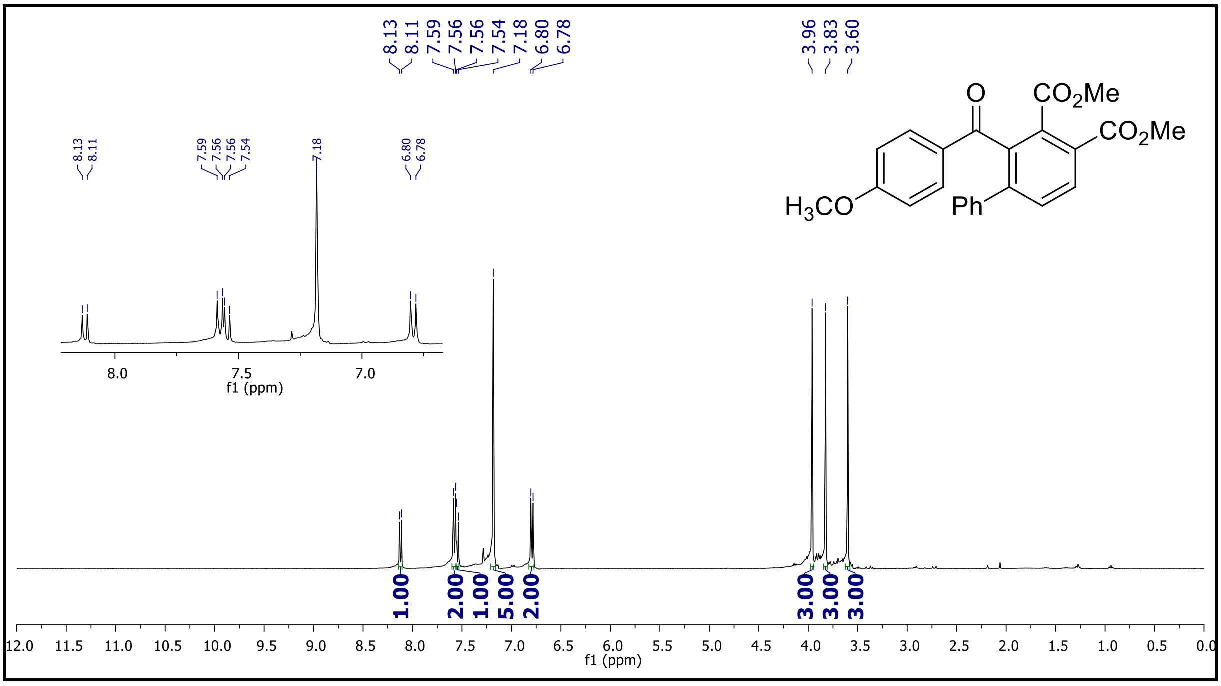

Figure S83. ^1H NMR (400 MHz, $CDCl_3$) spectrum of compound **5p**.

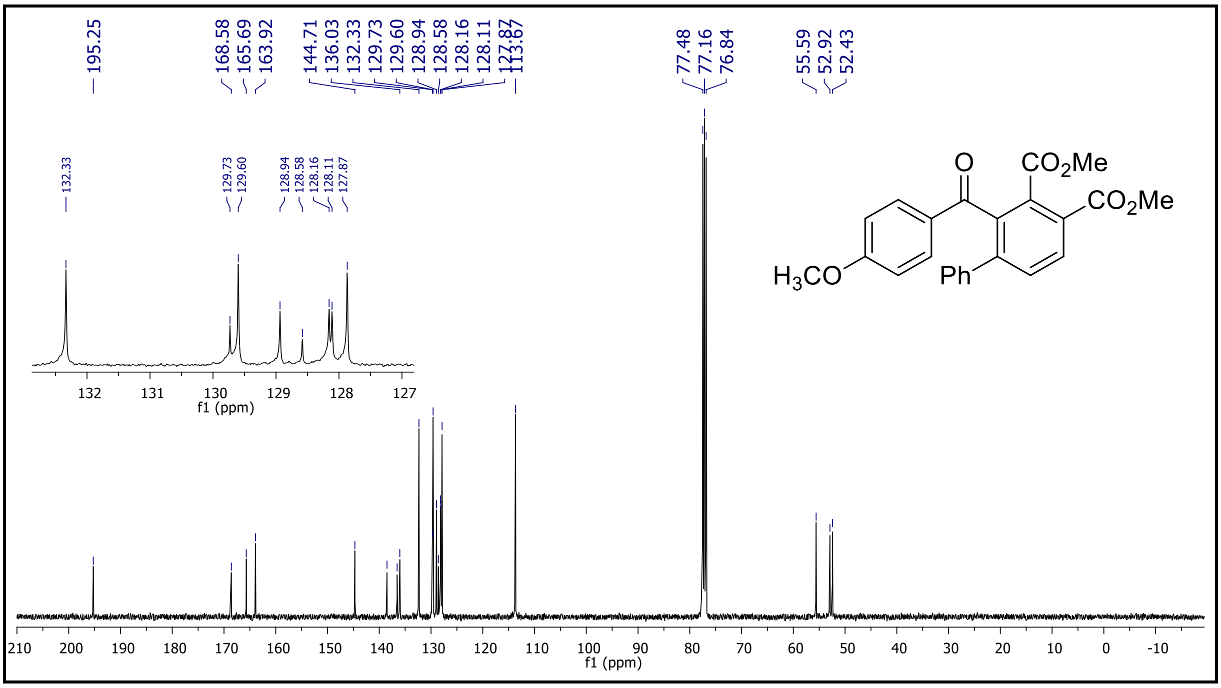

Figure S84. $^{13}C\{^{1}H\}$ NMR (100 MHz, $CDCl_3$) spectrum of compound **5p**.

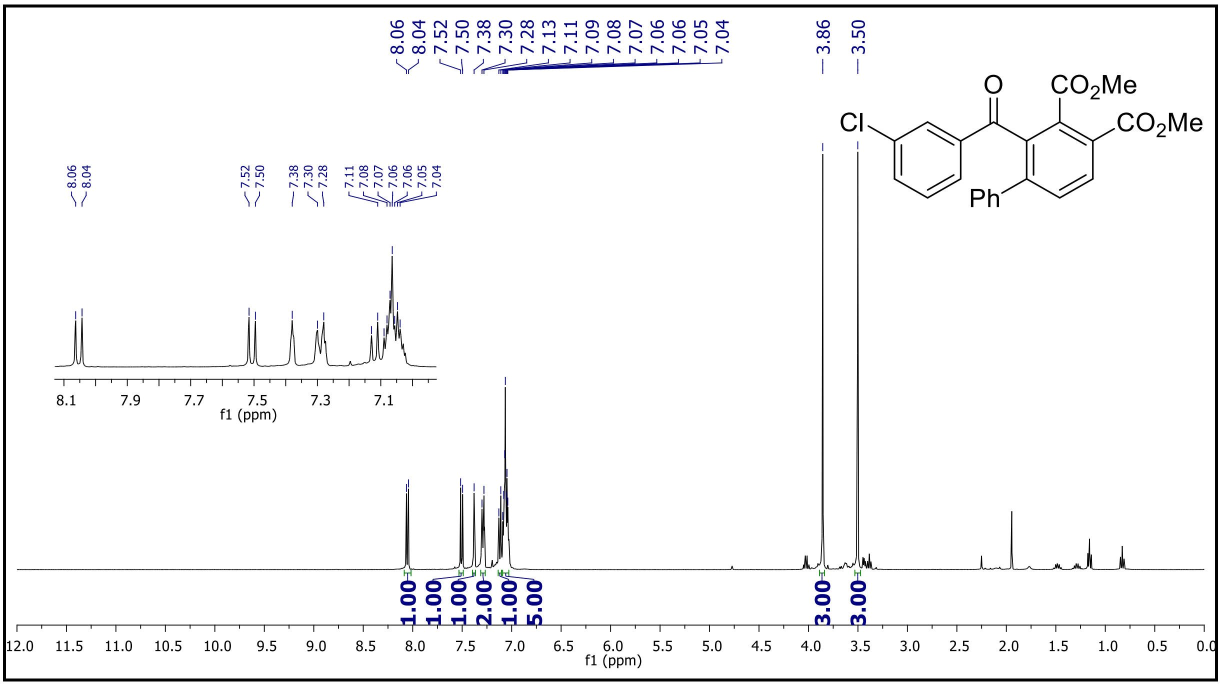

Figure S85. ^{1}H NMR (400 MHz, $CDCl_3$) spectrum of compound **5q**.

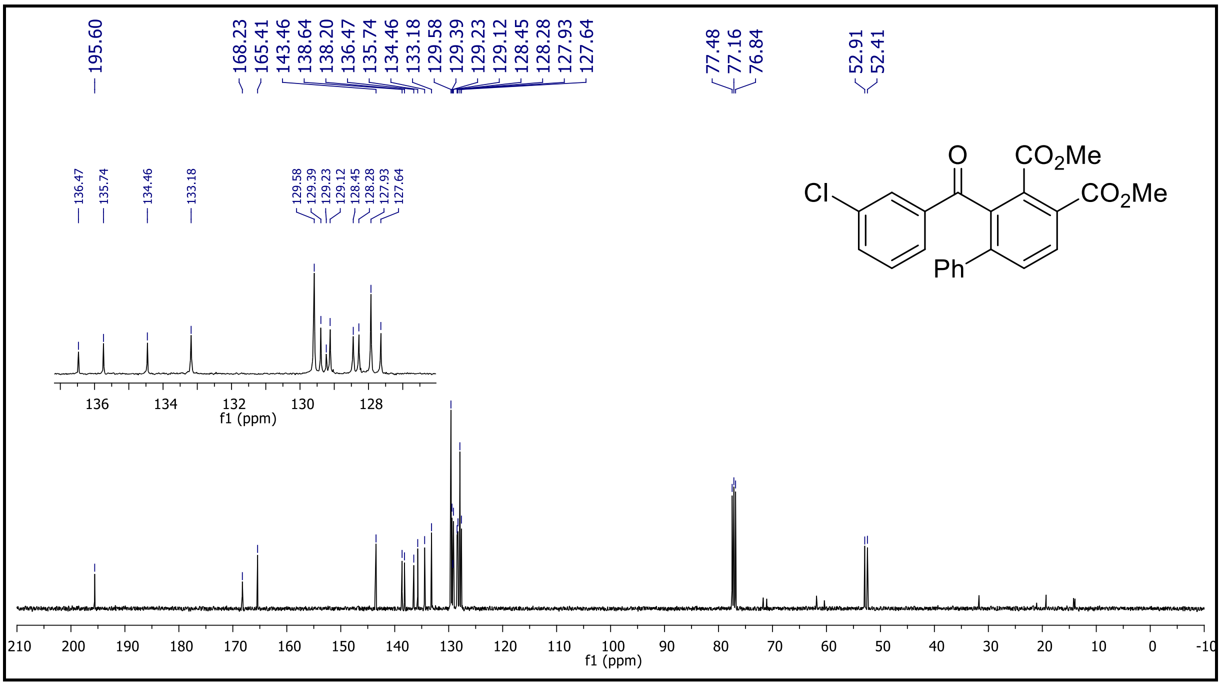

Figure S86. $^{13}C\{^{1}H\}$ NMR (100 MHz, $CDCl_3$) spectrum of compound **5q**.

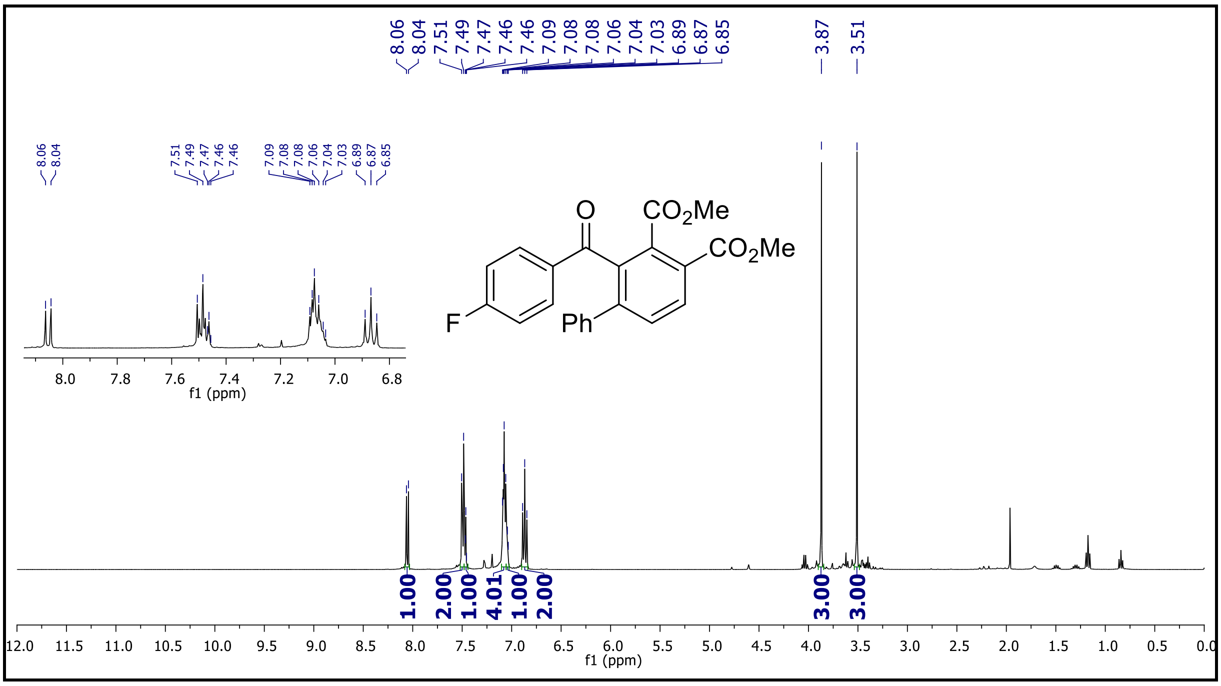

Figure S87. ^{1}H NMR (400 MHz, $CDCl_3$) spectrum of compound **5r**.

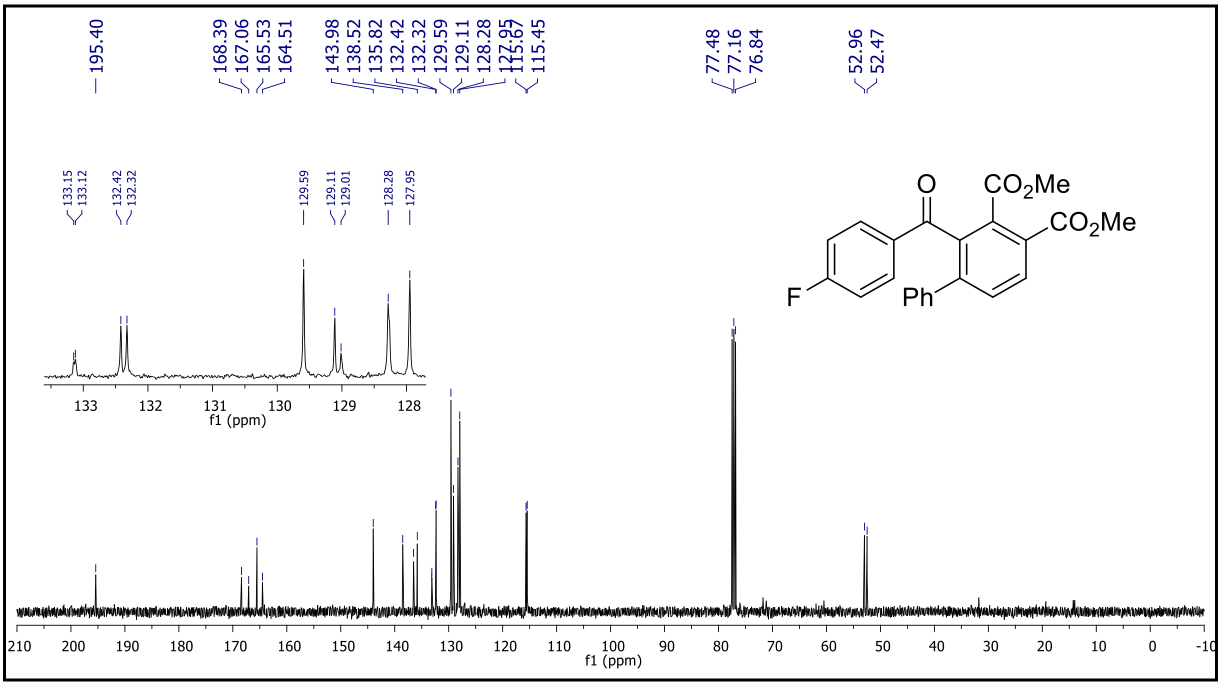

Figure S88. $^{13}C\{^{1}H\}$ NMR (100 MHz, $CDCl_3$) spectrum of compound **5r**.

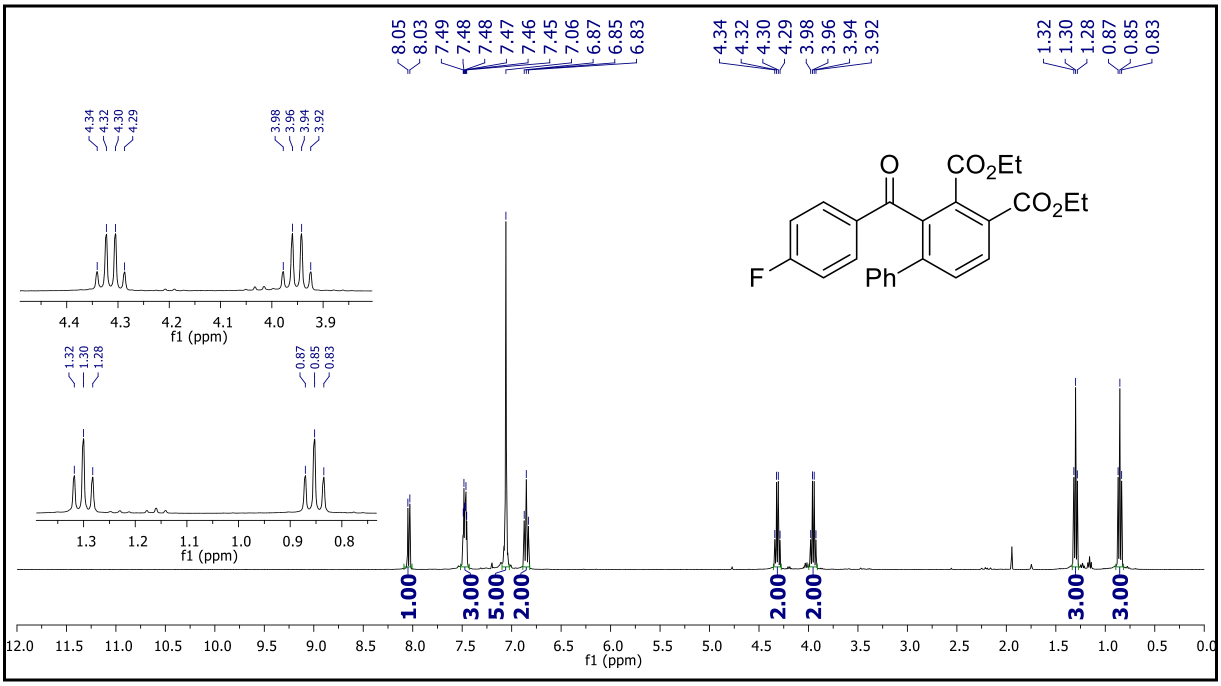

Figure S89. ^{1}H NMR (400 MHz, $CDCl_3$) spectrum of compound **5r'**.

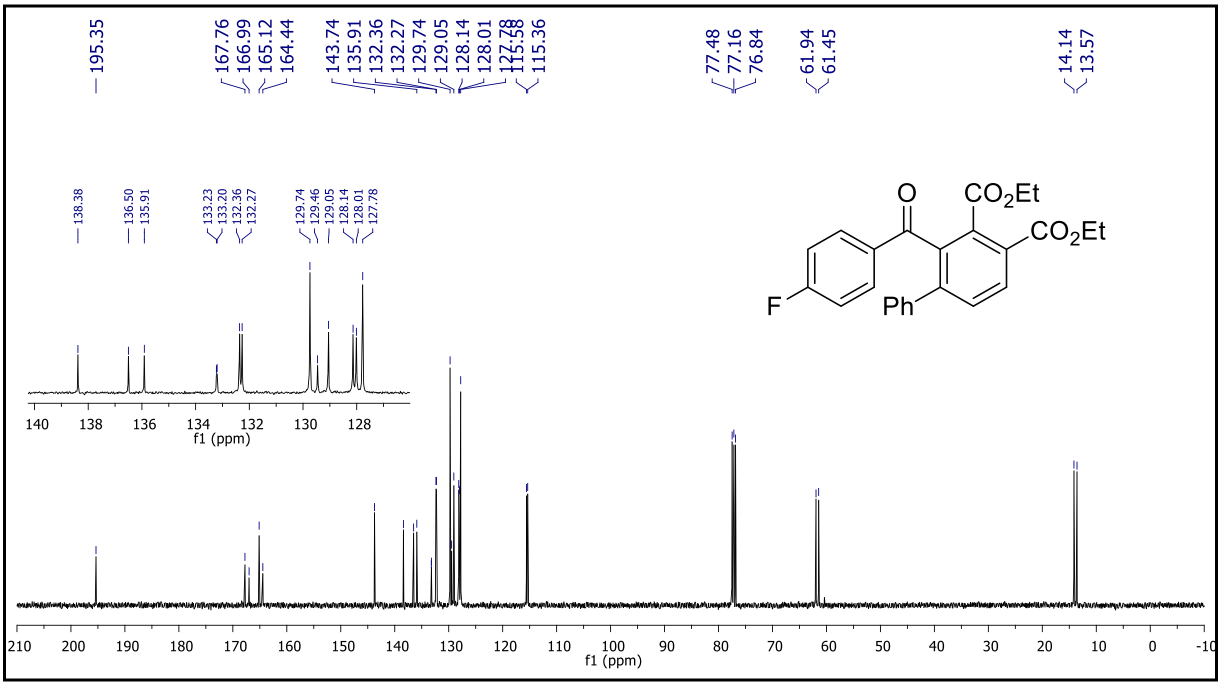

Figure S90. $^{13}C\{^{1}H\}$ NMR (100 MHz, $CDCl_3$) spectrum of compound **5r'**.

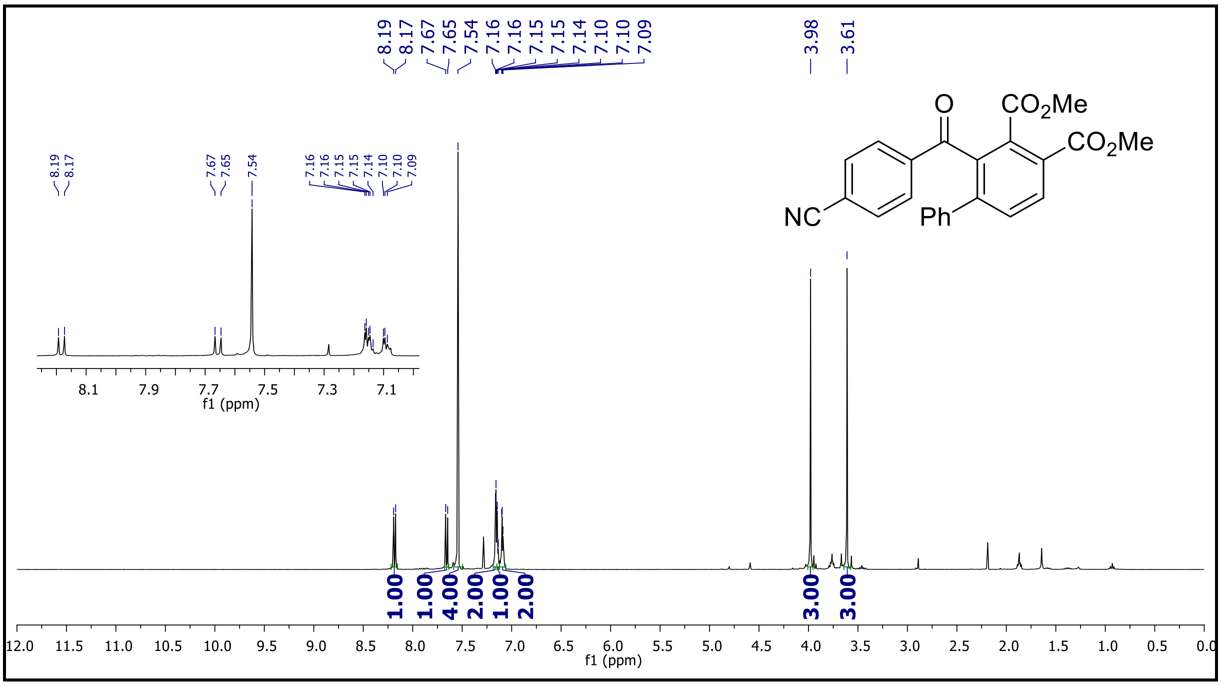

Figure S91. ^{1}H NMR (400 MHz, $CDCl_3$) spectrum of compound **5s**.

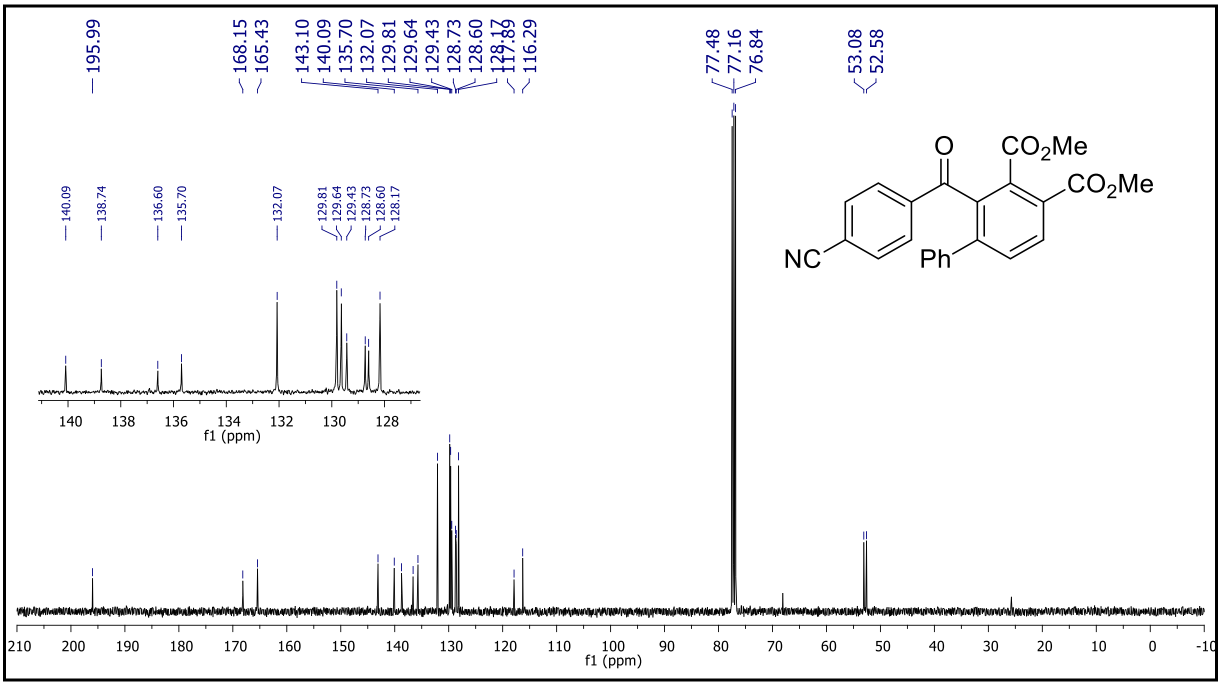

Figure S92. $^{13}C\{^{1}H\}$ NMR (100 MHz, $CDCl_3$) spectrum of compound **5s**.

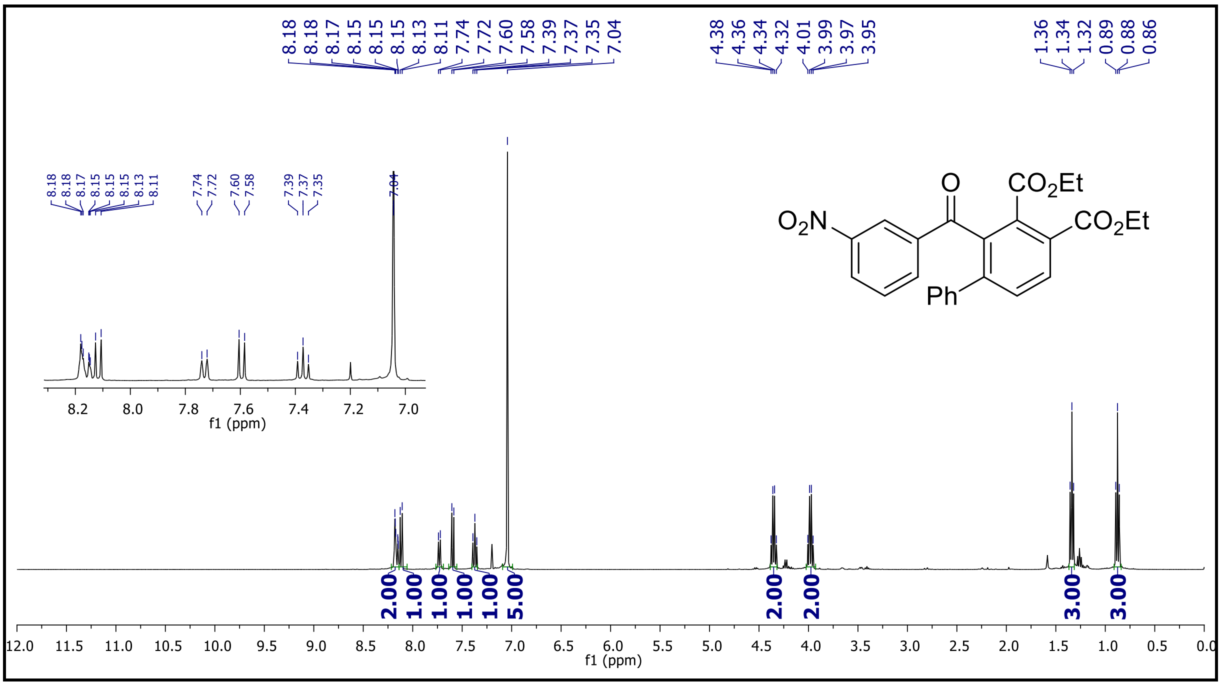

Figure S93. ^{1}H NMR (400 MHz, $CDCl_3$) spectrum of compound **5t'**.

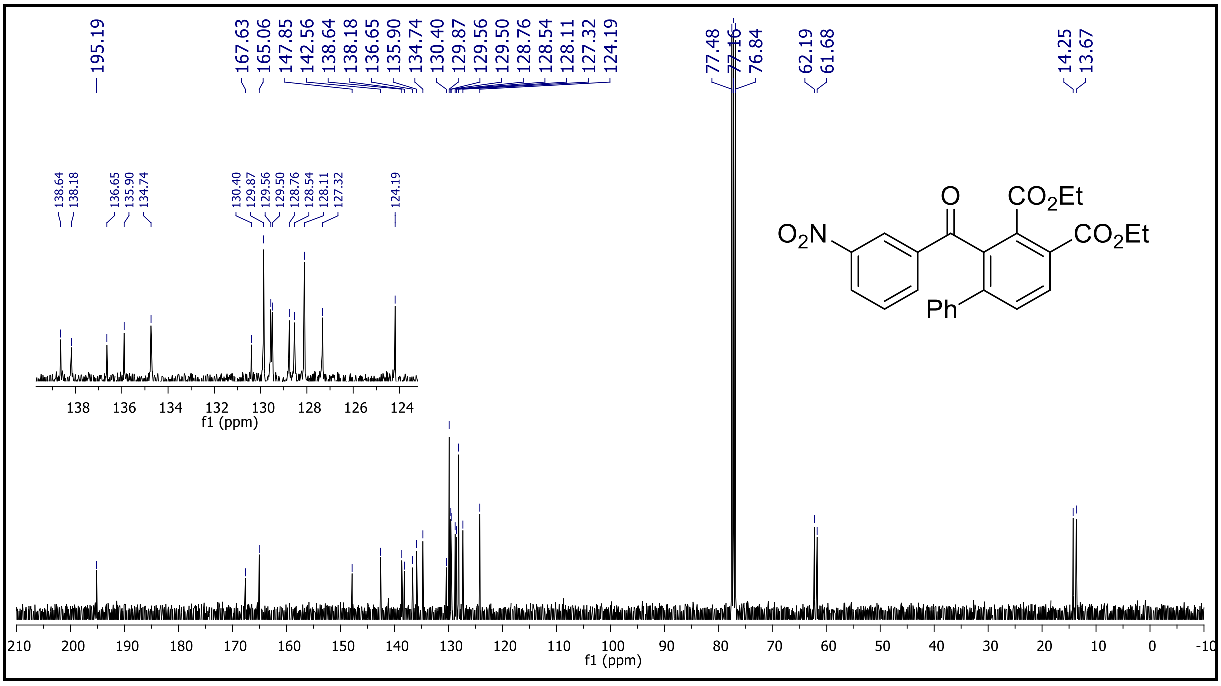

Figure S94. $^{13}C\{^{1}H\}$ NMR (100 MHz, $CDCl_3$) spectrum of compound **5t'**.

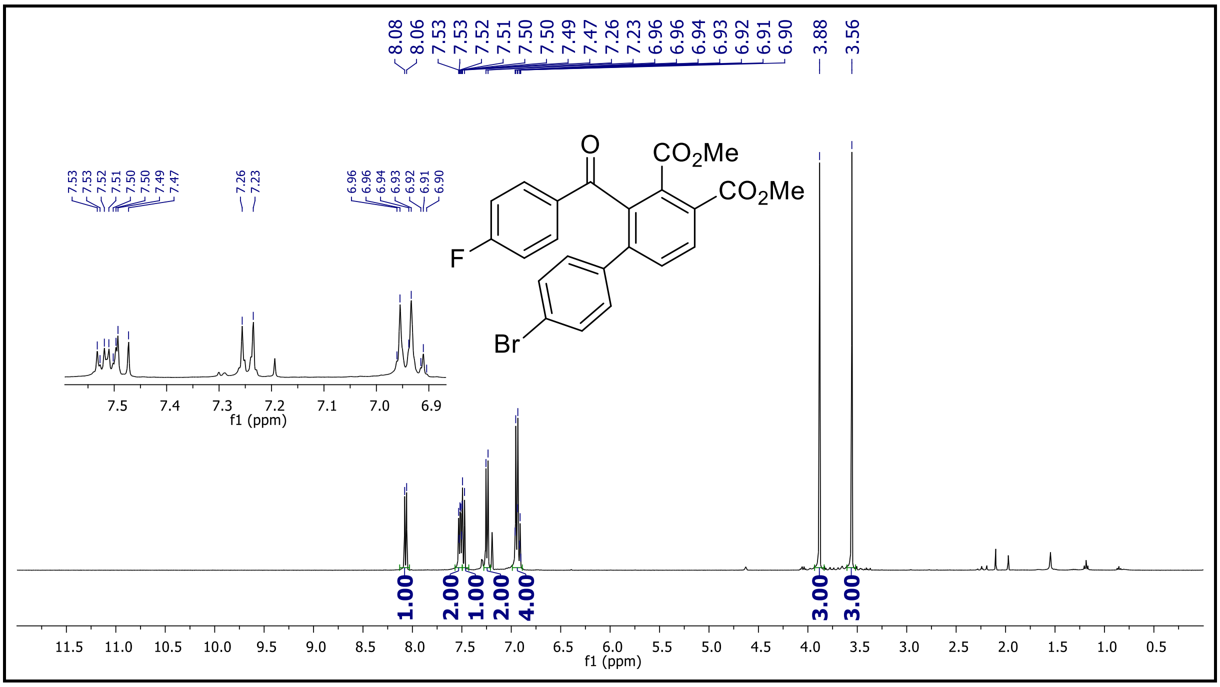

Figure S95. ^{1}H NMR (400 MHz, $CDCl_3$) spectrum of compound **5u**.

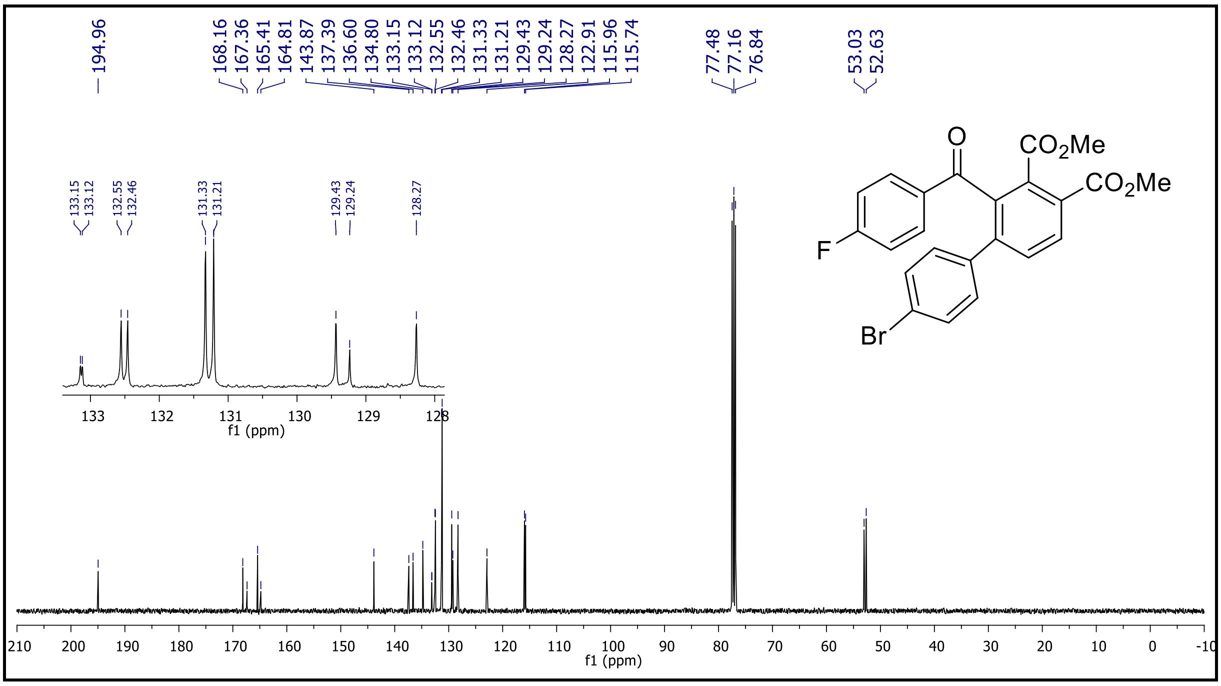

Figure S96. $^{13}C\{^{1}H\}$ NMR (100 MHz, $CDCl_3$) spectrum of compound **5u**.

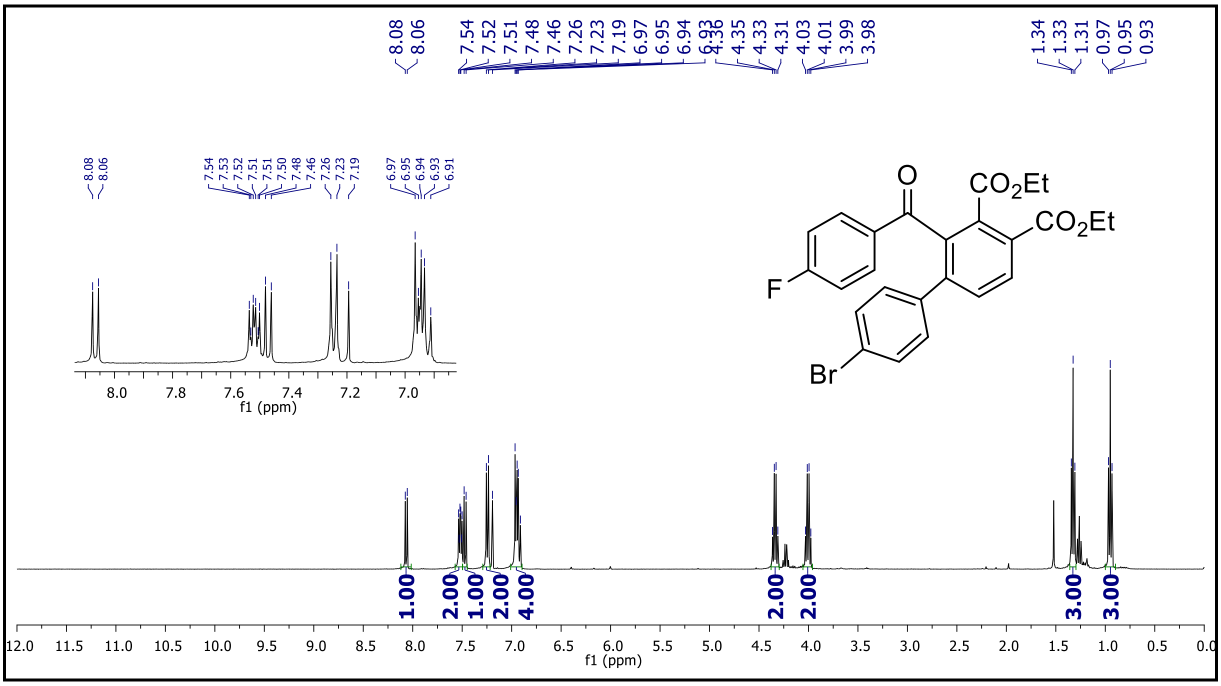

Figure S97. ^{1}H NMR (400 MHz, $CDCl_3$) spectrum of compound **5u'**.

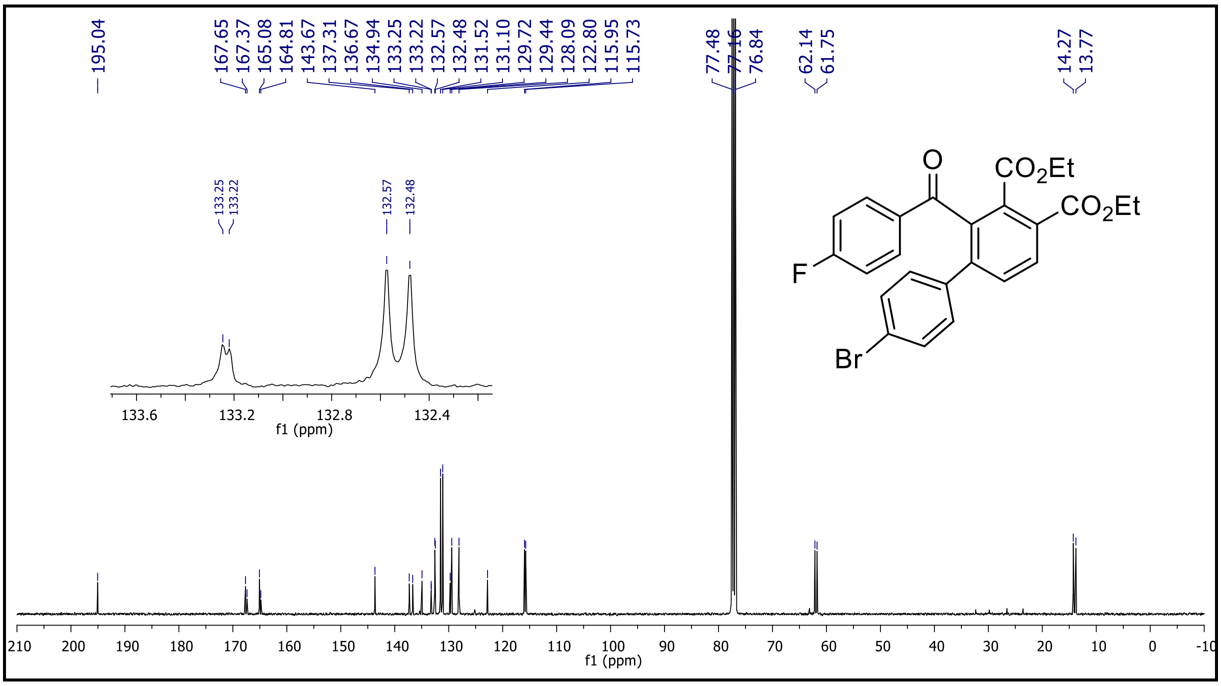

Figure S98. $^{13}C\{^1H\}$ NMR (100 MHz, $CDCl_3$) spectrum of compound **5u'**.

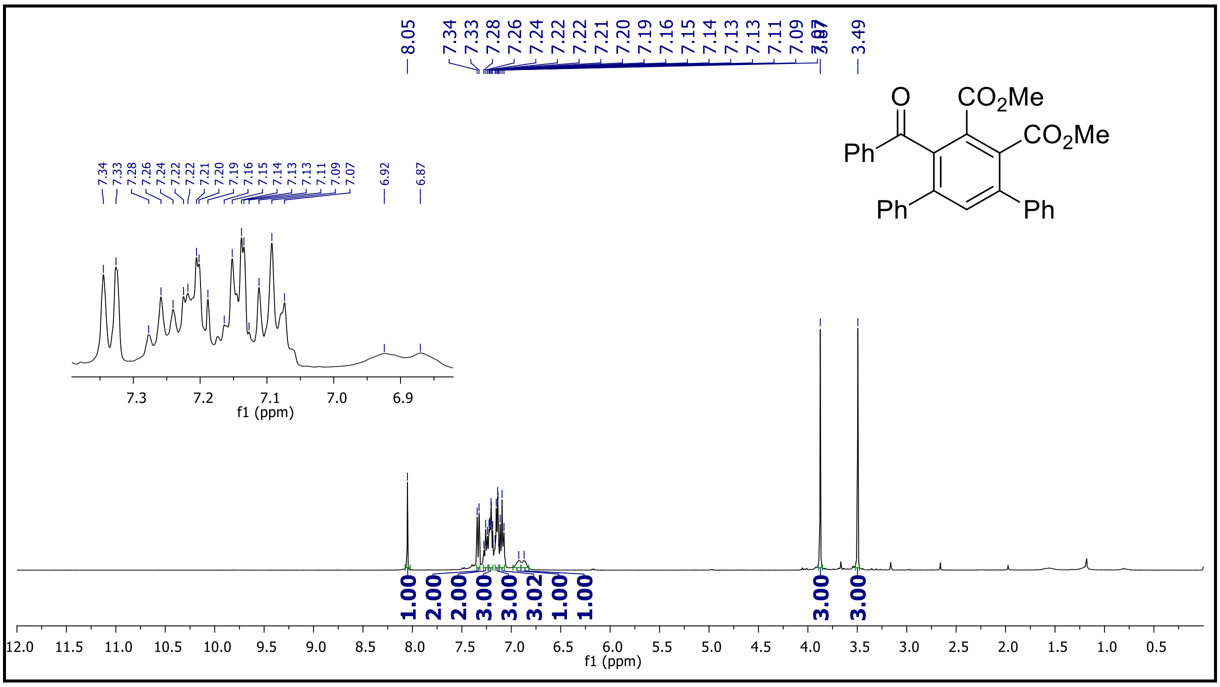

Figure 99. ^1H NMR (400 MHz, $CDCl_3$) spectrum of compound **5v**.

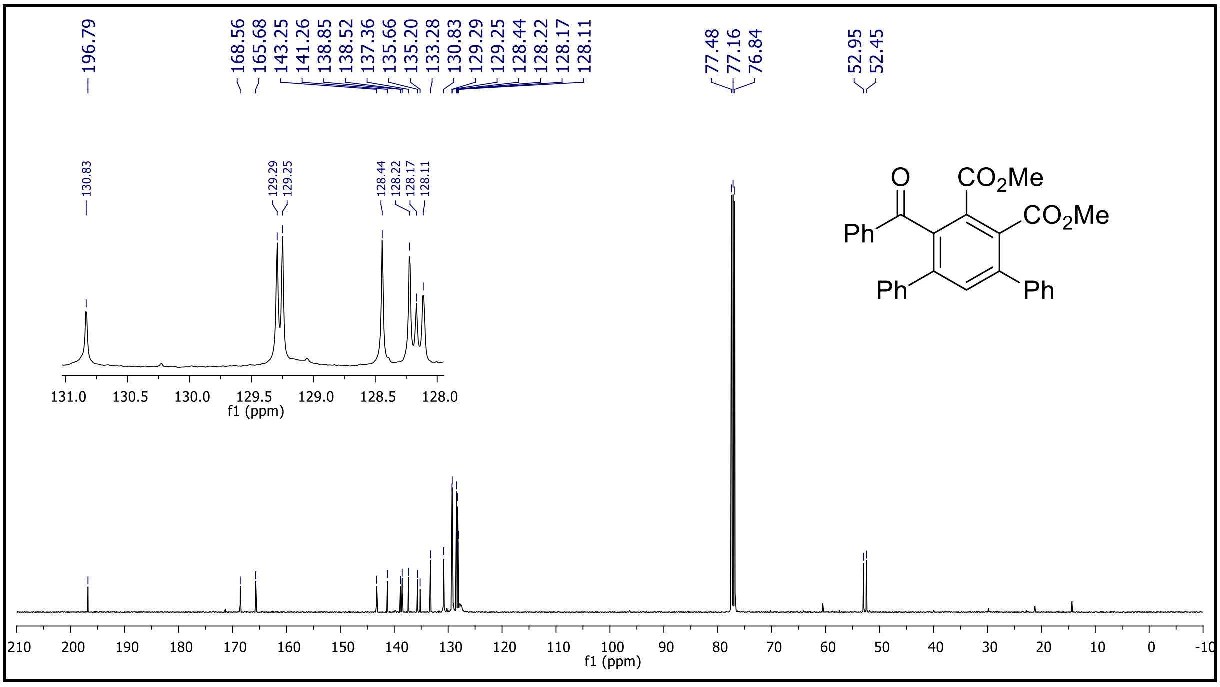

Figure S100. $^{13}C\{^{1}H\}$ NMR (100 MHz, $CDCl_3$) spectrum of compound **5v**.

Copies of ^{1}H and $^{13}C\{^{1}H\}$ NMR Spectra for 3-Alkenyl-1,2-dihydropyridines 6 ve 6'

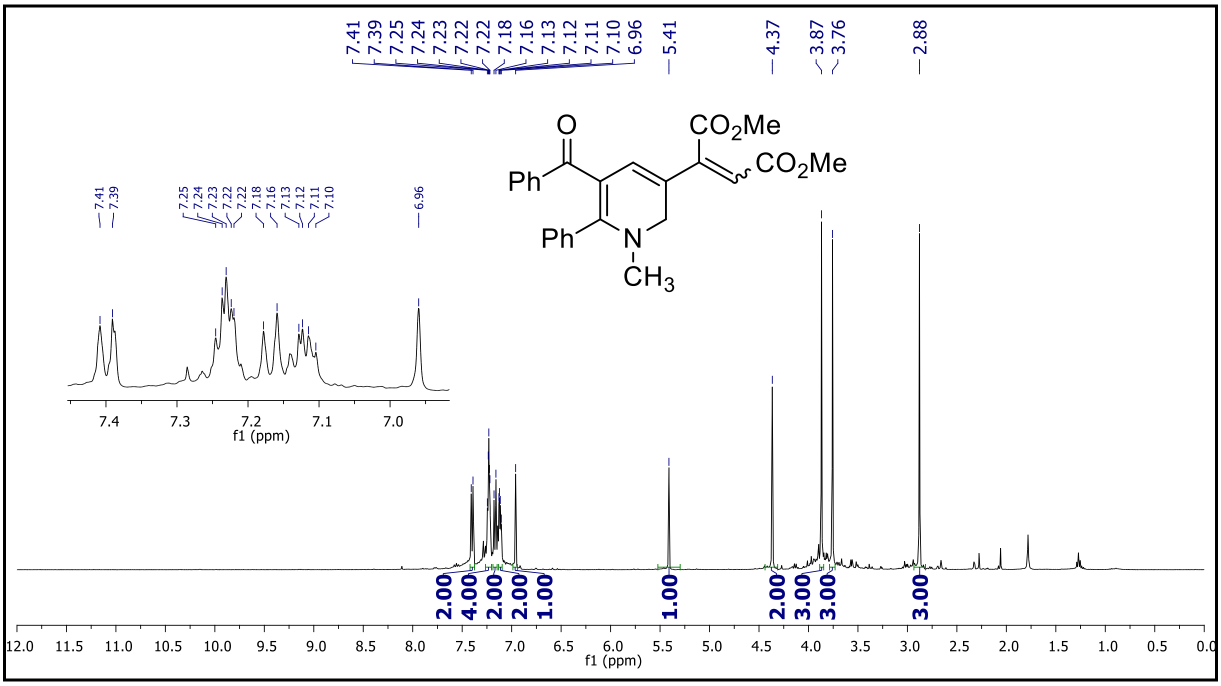

Figure S101. ^{1}H NMR (400 MHz, $CDCl_3$) spectrum of compound **6a**.

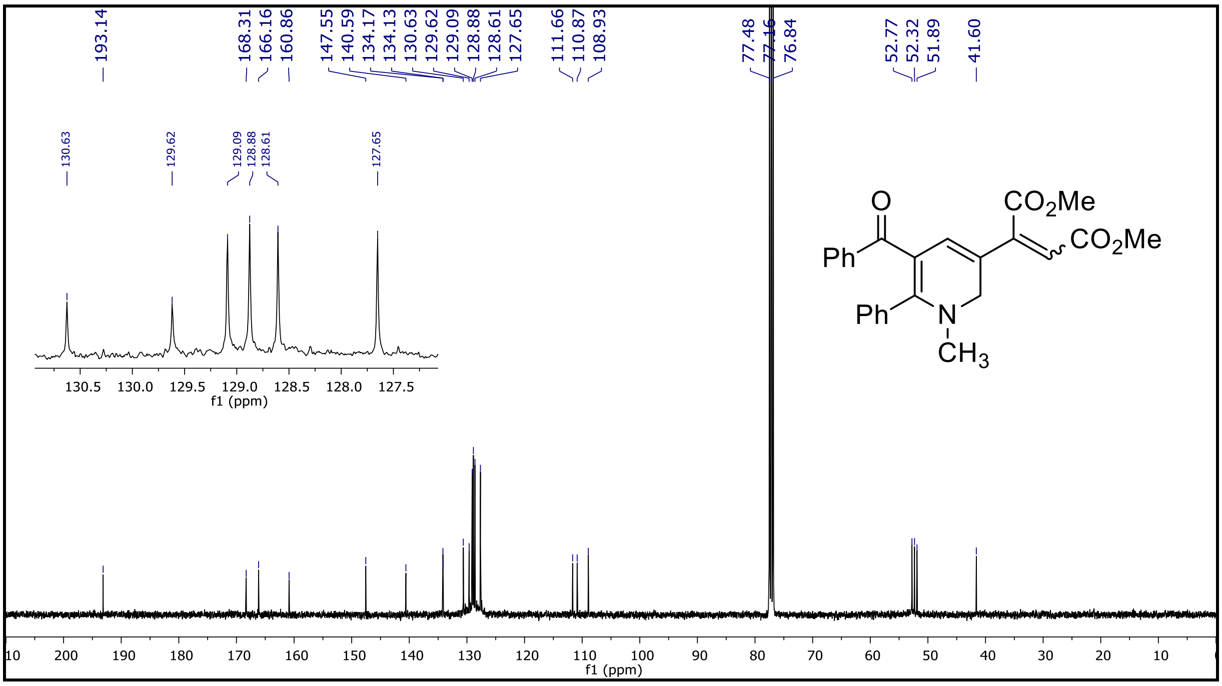

Figure S102. $^{13}C\{^{1}H\}$ NMR (100 MHz, $CDCl_3$) spectrum of compound **6a**.

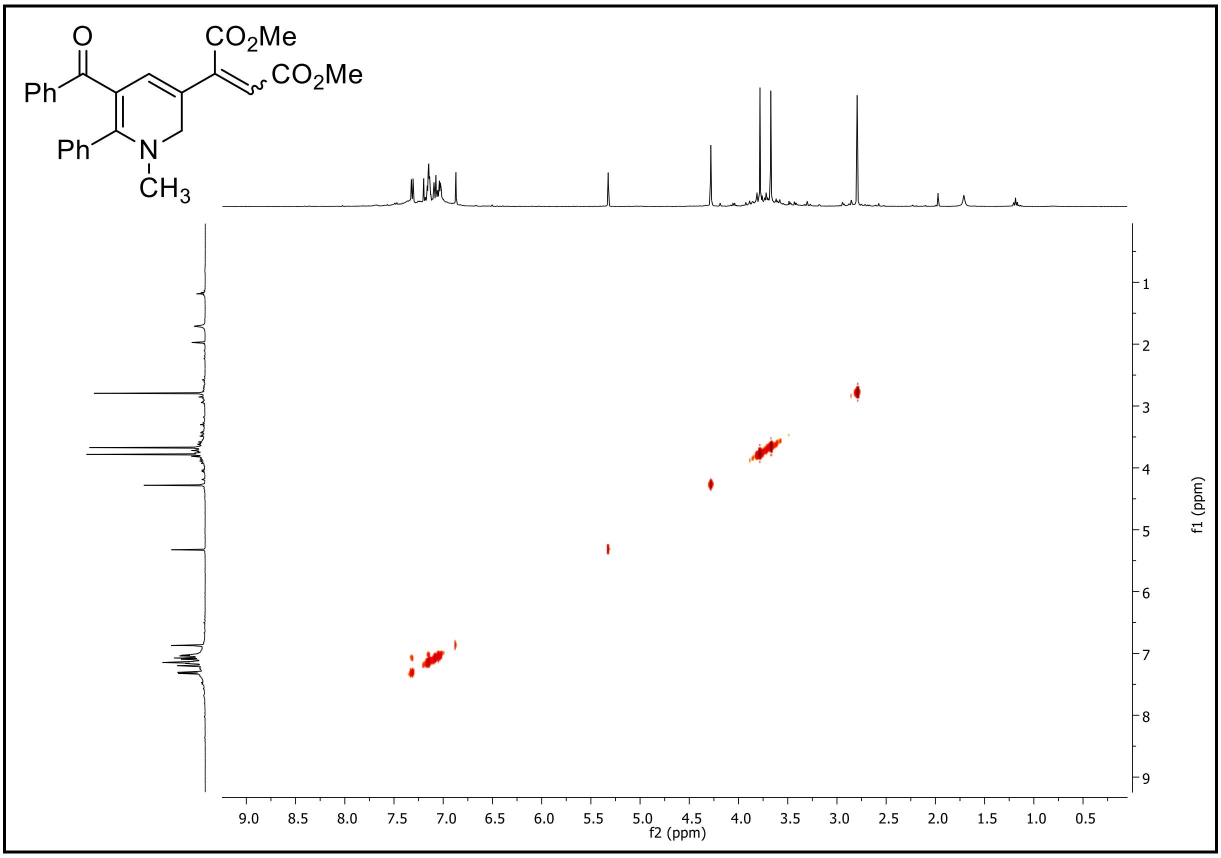

Figure S103. ^{1}H/^{1}H COSY (400/400 MHz, $CDCl_3$) spectrum of compound **6a**.

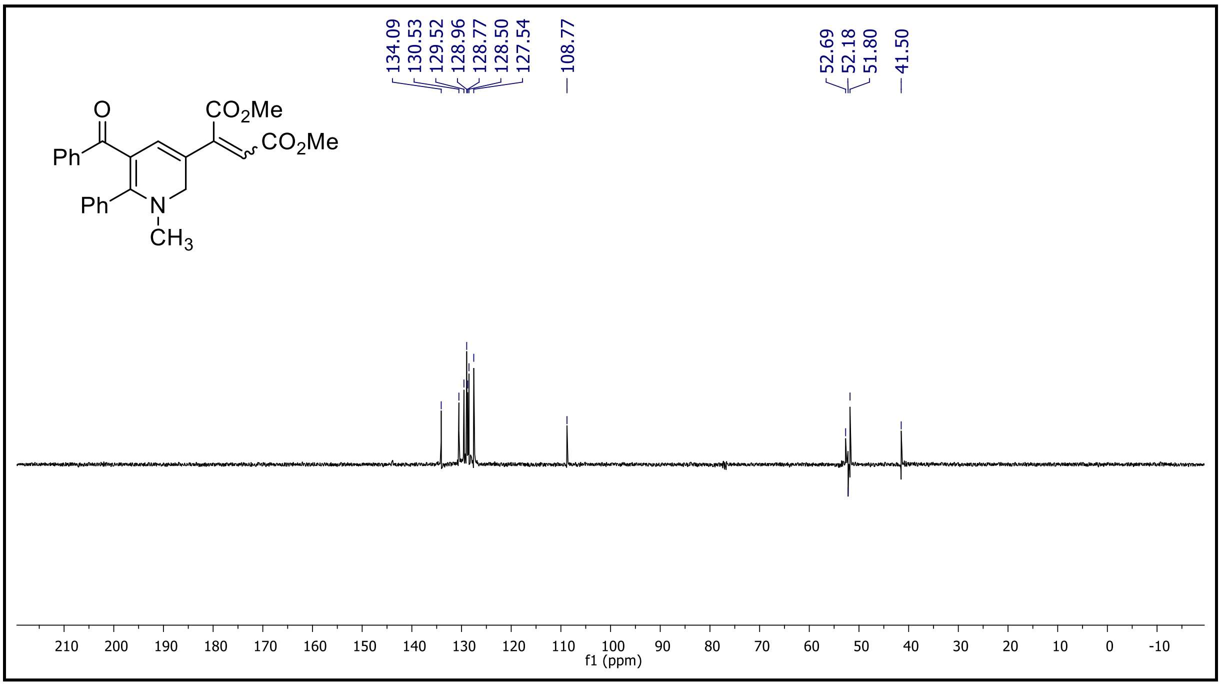

Figure S104. $^{13}C\{^1H\}$ DEPT–135 (100 MHz, $CDCl_3$) spectrum of compound **6a**.

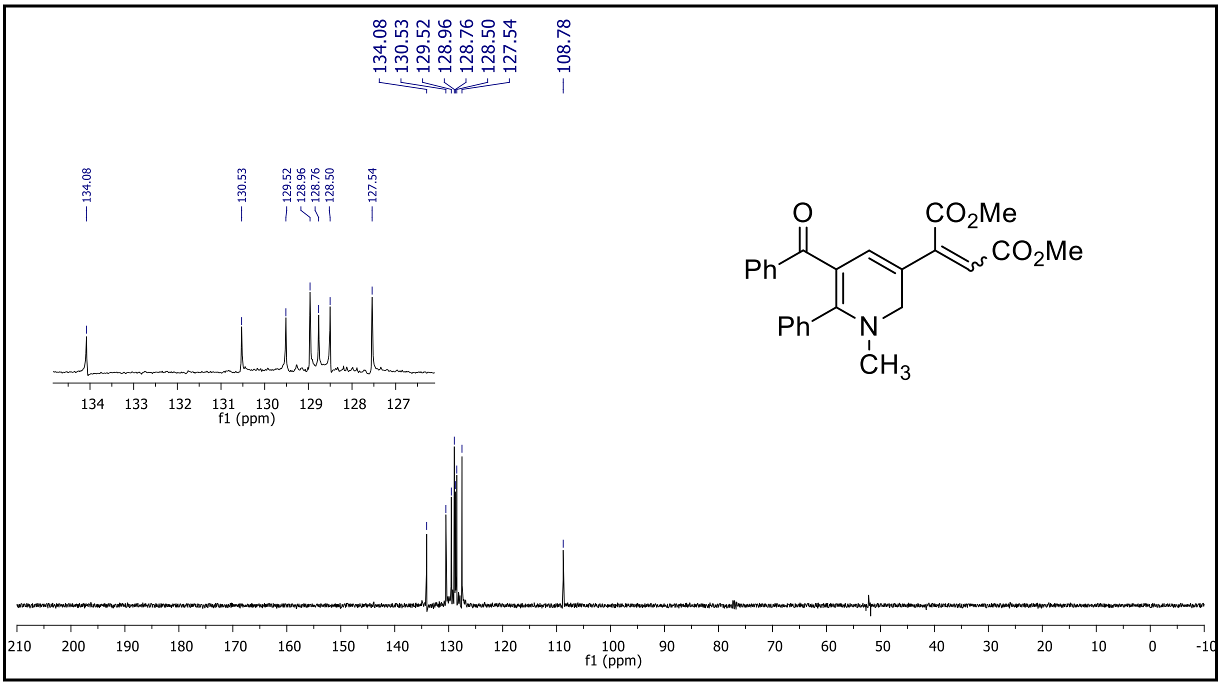

Figure S105. $^{13}C\{^{1}H\}$ DEPT–90 (100 MHz, $CDCl_3$) spectrum of compound **6a**.

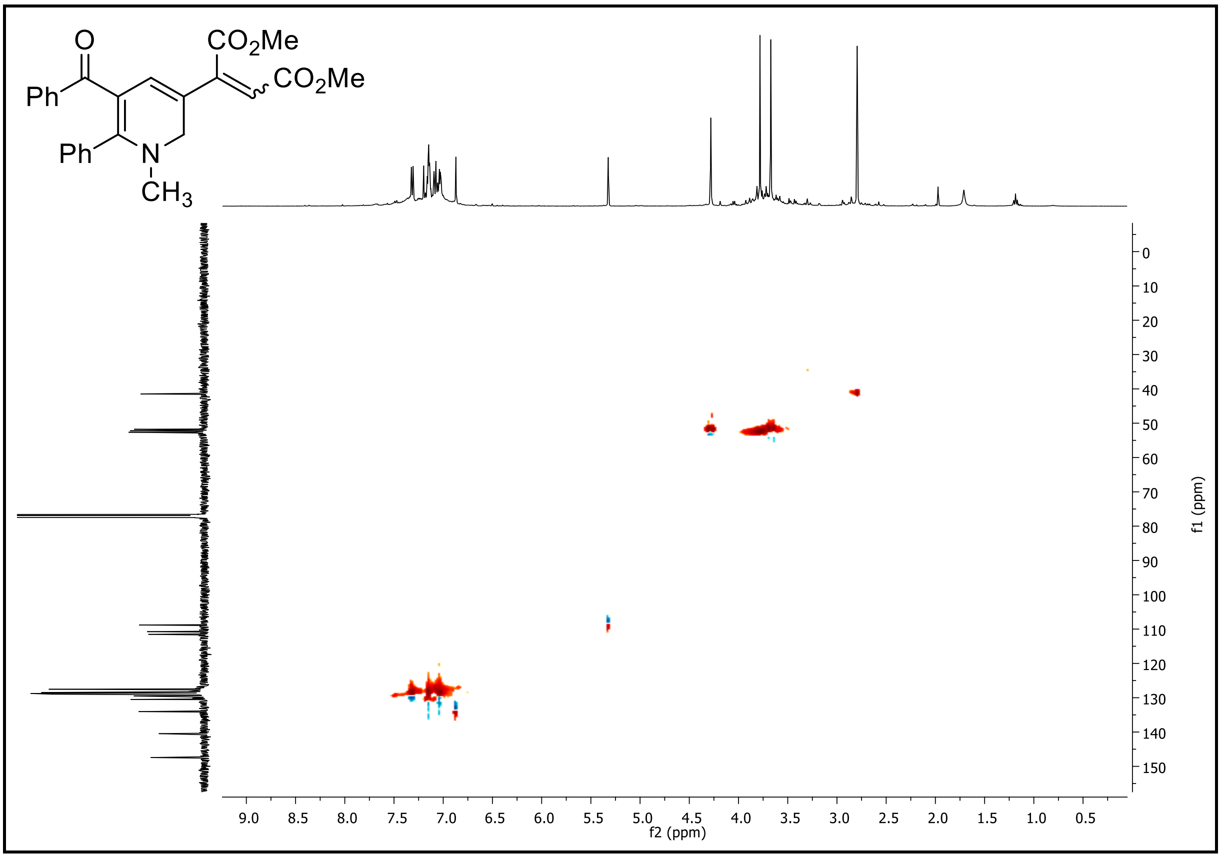

Figure S106. $^{1}H/^{13}C$ HSQC (400/100 MHz, $CDCl_3$) spectrum of compound **6a**.

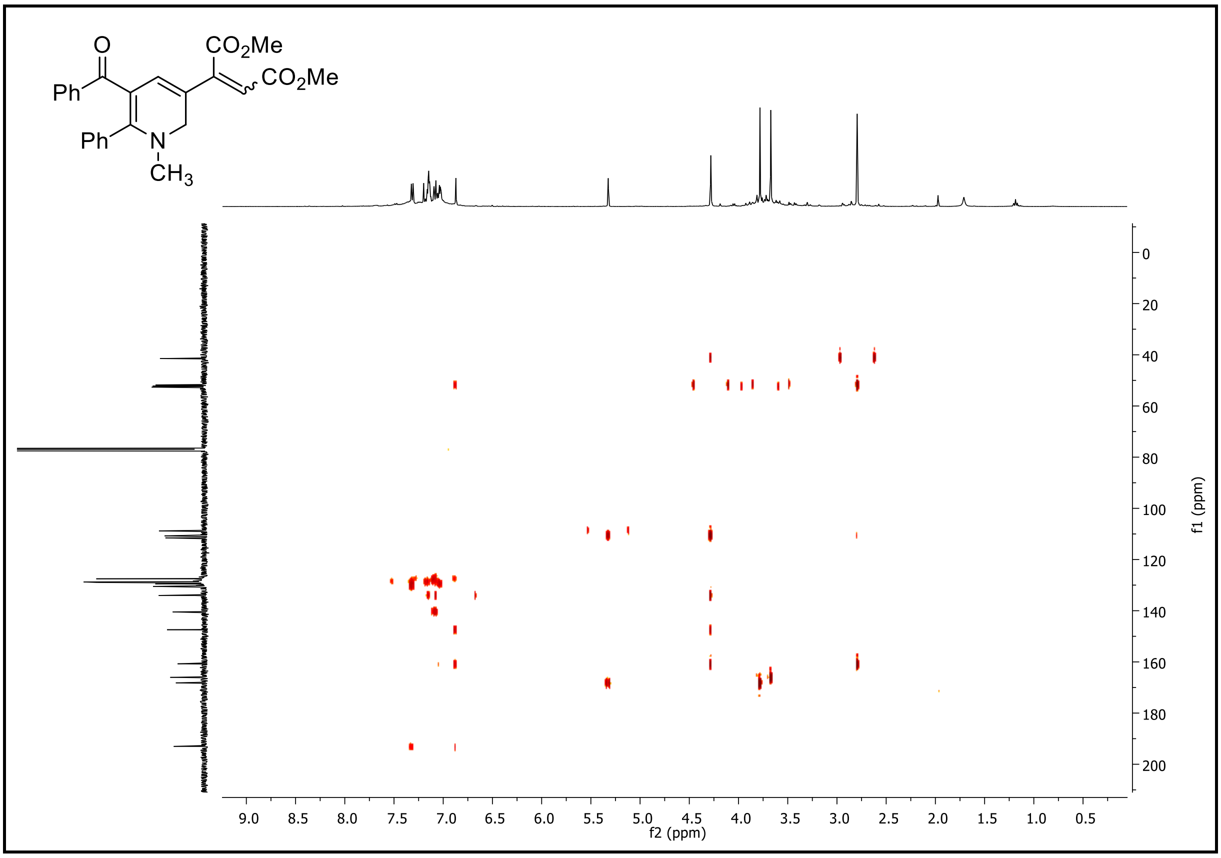

Figure S107. $^{1}H/^{13}C$ HMBC (400/100 MHz, $CDCl_3$) spectrum of compound **6a**.

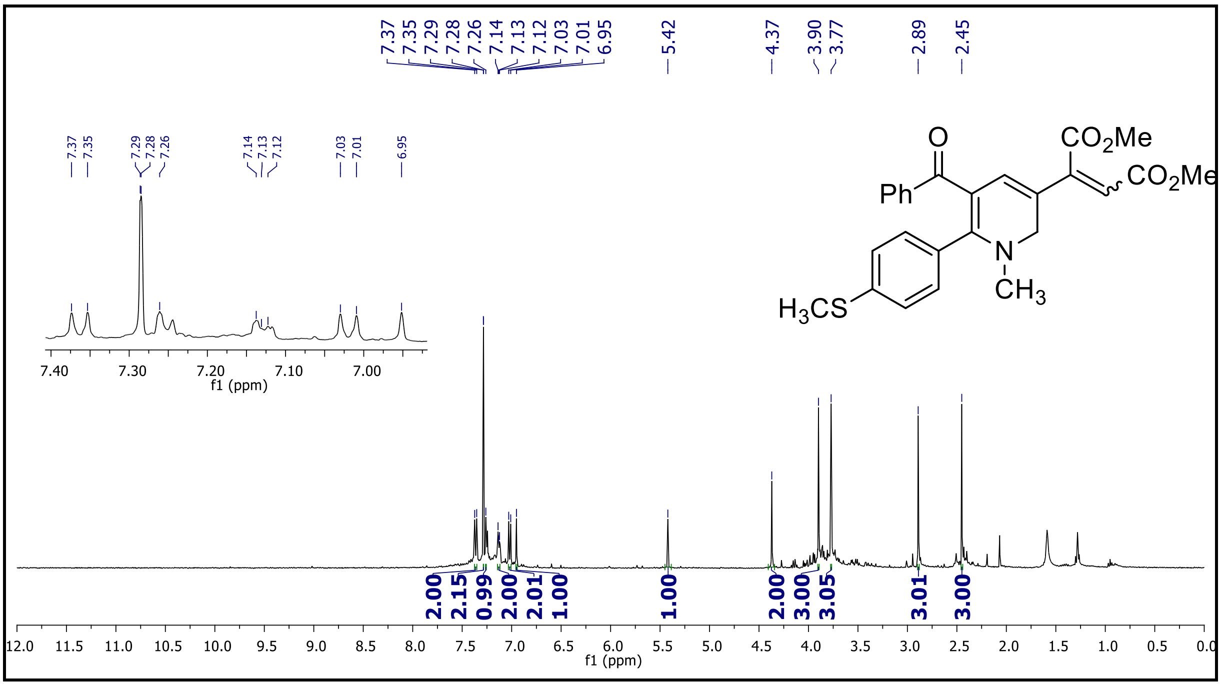

Figure S108. ^{1}H NMR (400 MHz, $CDCl_3$) spectrum of compound **6f**.

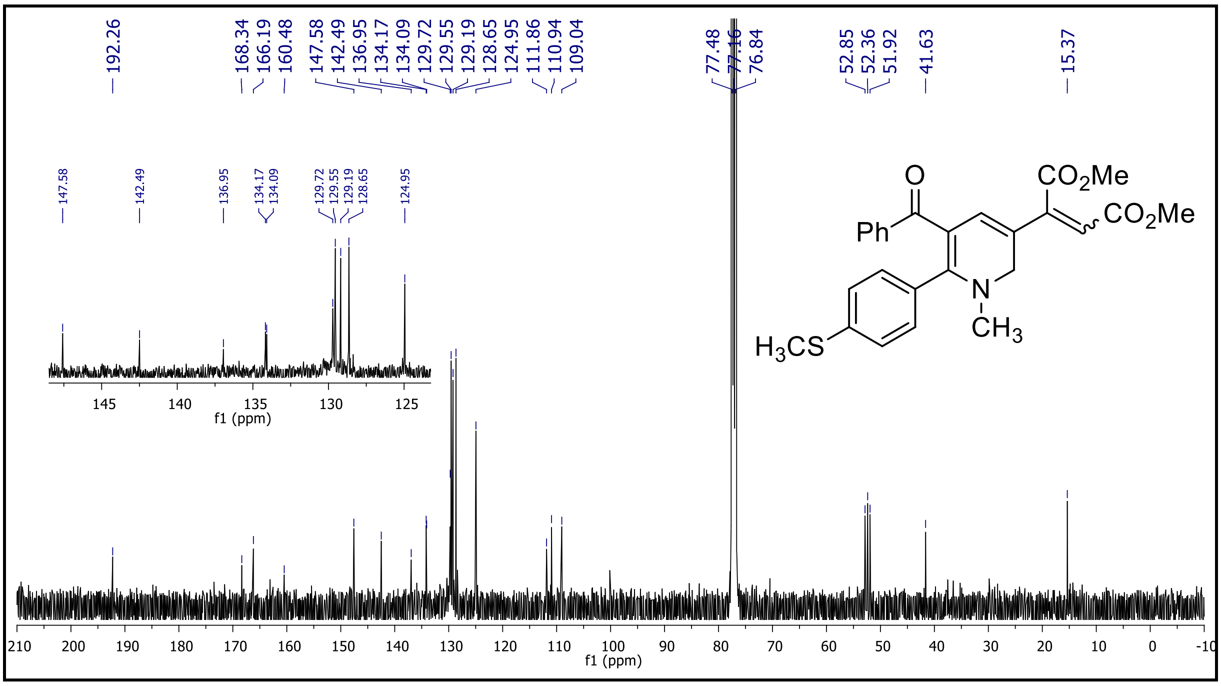

Figure S109. $^{13}C\{1H\}$ NMR (100 MHz, $CDCl_3$) spectrum of compound **6f**.

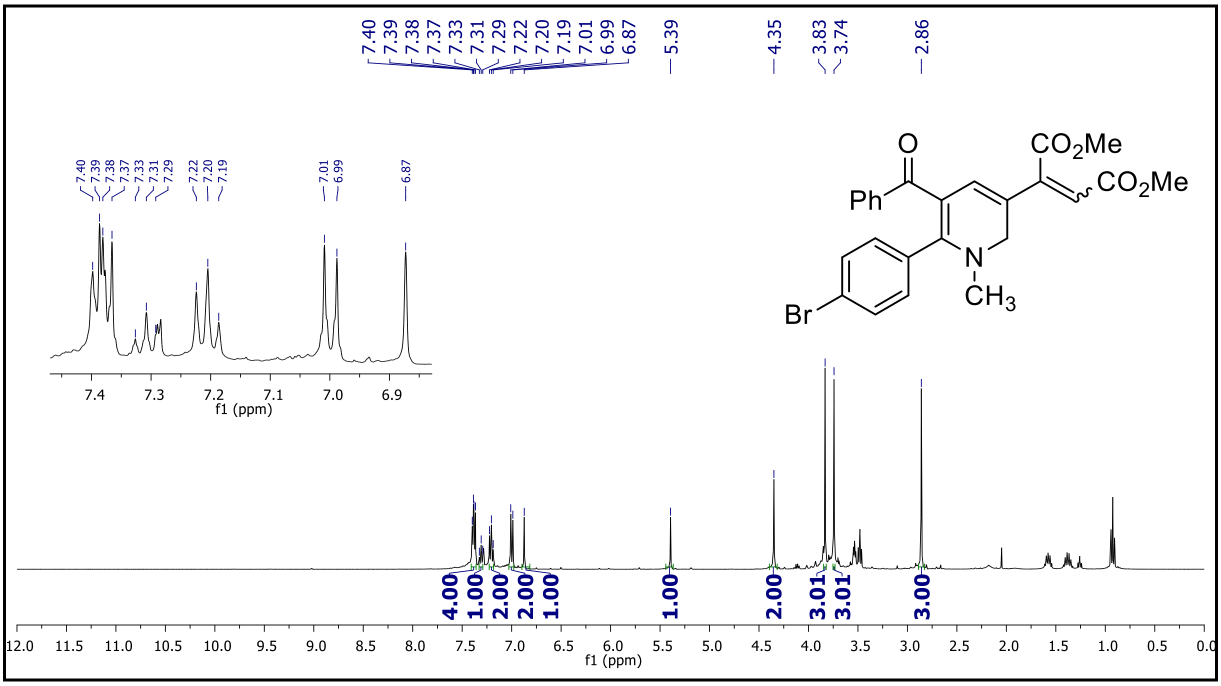

Figure S110. ^{1}H NMR (400 MHz, $CDCl_3$) spectrum of compound **6g**.

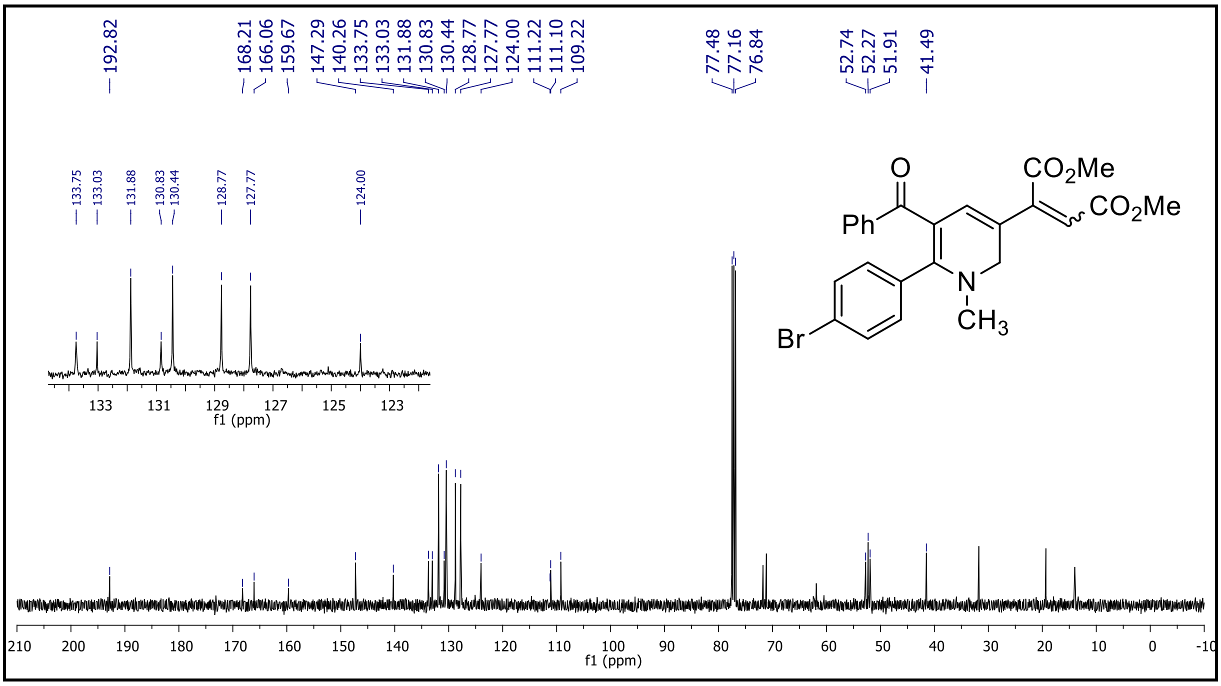

Figure S111. $^{13}C\{^1H\}$ NMR (100 MHz, $CDCl_3$) spectrum of compound **6g**.

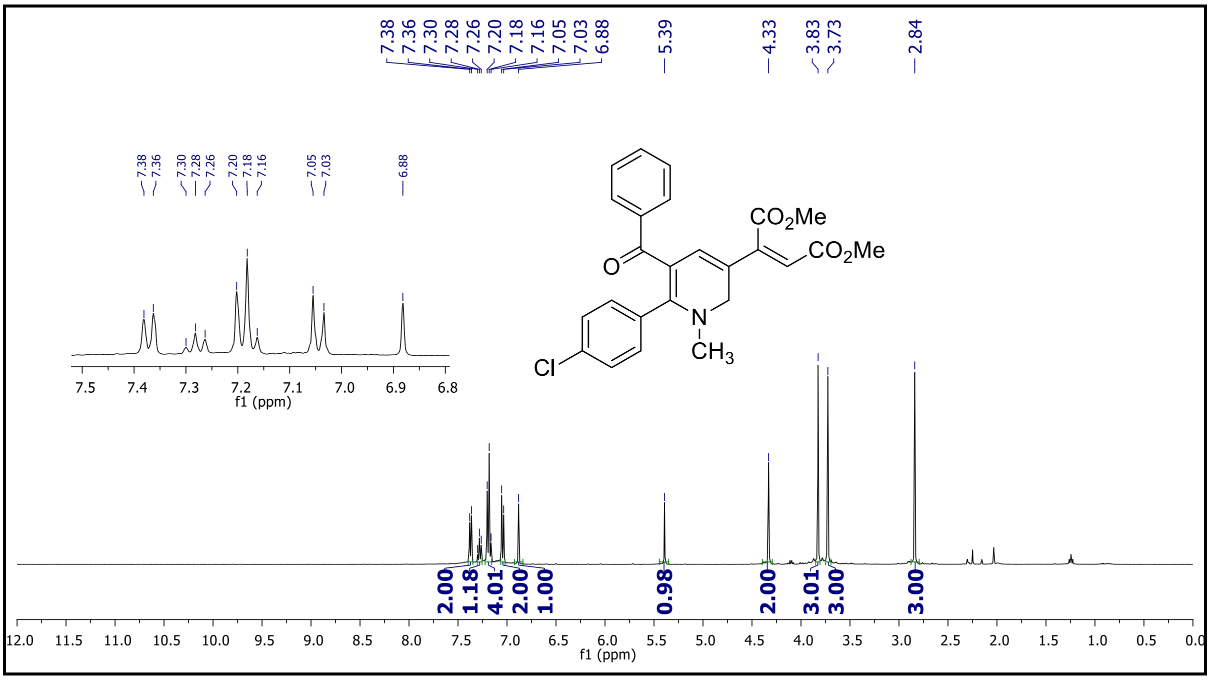

Figure S112. ^1H NMR (400 MHz, $CDCl_3$) spectrum of compound **6h**.

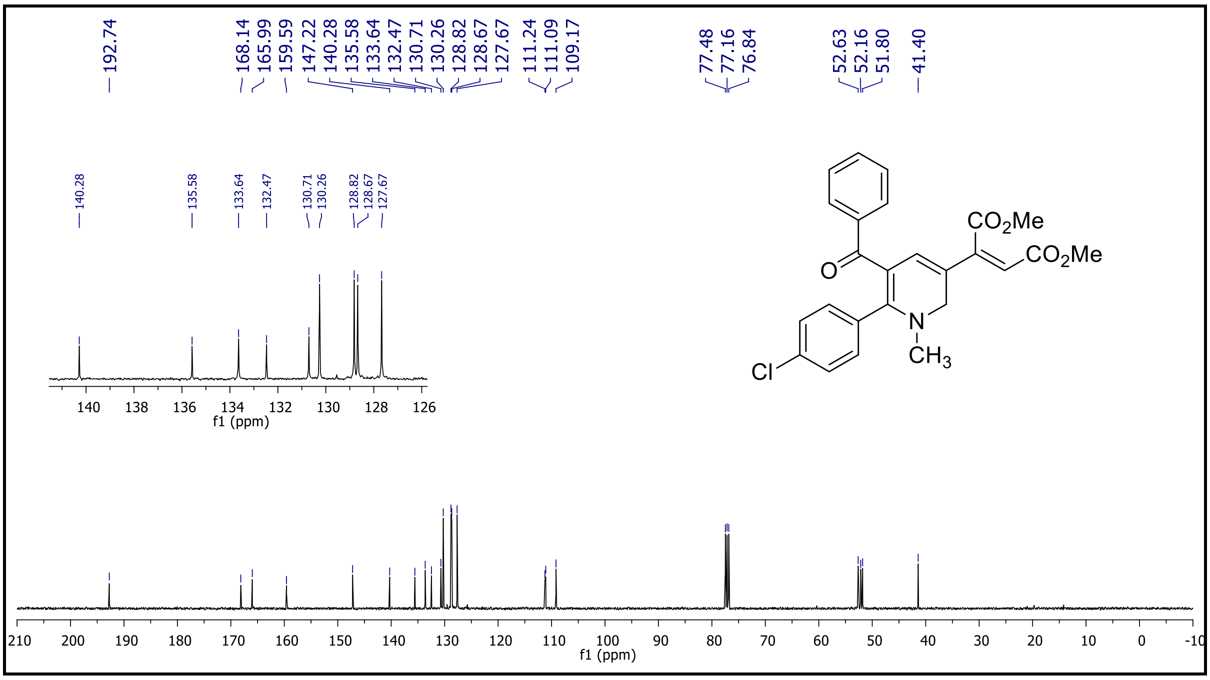

Figure S113. $^{13}C\{1H\}$ NMR (100 MHz, $CDCl_3$) spectrum of compound **6h**.

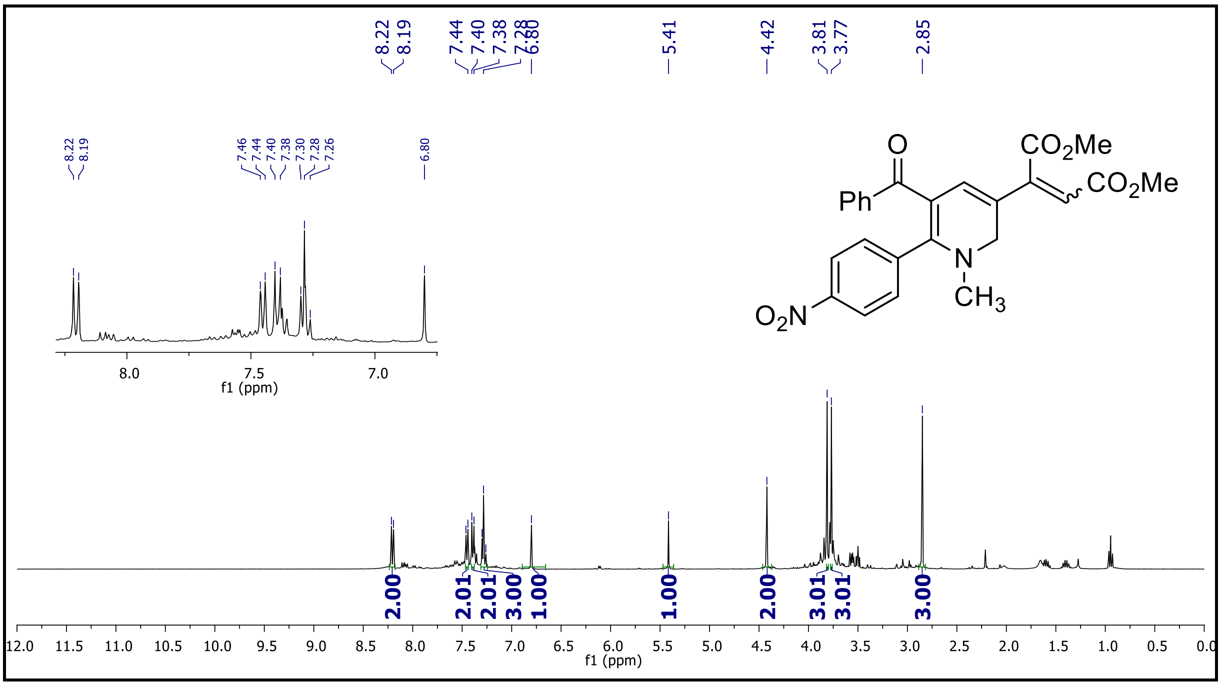

Figure S114. ^{1}H NMR (400 MHz, $CDCl_3$) spectrum of compound **6l**.

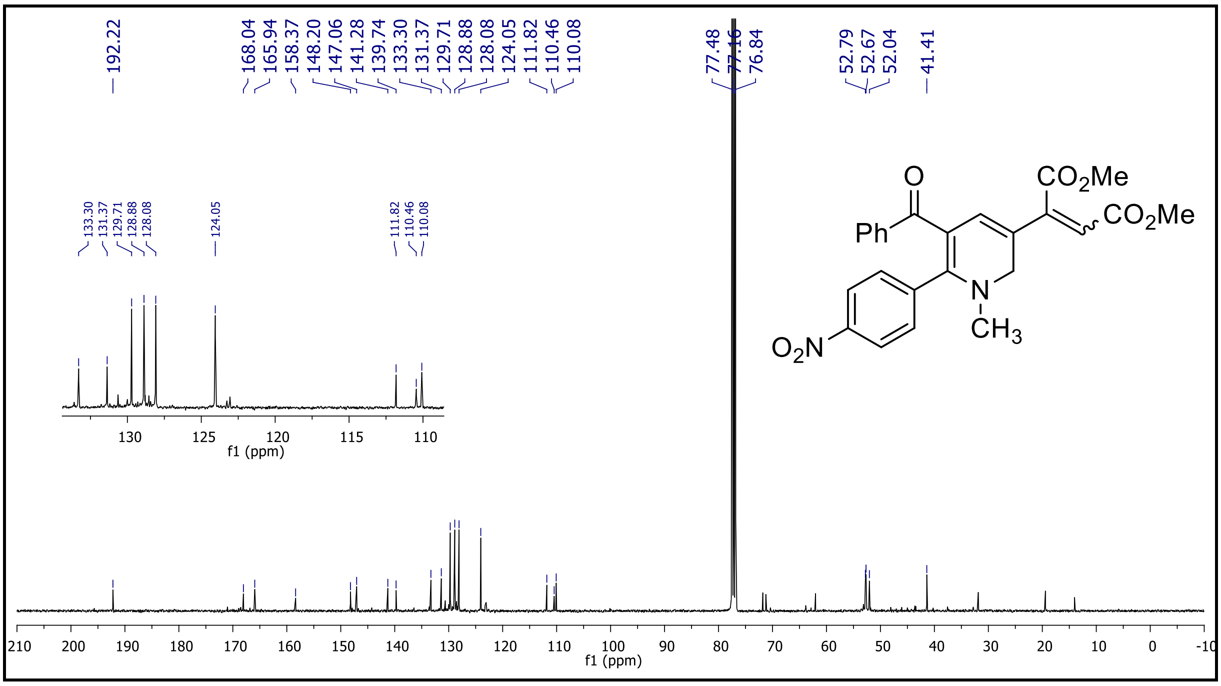

Figure S115. $^{13}C\{^{1}H\}$ NMR (100 MHz, $CDCl_3$) spectrum of compound **6l**.

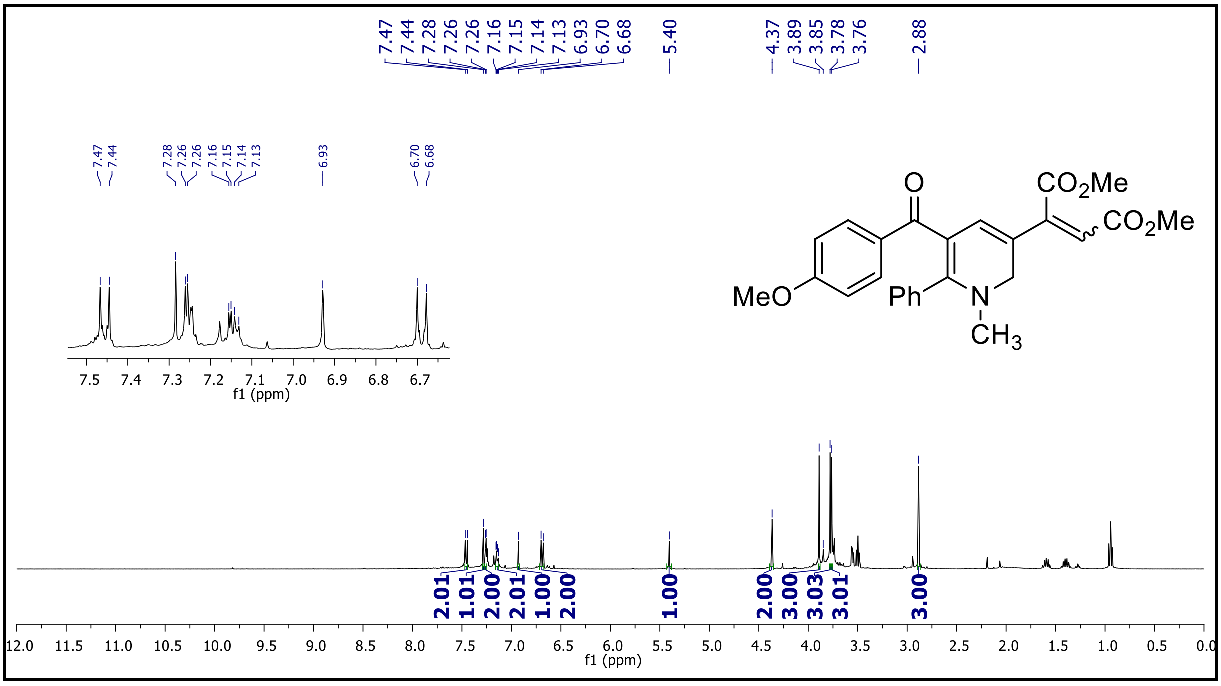

Figure S116. ^{1}H NMR (400 MHz, $CDCl_3$) spectrum of compound **6p**.

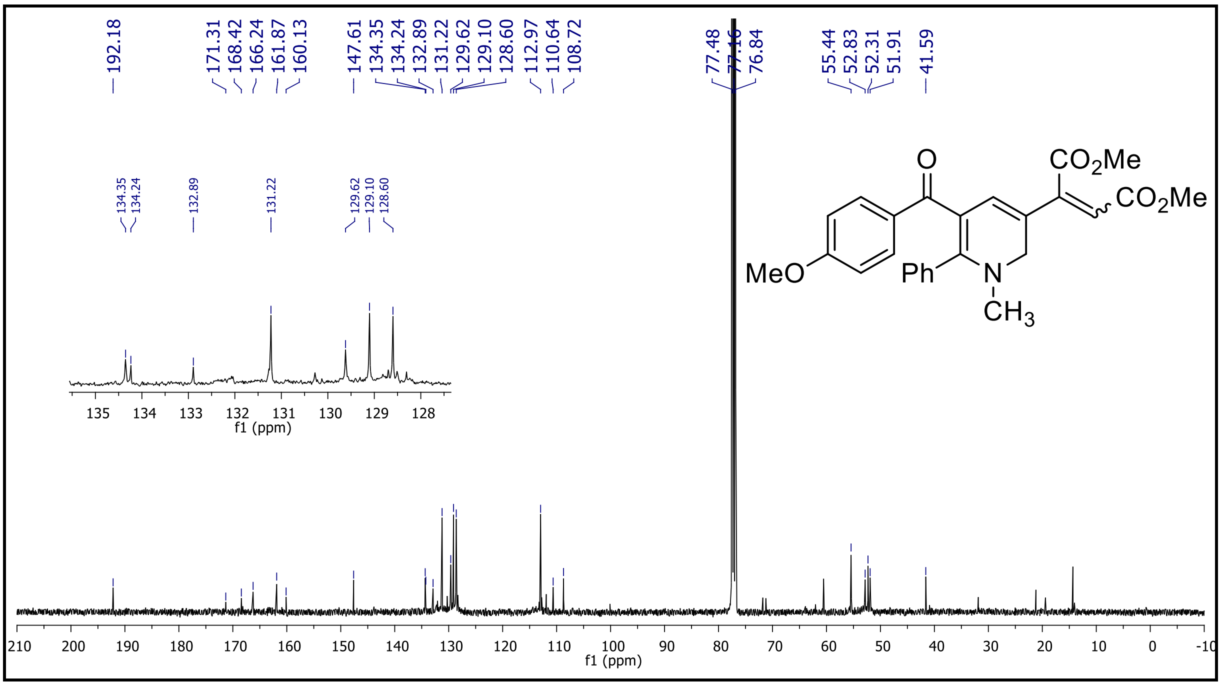

Figure S117. ^{13}C{1H} NMR (100 MHz, CDCl$_3$) spectrum of compound **6p**.

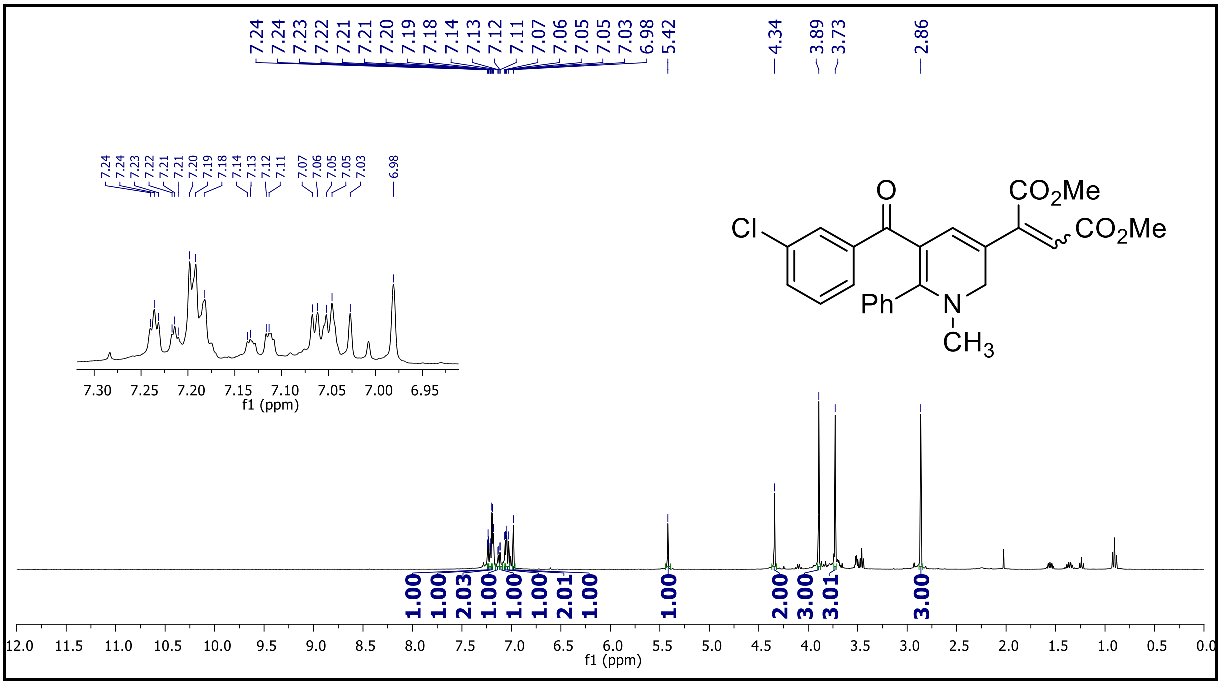

Figure S118. ^{1}H NMR (400 MHz, CDCl$_3$) spectrum of compound **6q**.

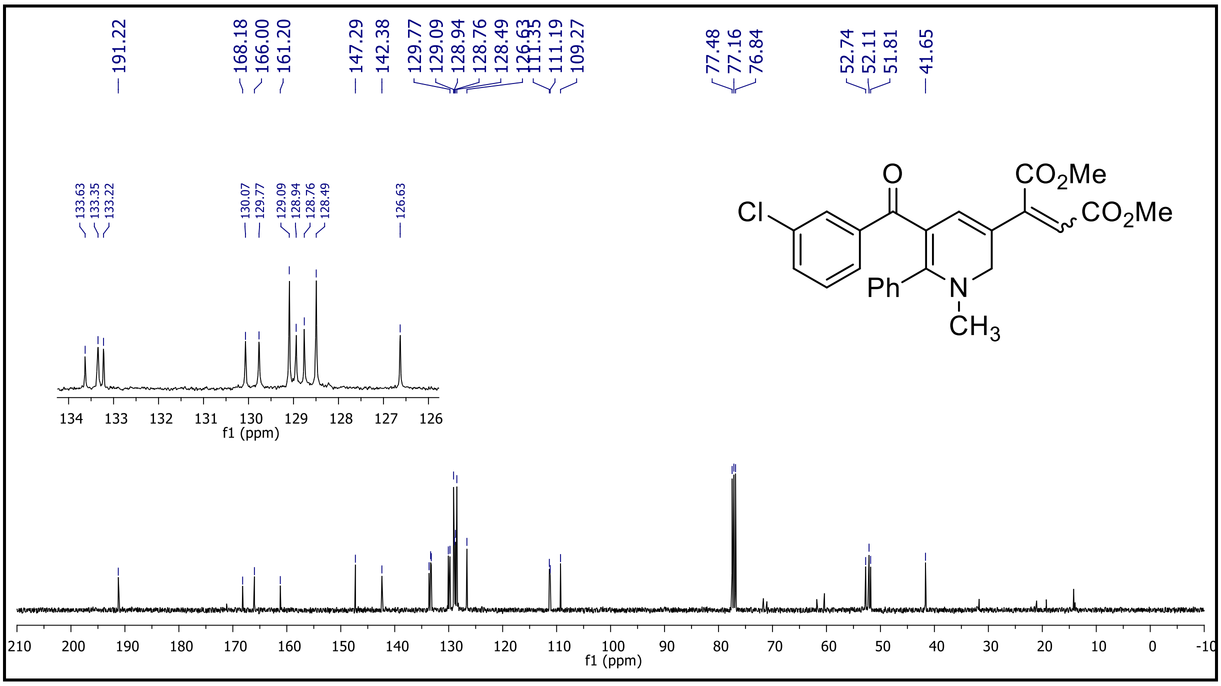

Figure S119. ${}^{13}C\{{}^{1}H\}$ NMR (100 MHz, $CDCl_3$) spectrum of compound **6q**.

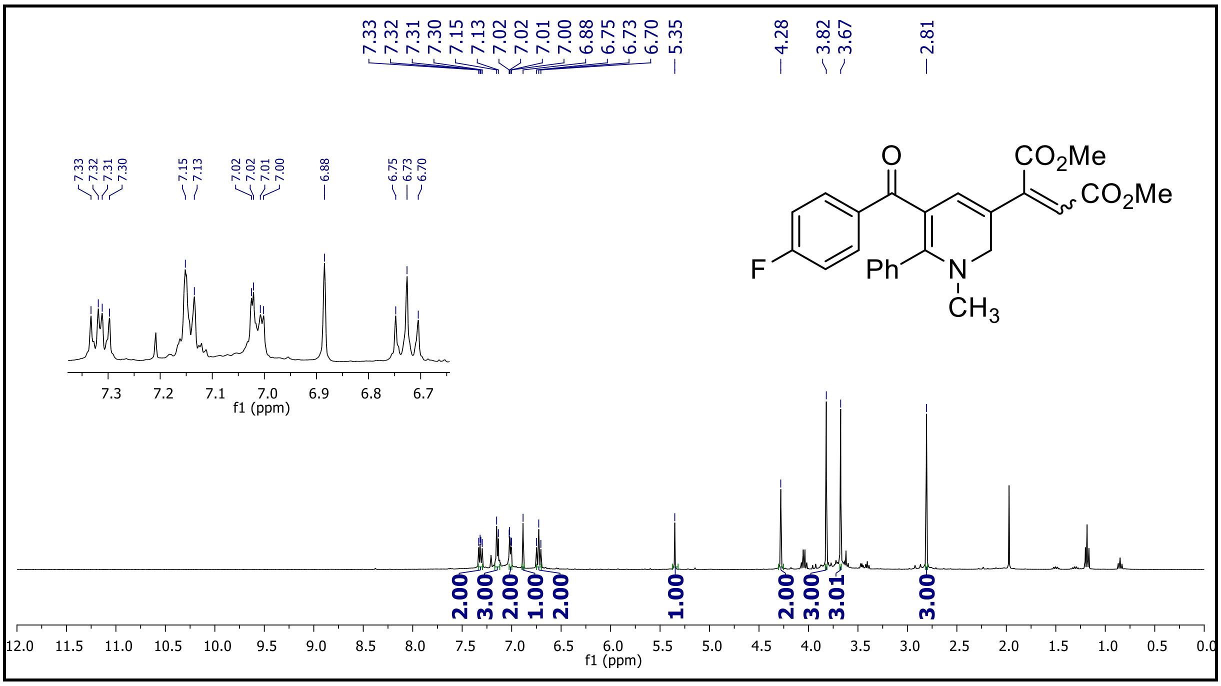

Figure S120. ${}^{1}H$ NMR (400 MHz, $CDCl_3$) spectrum of compound **6r**.

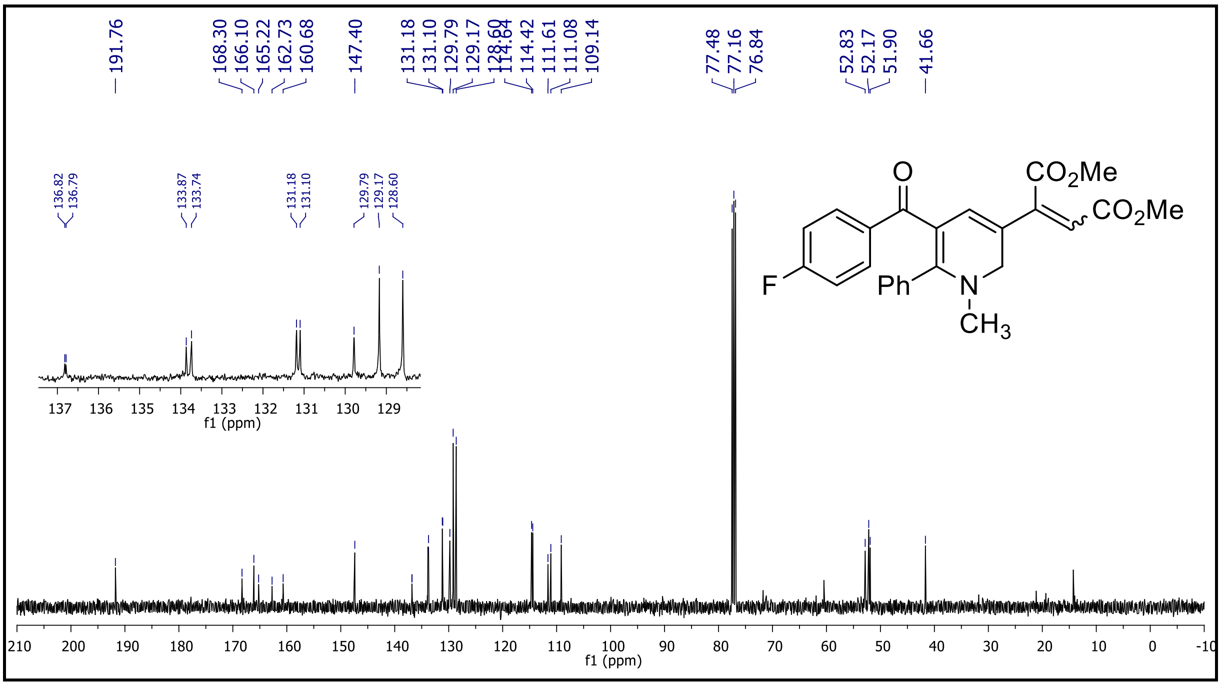

Figure S121. $^{13}C\{^{1}H\}$ NMR (100 MHz, $CDCl_3$) spectrum of compound **6r**.

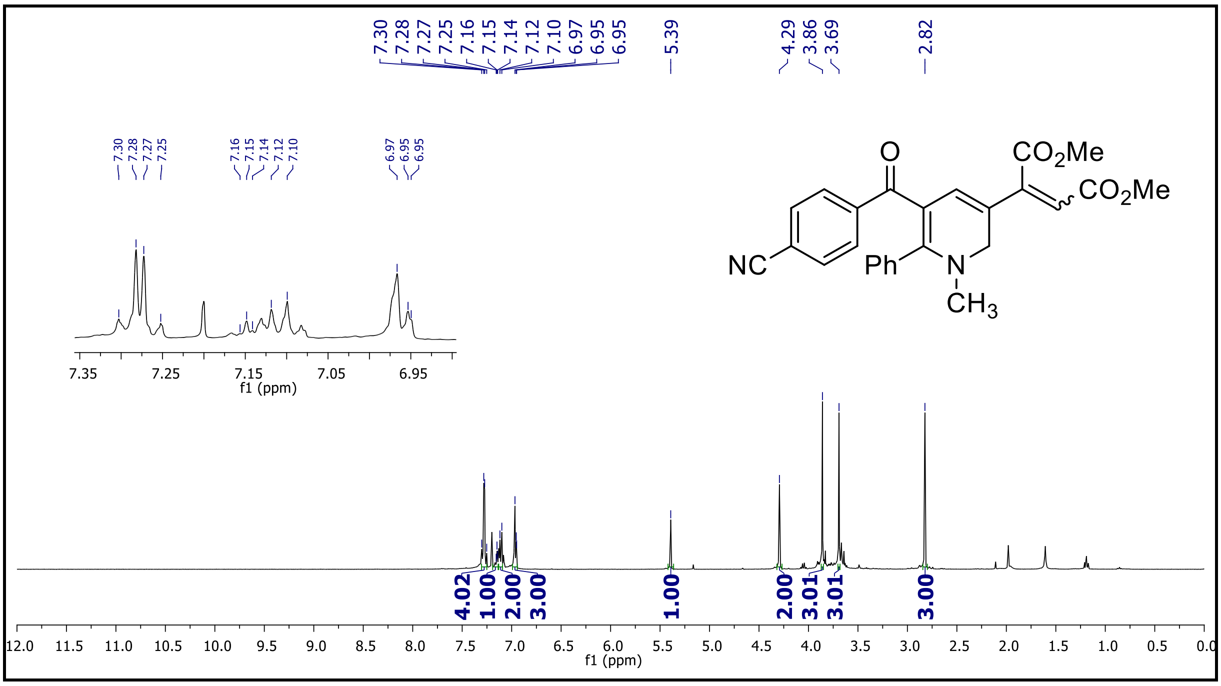

Figure S122. ^{1}H NMR (400 MHz, $CDCl_3$) spectrum of compound **6s**.

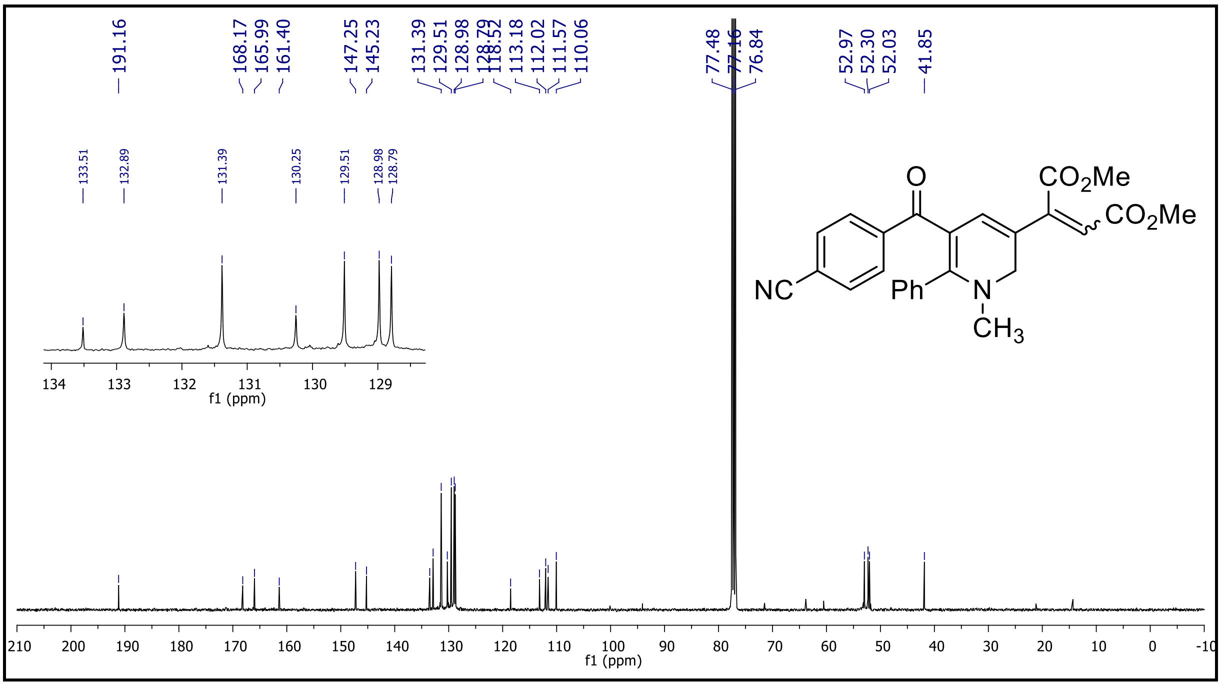

Figure S123. $^{13}C\{^{1}H\}$ NMR (100 MHz, $CDCl_3$) spectrum of compound **6s**.

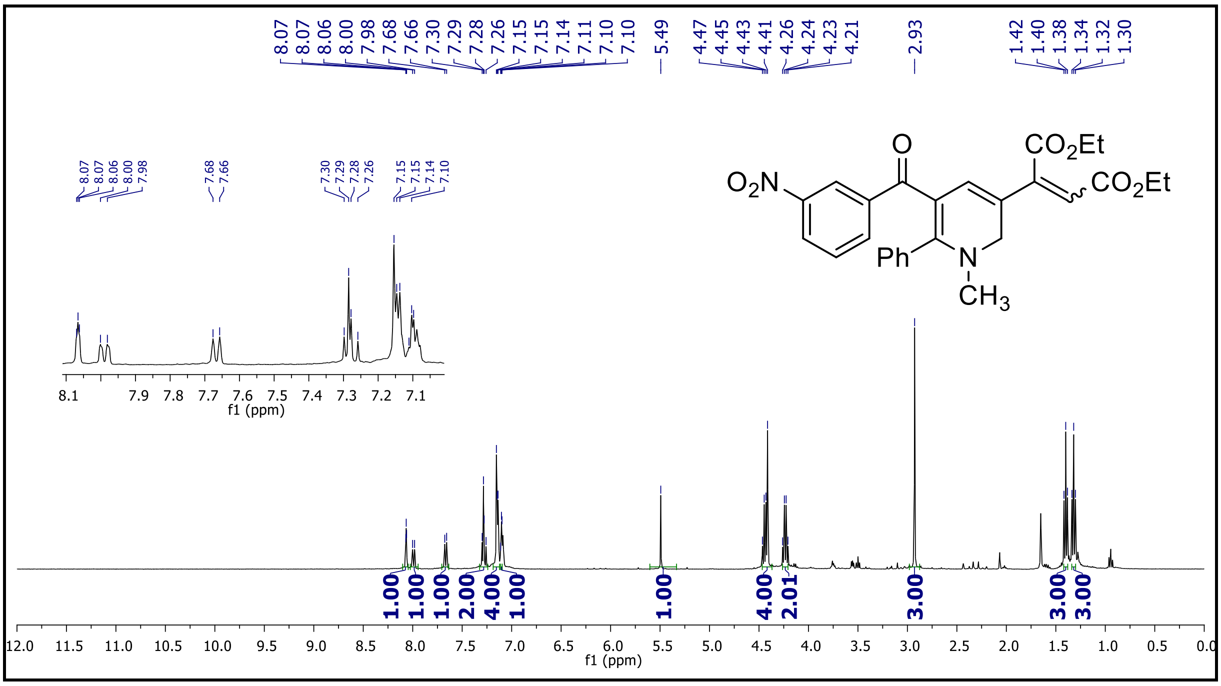

Figure S124. ^{1}H NMR (400 MHz, $CDCl_3$) spectrum of compound **6t'**.

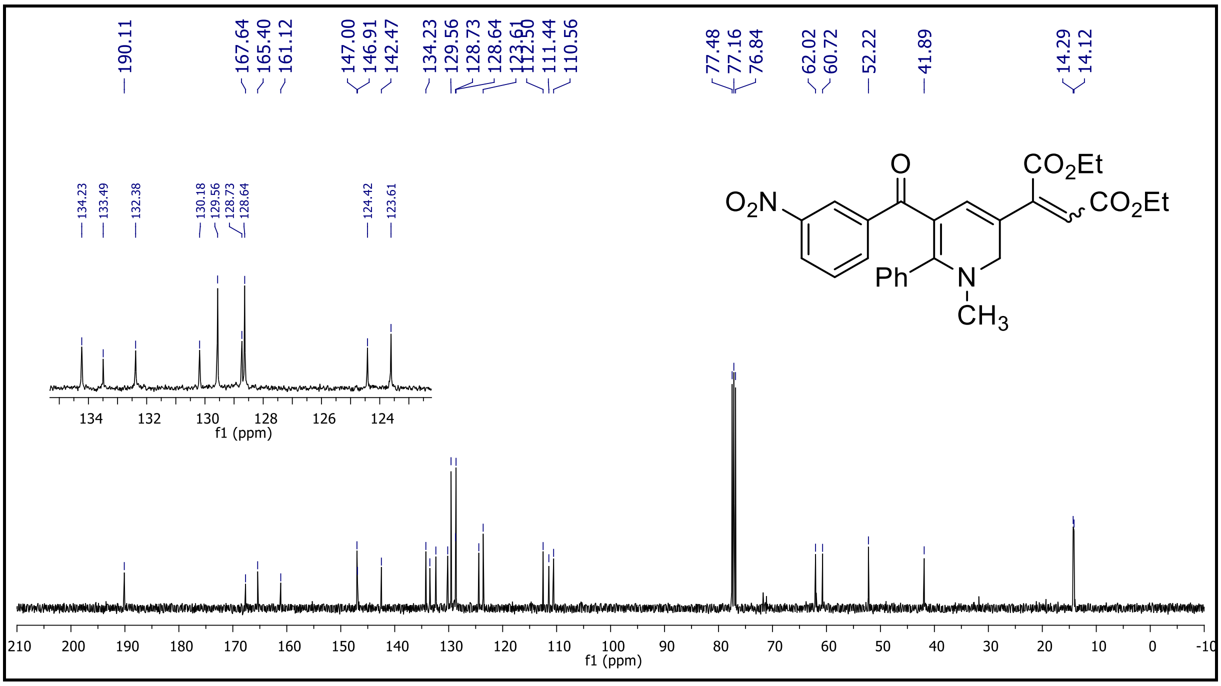

Figure S125. $^{13}C\{^1H\}$ NMR (100 MHz, $CDCl_3$) spectrum of compound **6t'**.

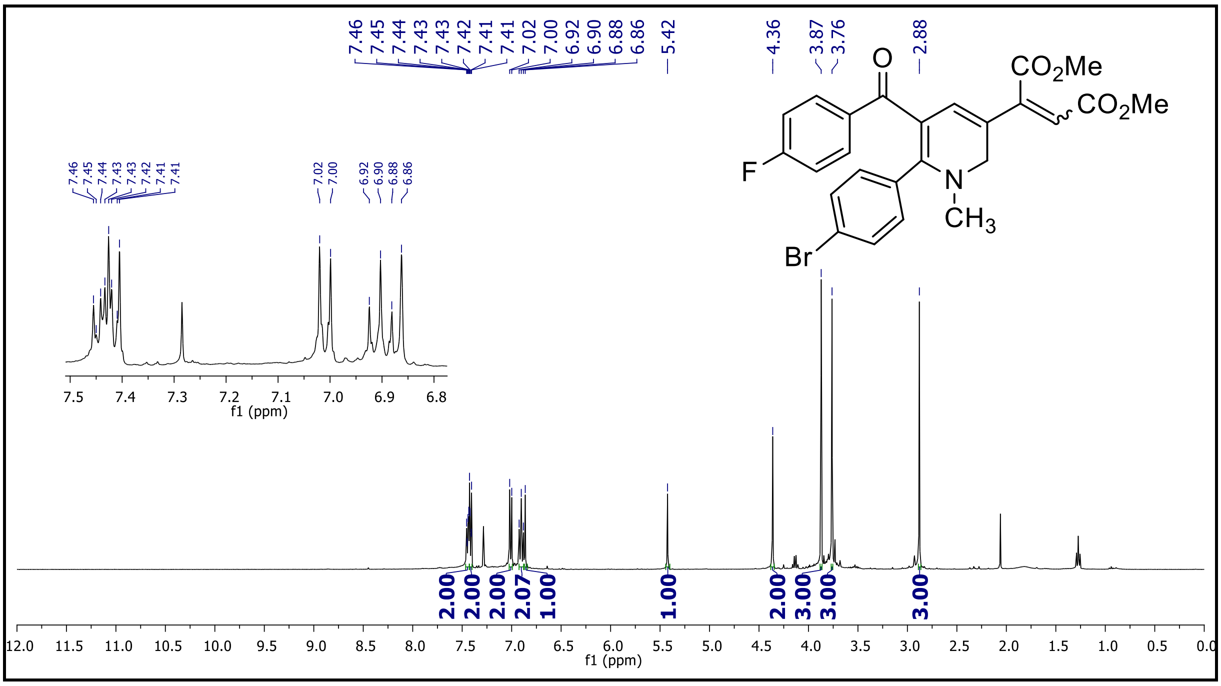

Figure S126. ^1H NMR (400 MHz, $CDCl_3$) spectrum of compound **6u**.

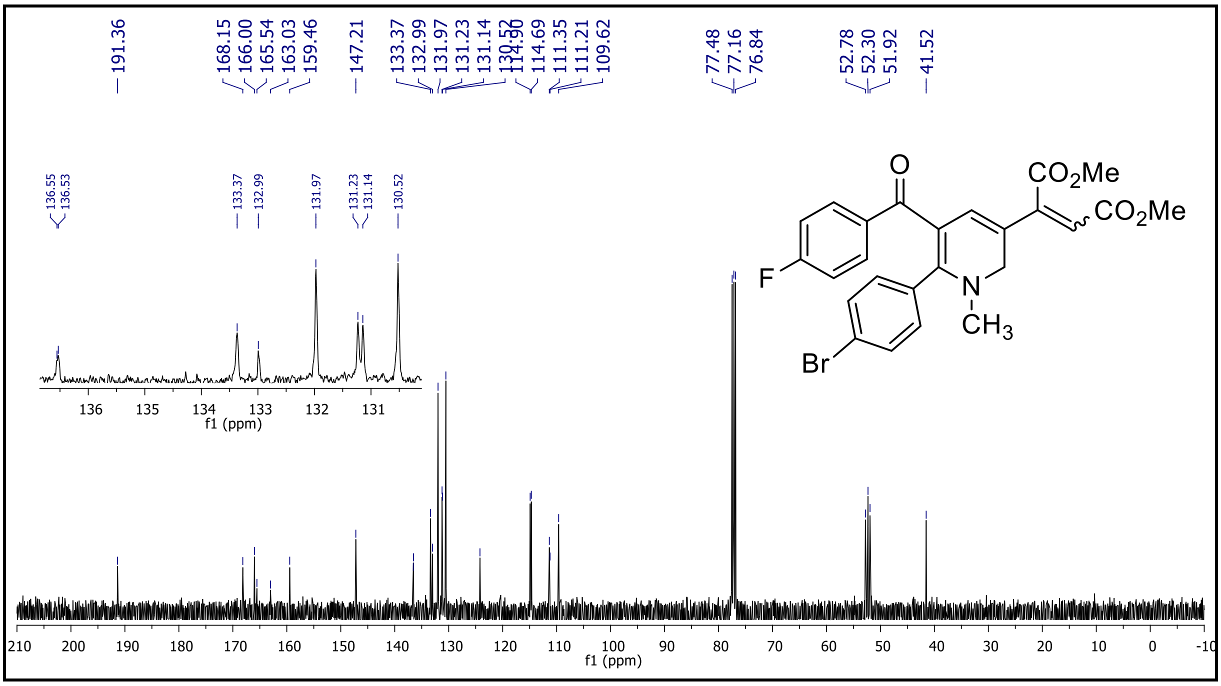

Figure S127. $^{13}C\{^{1}H\}$ NMR (100 MHz, $CDCl_3$) spectrum of compound **6u**.

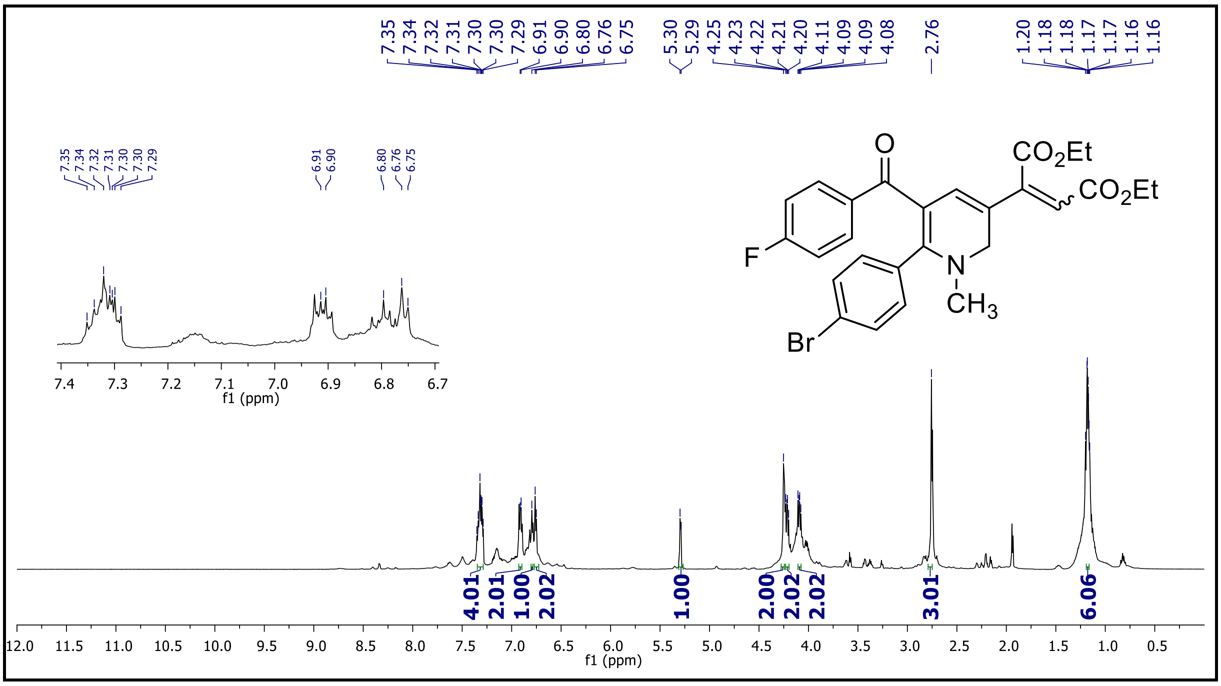

Figure S128. ^{1}H NMR (400 MHz, $CDCl_3$) spectrum of compound **6u'**.

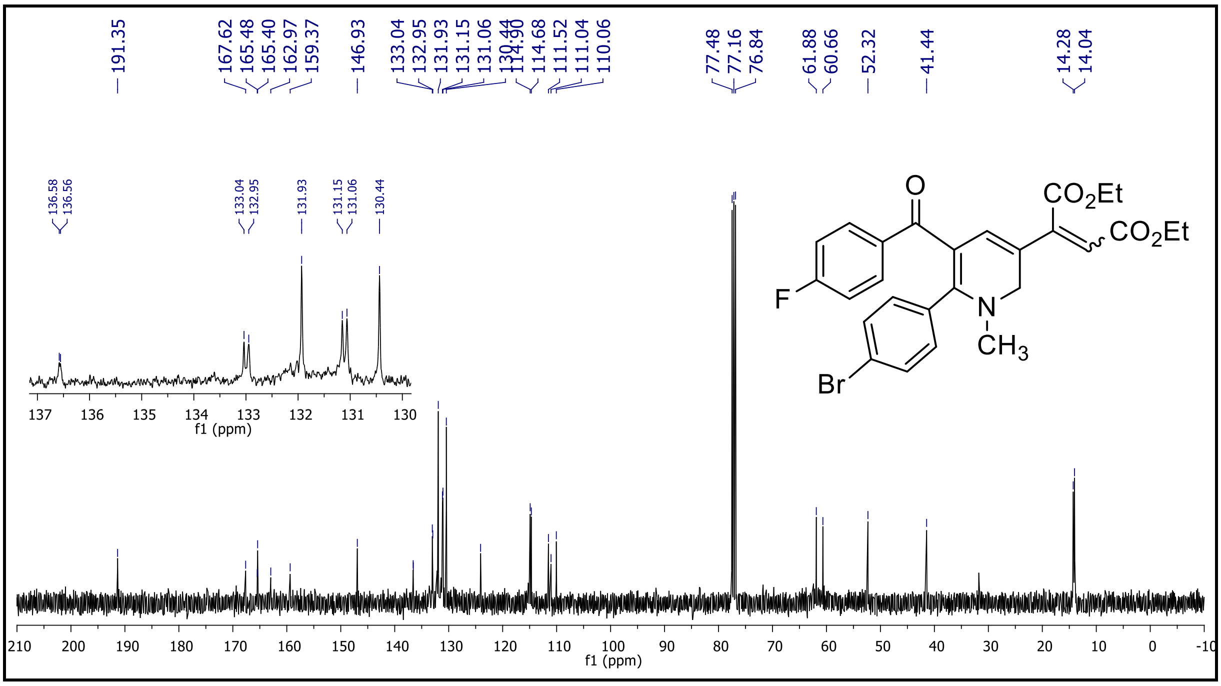

Figure S129. $^{13}C\{^1H\}$ NMR (100 MHz, $CDCl_3$) spectrum of compound **6u'**.

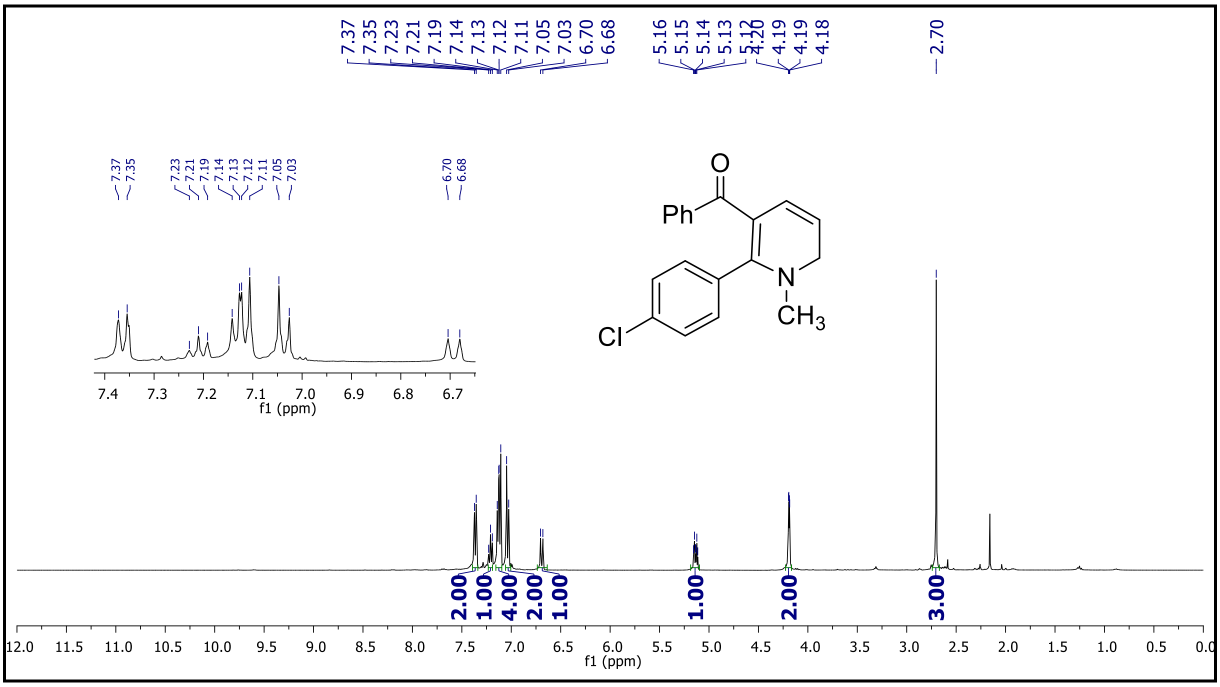

Figure S130. ^1H NMR (400 MHz, $CDCl_3$) spectrum of compound **8h**.

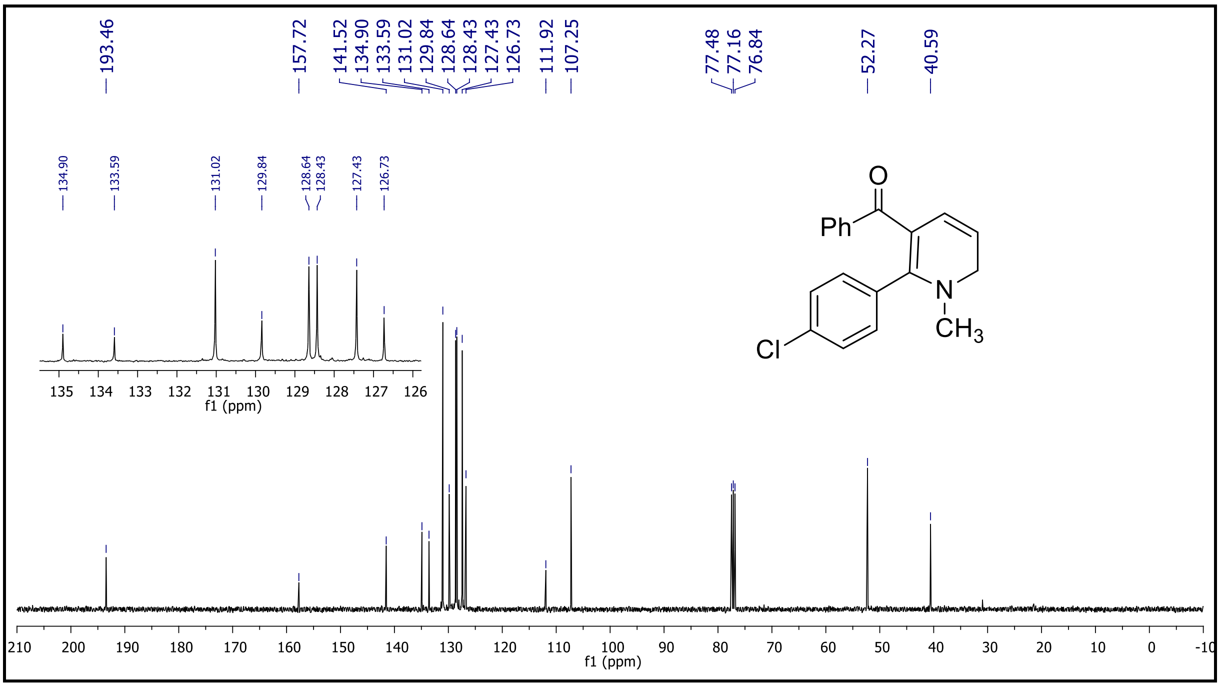

Figure S131. $^{13}C\{^{1}H\}$ NMR (100 MHz, $CDCl_3$) spectrum of compound **8h**.

.
